# Supplementary material for: 1,4-Aryl migration in ketene-derived enolates by a polar-radical-crossover cascade
Source: Nat Commun. 2022 Jun 2;13:3083. doi: 10.1038/s41467-022-30817-3 (PMC9163183; doi:10.1038/s41467-022-30817-3)
Supplement: Supplementary file 2 — Supplementary Information [file 41467_2022_30817_MOESM2_ESM.pdf]

Supplementary Information

1,4 Aryl Migration in Ketene-derived Enolates by a Polar-Radical-Cross-over Cascade

Niklas Radhoff and Armido Studer*

Organisch-Chemisches Institut, Westfälische Wilhelms-Universität, Corrensstrasse 40, 48149 Münster (Germany)

Email: studer@uni-muenster.de

Table of Contents

1. Supplementary Methods 1

 1.1 General Information 1

 1.2 Mechanistic studies 2

  1.2.1 Cross-over experiment 2

 1.3 General procedures 3

 1.4 Analytical Data of compounds 5

  1.4.1 Synthesis of sulfonamides 5

  1.4.2 Synthesis of ketenes 24

  1.4.3 Synthesis of α-quaternary amides 29

  1.4.4 Hydrolysis of α-quaternary amides 53

2. Supplementary Figures 54

 2.1 NMR Spectra of compounds 54

3. Supplementary References 142

1. Supplementary Methods

1.1 General Information

All reactions involving air or moisture sensitive compounds have been carried out in oven-dried glassware under an argon atmosphere using standard Schlenk-technique. Prior to use, anhydrous tetrahydrofuran (THF) used in the reactions was refluxed over sodium and freshly distilled from potassium afterwards. Anhydrous diethyl ether (Et_2O) was freshly distilled from sodium and potassium. All other anhydrous solvents used were purchased in extra-dry grade from *Acros Organics* and stored over molecular sieves. TEMPO-BF_4 was prepared following a procedure from the literature.[1] Unless stated otherwise, all other reagents used in the reactions are commercially available and have been used without further purification. They have been purchased from *Sigma Aldrich*, *Acros Organics*, *Alfa Aesar*, *ABCR*, *TCI*, *Fluorochem* and *BLDPharm*. Irradiation with blue LEDs was done with a *Kessil PR160-456 nm* lamp (456 nm, max. 40 W) and a *Kessil PR160-467 nm* lamp (467 nm, max. 40 W). Solvents used for chromatography and extractions were distilled prior to use. Flash column chromatography (FC) was conducted on *Merck* silica gel (40-63 µm) with an excess compressed air pressure up to 0.5 bar. For analytical thin layer chromatography (TLC) *Merck* silica gel 60 F254 plates have been used and compounds were detected using UV-light (254 nm) or $KMNO_4$-stain (1.5 g in 250 mL water, 5 g $NaHCO_3$). Medium pressure liquid chromatography (MPLC) was performed on an automatic flash-system by *Reveleris*® IES and on a *Büchi C-850 FlashPrep* device with commercially available 4 g, 12 g or 40 g *Reveleris*®-C_{18}-flash cartridges as the stationary phase. Detection was carried out by UV-absorption (λ = 210 nm, 230, 254, 320 nm) and electronic light scattering detection (ELSD) with isopropanol as carrier. Melting points were measured using a *Büchi Melting Point M-560* and are uncorrected. Infrared spectra were recorded using a *Digilab 3100 FT-IR Excalibur Series* and a *Jasco FT/IR-4600* spectrometer and the absorption bands are given in wave numbers ν (cm^{-1}). ^1H NMR (300 MHz, 400 MHz, 500 MHz and 600 MHz), ^{13}C NMR (75 MHz, 101 MHz, 126 MHz and 151 MHz) and ^{19}F NMR (282 MHz and 564 MHz) spectra were measured on a *Bruker Avance II 300*, *Bruker NEO 400*, *Agilent DD2 500* and *Agilent DD2 600* spectrometer. Chemical shifts (δ in ppm) were referenced to the solvent residual peak ($CDCl_3$: δ_H = 7.26 ppm and δ_c = 77.0 ppm; DMSO-d_6: δ_H = 2.50 ppm and δ_c = 39.5 ppm). The multiples of all signals were described as s (singlet), d (doublet), t (triplet), q (quartet), hept (heptet), m (multiplet) and combination of the above. HRMS (ESI) spectra were recorded on a *Bruker Daltonics Micro Tof*, *Thermo Fisher Scientific LTQ Orbitrap XL* and *Thermo Fisher Scientific Orbitrap Velos Pro* spectrometer. The peaks are given in *m/z*.

1.2 Mechanistic studies

1.2.1 Cross-over experiment

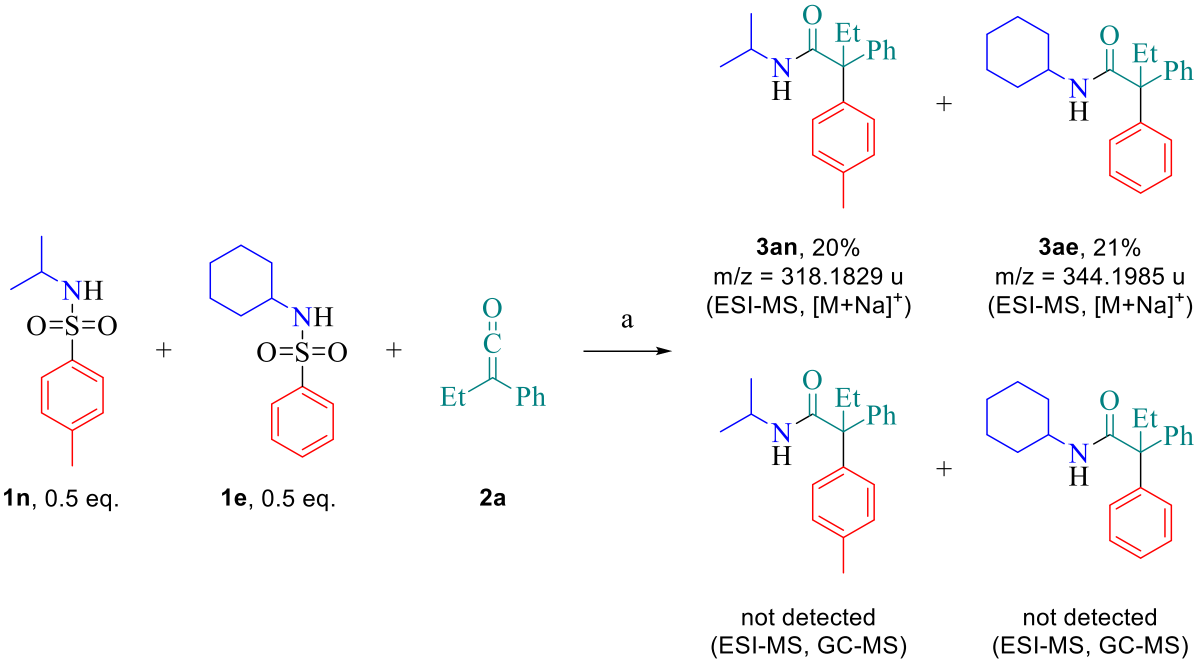

Supplementary Figure 1: Cross-over experiment. Conditions a: 1) n-BuLi (1.1 eq.), rt, 30 min, 2) $FcBF_4$ (1.0 eq.), rt, 15 min, 3) Addition of ketene **2a** (1.5 eq.) over 30 minutes by syringe pump, rt, 4) rt, overnight. Products were analyzed by GC-MS and ESI-MS, yields were determined by ^1H NMR spectroscopy using 1,3,5-trimethoxybenzene as an internal standard.

<u>Procedure:</u> To an oven-dried Schlenk-tube was added sulfonamide **1n** (21.3 mg, 0.100 mmol, 0.5 eq.) and anhydrous Et_2O (7.5 mL). *n*-Butyllithium (1.6 M-solution in hexanes, 69 µL, 0.11 mmol, 0.55 eq.) was added at room temperature and the mixture was stirred for 30 minutes. Ferrocenium tetrafluoroborate (27.3 mg, 0.100 mmol, 0.5 eq.) was added, stirring was continued for further 15 minutes and ketene **2a** (20 µL, 0.15 mmol, 0.75 eq.) dissolved in anhydrous Et_2O (2.5 mL) was added by syringe pump over 30 minutes. In a second oven-dried Schlenk-tube sulfonamide **1e** (23.9 mg, 0.100 mmol, 0.5 eq.) was reacted with *n*-butyllithium (1.6 M-solution in hexanes, 69 µL, 0.11 mmol, 0.55 eq.), ferrocenium tetrafluoroborate (27.3 mg, 0.100 mmol, 0.5 eq.) and ketene **2a** (20 µL, 0.15 mmol, 0.75 eq.) analogously. After ketene addition, the both solutions were unified by transfer cannula and stirred overnight. The crude reaction mixture was analyzed by GC-MS, ESI-MS and ^1H NMR spectroscopy (1,3,5-trimethoxybenzene as internal standard).

1.3 General procedures

General Procedure (GP1) for the preparation of *N*-alkylsulfonamides (1a-ab):

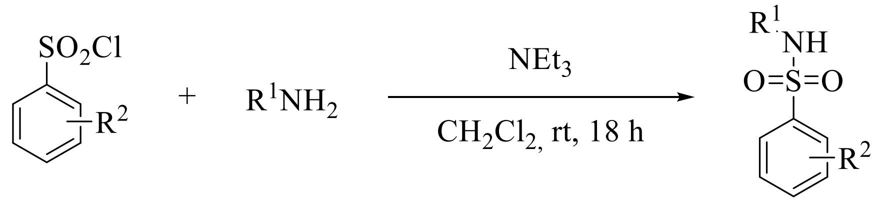

N-Alkylsulfonamides were prepared following a modified procedure from the literature.[2] A solution of an arylsulfonyl chloride (1.0 eq.) and NEt_3 (2.0 eq.) in anhydrous CH_2Cl_2 (1.0 M) was cooled to 0 °C. After adding a primary amine (1.2 eq.), the solution was allowed to warm to room temperature and stirred until reaction completion was indicated by TLC. After quenching with water, the phases were separated and the aqueous phase was extracted with CH_2Cl_2 (3 x). The combined organic phases were dried over $MgSO_4$ and concentrated *in vacuo*. Purification by flash column chromatography (pentane/EtOAc) led to isolation of the desired *N*-alkylsulfonamides as colourless solids and oils.

General Procedure (GP2) for the preparation of ketenes (2a-l):

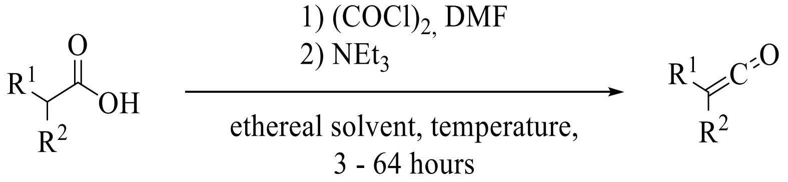

Ketenes were prepared following a representative procedure from the literature.[3] Carboxylic acids (1.0 eq.) were dissolved in anhydrous CH_2Cl_2 (approx. 0.1 M) and treated with oxalyl chloride (1.5 eq.) and a few drops of DMF at room temperature. When gas formation ceased, the solvent was removed *in vacuo* and the residue was dissolved in anhydrous Et_2O or THF (depending on the boiling point of the desired ketene, approx. 0.1 M). Triethylamine (4.0 eq.) was added slowly and the reaction mixture was stirred for 3 – 64 hours at the specified temperature. The ammonium salts were removed by filtration under inert gas atmosphere, the solvent was removed under reduced pressure and the crude ketene purified by short-path distillation. The desired ketenes were obtained as yellow oils.

General Procedure (GP3) for the *α*-arylation (3a-la):

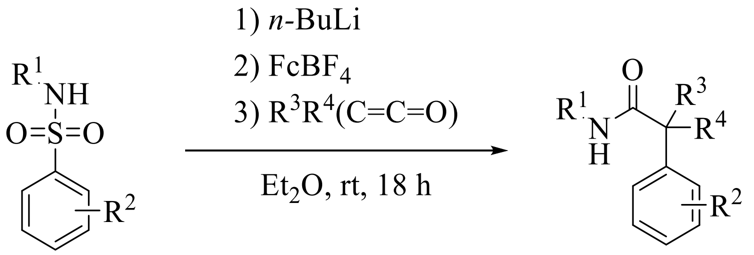

Sulfonamide (1.0 eq.) was placed into a Schlenk tube, which was subsequently evacuated and backfilled with argon three times. Anhydrous Et_2O (0.013 M) and *n*-butyllithium (1.6 M solution in hexanes, 1.1 eq.) were added at room temperature. After stirring for 30 minutes, ferrocenium tetrafluoroborate ($FcBF_4$, 1.0 eq.) was added and stirring was continued for further 15 minutes. Subsequently, the ketene (0.06 M-solution in Et_2O, 1.5 eq.) was added dropwise over 30 minutes by syringe pump. The reaction mixture was stirred at room temperature overnight and then filtered through a short path of silica (eluent: EtOAc). Purification by *reversed-phase* MPLC (MeOH/water, gradient from 40% to 90%) led to isolation of the desired amides as colourless oils and solids.

Regeneration (GP4) of ferrocenium tetrafluoroborate ($FcBF_4$):

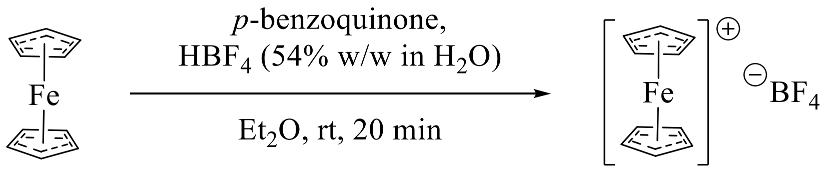

Ferrocenene was oxidized to the title compound following a procedure from the literature.[4] To a stirred solution of *p*-benzoquinone (222 mg, 2.06 mmol, 0.25 eq.) in Et_2O (11 mL) was added HBF_4 (54% w/w in H_2O, 2.81 mL, 17.3 mmol, 2.1 eq.) and then ferrocene (1.53 g, 8.22 mmol, 1.0 eq.) dissolved in Et_2O (24 mL). The yellow solution turned blue immediately and was stirred for further ten minutes at room temperature open to air. The blue suspension was allowed to settle for 10 minutes and then filtered. The crude product was obtained as a deep blue solid. Dissolution in acetone (48 mL) and filtration after addition of Et_2O (70 mL) gave the title compound as deep blue needles (1.08 g, 3.83 mmol, 62%).

1.4 Analytical Data of compounds

1.4.1 Synthesis of sulfonamides

***N*-Isopropylbenzenesulfonamide (1a):**

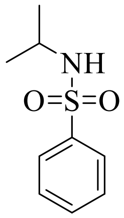

The title compound was prepared following the general procedure **GP1** using benzenesulfonyl chloride (2.56 mL, 20.0 mmol, 1.0 eq.), NEt_3 (5.54 mL, 40.0 mmol, 2.0 eq.) and isopropylamine (2.06 mL, 24.0 mmol, 1.2 eq.) in CH_2Cl_2 (10 mL). Purification by flash column chromatography (EtOAc/Pentane, *v/v* = 1:4) gave the title compound as a colourless oil (3.25 g, 17.7 mmol, 88%).

^{1}H NMR (300 MHz, $CDCl_3$, 300 K): δ_H (ppm) = 7.92 – 7.88 (m, 2H), 7.60 – 7.48 (m, 3H), 4.85 (d, *J* = 7.6 Hz, 1H), 3.54 – 3.38 (m, 1H), 1.07 (d, *J* = 6.5 Hz, 6H).

^{13}C NMR (75 MHz, $CDCl_3$, 300 K): δ_C (ppm) = 141.1 (C_q), 132.5 (*C*H), 129.1 (*C*H), 127.0 (*C*H), 46.1 (*C*H), 23.7 (CH_3).

HRMS (ESI) *m/z* = 222.0559 calcd. for $[C_9H_{13}NO_2SNa]^+$ $[M+Na]^+$, found: 222.0563.

The analytical data are consistent with those reported in literature.[5]

***N*-Methylbenzenesulfonamide (1b):**

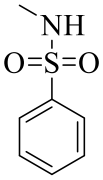

The title compound was prepared following the general procedure **GP1** using benzenesulfonyl chloride (1.28 mL, 10.0 mmol, 1.0 eq.), NEt_3 (2.77 mL, 20.0 mmol, 2.0 eq.) and methylamine (2 M solution in THF, 6.0 mL, 12 mmol, 1.2 eq.) in CH_2Cl_2 (10 mL). Purification by flash column chromatography (EtOAc/Pentane, *v/v* = 1:4) gave the title compound as a colourless oil (1.71 g, 10.0 mmol, >99%).

^{1}H NMR (300 MHz, $CDCl_3$, 300 K): δ_H (ppm) = 7.88 – 7.83 (m, 2H), 7.60 – 7.46 (m, 3H), 5.15 (q, *J* = 5.3 Hz, 1H), 2.60 (d, *J* = 5.3 Hz, 3H).

^{13}C NMR (75 MHz, $CDCl_3$, 300 K): δ_C (ppm) = 138.6 (C_q), 132.8 (C_q), 129.2 (*C*H), 127.2 (*C*H), 29.3 (CH_3).

HRMS (ESI) *m/z* = 194.0246 calcd. for $[C_7H_9NO_2SNa]^+$ $[M+Na]^+$, found: 194.0244.

The analytical data are consistent with those reported in literature.[6]

***N*-(2,2,2-Trifluoroethyl)benzenesulfonamide (1c):**

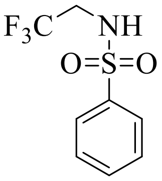

The title compound was prepared following the general procedure **GP1** using benzenesulfonyl chloride (1.28 mL, 10.0 mmol, 1.0 eq.), NEt_3 (5.50 mL, 40.0 mmol, 4.0 eq.) and 2,2,2-trifluoroethylammonium chloride (1.63 g, 12.0 mmol, 1.2 eq.) in CH_2Cl_2 (20 mL). Purification by flash column chromatography (EtOAc/Pentane, *v/v* = 1:4) gave the title compound as a colourless solid (560 mg, 2.34 mmol, 23%).

MP: 136-137 °C.

FT-IR (neat): ν (cm^{-1}) = 3270, 1448, 1409, 1332, 1319, 1275, 1148, 1108, 1089, 964, 849, 756, 722, 688, 667, 594.

^{1}H NMR (600 MHz, $CDCl_3$, 300 K): δ_H (ppm) = 7.75 – 7.73 (m, 2H), 7.59 – 7.55 (m, 1H), 7.53 – 7.49 (m, 2H), 3.60 (q, *J* = 9.5 Hz, 2H).

^{13}C{^{1}H,^{19}F} NMR (151 MHz, $CDCl_3$, 300 K): δ_C (ppm) = 141.1 (C_q), 133.2 (*C*H), 129.8 (*C*H), 126.7 (*C*H), 124.8 (C_q), 43.8 (CH_2).

^{19}F{^{1}H,^{13}C} NMR (564 MHz, $CDCl_3$, 300 K): δ_C (ppm) = −71.1.

HRMS (ESI) *m/z* = 262.0120 calcd. for $[C_8H_8NO_2SF_3Na]^+$ $[M+Na]^+$, found: 262.0118.

***N*-Benzylbenzenesulfonamide (1d):**

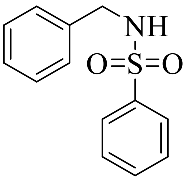

The title compound was prepared following the general procedure **GP1** using benzenesulfonyl chloride (640 µL, 5.00 mmol, 1.0 eq.), NEt_3 (2.77 mL, 10.0 mmol, 2.0 eq.) and benzylamine (981 µL, 6.00 mmol, 1.2 eq.) in CH_2Cl_2 (10 mL). Purification by flash column chromatography (EtOAc/Pentane, *v/v* = 1:4) gave the title compound as a colourless solid (1.17 g, 4.75 mmol, 95%).

^{1}H NMR (300 MHz, $CDCl_3$, 300 K): δ_H (ppm) = 7.91 – 7.88 (m, 2H), 7.62 – 7.48 (m, 3H), 7.29 – 7.18 (m, 5H), 5.16 (t, *J* = 6.2 Hz, 1H), 4.15 (d, *J* = 6.2 Hz, 2H).

^{13}C NMR (75 MHz, $CDCl_3$, 300 K): δ_C (ppm) = 139.9 (C_q), 136.3 (C_q), 132.7 (C_q), 129.2 (*C*H), 128.7 (*C*H), 127.9 (*C*H), 127.1 (*C*H), 47.2 (CH_2).

HRMS (ESI) *m/z* = 270.0559 calcd. for $[C_{13}H_{13}NO_2SNa]^+$ $[M+Na]^+$, found: 270.0556.

The analytical data are consistent with those reported in literature.[7]

***N*-Cyclohexylbenzenesulfonamide (1e):**

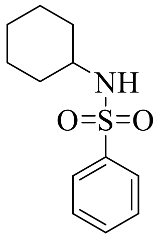

The title compound was prepared following the general procedure **GP1** using benzenesulfonyl chloride (1.28 mL, 10.0 mmol, 1.0 eq.), NEt_3 (2.77 mL, 20.0 mmol, 2.0 eq.) and cyclohexylamine (1.38 mL, 12.0 mmol, 1.2 eq.) in CH_2Cl_2 (10 mL). Purification by flash column chromatography (EtOAc/Pentane, *v/v* = 1:4) gave the title compound as a colourless solid (2.39 g, 10.0 mmol, >99%.).

^{1}H NMR (300 MHz, $CDCl_3$, 300 K): δ_H (ppm) = 7.92 – 7.89 (m, 2H), 7.60 – 7.47 (m, 3H), 4.89 (d, *J* = 7.6 Hz, 1H), 3.20 – 3.08 (m, 1H), 1.76 – 1.48 (m, 5H), 1.29 – 1.07 (m, 5H).

^{13}C NMR (75 MHz, $CDCl_3$, 300 K): δ_C (ppm) = 141.5 (C_q), 132.4 (*C*H), 129.0 (*C*H), 126.9 (*C*H), 52.7 (*C*H), 33.9 (CH_2), 25.1 (CH_2), 24.6 (CH_2).

HRMS (ESI) *m/z* = 262.0872 calcd. for $[C_{12}H_{17}NO_2SNa]^+$ $[M+Na]^+$, found: 262.0872.

The analytical data are consistent with those reported in literature.[6]

***N*-(2,2-Dimethyl-1,3-dioxan-5-yl)benzenesulfonamide (1f):**

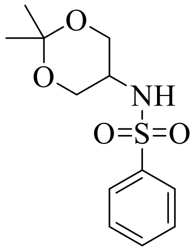

2,2-Dimethyl-1,3-dioxan-5-amine was prepared following a procedure by *Jie et al.*[8] The title compound was prepared following the general procedure **GP1** using benzenesulfonyl chloride (2.58 mL, 20.2 mmol, 1.0 eq.), NEt_3 (5.59 mL, 40.3 mmol, 2.0 eq.) and 2,2-dimethyl-1,3-dioxan-5-amine (3.18 g, 24.2 mmol, 1.2 eq.) in CH_2Cl_2 (15 mL). Purification by flash column chromatography (EtOAc/Pentane, gradient from *v/v* = 1:9 to 1:4) gave the title compound as a colourless oil (2.08 g, 7.68 mmol, 38% over two steps).

FT-IR (neat): ν (cm^{-1}) = 3282, 3068, 2994, 2879, 1481, 1448, 1426, 1375, 1330, 1310, 1246, 1199, 1159, 1079, 1045, 1026, 956, 922, 891, 821, 756, 721, 690, 591, 573.

^{1}H NMR (300 MHz, $CDCl_3$, 300 K): δ_H (ppm) = 7.85 – 7.82 (m, 2H), 7.54 – 7.42 (m, 3H), 5.57 (d, *J* = 9.6 Hz, 1H), 3.89 – 3.84 (m, 2H), 3.47 – 3.42 (m, 2H), 3.23 (dq, *J* = 9.6, 3.1 Hz, 1H), 1.31 (d, *J* = 3.3 Hz, 1H).

^{13}C NMR (75 MHz, $CDCl_3$, 300 K): δ_C (ppm) = 141.0 (C_q), 132.8 (*C*H), 129.3 (*C*H), 126.9 (*C*H), 98.6 (C_q), 63.7 (CH_2), 47.4 (*C*H), 27.1 (CH_3), 20.0 (CH_3).

HRMS (ESI) *m/z* = 294.0771 calcd. for $[C_{12}H_{17}NO_4SNa]^+$ $[M+Na]^+$, found: 294.0769.

***N*-(*tert*-Butyl)benzenesulfonamide (1g):**

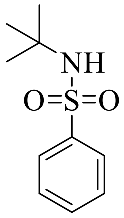

The title compound was prepared following the general procedure **GP1** using benzenesulfonyl chloride (2.56 mL, 20.0 mmol, 1.0 eq.), NEt_3 (5.54 mL, 40.0 mmol, 2.0 eq.) and *tert*-butyl amine (2.51 mL, 24.0 mmol, 1.2 eq.) in CH_2Cl_2 (100 mL). Purification by flash column chromatography (EtOAc/Pentane, *v/v* = 3:17) gave the title compound as a colourless solid (3.25 g, 15.3 mmol, 76%.).

^{1}H NMR (300 MHz, $CDCl_3$, 300 K): δ_H (ppm) = 7.95 – 7.90 (m, 2H), 7.57 – 7.45 (m, 3H), 5.19 (s, 1H), 1.21 (s, 9H).

^{13}C NMR (75 MHz, $CDCl_3$, 300 K): δ_C (ppm) = 143.5 (C_q), 132.1 (*C*H), 128.9 (*C*H), 126.9 (*C*H), 54.6 (C_q), 30.1 (CH_3).

HRMS (ESI) *m/z* = 236.0716 calcd. for $[C_{10}H_{15}NO_2SNa]^+$ $[M+Na]^+$, found: 236.0711.

The analytical data are consistent with those reported in literature.[9]

***N*-Phenylbenzenesulfonamide (1h):**

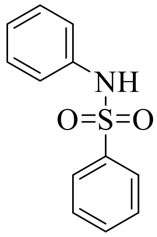

The title compound was prepared following the general procedure **GP2** using benzenesulfonyl chloride (1.28 mL, 10.0 mmol, 1.0 eq.), NEt_3 (2.10 mL, 15.0 mmol, 1.5 eq.) and aniline (904 µL, 10.0 mmol, 1.0 eq.) in CH_2Cl_2 (20 mL). Purification by flash column chromatography (CH_2Cl_2/Pentane, *v/v* = 1:4) gave the title compound as a colourless solid (2.12 g, 9.09 mmol, 91%).

^{1}H NMR (300 MHz, $CDCl_3$, 300 K): δ_H (ppm) = 7.84 – 7.79 (m, 2H), 7.55 – 7.39 (m, 3H), 7.25 – 7.19 (m, 2H), 7.12 – 7.07 (m, 4H).

^{13}C NMR (75 MHz, $CDCl_3$, 300 K): δ_C (ppm) = 138.9 (C_q), 136.4 (C_q), 133.1 (*C*H), 129.4 (*C*H), 129.1 (*C*H), 127.3 (*C*H), 125.4 (*C*H), 121.6 (*C*H).

HRMS (ESI) *m/z* = 256.0403 calcd. for $[C_{12}H_{11}NO_2SNa]^+$ $[M+Na]^+$, found: 256.0401.

The analytical data are consistent with those reported in literature.[7]

(*S*)-4-Methyl-N-(1-phenylethyl)benzenesulfonamide (1i):

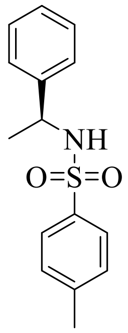

The title compound was prepared following the general procedure **GP1** using tosyl chloride (1.91 g, 10.0 mmol, 1.0 eq.), NEt_3 (2.77 mL, 20.0 mmol, 2.0 eq.) and (*S*)-phenethylamine (1.53 mL, 12.0 mmol, 1.2 eq.) in CH_2Cl_2 (20 mL). Purification by flash column chromatography (EtOAc/Pentane, *v/v* = 1:9) gave the title compound as a colourless solid (2.70 g, 9.81 mmol, 98%).

^{1}H NMR (300 MHz, $CDCl_3$, 300 K): δ_H (ppm) = 7.65 – 7.61 (m, 2H), 7.23 – 7.09 (m, 7H), 5.21 (d, *J* = 7.1 Hz, 1H), 4.47 (p, *J* = 6.9 Hz, 1H), 2.39 (s, 3H), 1.42 (d, *J* = 7.0 Hz, 3H).

^{13}C NMR (75 MHz, $CDCl_3$, 300 K): δ_C (ppm) = 143.1 (C_q), 142.1 (C_q), 137.7 (C_q), 129.4 (*C*H), 128.5 (*C*H), 127.4 (*C*H), 127.1 (*C*H), 126.1 (*C*H), 53.7 (*C*H), 23.6 (CH_3), 21.5 (CH_3).

HRMS (ESI) *m/z* = 298.0872 calcd. for $[C_{15}H_{17}NO_2SNa]^+$ $[M+Na]^+$, found: 298.0876.

The analytical data are consistent with those reported in literature.[10]

Methyl tosyl-*L*-valinate (1j):

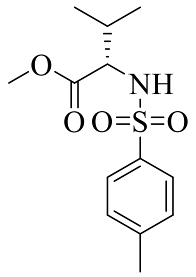

The title compound was prepared following the general procedure **GP1** using tosyl chloride (953 mg, 5.00 mmol, 1.0 eq.), NEt_3 (5.54 mL, 20.0 mmol, 4.0 eq.) and *L*-valinemethylester hydrochloride (1.00 g, 6.00 mmol, 1.2 eq.) in CH_2Cl_2 (10 mL). Purification by flash column chromatography (EtOAc/Pentane, *v/v* = 1:9) gave the title compound as a colourless solid (829 mg, 2.90 mmol, 58%).

^{1}H NMR (300 MHz, $CDCl_3$, 300 K): δ_H (ppm) = 7.74 – 7.70 (m, 2H), 7.31 – 7.27 (m, 2H), 5.15 (d, *J* = 10.1 Hz, 1H), 3.74 (dd, *J* = 10.1, 5.2 Hz, 1H), 3.45 (s, 3H), 2.42 (s, 3H), 2.03 (dhept, *J* = 6.8, 5.2 Hz, 1H), 0.96 (d, *J* = 6.8 Hz, 3H), 0.88 (d, *J* = 6.8 Hz, 3H).

^{13}C NMR (75 MHz, $CDCl_3$, 300 K): δ_C (ppm) = 171.8 (C_q), 143.6 (C_q), 136.6 (C_q), 129.6 (*C*H), 127.3 (*C*H), 61.0 (*C*H), 52.2 (CH_3), 31.6 (*C*H), 21.5 (CH_3), 18.9 (CH_3), 17.5 (CH_3).

HRMS (ESI) *m/z* = 308.0927 calcd. for [C13H19NO4SNa]$^+$ $[M+Na]^+$, found: 308.0935.

The analytical data are consistent with those reported in literature.[11]

***N*-((1S,2S)-2-hydroxy-1,2-diphenylethyl)-4-methylbenzenesulfonamide (1k):**

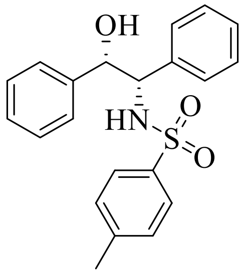

(1S,2S)-Pseudonorephenamine was prepared from (1S,2R)-norephenamine following a literature procedure by *Myers et al.*[12] The title compound was prepared following the general procedure **GP1** using tosyl chloride (953 mg, 5.00 mmol, 1.0 eq.), NEt_3 (5.54 mL, 20.0 mmol, 4.0 eq.) and (1S,2S)-pseudonorephenamine (1.00 g, 6.00 mmol, 1.2 eq.) in CH_2Cl_2 (10 mL). Purification by flash column chromatography (EtOAc/Pentane, *v/v* = 1:9) gave the title compound as a colourless solid (829 mg, 2.90 mmol, 58%).

^{1}H NMR (300 MHz, $CDCl_3$, 300 K): δ_H (ppm) = 7.39 – 7.36 (m, 2H), 7.20 – 7.00 (m, 12H), 5.51 (d, *J* = 6.2 Hz, 1H), 4.80 (d, *J* = 5.9 Hz, 1H), 4.42 (t, *J* = 6.0 Hz, 1H), 2.44 (s, 1H), 2.34 (s, 3H).

^{13}C NMR (75 MHz, $CDCl_3$, 300 K): δ_C (ppm) = 142.9 (C_q), 139.4 (C_q), 137.7 (C_q), 136.8 (C_q), 129.3 (*C*H), 128.3 (2 x *C*H), 128.0 (*C*H), 127.7 (*C*H), 127.4 (*C*H), 127.0 (*C*H), 126.3 (*C*H), 77.2 (*C*H), 64.0 (*C*H), 21.5 (CH_3).

HRMS (ESI) *m/z* = 390.1134 calcd. for $[C_{21}H_{21}NO_3SNa]^+$ $[M+Na]^+$, found: 390.1134.

The analytical data are consistent with those reported in literature.[13]

***N*-((1S,2S)-2-Methoxy-1,2-diphenylethyl)-4-methylbenzenesulfonamide (1l):**

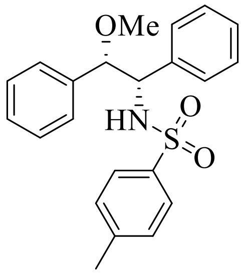

(1S,2S)-Pseudonorephenamine was prepared from (1S,2R)-norephenamine following a literature procedure by *Myers et al.*[12], which was subsequently O-methylated to give (1S,2S)-2-methoxy-1,2-diphenylethan-1-amine following a procedure by *Hashimoto et al.*[14] The title compound was prepared following the general procedure **GP1** using tosyl chloride (128 mg, 0.669 mmol, 1.0 eq.), NEt_3 (185 µL, 1.34 mmol, 2.0 eq.) and (1S,2S)-2-methoxy-1,2-diphenylethan-1-amine (167 mg, 0.736 mmol, 1.1 eq.) in CH_2Cl_2 (10 mL). Purification by flash column chromatography (EtOAc/Pentane, *v/v* = 1:9) gave the title compound as a colourless solid (194 mg, 0.509 mmol, 69%).

MP: 123-124 °C

FT-IR (neat): ν (cm^{-1}) = 3281, 3068, 3033, 2826, 1600, 1495, 1454, 1402, 1325, 1207, 1156, 1091, 1067, 1028, 976, 909, 813, 768, 730, 698, 665, 648, 625, 603.

^{1}H NMR (300 MHz, $CDCl_3$, 300 K): δ_H (ppm) = 7.41 – 7.38 (m, 2H), 7.21 – 7.01 (m, 8H), 6.96 – 6.91 (m, 4H), 5.70 (d, *J* = 3.6 Hz, 1H), 4.32 (dd, *J* = 7.2, 3.6 Hz, 1H), 4.18 (d, *J* = 7.2 Hz, 1H), 3.18 (s, 3H), 2.35 (s, 3H).

^{13}C NMR (75 MHz, $CDCl_3$, 300 K): δ_C (ppm) = 142.8 (C_q), 137.1 (2 x C_q), 137.0 (C_q), 129.1 (*C*H), 128.2 (2 x *C*H), 128.1 (2 x *C*H), 127.8 (*C*H), 127.5 (C_q), 127.4 (*C*H), 127.2 (*C*H), 86.4 (*C*H), 63.8 (*C*H), 57.0 (CH_3), 21.5 (CH_3).

HRMS (ESI) *m/z* = 404.1291 calcd. for $[C_{22}H_{23}NO_3SNa]^+$ $[M+Na]^+$, found: 404.1298.

***N*-((1S,2S)-2-(Benzyloxy)-1,2-diphenylethyl)-4-methylbenzenesulfonamide (1m):**

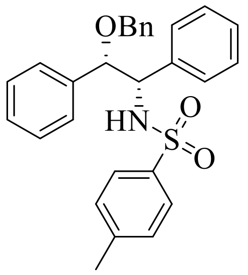

(1S,2S)-Pseudonorephenamine was prepared from (1S,2R)-norephenamine following a literature procedure by *Myers et al.*[12], which was subsequently O-benzylated to give (1S,2S)-2-(benzyloxy)-1,2-diphenylethan-1-amine following a procedure by *Ishii et al.*[15] The title compound was prepared following the general procedure **GP1** using tosyl chloride (294 mg, 1.54 mmol, 1.0 eq.), NEt_3 (428 µL, 3.09 mmol, 2.0 eq.) and (1S,2S)-2-(benzyloxy)-1,2-diphenylethan-1-amine (515 mg, 1.70 mmol, 1.1 eq.) in CH_2Cl_2 (15 mL). Purification by flash column chromatography (Pentane/EtOAc, *v/v* = 1:9) gave the title compound as a colourless solid (675 mg, 1.48 mmol, 87%).

MP: 89-91 °C.

FT-IR (neat): ν (cm^{-1}) = 3294, 3063, 3031, 2869, 1658, 1600, 1495, 1454, 1407, 1326, 1202, 1161, 1089, 1072, 1027, 1003, 908, 813, 770, 734, 699, 667, 589, 562.

^{1}H NMR (300 MHz, $CDCl_3$, 300 K): δ_H (ppm) = 7.39 – 6.98 (m, 19H), 5.68 – 5.67 (m, 1H), 4.46 – 4.43 (m, 3H), 4.18 (d, *J* = 11.6 Hz, 1H), 2.35 (s, 3H).

^{13}C NMR (151 MHz, $CDCl_3$, 300 K): δ_C (ppm) = 142.7 (C_q), 137.7 (C_q) , 137.3 (C_q), 137.1 (C_q), 129.1 (*C*H), 128.4 (*C*H), 128.3 (*C*H), 128.2 (*C*H), 127.9 (*C*H), 127.8 (*C*H), 127.8 (*C*H), 127.5 (*C*H), 127.4 (*C*H), 127.1 (*C*H), 83.9 (*C*H), 70.9 (CH_2), 63.6 (*C*H), 21.4 (CH_3).[1]

HRMS (ESI) *m/z* = 480.1604 calcd. for $[C_{28}H_{27}NO_3SNa]^+$ $[M+Na]^+$, found: 480.1616.

[1] some aromatic signals are superimposed.

***N*-((1S,2S)-2-(Methoxymethoxy)-1,2-diphenylethyl)-4-methylbenzenesulfonamide (1n):**

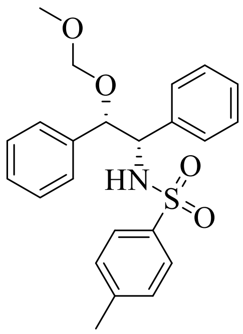

(1S,2S)-Pseudonorephenamine was prepared from (1S,2R)-norephenamine following a literature procedure by *Myers et al.*[12], which was subsequently O-MOM-protected. To this end, to a solution of (1S,2S)-pseudoephenamine (1.00 g, 4.69 mmol, 1.0 eq.) in anhydrous DMF (10 mL) was added sodium hydride (60% dispersion in mineral oil, 206 mg, 5.16 mmol, 1.1 eq.) and the suspension was stirred for one hour at room temperature. Methoxymethyl chloride (392 µL, 5.16 mmol, 1.1 eq.) was added and stirring was continued overnight. The reaction mixture was quenched by addition of NH_4Cl-solution (sat. aq., 20 mL) and EtOAc (20 mL). The mixture was stirred for one more hour at room temperature to make sure that all residual methoxymethyl chloride got quenched. The phases were separated and the aqueous phase was extracted with EtOAc (3 x 30 mL). The combined organic phases were dried over $NaSO_4$ and concentrated *in vacuo*. Purification by *reversed-phase* MPLC (MeOH/H_2O, gradient from 40% to 90%) gave 2-(methoxymethoxy)-1,2-diphenylethan-1-amine as a colourless solid (363 mg, 1.41 mmol, 30%). The title compound was prepared following the general procedure **GP1** using tosyl chloride (246 mg, 1.29 mmol, 1.0 eq.), NEt_3 (358 µL, 2.58 mmol, 2.0 eq.) and (1S,2S)-2-(methoxymethoxy)-1,2-diphenylethan-1-amine (349 mg, 1.36 mmol, 1.05 eq.) in CH_2Cl_2 (10 mL). Purification by flash column chromatography (EtOAc/Pentane, *v/v* = 1:9) gave the title compound as a colourless solid (392 mg, 0.952 mmol, 74%).

MP: 101-103 °C.

FT-IR (neat): ν (cm^{-1}) = 3294, 3034, 2896, 1600, 1496, 1454, 1328, 1157, 1093, 1076, 1026, 965, 916, 813, 773, 700, 665, 607, 562.

^{1}H NMR (300 MHz, $CDCl_3$, 300 K): δ_H (ppm) = 7.36 – 7.34 (m, 2H), 7.20 – 7.00 (m, 12H), 5.61 (d, *J* = 5.4 Hz, 1H), 4.71 (d, *J* = 5.8 Hz, 1H), 4.51 – 4.42 (m, 3H), 3.10 (s, 3H), 2.34 (s, 3H).

^{13}C NMR (75 MHz, $CDCl_3$, 300 K): δ_C (ppm) = 142.7 (C_q), 137.8 (C_q), 137.3 (C_q), 137.2 (C_q), 129.1 (*C*H), 128.3 (*C*H), 128.1 (*C*H), 127.9 (*C*H), 127.7 (*C*H), 127.4 (*C*H), 127.3 (*C*H), 127.0 (*C*H), 94.4 (CH_2), 80.8 (*C*H), 63.3 (*C*H), 55.9 (CH_3), 21.4 (CH_3).

HRMS (ESI) *m/z* = 434.1397 calcd. for $[C_{23}H_{25}NO_4SNa]^+$ $[M+Na]^+$, found: 434.1404.

_N_-Isopropyl-4-methylbenzenesulfonamide (1o):

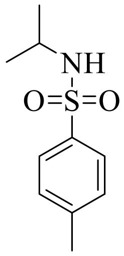

The title compound was prepared following the general procedure **GP1** using tosyl chloride (1.91 g, 10.0 mmol, 1.0 eq.), NEt_3 (2.77 mL, 20.0 mmol, 2.0 eq.) and iso-propylamine (1.03 mL, 12.0 mmol, 1.2 eq.) in CH_2Cl_2 (10 mL). Purification by flash column chromatography (EtOAc/Pentane, *v/v* = 1:4) gave the title compound as a colourless oil (2.05 g, 9.62 mmol, 96%).

^{1}H NMR (300 MHz, $CDCl_3$, 300 K): δ_H (ppm) = 7.80 (d, *J* = 8.3 Hz, 1H), 7.31 (d, *J* = 8.3 Hz, 1H), 5.06 (d, *J* = 7.4 Hz, 1H), 3.51 – 3.35 (m, 1H), 2.43 (s, 3H), 1.08 (d, *J* = 6.6 Hz, 6H).

^{13}C NMR (75 MHz, $CDCl_3$, 300 K): δ_C (ppm) = 143.1 (C_q), 138.2 (C_q), 129.6 (*C*H), 127.0 (*C*H), 46.0 (*C*H), 23.6 (CH_3), 21.5 (CH_3).

HRMS (ESI) *m/z* = 236.0716 calcd. for $[C_{10}H_{15}NO_2SNa]^+$ $[M+Na]^+$, found: 236.0717.

The analytical data are consistent with those reported in literature.[16]

4-Fluoro-_N_-isopropylbenzenesulfonamide (1p):

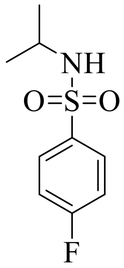

The title compound was prepared following the general procedure **GP1** using 4-fluorobenzenesulfonyl chloride (584 mg, 3.00 mmol, 1.0 eq.), NEt_3 (832 µL, 6.00 mmol, 2.0 eq.) and isopropylamine (308 µL, 3.60 mmol, 1.2 eq.) in CH_2Cl_2 (10 mL). Purification by flash column chromatography (EtOAc/Pentane, *v/v* = 1:4) gave the title compound as a colourless oil (626 mg, 2.88 mmol, 96%).

FT-IR (neat) υ (cm^{-1}) = 3287, 2977, 2938, 1593, 1495, 1467, 1426, 1389, 1371, 1325, 1291, 1267, 1237, 1152, 1090, 1001, 893, 837, 818, 735.

^{1}H NMR (300 MHz, $CDCl_3$, 300 K): δ_H (ppm) = 7.94 – 7.85 (m, 2H), 7.22 – 7.14 (m, 2H), 4.95 (d, *J* = 7.6 Hz, 1H), 3.52 – 3.36 (m, 1H), 1.07 (d, *J* = 6.6 Hz, 6H).

^{13}C NMR (75 MHz, $CDCl_3$, 300 K): δ_C (ppm) = 164.9 (d, *J* = 254.3 Hz, C_q), 137.2 (d, *J* = 3.2 Hz, C_q), 129.7 (d, *J* = 9.3 Hz, *C*H), 116.3 (d, *J* = 22.4 Hz, *C*H), 46.2 (*C*H), 23.7 (CH_3).

^{19}F NMR (283 MHz, $CDCl_3$, 300 K): δ_F (ppm) = −105.7.

HRMS (ESI) *m/z* = 240.0465 calcd. for $[C_9H_{12}NO_2SFNa]^+$ $[M+Na]^+$, found: 240.0462.

The analytical data are consistent with those reported in literature.[17]

4-Chloro-*N*-isopropylbenzenesulfonamide (1q):

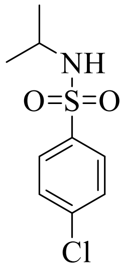

The title compound was prepared following the general procedure **GP1** using 4-chlorobenzenesulfonyl chloride (633 mg, 3.00 mmol, 1.0 eq.), NEt_3 (832 µL, 6.00 mmol, 2.0 eq.) and isopropylamine (308 µL, 3.60 mmol, 1.2 eq.) in CH_2Cl_2 (10 mL). Purification by flash column chromatography (EtOAc/Pentane, *v/v* = 1:4) gave the title compound as a colourless solid (649 mg, 2.78 mmol, 93%).

^{1}H NMR (300 MHz, $CDCl_3$, 300 K): δ_H (ppm) = 7.86 – 7.81 (m, 2H), 7.50 – 7.46 (m, 2H), 4.90 (d, *J* = 7.6 Hz, 1H), 3.53 – 3.38 (m, 1H), 1.08 (d, *J* = 6.5 Hz, 6H).

^{13}C NMR (75 MHz, $CDCl_3$, 300 K): δ_C (ppm) = 139.7 (C_q), 138.9 (C_q), 129.4 (*C*H), 128.5 (*C*H), 46.2 (*C*H), 23.7 (CH_3).

HRMS (ESI) *m/z* = 256.0170 calcd. for $[C_9H_{12}NO_2SClNa]^+$ $[M+Na]^+$, found: 256.0168.

The analytical data are consistent with those reported in literature.[18]

***N*-Isopropyl-4-methoxybenzenesulfonamide (1r):**

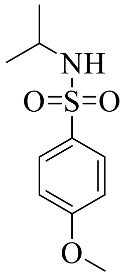

The title compound was prepared following the general procedure **GP1** using 4-methoxybenzenesulfonyl chloride (459 mg, 3.00 mmol, 1.0 eq.), NEt_3 (832 µL, 6.00 mmol, 2.0 eq.) and isopropylamine (308 µL, 3.60 mmol, 1.2 eq.) in CH_2Cl_2 (10 mL). Purification by flash column chromatography (EtOAc/Pentane, *v/v* = 1:9) gave the title compound as a yellowish oil (515 mg, 2.25 mmol, 75%).

^{1}H NMR (400 MHz, $CDCl_3$, 300 K): δ_H (ppm) = 7.83 – 7.81 (m, 2H), 6.98 – 6.96 (m, 2H), 4.73 (d, *J* = 7.4 Hz, 1H), 3.86 (s, 3H), 3.47 – 3.35 (m, 1H), 1.06 (d, *J* = 6.6 Hz, 6H).

^{13}C NMR (101 MHz, $CDCl_3$, 300 K): δ_C (ppm) = 162.7 (C_q), 132.7 (C_q), 129.1 (*C*H), 114.2 (*C*H), 55.6 (CH_3), 46.0 (*C*H), 23.7 (CH_3).

HRMS (ESI) *m/z* = 252.0665 calcd. for $[C_{10}H_{15}NO_3SNa]^+$ $[M+Na]^+$, found: 252.0548.

The analytical data are consistent with those reported in literature.[5]

***N*-Isopropyl-4-(trifluoromethyl)benzenesulfonamide (1s):**

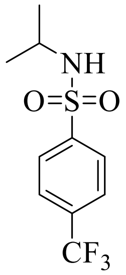

The title compound was prepared following the general procedure **GP1** using 4-trifluoromethylbenzenesulfonyl chloride (466 mg, 1.90 mmol, 1.0 eq.), NEt_3 (528 µL, 3.80 mmol, 2.0 eq.) and isopropylamine (196 µL, 2.28 mmol, 1.2 eq.) in CH_2Cl_2 (10 mL). Purification by flash column chromatography (EtOAc/Pentane, *v/v* = 1:4) gave the title compound as a colourless solid (451 mg, 1.69 mmol, 75%).

MP: 68-69 °C.

FT-IR (neat) υ (cm^{-1}) = 3525, 3343, 3204, 2968, 2925, 2859, 1999, 1641, 1600, 1490, 1440, 1385, 1365, 1324, 1250, 1197, 1170, 1130, 1081, 1036, 952, 913, 846, 812, 760, 724, 698, 644.

^{1}H NMR (600 MHz, $CDCl_3$, 300 K): δ_H (ppm) = 8.04 – 8.02 (m, 2H), 7.80 – 7.78 (m, 2H), 4.72 (d, *J* = 7.7 Hz, 1H), 3.52 (dhept, *J* = 7.7, 6.5 Hz, 1H), 1.11 (d, *J* = 6.5 Hz, 6H).

^{13}C NMR (101 MHz, $CDCl_3$, 300 K): δ_C (ppm) = 144.9 (C_q), 134.2 (C_q), 127.5 (*C*H), 126.2 (*C*H), 123.3 (C_q), 46.4 (*C*H), 23.8 (CH_3).

^{19}F{^{1}H, ^{13}C} NMR (377 MHz, $CDCl_3$, 300 K): δ_F (ppm) = −63.1.

HRMS (ESI) *m/z* = 290.0433 calcd. for $[C_{10}H_{12}NO_2SF_3Na]^+$ $[M+Na]^+$, found: 290.0430.

***N*-Isopropyl-4-nitrobenzenesulfonamide (1t):**

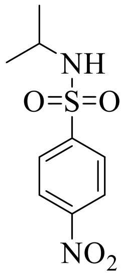

The title compound was prepared following the general procedure **GP1** using 4-nitrobenzenesulfonyl chloride (665 mg, 3.00 mmol, 1.0 eq.), NEt_3 (832 µL, 6.00 mmol, 2.0 eq.) and Isopropylamine (308 µL, 3.60 mmol, 1.2 eq.) in CH_2Cl_2 (10 mL). Purification by flash column chromatography (EtOAc/Pentane, *v/v* = 1:4) gave the title compound as a yellow solid (637 mg, 2.61 mmol, 87%).

^{1}H NMR (300 MHz, $CDCl_3$, 300 K): δ_H (ppm) = 8.39 – 8.36 (m, 2H), 8.10 – 8.08 (m, 2H), 4.88 (d, *J* = 7.5 Hz, 1H), 3.63 – 3.47 (m, 1H), 1.12 (d, *J* = 6.5 Hz, 6H).

^{13}C NMR (75 MHz, $CDCl_3$, 300 K): δ_C (ppm) = 149.9 (C_q), 147.2 (C_q), 128.2 (*C*H), 124.4 (*C*H), 46.6 (*C*H), 23.7 (CH_3).

HRMS (ESI) *m/z* = 243.0445 calcd. for $[C_9H_{11}N_2O_4S]^-$ $[M-H]^-$, found: 243.0441.

The analytical data are consistent with those reported in literature.[5]

4-Cyano-*N*-isopropylbenzenesulfonamide (1u):

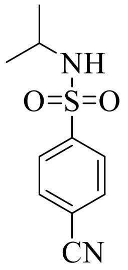

The title compound was prepared following the general procedure **GP1** using 4-cyanobenzenesulfonyl chloride (140 mg, 0.696 mmol, 1.0 eq.), NEt_3 (193 µL, 1.39 mmol, 2.0 eq.) and isopropylamine (72 µL, 0.84 mmol, 1.2 eq.) in CH_2Cl_2 (10 mL). Purification by filtration over a short plug of silica (eluent: EtOAc) gave the title compound as a colourless solid (115 mg, 0.513 mmol, 74%).

MP: 81-82 °C.

FT-IR (neat) υ (cm^{-1}) = 3263, 2976, 2936, 2234, 1489, 1455, 1425, 1390, 1371, 1332, 1304, 1283, 1150, 1141, 1090, 1003, 895, 837.

^{1}H NMR (300 MHz, $CDCl_3$, 300 K): δ_H (ppm) = 8.03 – 7.99 (m, 2H), 7.85 – 7.81 (m, 2H), 4.74 (d, J = 7.7 Hz, 1H), 3.53 (dhept, J = 7.7, 6.5 Hz, 1H), 1.11 (d, J = 6.5 Hz, 6H).

^{13}C NMR (75 MHz, $CDCl_3$, 300 K): δ_C (ppm) = 145.6 (C_q), 133.0 (CH), 127.6 (CH), 117.4 (C_q), 116.2 (C_q), 46.6 (CH), 23.8 (CH$_3$).

HRMS (ESI) m/z = 247.0512 calcd. for $[C_{10}H_{12}N_2O_3SNa]^+$ $[M+Na]^+$, found: 247.0511.

***N*-Isopropyl-4-(*N*-isopropylsulfamoyl)benzamide (1v):**

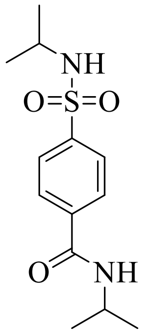

The title compound was prepared following the general procedure **GP1** using 4-(chlorosulfonyl)benzoic acid (662 mg, 3.00 mmol, 0.83 eq.), NEt_3 (1.66 mL, 12.0 mmol, 3.3 eq.) and isopropylamine (308 µL, 3.60 mmol, 1.0 eq.) in CH_2Cl_2 (10 mL). Purification by flash column chromatography (EtOAc/Pentane, v/v = 3:7) gave the title compound as a colourless solid (148 mg, 0.610 mmol, 40%).

MP: 158-160°C

FT-IR (neat) υ (cm^{-1}) = 3849, 3275, 2976, 1727, 1709, 1688, 1641, 1568, 1537, 1489, 1467, 1434, 1389, 1369, 1327, 1296, 1161, 1135, 1092, 1006, 869, 880, 857, 830, 765, 732, 699, 611, 572.

^{1}H NMR (300 MHz, DMSO-d^6, 300 K): δ_H (ppm) = 8.44 (d, J = 7.7 Hz, 1H), 8.01 – 7.97 (m, 2H), 7.88 – 7.84 (m, 2H), 7.71 (d, J = 7.3 Hz, 1H), 4.15 – 4.04 (m, 1H), 3.31 – 3.20 (m, 1H), 1.17 (d, J = 6.6 Hz, 6H), 0.93 (d, J = 6.5 Hz, 6H).

^{13}C NMR (75 MHz, DMSO-d^6, 300 K): δ_C (ppm) = 164.7 (C_q), 144.4 (C_q), 138.5 (C_q), 128.5 (CH), 126.7 (CH), 45.8 (CH), 41.7 (CH), 23.6 (CH$_3$), 22.7 (CH$_3$).

HRMS (ESI) *m/z* = 307.1087 calcd. for $[C_{13}H_{20}N_2O_3S]^+$ $[M+H]^+$, found: 307.1084.

***N*-Isopropyl-2-methylbenzenesulfonamide (1w):**

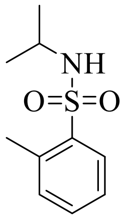

The title compound was prepared following the general procedure **GP1** using 2-methylbenzenesulfonyl chloride (433 µL, 3.00 mmol, 1.0 eq.), NEt_3 (832 µL, 6.00 mmol, 2.0 eq.) and isopropylamine (308 µL, 3.60 mmol, 1.2 eq.) in CH_2Cl_2 (10 mL). Purification by flash column chromatography (EtOAc/Pentane, *v/v* = 1:9) gave the title compound as a colourless solid (827 mg, 2.47 mmol, 82%).

^{1}H NMR (300 MHz, CDCl$_3$, 300 K): δ_H (ppm) = 8.02 – 7.99 (m, 1H), 7.47 – 7.42 (m, 1H), 7.33 – 7.27 (m, 2H), 4.82 (d, *J* = 7.6 Hz, 1H), 3.49 – 3.37 (m, 1H), 2.65 (s, 3H), 1.07 (d, *J* = 6.6 Hz, 6H).

^{13}C NMR (75 MHz, CDCl$_3$, 300 K): δ_C (ppm) = 138.8 (C_q), 136.9 (C_q), 132.6 (CH), 132.5 (CH), 129.4 (CH), 126.2 (CH), 46.0 (CH), 23.7 (CH$_3$), 20.3 (CH$_3$).

HRMS (ESI) *m/z* = 236.0716 calcd. for $[C_{10}H_{15}NO_2SNa]^+$ $[M+Na]^+$, found: 236.0711.

The analytical data are consistent with those reported in literature.[19]

***N*-Isopropyl-3-methylbenzenesulfonamide (1x):**

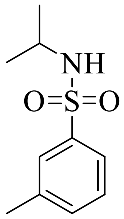

The title compound was prepared following the general procedure **GP1** using 3-methylbenzenesulfonyl chloride (432 µL, 3.00 mmol, 1.0 eq.), NEt_3 (832 µL, 6.00 mmol, 2.0 eq.) and isopropylamine (308 µL, 3.60 mmol, 1.2 eq.) in CH_2Cl_2 (10 mL). Purification by flash column chromatography (EtOAc/Pentane, *v/v* = 1:9) gave the title compound as a colourless oil (640 mg, 3.00 mmol, >99%).

FT-IR (neat) υ (cm^{-1}) = 3279, 2976, 2935, 1602, 1455, 1426, 1389, 1370, 1324, 1298, 1220, 1137, 1096, 1007, 994, 894, 861, 828, 786, 689, 599, 580.

^{1}H NMR (300 MHz, CDCl$_3$, 300 K): δ_H (ppm) = 7.73 – 7.68 (m, 2H), 7.41 – 7.36 (m, 2H), 4.97 (d, *J* = 7.5 Hz, 1H), 3.51 – 3.36 (m, 1H), 2.41 (s, 3H), 1.07 (d, *J* = 6.6 Hz, 6H).

^{13}C NMR (75 MHz, CDCl$_3$, 300 K): δ_C (ppm) = 140.9 (C_q), 139.2 (C_q), 133.2 (CH), 128.9 (CH), 127.3 (CH), 124.1 (CH), 46.1 (CH), 23.7 (CH$_3$), 21.4 (CH$_3$).

HRMS (ESI) *m/z* = 236.0716 calcd. for [xxx]$^+$ $[M+Na]^+$, found: 236.0712.

N-Isopropyl-3,5-bis(trifluoromethyl)benzenesulfonamide (1y):

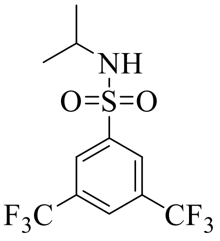

The title compound was prepared following the general procedure **GP1** using 3,5-bis(trifluoromethyl)benzenesulfonyl chloride (938 mg, 3.00 mmol, 1.0 eq.), NEt_3 (832 µL, 6.00 mmol, 2.0 eq.) and isopropylamine (308 µL, 3.60 mmol, 1.2 eq.) in CH_2Cl_2 (10 mL). Purification by flash column chromatography (EtOAc/Pentane, *v/v* = 1:4) gave the title compound as a colourless solid (779 mg, 2.323 mmol, 77%).

MP: 73-74 °C.

FT-IR (neat) υ (cm^{-1}) = 3280, 2979, 1611, 1360, 1279, 1144, 1109, 1011, 904, 845, 727, 699, 662, 650, 633, 591.

^{1}H NMR (600 MHz, $CDCl_3$, 300 K): δ_H (ppm) = 8.32 (s, 2H), 8.06 (s, 1H), 4.59 – 4.57 (m, 1H), 3.62 – 3.54 (m, 1H), 1.13 (d, *J* = 6.5 Hz, 6H).

^{13}C{^{1}H,^{19}F} NMR (101 MHz, $CDCl_3$, 300 K): δ_C (ppm) = 144.3 (C_q), 132.9 (C_q), 127.2 (*C*H), 126.0 (*C*H), 122.4 (*C*H), 46.8 (*C*H), 23.8 (*C*H_3).

^{19}F{^{1}H,^{13}C} NMR (564 MHz, $CDCl_3$, 300 K): δ_F (ppm) = −63.0.

HRMS (ESI) *m/z* = 358.0307 calcd. for $[C_{11}H_{11}NO_2SF_6Na]^+$ $[M+Na]^+$, found: 358.0307.

N-Isopropylnaphthalene-1-sulfonamide (1z):

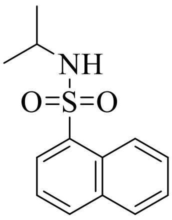

The title compound was prepared following the general procedure **GP1** using naphtalene-1-sulfonyl chloride (680 mg, 3.00 mmol, 1.0 eq.), NEt_3 (832 µL, 6.00 mmol, 2.0 eq.) and isopropylamine (308 µL, 3.60 mmol, 1.2 eq.) in CH_2Cl_2 (10 mL). Purification by flash column chromatography (EtOAc/Pentane, *v/v* = 1:9) gave the title compound as a colourless solid (690 mg, 2.80 mmol, 94%).

^{1}H NMR (300 MHz, $CDCl_3$, 300 K): δ_H (ppm) = 8.68 – 8.64 (m, 1H), 8.33 – 8.30 (m, 1H), 8.09 – 8.05 (m, 1H), 7.97 – 7.94 (m, 1H), 7.70 – 7.52 (m, 3H), 4.81 (d, *J* = 7.7 Hz, 1H), 3.52 – 3.37 (m, 1H), 0.98 (d, *J* = 6.5 Hz, 6H).

^{13}C NMR (75 MHz, $CDCl_3$, 300 K): δ_C (ppm) = 135.5 (C_q), 134.3 (C_q), 134.2 (*C*H), 129.5 (*C*H), 129.1 (*C*H), 128.3 (*C*H), 128.2 (C_q), 126.8 (*C*H), 124.4 (*C*H), 124.2 (*C*H), 46.3 (*C*H), 23.7 (*C*H_3).

HRMS (ESI) *m/z* = 272.0716 calcd. for $[C_{13}H_{15}NO_2SNa]^+$ $[M+Na]^+$, found: 272.0711.

The analytical data are consistent with those reported in literature.[20]

***N*-Isopropylnaphthalene-2-sulfonamide (1aa):**

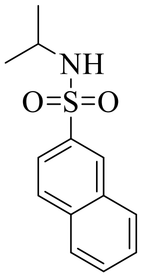

The title compound was prepared following the general procedure **GP1** using naphtalene-2-sulfonyl chloride (680 mg, 3.00 mmol, 1.0 eq.), NEt_3 (832 µL, 6.00 mmol, 2.0 eq.) and isopropylamine (308 µL, 3.60 mmol, 1.2 eq.) in CH_2Cl_2 (10 mL). Purification by flash column chromatography (EtOAc/Pentane, *v/v* = 1:9) gave the title compound as a colourless solid (748 mg, 3.00 mmol, >99%).

^{1}H NMR (300 MHz, $CDCl_3$, 300 K): δ_H (ppm) = 8.48 (s, 1H), 7.99 – 7.86 (m, 4H), 7.68 – 7.59 (m, 2H), 4.75 (d, *J* = 7.6 Hz, 1H), 3.58 – 3.46 (m, 1H), 1.09 (d, *J* = 6.5 Hz, 6H).

^{13}C NMR (75 MHz, $CDCl_3$, 300 K): δ_C (ppm) = 137.8 (C_q), 134.7 (C_q), 132.2 (C_q), 129.5 (*C*H), 129.3 (*C*H), 128.7 (*C*H), 128.3 (*C*H), 127.9 (*C*H), 127.5 (*C*H), 122.4 (*C*H), 46.2 (*C*H), 23.8 (CH_3).

HRMS (ESI) *m/z* = 272.0716 calcd. for $[C_{13}H_{15}NO_2SNa]^+$ $[M+Na]^+$, found: 272.0716.

The analytical data are consistent with those reported in the literature.[20]

***N*-Isopropylthiophene-2-sulfonamide (1ab):**

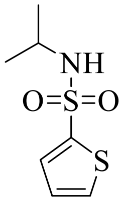

The title compound was prepared following the general procedure **GP1** using thiophene-2-sulfonyl chloride (548 mg, 3.00 mmol, 1.0 eq.), NEt_3 (832 µL, 6.00 mmol, 2.0 eq.) and isopropylamine (308 µL, 3.60 mmol, 1.2 eq.) in CH_2Cl_2 (10 mL). Purification by flash column chromatography (EtOAc/Pentane, *v/v* = 1:4) gave the title compound as a colourless solid (571 mg, 2.78 mmol, 93%).

MP: 44-45 °C.

FT-IR (neat) υ (cm^{-1}) = 3280, 2976, 2260, 1466, 1407, 1332, 1227, 1158, 1139, 1092, 1068, 1019, 998, 905, 856, 724, 685, 649, 598, 576.

^{1}H NMR (300 MHz, $CDCl_3$, 300 K): δ_H (ppm) = 7.64 – 7.57 (m, 2H), 7.09 – 7.06 (m, 1H), 4.89 (d, *J* = 7.5 Hz, 1H), 3.59 – 3.48 (m, 1H), 1.12 (d, *J* = 6.6 Hz, 6H).

^{13}C NMR (75 MHz, $CDCl_3$, 300 K): δ_C (ppm) = 142.2 (C_q), 132.0 (*C*H), 131.7 (*C*H), 127.4 (*C*H), 46.6 (*C*H), 23.6 (CH_3).

HRMS (ESI) *m/z* = 228.0123 calcd. for $[C_7H_{11}NO_2S_2Na]^+$ $[M+Na]^+$, found: 228.0120.

***N*-Isopropylbenzo[b]thiophene-2-sulfonamide (1ac):**

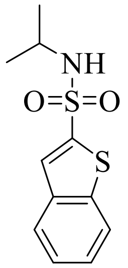

2,4,6-Trichlorophenyl sulfurochloridate was prepared following a procedure from the literature[21] and used without further purification. Following a modified procedure by *Buchwald et al.*[21], benzo[b]thiophene (671 mg, 5.00 mmol, 1.0 equiv.) was dissolved in anhydrous THF (10 mL) at −78 °C. *n*-BuLi (1.6 M-solution in hexanes, 3.13 mL, 5.00 mmol, 1.0 equiv.) was added at this temperature dropwise and stirring was continued for one hour. Zinc chloride (2.0 M-solution in 2-Me-THF, 2.50 mL, 5.00 mmol, 1.0 equiv.) was added, the mixture warmed to room temperature and stirred for one hour. After cooling to 0 °C, 2,4,6-trichlorophenyl sulfurochloridate (875 µL, 5.00 mmol, 1.0 equiv.) was added dropwise over 5 minutes. Stirring under slow warming of the ice/water cooling bath was continued for further two hours, then the cooling bath was refreshed and isopropyl amine (857 µL, 10.0 mmol, 2.0 equiv.) was added slowly. The formed suspension was allowed to warm to room temperature overnight until it was quenched by addition of brine (25 mL), water (25 mL) and EtOAc (50 mL). The phases were separated and the aqueous phase was extracted with EtOAc (2 x 50 mL). The combined organic phases were washed with brine (100 mL), dried over $MgSO_4$, filtered and concentrated *in vacuo*. Purification by FC (EtOAc/pentane, from *v/v* = 1:9 to *v/v* = 3:7) gave the title compound as an off-white solid (755 mg, 2.96 mmol, 59%).

MP: 98-99 °C.

FT-IR (neat) υ (cm^{-1}) = 3277, 3064, 2975, 2934, 2258, 1594, 1504, 1458, 1423, 1388, 1370, 1324, 1248, 1152, 1135, 1069, 1022, 1005, 945, 893, 868, 843, 746, 725, 689, 619, 582, 549.

^{1}H NMR (300 MHz, $CDCl_3$, 300 K): δ_H (ppm) = 7.90 – 7.85 (m, 3H), 7.52 – 7.42 (m, 2H), 4.84 (d, *J* = 7.5 Hz, 1H), 3.71 – 3.55 (m, 1H), 1.17 (d, *J* = 6.5 Hz, 6H).

^{13}C NMR (75 MHz, $CDCl_3$, 300 K): δ_C (ppm) = 142.2 (C_q), 141.7 (C_q), 137.7 (C_q), 129.1 (*C*H), 127.2 (*C*H), 125.6 (*C*H), 125.5 (*C*H), 122.8 (*C*H), 46.7 (*C*H), 23.8 (CH_3).

HRMS (ESI) *m/z* = 278.0280 calcd. for $[C_{11}H_{13}NO_2S_2Na]^+$ $[M+Na]^+$, found: 078.0276.

***N*-Isopropylbenzo[b]furan-2-sulfonamide (1ad):**

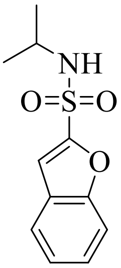

2,4,6-Trichlorophenyl sulfurochloridate was prepared following a procedure from the literature[21] and used without further purification. Following a modified procedure by *Buchwald et al.*[21], 2,3-benzofuran (537 µL, 5.00 mmol, 1.0 equiv.) was dissolved in anhydrous THF (10 mL) at −78 °C. *n*-BuLi (1.6 M-solution in hexanes, 3.13 mL, 5.00 mmol, 1.0 equiv.) was added at this temperature dropwise and stirring was continued for one hour. Zinc chloride (2.0 M-solution in 2-Me-THF, 2.50 mL, 5.00 mmol, 1.0 equiv.) was added, the mixture warmed to room temperature and stirred for one hour. After cooling to 0 °C, 2,4,6-trichlorophenyl sulfurochloridate (875 µL, 5.00 mmol, 1.0 equiv.) was added dropwise over 5 minutes. Stirring under slow warming of the ice/water cooling bath was continued for further two hours, then the cooling bath was refreshed and isopropyl amine (857 µL, 10.0 mmol, 2.0 equiv.) was added slowly. The formed suspension was allowed to warm to room temperature overnight until it was quenched by addition of brine (25 mL), water (25 mL) and EtOAc (50 mL). The phases were separated and the aqueous phase was extracted with EtOAc (2 x 50 mL). The combined organic phases were washed with brine (100 mL), dried over $MgSO_4$, filtered and concentrated *in vacuo*. Purification by FC (EtOAc/pentane, from *v/v* = 1:9 to *v/v* = 3:7) gave the title compound as an off-white solid (535 mg mg, 2.24 mmol, 45%).

MP: 104-105 °C.

FT-IR (neat) υ (cm^{-1}) = 3286, 2977, 2359, 2258, 1613, 1546, 1466, 1445, 1424, 1389, 1342, 1238, 1160, 1138, 1110, 1082, 1007, 932, 908, 878, 828, 793, 728, 644, 626.

^{1}H NMR (300 MHz, $CDCl_3$, 300 K): δ_H (ppm) = 7.70 – 7.67 (m, 1H), 7.57 – 7.54 (m, 1H), 7.49 – 7.27 (m, 3H), 5.00 (d, *J* = 7.8 Hz, 1H), 3.75 – 3.59 (m, 1H), 1.16 (d, *J* = 6.5 Hz, 6H).

^{13}C NMR (75 MHz, $CDCl_3$, 300 K): δ_C (ppm) = 155.6 (C_q), 150.8 (C_q), 127.6 (*C*H), 126.1 (C_q), 124.2 (*C*H), 122.9 (*C*H), 112.2 (*C*H), 111.9 (*C*H), 46.8 (*C*H), 23.8 (CH_3).

HRMS (ESI) *m/z* = 262.0508 calcd. for $[C_{11}H_{13}NO_3SNa]^+$ $[M+Na]^+$, found: 262.0506.

***N*-Isopropylpyridine-2-sulfonamide (1ae):**

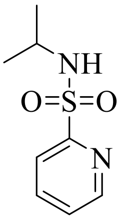

The title compound was prepared following a modified procedure from the literature.[22] Pyridine-2-thiol (561 mg, 5.00 mmol, 1.0 eq.) was dissolved in CH_2Cl_2 (25 mL) at 0 °C. HCl (37% aq., 25 mL) and NaOCl (11% aq., 9.3 mL) were added dropwise under vigorous stirring. After 15 minutes, the suspension was transferred

to a precooled separatory funnel and the phases were separated. The organic phase was cooled to 0 °C and NEt_3 (1.39 mL, 10.0 mmol, 2.0 eq.) and isopropylamine (514 µL, 6.0 mmol, 1.2 eq.) were added. The solution was allowed to warm to room temperature overnight, washed with HCl (1 N aq., 25 mL) and brine (25 mL), dried over $MgSO_4$ and concentrated *in vacuo*. Purification by RP-MPLC (MeOH/water, gradient from 5% to 90%) gave the title compound as a colourless solid (310 mg, 1.55 mmol, 31%).

MP: 107-108 °C.

FT-IR (neat) υ (cm^{-1}) = 3290, 3187, 2976, 2259, 1581, 1428, 1389, 1332, 1257, 1178, 1118, 1087, 1013, 992, 905, 776, 729, 648, 621, 599, 567.

^{1}H NMR (300 MHz, $CDCl_3$, 300 K): δ_H (ppm) = 8.72 (ddd, *J* = 4.7, 1.8, 0.9 Hz, 1H), 8.03 (dt, *J* = 7.9, 1.1 Hz, 1H), 7.91 (td, *J* = 7.7, 1.7 Hz, 1H), 7.50 (ddd, *J* = 7.6, 4.7, 1.1 Hz, 1H), 5.21 (d, *J* = 7.4 Hz, 1H), 3.62 – 3.48 (m, 1H), 1.09 (d, *J* = 6.6 Hz, 6H).

^{13}C NMR (75 MHz, $CDCl_3$, 300 K): δ_C (ppm) = 158.2 (C_q), 150.0 (*C*H), 138.1 (*C*H), 126.6 (*C*H), 122.1 (*C*H), 46.7 (*C*H), 23.7 (CH_3).

HRMS (ESI) *m/z* = 223.0512 calcd. for $[C_8H_{12}N_2O_2SNa]^+$ $[M+Na]^+$, found: 223.0508.

***N*-Isopropylbenzo[d]thiazole-2-sulfonamide (1af):**

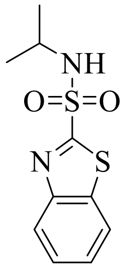

Benzo[d]thiazole-2-sulfonyl chloride was prepared from the corresponding thiol following a procedure from the literature[23] and used without further purification. The title compound was prepared following the general procedure **GP1** using benzo[d]thiazole-2-sulfonyl chloride (4.67 g[2], 20.0 mmol, 1.0 equiv.), NEt_3 (5.54 mL, 40.0 mmol 2.0 equiv.) and isopropylamine (2.06 mL, 24.0 mmol, 1.2 equiv.) in CH_2Cl_2 (100 mL). Purification by flash column chromatography (EtOAc/pentane, *v/v* = 3:17) gave the title compound as a colourless solid (869 mg, 3.39 mmol, 17%).

MP: 133-135 °C

FT-IR (neat) υ (cm^{-1}) = 3273, 2978, 2359, 1551, 1470, 1424, 1343, 1311, 1163, 1124, 1087, 1028, 998, 913, 851, 757, 690, 628, 605, 561.

^{1}H NMR (300 MHz, $CDCl_3$, 300 K): δ_H (ppm) = 8.20 – 8.14 (m, 1H), 7.99 – 7.94 (m, 1H), 7.64 – 7.46 (m, 2H), 5.40 (s, 1H), 3.84 – 3.80 (m, 1H), 1.21 (d, *J* = 6.6 Hz, 6H).

[2] The crude product obtained from the previous step was assumed to be pure for the calculation of stoichiometry.

^{13}C NMR (75 MHz, $CDCl_3$, 300 K): δ_C (ppm) = 152.4 (C_q), 136.4 (C_q), 127.6 (*C*H), 127.4 (*C*H), 125.0 (*C*H), 122.2 (*C*H), 47.5 (*C*H), 23.8 (CH_3).[3]

HRMS (ESI) *m/z* = 279.0232 calcd. for $[C_{10}H_{12}N_2O_2S_2Na]^+$ $[M+Na]^+$, found: 279.0229.

---

[3] One signal of a quaternary carbon atom was not resolved.

1.4.2 Synthesis of ketenes

Ethylphenylketene (2a):

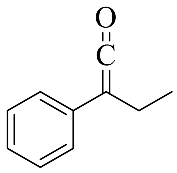

Ethylphenylketene **2a** was prepared following general procedure **GP2** using 2-phenylbutanoic acid (3.28 g, 20.0 mmol, 1.0 eq.), oxalyl chloride (3.43 mL, 40.0 mmol, 2.0 eq.) and NEt_3 (11.1 mL, 80.0 mmol, 4.0 eq.) in CH_2Cl_2 (35 mL) and THF (30 mL). Purification by short-path vacuum distillation (high-vacuum, 70 °C) gave the title compound as a deep yellow oil (2.45 g, 16.7 mmol, 84%) which can be stored in the fridge for months.

^{1}H NMR (300 MHz, $CDCl_3$, 300 K): δ_H (ppm) = 7.55 – 7.50 (m, 2H), 7.31 – 7.24 (m, 3H), 2.65 (q, *J* = 7.4 Hz, 2H), 1.45 (t, *J* = 7.4 Hz, 3H).

^{13}C NMR (75 MHz, $CDCl_3$, 300 K): δ_C (ppm) = 205.4 (C_q), 132.9 (C_q), 129.0 (*C*H), 124.2 (*C*H), 124.0 (*C*H), 41.9 (C_q), 17.0 (CH_2), 12.9 (CH_3).

The analytical data are consistent with those reported in the literature.[24]

Methylphenylketene (2b):

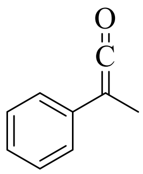

Methylphenylketene **2b** was prepared following general procedure **GP2** using 2-phenylpropanoic acid (1.37 mL, 10.0 mmol, 1.0 eq.), oxalyl chloride (1.29 mL, 15.0 mmol, 1.5 eq.) and NEt_3 (5.55 mL, 40.0 mmol, 4.0 eq.) in CH_2Cl_2 (10 mL) and Et_2O (15 mL). Purification by short-path vacuum distillation (70 °C, 0.47 mbar) gave the title compound as a deep yellow oil (768 mg, 5.81 mmol, 58%).

^{1}H NMR (300 MHz, $CDCl_3$, 300 K): δ_H (ppm) = 7.23 – 7.17 (m, 2H), 6.99 – 6.88 (m, 3H), 1.87 (s, 3H).

^{13}C NMR (75 MHz, $CDCl_3$, 300 K): δ_C (ppm) = 205.6 (C_q), 133.4 (C_q), 129.0 (*C*H), 124.2 (*C*H), 123.7 (*C*H), 123.6 (*C*H), 33.8 (C_q), 8.6 (CH_3).

The analytical data are consistent with those reported in the literature.[25]

Isopropylphenylketene (2c):

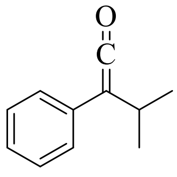

Isopropylphenylketene **2c** was prepared following general procedure **GP2** using 3-methyl-2-phenylbutanoic acid (1.78 g, 10.0 mmol, 1.0 eq.), oxalyl chloride (1.29 mL, 15.0 mmol, 1.5 eq.) and NEt_3 (5.55 mL, 40.0 mmol, 4.0 eq.) in CH_2Cl_2 (10 mL) and Et_2O (15 mL). Purification by short-path vacuum distillation (100 °C, 0.30 mbar) gave the title compound as an inseperable 11.5:1-mixture with residual

acid chloride (NMR yield **2c**: 861 mg, 5.38 mmol, 54%).[4] This mixture was used for the arylation step without further purification and analysis.

Cyclopentylphenylketene (2d):

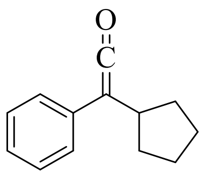

Cyclopentylphenylketene **2d** was prepared following general procedure **GP2** using 2-cyclopentyl-2-phenylacetic acid (2.04 g, 10.0 mmol, 1.0 eq.), oxalyl chloride (1.29 mL, 15.0 mmol, 1.5 eq.) and NEt_3 (5.55 mL, 40.0 mmol, 4.0 eq.) in CH_2Cl_2 (10 mL) and Et_2O (15 mL). Purification by Kugelrohr distillation (170 °C, 0.28 mbar) gave the title compound as an inseparable 1:1-mixture with residual acid chloride (NMR yield **2d**: 427 mg, 2.29 mmol, 23%).[5] This mixture was used for the arylation step without further purification and analysis.

(3,4-Dihydronaphthalen-1(2H)-ylidene)methanone (2e):

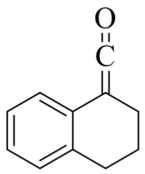

Ketene **2e** was prepared following general procedure **GP2** using 1,2,3,4-tetrahydronaphthalene-1-carboxylic acid (1.76 g, 10.0 mmol, 1.0 eq.), oxalyl chloride (1.29 mL, 15.0 mmol, 1.5 eq.) and NEt_3 (5.54 mL, 40.0 mmol, 4.0 eq.) in CH_2Cl_2 (20 mL) and Et_2O (20 mL). Purification by short-path vacuum distillation (140 °C, 0.34 mbar) gave the title compound as a deep yellow oil (264 mg, 1.67 mmol, 17%).

^{1}H NMR (300 MHz, $CDCl_3$, 300 K): δ_H (ppm) = 7.27 – 7.19 (m, 2H), 7.12 – 7.05 (m, 2H), 2.89 (t, J = 6.2 Hz, 2H), 2.81 – 2.76 (m, 2H), 2.07 – 1.99 (m, 2H).

^{13}C NMR (75 MHz, $CDCl_3$, 300 K): δ_C (ppm) = 206.4 (C_q), 133.5 (C_q), 129.7 (CH), 127.8 (C_q), 126.5 (CH), 124.7 (CH), 123.8 (CH), 34.9 (C_q), 29.6 (CH_2), 22.2 (CH_2), 20.7 (CH_2).

The analytical data are consistent with those reported in the literature.[27]

2-(4-Iodophenyl)but-1-en-1-one (2f):

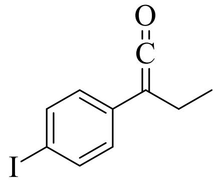

Following a modified procedure from the literature[28], diisopropylamine (6.32 mL, 45.0 mmol, 3.0 eq.) was dissolved in anhydrous THF (20 mL). *n*-BuLi (1.6 M in hexanes, 23.4 mL, 37.5 mmol, 2.5 eq.) was added slowly at 0 °C and the mixture was stirred at room temperature for 30 minutes. After cooling again to 0 °C, a solution of 4-iodophenylacetic acid (3.93 g, 15.0 mmol, 1.0 eq.) in THF (15) was added slowly. The suspension was stirred for one hour at 0 °C, then ethyliodide

---

[4] Determined by ^{1}H-NMR spectroscopy. Ketene **2c** was identified by comparison with spectra from the literature.[25].
[5] Determined by ^{1}H-NMR spectroscopy. Ketene **2d** was identified by comparison with spectra from the literature[2622]

(1.81 mL, 22.5 mmol, 1.5 eq.) was added slowly at 0 °C. The reaction mixture was allowed to reach room temperature overnight and then adjusted to pH 1 by hydrochloric acid (2 N aqueous solution, 5 mL). EtOAc (50 mL) was added and the phases were separated. The aqueous phase was extracted with EtOAc (3 x 50 mL), the combined organic phases were washed with brine (100 mL), dried over $MgSO_4$ and concentrated *in vacuo*. Purification by RP-MPLC (MeOH/water, gradient from 5% to 90%) gave 2-(4-iodophenyl)butanoic acid as a colourless solid (2.07 g, 7.03 mmol, 47%). The acid (2.03 g, 7.00 mmol, 1.0 eq.) was then transformed to the desired ketene following general procedure **GP2** using oxalyl chloride (952 µL, 10.5 mmol, 1.5 eq.) and NEt_3 (3.88 mL, 28.0 mmol, 4.0 eq.) in CH_2Cl_2 (20 mL) and Et_2O (20 mL). Purification by Kugelrohr distillation (125 °C, 0.40 mbar) gave the title compound as a slightly yellow oil (331 mg, 1.22 mmol, 17%) which has been used immediately after preparation.

^{1}H NMR (300 MHz, $CDCl_3$, 300 K): δ_H (ppm) = 7.28 (d, J = 8.2 Hz, 2H), 6.46 (d, J = 8.2 Hz, 2H), 2.08 (q, J = 7.4 Hz, 2H), 0.91 (t, J = 7.4 Hz, 3H).

^{13}C NMR (75 MHz, $CDCl_3$, 300 K): δ_C (ppm) = 204.1 (C_q), 137.8 (CH), 132.7 (C_q), 125.8 (CH), 87.8 (C_q), 41.9 (C_q), 16.9 (CH_2), 12.8 (CH_3).

2-(4-Bromophenyl)but-1-en-1-one (2g):

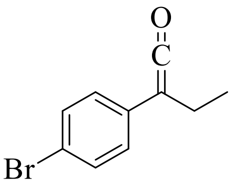

Following a modified procedure from the literature[28], diisopropylamine (8.43 mL, 60.0 mmol, 3.0 eq.) was dissolved in anhydrous THF (25 mL). *n*-BuLi (1.6 M in hexanes, 31.3 mL, 50.0 mmol, 2.5 eq.) was added slowly at 0 °C and the mixture was stirred at room temperature for 30 minutes. After cooling again to 0 °C, a solution of 4-bromophenylacetic acid (4.30 g, 20.0 mmol, 1.0 eq.) in THF (20) was added slowly. The suspension was stirred for one hour at 0 °C, then ethyliodide (2.41 mL, 30.0 mmol, 1.5 eq.) was added slowly at 0 °C. The reaction mixture was allowed to reach room temperature overnight and then adjusted to pH 1 by hydrochloric acid (2 N aqueous solution, 7 mL). EtOAc (50 mL) was added and the phases were separated. The aqueous phase was extracted with EtOAc (3 x 50 mL), the combined organic phases were washed with brine (100 mL), dried over $MgSO_4$ and concentrated *in vacuo*. Purification by RP-MPLC (MeOH/water, gradient from 5% to 90%) gave 2-(4-bromophenyl)butanoic acid as a colourless solid (3.09 g, 12.7 mmol, 64%). The acid (3.10 g, 12.7 mmol, 1.0 eq.) was then transformed to the desired ketene following general procedure **GP2** using oxalyl chloride (1.64 mL, 19.1 mmol, 1.5 eq.) and NEt_3 (7.06 mL, 50.9 mmol, 4.0 eq.) in CH_2Cl_2 (20 mL) and Et_2O (20 mL). Purification by Kugelrohr distillation (100 °C, 0.28 mbar) gave the title compound as

a slightly yellow oil (982 mg, 4.36 mmol, 34%) which has been used immediately after preparation.

^{1}H NMR (300 MHz, $CDCl_3$, 300 K): δ_H (ppm) = 7.30 – 7.25 (m, 2H), 6.78 – 6.73 (m, 2H), 2.27 (q, J = 7.4 Hz, 2H), 1.09 (t, J = 7.4 Hz, 3H).

^{13}C NMR (75 MHz, $CDCl_3$, 300 K): δ_C (ppm) = 204.3 (C_q), 132.0 (C_q and CH)), 125.5 (CH), 117.2 (C_q), 41.8 (C_q), 17.0 (CH$_2$), 12.8 (CH$_3$).

The analytical data are consistent with those reported in the literature.[25]

2-(4-Isobutylphenyl)prop-1-en-1-one (2h):

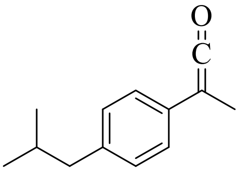

Ibuprofeneketene **2h** was prepared following general procedure **GP2** using ibuprofene (2.06 g, 10.0 mmol, 1.0 eq.), oxalyl chloride (1.72 mL, 20.0 mmol, 2.0 eq.) and NEt_3 (5.54 mL, 40.0 mmol, 4.0 eq.) in CH_2Cl_2 (15 mL) and Et_2O (15 mL). Purification by short-path vacuum distillation (150 °C, 0.28 mbar) gave the title compound as a deep yellow oil (862 mg, 4.58 mmol, 46%).

^{1}H NMR (300 MHz, $CDCl_3$, 300 K): δ_H (ppm) = 7.18 – 7.14 (m, 2H), 7.01 – 6.97 (m, 2H), 2.50 (d, J = 7.2 Hz, 2H), 2.04 (s, 3H), 1.97 – 1.81 (m, 1H), 0.96 (d, J = 6.6 Hz, 6H).

^{13}C NMR (75 MHz, $CDCl_3$, 300 K): δ_C (ppm) = 206.8 (C_q), 137.7 (C_q), 130.3 (C_q), 129.8 (CH), 123.4 (CH), 45.0 (CH$_2$), 33.4 (C_q), 30.4 (CH), 22.4 (CH$_3$), 8.7 (CH$_3$).

The analytical data are consistent with those reported in the literature.[29]

Diphenylketene (2i):

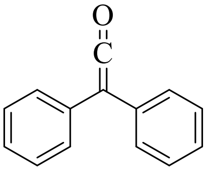

Diphenylketene **2i** was prepared following general procedure **GP2** using 2,2-diphenylacetic acid (2.12 g, 10.0 mmol, 1.0 eq.), oxalyl chloride (1.72 mL, 20.0 mmol, 2.0 eq.) and NEt_3 (5.54 mL, 40.0 mmol, 4.0 eq.) in CH_2Cl_2 (15 mL) and Et_2O (15 mL). Purification by short-path vacuum distillation (150 °C, 0.24 mbar) gave the title compound as a deep yellow oil (1.34 g, 6.88 mmmol, 69%).

^{1}H NMR (300 MHz, $CDCl_3$, 300 K): δ_H (ppm) = 7.34 – 7.28 (m, 4H), 7.19 – 7.13 (m, 6H).

^{13}C NMR (75 MHz, $CDCl_3$, 300 K): δ_C (ppm) = 200.0 (C_q), 129.7 (C_q), 128.2 (CH), 126.6 (CH), 125.1 (CH), 45.8 (C_q).

The analytical data are consistent with those reported in the literature.[30]

Benzylmethylketene (2j):

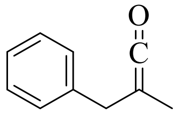

Benzylmethylketene **2j** was prepared following general procedure **GP2** using 2-methyl-3-phenylpropanoic acid (1.64 g, 10.0 mmol, 1.0 eq.), oxalyl chloride (1.29 mL, 15.0 mmol, 1.5 eq.) and NEt_3 (5.55 mL, 40.0 mmol, 4.0 eq.) in CH_2Cl_2 (10 mL) and Et_2O (15 mL). Purification by short-path vacuum distillation (70 °C, 0.47 mbar) gave the title compound as a slightly yellow oil (696 mg, 4.76 mmol, 48%) which has been used immediately after distillation due to rapid decomposition.

^{1}H NMR (300 MHz, $CDCl_3$, 300 K): δ_H (ppm) = 7.36 – 7.24 (m, 5H), 3.29 (s, 2H), 1.59 (s, 3H).

The analytical data are consistent with those reported in the literature.[31]

Cyclohexylmethylketene (2k):

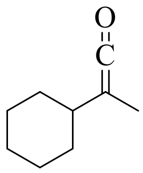

Cyclohexylmethylketene **2k** was prepared following general procedure **GP2** using 2-cyclohexylpropanoyl chloride[6] (1.75 g, 10.0 mmol, 1.0 eq.) and NEt_3 (5.54 mL, 40.0 mmol, 4.0 eq.) in Et_2O (20 mL). Purification by short-path vacuum distillation (60 °C, 0.30 mbar) gave the title compound as a slightly yellow oil (1.09 g, 7.88 mmol, 79%) which has been used immediately after distillation without further purification and analysis due to rapid decomposition.

Cycloheptylidenemethanone (2l):

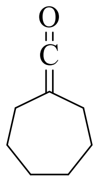

Ketene **2l** was prepared following general procedure **GP2** using Cycloheptanecarboxylic acid (2.40 mL, 20.0 mmol, 1.0 eq.), Oxalyl chloride (3.41 mL, 40.0 mmol, 2.0 eq.) and NEt_3 (11.1 mL, 80.0 mmol, 4.0 eq.) in CH_2Cl_2 (20 mL) and Et_2O (25 mL). Purification by short-path vacuum distillation (70 °C, 0.8 mbar) gave the title compound as a yellow oil (412 mg, 3.32 mmol, 17%) which has been used immediately after distillation due to rapid decomposition.

^{1}H NMR (300 MHz, $CDCl_3$, 300 K): δ_H (ppm) = 2.25 – 2.21 (m, 4H), 1.70 – 1.61 (m, 4H), 1.59 – 1.51 (m, 4H).

The analytical data are consistent with those reported in the literature.[30]

---

[6] 2-Cyclohexylpropanoyl chloride was prepared in two steps from commercially available 2-cyclohexylacetic acid following a procedure from the literature[32].

1.4.3 Synthesis of α-quaternary amides

N-Isopropyl-2,2-diphenylbutanamide (3a):

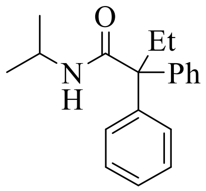

0.2 mmol Scale: The title compound was prepared following the general procedure **GP3** using sulfonamide **1a** (39.9 mg, 0.200 mmol, 1.0 eq.), *n*-BuLi (1.6 M-solution in hexanes, 0.138 mL, 0.220 mmol, 1.1 eq.), ferrocenium tetrafluoroborate (54.7 mg, 0.200 mmol, 1.0 eq.) and ethylphenylketene **2a** (39 µL, 0.30 mmol, 1.5 eq.) in Et_2O (20 mL). Purification by FC (pentane) gave ferrocene (35.7 mg, 0.192 mmol, 96%) as orange needles, the column was flushed with EtOAc and the combined fractions were purified by RP-MPLC (MeOH/H_2O, gradient from 20% to 90%) to give the title compound as a colourless oil (52.9 mg, 0.188 mmol, 94%). The recovered ferrocene has been re-oxidized to ferrocenium tetrafluoroborate following general procedure **GP4**.

5 mmol Scale: An oven-dried 1 L three-necked round bottom flask with a dropping funnel was charged with sulfonamide **1a** (996 mg, 5.00 mmol, 1.0 eq.) under an atmosphere of argon. The dropping funnel was charged with a solution of ethylphenylketene **2a** (1.10 g, 7.50 mmol, 1.5 eq.) in Et_2O (125 mL). Anhydrous Et_2O (375 mL) and *n*-BuLi (1.6 M-solution in hexanes, 3.44 mL, 5.50 mmol, 1.1 eq.) were added subsequently to the round bottom flask under stirring at room temperature. After 30 minutes ferrocenium tetrafluoroborate (1.36 g, 5.00 mmol, 1.0 eq.) was added at room temperature and stirring was continued for further 30 minutes. The ketene solution was dropped to the deep blue solution over 30 minutes. Stirring was continued for 18 hours and the reaction mixture was quenched by the addition of water (200 mL). The Et_2O was removed under reduced pressure and the aqueous residue was extracted with CH_2Cl_2 (5 x 100 mL). The combined organic phases were dried over $MgSO_4$, filtered and concentrated *in vacuo*. Purification by RP-MPLC (MeOH/H_2O, gradient from 20% to 90%) gave the title compound as a mixture with ferrocene. Multiple Co-evaporation with MeOH and EtOAc gave the title compound as a yellowish oil (1.28 g, 4.53 mmol, 91%).

FT-IR (neat): ν (cm^{-1}) = 3428, 3057, 2971, 2938, 2880, 1709, 1661, 1599, 1494, 1455, 1385, 1367, 1325, 1171, 1130, 1084, 1034, 812, 758, 729, 700, 654, 621, 577.

^{1}H NMR (300 MHz, $CDCl_3$, 300 K): δ_H (ppm) = 7.32 – 7.19 (m, 10H), 5.21 (d, *J* = 7.1 Hz, 1H), 4.12 – 3.96 (m, 1H), 2.41 (q, *J* = 7.3 Hz, 2H), 0.97 (d, *J* = 6.6 Hz, 6H), 0.80 (t, *J* = 7.3 Hz, 3H).

^{13}C NMR (75 MHz, $CDCl_3$, 300 K): δ_C (ppm) = 173.5 (C_q), 143.3 (C_q), 129.0 (*C*H), 128.2 (*C*H), 126.7 (*C*H), 61.0 (C_q), 41.6 (*C*H), 31.4 (CH_2), 22.4 (CH_3), 10.1 (CH_3).

HRMS (ESI) *m/z* = 304.1672 calcd. for $[C_{19}H_{23}NONa]^+$ $[M+Na]^+$, found: 304.1671.

***N*-Methyl-2,2-diphenylbutanamide (3ab):**

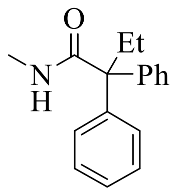

The title compound was prepared following the general procedure **GP3** using sulfonamide **1b** (34.2 mg, 0.200 mmol, 1.0 eq.), *n*-BuLi (1.6 M-solution in hexanes, 0.138 mL, 0.220 mmol, 1.1 eq.), ferrocenium tetrafluoroborate (54.7 mg, 0.200 mmol, 1.0 eq.) and ethylphenylketene **2a** (39 µL, 0.30 mmol, 1.5 eq.) in Et_2O (20 mL). Purification by RP-MPLC ($MeOH/H_2O$, gradient from 20% to 90%) gave the title compound as a colourless solid (28.5 mg, 0.113 mmol, 57%).

MP: 101-103 °C.

FT-IR (neat): ν (cm^{-1}) = 3448, 3368, 3058, 2971, 2938, 2879, 1650, 1600, 1515, 1494, 1447, 1409, 1359, 1264, 1173, 1157, 1080, 1032, 814, 807, 787, 757, 731, 698, 654, 631, 599, 571.

^{1}H NMR (300 MHz, $CDCl_3$, 300 K): δ_H (ppm) = 7.37 – 7.24 (m, 10H), 5.58 – 5.37 (bs, 1H), 2.77 (d, *J* = 4.8 Hz, 3H), 2.47 (q, *J* = 7.3 Hz, 1H), 0.85 (t, *J* = 7.3 Hz, 4H).

^{13}C NMR (75 MHz, $CDCl_3$, 300 K): δ_C (ppm) = 175.2 (C_q), 143.3 (C_q), 129.0 (*C*H), 128.3 (*C*H), 126.8 (*C*H), 61.0 (C_q), 31.5 (CH_2), 26.8 (CH_3), 10.2 (CH_3).

HRMS (ESI) *m/z* = 276.1359 calcd. for $[C_{17}H_{19}NONa]^+$ $[M+Na]^+$, found: 276.1356.

***N*-(2,2,2-Trifluoroethyl)-2,2-diphenylbutanamide (3ac):**

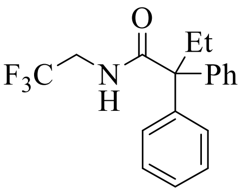

The title compound was prepared following the general procedure **GP3** using sulfonamide **1c** (47.8 mg, 0.200 mmol, 1.0 eq.), *n*-BuLi (1.6 M-solution in hexanes, 0.138 mL, 0.220 mmol, 1.1 eq.), ferrocenium tetrafluoroborate (54.7 mg, 0.200 mmol, 1.0 eq.) and ethylphenylketene **2a** (39 µL, 0.30 mmol, 1.5 eq.) in Et_2O (20 mL). Purification by RP-MPLC ($MeOH/H_2O$, gradient from 20% to 90%) gave the title compound as a colourless solid (49.9 mg, 0.155 mmol, 78%).

MP: 89-91 °C.

FT-IR (neat): ν (cm^{-1}) = 3375, 3061, 2970, 2940, 2885, 1665, 1599, 1514, 1495, 1448, 1421, 1395, 1343, 1275, 1239, 1151, 1093, 1033, 985, 903, 831, 808, 782, 758, 734, 698, 667, 583.

^{1}H NMR (300 MHz, $CDCl_3$, 300 K): δ_H (ppm) = 7.33 – 7.22 (m, 10H), 5.69 (m, 1H), 3.82 (qd, *J* = 9.1, 6.5 Hz, 2H), 2.42 (q, *J* = 7.3 Hz, 2H), 0.79 (t, *J* = 7.3 Hz, 3H).

$^{13}C\{^1H,^{19}F\}$ NMR (151 MHz, $CDCl_3$, 300 K): δ_C (ppm) = 174.7 (C_q), 142.3 (C_q), 128.9 (*C*H), 128.5 (*C*H), 127.1 (*C*H), 124.0 (C_q), 61.2 (C_q), 40.8 (CH_2), 31.5 (CH_2), 9.9 (CH_3).

$^{19}F\{^1H,^{13}C\}$ NMR (564 MHz, $CDCl_3$, 300 K): δ_F (ppm) = −72.4.

HRMS (ESI) *m/z* = 344.1233 calcd. for $[C_{18}H_{18}NOF_3Na]^+$ $[M+Na]^+$, found: 344.1230.

***N*-Benzyl-2,2-diphenylbutanamide (3ad):**

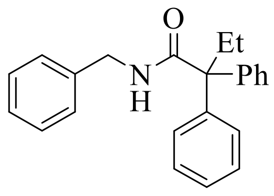

The title compound was prepared following the general procedure **GP3** using sulfonamide **1d** (49.5 mg, 0.200 mmol, 1.0 eq.), *n*-BuLi (1.6 M-solution in hexanes, 0.138 mL, 0.220 mmol, 1.1 eq.), ferrocenium tetrafluoroborate (54.7 mg, 0.200 mmol, 1.0 eq.) and ethylphenylketene **2a** (39 µL, 0.30 mmol, 1.5 eq.) in Et_2O (20 mL). Purification by RP-MPLC ($MeOH/H_2O$, gradient from 20% to 90%) gave the title compound as a colourless solid (50.9 mg, 0.155 mmol, 78%).

MP: 145-146 °C.

FT-IR (neat): ν (cm^{-1}) = 3424, 3369, 3060, 3031, 2971, 2937, 2879, 1678, 1600, 1495, 1453, 1360, 1265, 1169, 1080, 1029, 733, 697, 598, 568.

^1H NMR (300 MHz, $CDCl_3$, 300 K): δ_H (ppm) = 7.34 – 7.23 (m, 13H), 7.05 – 7.02 (m, 2H), 5.86 – 5.72 (bs, 1H), 4.43 (d, *J* = 5.7 Hz, 2H), 2.50 (q, *J* = 7.3 Hz, 2H), 0.84 (t, *J* = 7.3 Hz, 3H).

^{13}C NMR (75 MHz, $CDCl_3$, 300 K): δ_C (ppm) = 174.4 (C_q), 143.0 (C_q), 138.3 (C_q), 129.1 (*C*H), 128.6 (*C*H), 128.3 (*C*H), 127.3 (2 x *C*H), 126.9 (*C*H), 61.2 (C_q), 43.7 (CH_2), 31.5 (CH_2), 10.1 (CH_3).

HRMS (ESI) *m/z* = 352.1672 calcd. for $[C_{23}H_{23}NONa]^+$ $[M+Na]^+$, found: 352.1668.

***N*-Cyclohexyl-2,2-diphenylbutanamide (3ae):**

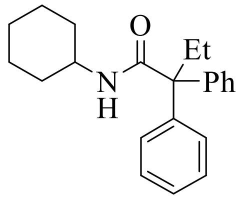

The title compound was prepared following the general procedure **GP3** using sulfonamide **1e** (47.9 mg, 0.200 mmol, 1.0 eq.), *n*-BuLi (1.6 M-solution in hexanes, 0.138 mL, 0.220 mmol, 1.1 eq.), ferrocenium tetrafluoroborate (54.7 mg, 0.200 mmol, 1.0 eq.) and ethylphenylketene **2a** (39 µL, 0.30 mmol, 1.5 eq.) in Et_2O (20 mL). Purification by RP-MPLC ($MeOH/H_2O$, gradient from 20% to 90%) gave the title compound as a colourless solid (63.2 mg, 0.197 mmol, 98%).

MP: 97 °C.

FT-IR (neat): ν (cm^{-1}) = 3423, 3058, 3034, 2972, 2931, 2855, 1658, 1599, 1493, 1448, 1380, 1313, 1265, 1226, 1152, 1088, 1033, 892, 827, 732, 699, 656.

^{1}H NMR (300 MHz, $CDCl_3$, 300 K): δ_H (ppm) = 7.34 – 7.24 (m, 10H), 5.34 (d, *J* = 8.2 Hz, 1H), 3.86 – 3.74 (m, 1H), 2.45 (q, *J* = 7.3 Hz, 2H), 1.81 – 1.75 (m, 2H), 1.57 – 1.52 (m, 3H), 1.38 – 1.25 (m, 2H), 1.13 – 0.92 (m, 3H), 0.85 (t, *J* = 7.3 Hz, 3H).

^{13}C NMR (75 MHz, $CDCl_3$, 300 K): δ_C (ppm) = 173.4 (C_q), 143.4 (C_q), 129.0 (*C*H), 128.2 (*C*H), 126.7 (*C*H), 61.0 (C_q), 48.2 (*C*H), 32.6 (CH_2), 31.4 (CH_2), 25.5 (CH_2), 24.5 (CH_2), 10.1 (CH_3).

HRMS (ESI) *m/z* = 344.1985 calcd. for $[C_{22}H_{27}NONa]^+$ $[M+Na]^+$, found: 344.1991.

***N*-(2,2-Dimethyl-1,3-dioxan-5-yl)-2,2-diphenylbutanamide (3af):**

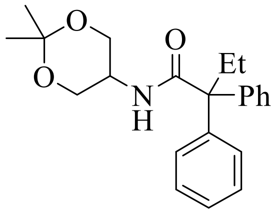

The title compound was prepared following the general procedure **GP3** using sulfonamide **1f** (54.3 mg, 0.200 mmol, 1.0 eq.), *n*-BuLi (1.6 M-solution in hexanes, 0.138 mL, 0.220 mmol, 1.1 eq.), ferrocenium tetrafluoroborate (54.7 mg, 0.200 mmol, 1.0 eq.) and ethylphenylketene **2a** (39 µL, 0.30 mmol, 1.5 eq.) in Et_2O (20 mL). Purification by RP-MPLC ($MeOH/H_2O$, gradient from 20% to 90%) gave the title compound as a colourless oil (38.0 mg, 0.108 mmol, 54%).

FT-IR (neat): ν (cm^{-1}) = 3420, 2987, 2939, 2879, 2249, 1658, 1494, 1449, 1261, 1243, 1197, 1153, 1083, 1034, 984, 907, 821, 728, 700, 848, 598.

^{1}H NMR (300 MHz, $CDCl_3$, 300 K): δ_H (ppm) = 7.38 – 7.25 (m, 10H), 6.53 (d, *J* = 8.2 Hz, 1H), 4.03 (d, *J* = 11.8 Hz, 2H), 3.82 – 3.79 (m, 1H), 3.61 (d, *J* = 11.8 Hz, 2H), 2.49 (q, *J* = 7.3 Hz, 2H), 1.41 (s, 3H), 1.20 (s, 3H), 0.86 (t, *J* = 7.3 Hz, 3H).

^{13}C NMR (75 MHz, $CDCl_3$, 300 K): δ_C (ppm) = 174.2 (C_q), 143.0 (C_q), 129.1 (*C*H), 128.3 (*C*H), 126.9 (*C*H), 98.4 (C_q), 63.4 (CH_2), 61.1 (C_q), 44.0 (*C*H), 31.2 (CH_2), 28.9 (CH_3), 18.3 (CH_3), 10.1 (CH_3).

HRMS (ESI) *m/z* = 376.1883 calcd. for $[C_{22}H_{27}NO_3Na]^+$ $[M+Na]^+$, found: 376.1882.

***N*-(*tert*-Butyl)-2,2-diphenylbutanamide (3ag):**

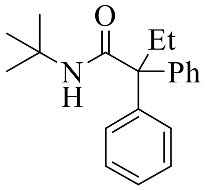

The title compound was prepared following the general procedure **GP3** using sulfonamide **1g** (42.7 mg, 0.200 mmol, 1.0 eq.), *n*-BuLi (1.6 M-solution in hexanes, 0.138 mL, 0.220 mmol, 1.1 eq.), ferrocenium tetrafluoroborate (54.7 mg, 0.200 mmol, 1.0 eq.) and ethylphenylketene **2a** (39 µL, 0.30 mmol, 1.5 eq.) in Et_2O (20 mL). Purification by RP-MPLC (MeOH/H_2O, gradient from 5% to 70%) gave the title compound as a colourless oil (19.3 mg, 65.3 µmol, 33%).

FT-IR (neat): ν (cm^{-1}) = 3426, 2967, 1671, 1599, 1507, 1450, 1364, 1263, 1215, 1085, 1033, 911, 756, 730, 701, 553.

^{1}H NMR (500 MHz, $CDCl_3$, 300 K): δ_H (ppm) = 7.35 – 7.24 (m, 10H), 5.27 (s, 1H), 2.43 (q, *J* = 7.3 Hz, 2H), 1.24 (s, 9H), 0.84 (t, *J* = 7.3 Hz, 3H).

^{13}C NMR (126 MHz, $CDCl_3$, 300 K): δ_C (ppm) = 173.6 (C_q), 143.6 (C_q), 128.9 (*C*H), 128.2 (*C*H), 126.6 (*C*H), 61.4 (C_q), 51.3 (C_q), 31.5 (CH_2), 28.5 (CH_3), 10.1 (CH_3).

HRMS (ESI) *m/z* = 318.1828 calcd. for $[C_{20}H_{25}NONa]^+$ $[M+Na]^+$, found: 318.1828.

***N*-Phenyl-2,2-diphenylbutanamide (3ah):**

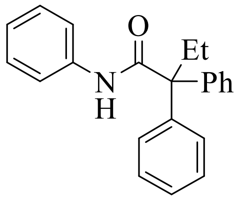

The title compound was prepared following the general procedure **GP3** using sulfonamide **1h** (46.7 mg, 0.200 mmol, 1.0 eq.), *n*-BuLi (1.6 M-solution in hexanes, 0.138 mL, 0.220 mmol, 1.1 eq.), ferrocenium tetrafluoroborate (54.7 mg, 0.200 mmol, 1.0 eq.) and ethylphenylketene **2a** (39 µL, 0.30 mmol, 1.5 eq.) in Et_2O (20 mL). Purification by RP-MPLC (MeOH/H_2O, gradient from 20% to 90%) gave the title compound as a colourless oil (16.4 mg, 0.052 mmol, 26%).

^{1}H NMR (300 MHz, $CDCl_3$, 300 K): δ_H (ppm) = 7.43 – 7.24 (m, 14H), 7.19 – 7.13 (m, 1H), 7.10 – 7.05 (m, 1H), 2.55 (q, *J* = 7.3 Hz, 2H), 0.88 (t, *J* = 7.3 Hz, 3H).

^{13}C NMR (75 MHz, $CDCl_3$, 300 K): δ_C (ppm) = 172.6 (C_q), 142.7 (C_q), 137.8 (C_q), 129.0 (*C*H), 128.9 (*C*H), 128.6 (*C*H), 127.1 (*C*H), 124.3 (*C*H), 119.7 (*C*H), 62.0 (C_q), 31.6 (CH_2), 10.1 (CH_3).

HRMS (ESI) *m/z* = 338.1515 calcd. for $[C_{22}H_{21}NONa]^+$ $[M+Na]^+$, found: 338.1527.

The analytical data are consistent with those reported in the literature.[33]

2-Phenyl-*N*-(*(S)*-1-phenylethyl)-2-(p-tolyl)butanamide (3ai):

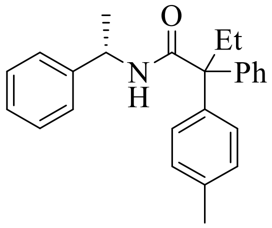

The title compound was prepared following the general procedure **GP3** using sulfonamide **1i** (55.1 mg, 0.200 mmol, 1.0 eq.), *n*-BuLi (1.6 M-solution in hexanes, 0.138 mL, 0.220 mmol, 1.1 eq.), ferrocenium tetrafluoroborate (54.7 mg, 0.200 mmol, 1.0 eq.) and ethylphenylketene **2a** (39 µL, 0.30 mmol, 1.5 eq.) in Et_2O (20 mL). Purification by RP-MPLC (MeOH/H_2O, gradient from 20% to 90%) gave the title compound as a colourless oil (1:1 mixture of diastereoisomers, 56.0 mg, 0.157 mmol, 79%).

FT-IR (neat): ν (cm^{-1}) = 3421, 3027, 2971, 2934, 2878, 1857, 1664, 1599, 1494, 1446, 1377, 1353, 1233, 1167, 1089, 1021, 960, 914, 890, 814, 762, 735, 696, 667, 608, 583.

^{1}H NMR (300 MHz, $CDCl_3$, 300 K): δ_H (ppm) = 7.35 – 7.02 (m, 14H, both diastereoisomers), 5.68 (d, *J* = 7.9 Hz, 1H, both diastereoisomers), 5.16 – 5.06 (m, 1H, both diastereoisomers), 2.47 – 2.35 (m, 5H, both diastereoisomers), 1.35 – 1.32 (m, 3H, both diastereoisomers), 0.83 – 0.78 (m, 3H, both diastereoisomers).

^{13}C NMR (151 MHz, $CDCl_3$, 300 K): δ_C (ppm) = 173.6 (2 x C_q), 143.3 (C_q), 143.1 (C_q), 140.1 (C_q), 139.8 (C_q), 136.4 (2 x C_q), 129.0 (3 x *C*H), 128.9 (2 x *C*H), 128.4 (*C*H), 128.2 (2 x *C*H), 127.0 (*C*H), 126.7 (2 x *C*H), 125.8 (2 x *C*H), 60.8 (C_q), 48.8 (*C*H), 31.4 (2 x CH_2), 21.7 (2 x CH_3), 20.9 (2 x CH_3), 10.0 (CH_3).[7]

HRMS (ESI) *m/z* = 380.1985 calcd. for $[C_{25}H_{27}NONa]^+$ $[M+Na]^+$, found: 380.1984.

Methyl (2-phenyl-2-(p-tolyl)butanoyl)-*L*-valinate (3aj):

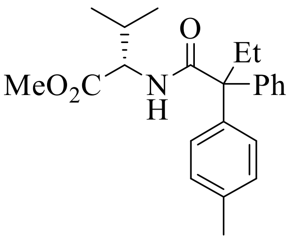

The title compound was prepared following the general procedure **GP3** using sulfonamide **1j** (57.1 mg, 0.200 mmol, 1.0 eq.), *n*-BuLi (1.6 M-solution in hexanes, 0.138 mL, 0.220 mmol, 1.1 eq.), ferrocenium tetrafluoroborate (54.7 mg, 0.200 mmol, 1.0 eq.) and ethylphenylketene **2a** (39 µL, 0.30 mmol) in Et_2O (20 mL). Purification by RP-MPLC (MeOH/H_2O, gradient from 20% to 90%) gave the title compound as a colourless oil (1:1 mixture of diastereoisomers, 58.8 mg, 0.160 mmol, 80%).

FT-IR (neat): ν (cm^{-1}) = 3449, 2958, 2933, 2872, 1741, 1665, 1600, 1495, 1466, 1446, 1371, 1323, 1265, 1155, 1093, 1036, 963, 930, 907, 814, 736, 701, 674, 568.

[7] Some signals of the diastereoisomers are superimposed.

^{1}H NMR (300 MHz, $CDCl_3$, 300 K): δ_H (ppm) = 7.35 – 7.10 (m, 9H, both diastereoisomers), 5.91 (d, *J* = 8.6 Hz, 1H, both diastereoisomers), 4.52 – 4.46 (m, 1H, both diastereoisomers), 3.65 (m, 3H, both diastereoisomers), 2.52 – 2.31 (m, 5H, both diastereoisomers), 2.10 – 1.99 (m, 1H, both diastereoisomers), 0.81 – 0.69 (m, 6H, both diastereoisomers), 0.62 – 0.59 (m, 3H, both diastereoisomers).

^{13}C NMR (151 MHz, $CDCl_3$, 300 K): δ_C (ppm) = 174.5 (2 x C_q), 172.3 (C_q), 172.2 (C_q), 143.5 (C_q), 142.8 (C_q), 140.2 (C_q), 139.4 (C_q), 136.5 (2 x C_q), 129.2 (*C*H), 129.1 (*C*H), 128.9 (4 x *C*H), 128.2 (*C*H), 128.1 (*C*H), 126.8 (2 x *C*H), 60.9 (2 x C_q), 57.3 (*C*H), 57.2 (*C*H), 52.0 (CH_3), 31.3 (2 x CH_2), 30.9 (2 x *C*H), 20.9 (2 x CH_3), 18.9 (2 x CH_3), 17.4 (2 x CH_3), 10.0 (2 x CH_3).[4]

HRMS (ESI) *m/z* = 390.2040 calcd. for $[C_{23}H_{29}NO_3Na]^+$ $[M+Na]^+$, found: 390.2036.

***N*-(*(1S,2S)*-2-methoxy-1,2-diphenylethyl)-2-phenyl-2-(p-tolyl)butanamide (3al):**

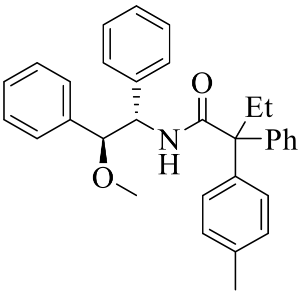

The title compound was prepared following the general procedure **GP3** using sulfonamide **1l** (76.3 mg, 0.200 mmol, 1.0 eq.), *n*-BuLi (1.6 M-solution in hexanes, 0.138 mL, 0.220 mmol, 1.1 eq.), lithium chloride (50.9 mg, 1.20 mmol, 6.0 eq.), ferrocenium tetrafluoroborate (54.7 mg, 0.200 mmol, 1.0 eq.) and ethylphenylketene **2a** (39 µL, 0.30 mmol) in Et_2O (20 mL). Purification by RP-MPLC (MeOH/H_2O, gradient from 50% to 90%) gave the title compound as a colourless oil (1:1 mixture of diastereoisomers, 31.8 mg, 0.069 mmol, 34%).

FT-IR (neat): ν (cm^{-1}) = 3430, 3059, 3030, 2975, 2829, 1670, 1600, 1492, 1453, 1349, 1332, 1185, 1094, 1030, 977, 913, 839, 814, 765, 737, 698, 667, 606.

^{1}H NMR (300 MHz, $CDCl_3$, 300 K): δ_H (ppm) = 7.35 – 6.90 (m, 19H, both diastereoisomers), 6.48 – 6.45 (m, 1H, both diastereoisomers), 5.06 – 5.04 (m, 1H, both diastereoisomers), 4.26 – 4.24 (m, 1H, both diastereoisomers), 3.04 (m, 3H, both diastereoisomers), 2.41 – 2.25 (m, 5H, both diastereoisomers), 0.70 (t, *J* = 7.3 Hz, 3H, both diastereoisomers).

^{13}C NMR (75 MHz, $CDCl_3$, 300 K): δ_C (ppm) = 173.7 (C_q), 143.7 (C_q), 143.1 (C_q), 140.5 (C_q), 140.4 (C_q), 139.9 (C_q), 138.4 (C_q), 136.4 (C_q), 136.3 (C_q), 129.3 (*C*H), 129.2 (*C*H), 129.1 (*C*H), 129.0 (*C*H), 128.9 (*C*H), 128.3 (3 x *C*H), 128.2 (*C*H), 128.0 (*C*H), 127.8 (*C*H), 127.1 (*C*H),

126.9 (*C*H), 126.8 (2 x *C*H), 126.7 (*C*H), 126.6 (*C*H), 94.1 (*C*H_2), 79.7 (*C*H), 60.7 (C_q), 58.9 (*C*H), 55.4 (*C*H_3), 31.5 (2 x *C*H_2), 21.1 (*C*H_3), 21.0 (*C*H_3), 10.0 (2 x *C*H_3).[8]

HRMS (ESI) *m/z* = 486.2404 calcd. for $[C_{32}H_{33}NO_2Na]^+$ $[M+Na]^+$, found: 486.2402.

***N*-(*(1S,2S)*-2-(benzyloxy)-1,2-diphenylethyl)-2-phenyl-2-(p-tolyl)butanamide (3am):**

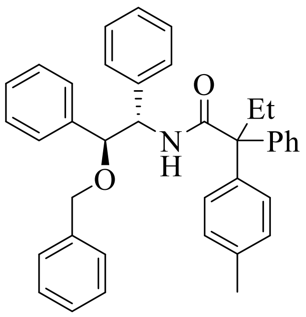

The title compound was prepared following the general procedure **GP3** using sulfonamide **1m** (91.5 mg, 0.200 mmol, 1.0 eq.), *n*-BuLi (1.6 M-solution in hexanes, 0.138 mL, 0.220 mmol, 1.1 eq.), lithium chloride (50.9 mg, 1.20 mmol, 6.0 eq.), ferrocenium tetrafluoroborate (54.7 mg, 0.200 mmol, 1.0 eq.) and ethylphenylketene **2a** (39 µL, 0.30 mmol) in Et_2O (20 mL). Purification by RP-MPLC (MeOH/H_2O, gradient from 50% to 90%) gave the title compound as a colourless oil (1:1 mixture of diastereoisomers, 48.1 mg, 0.089 mmol, 45%).

FT-IR (neat): ν (cm^{-1}) = 3428, 3062, 3029, 2971, 2876, 1671, 1602, 1493, 1453, 1378, 1346, 1205, 1158, 1093, 1070, 1028, 912, 814, 765, 735, 698, 616, 587.

^{1}H NMR (300 MHz, $CDCl_3$, 300 K): δ_H (ppm) = 7.27 – 6.89 (m, 24H, both diastereoisomers), 6.58 – 6.56 (m, 1H, both diastereoisomers), 5.09 – 5.05 (m, 1H, both diastereoisomers), 4.46 – 4.44 (m, 1H, both diastereoisomers), 4.32 – 4.28 (m, 1H, both diastereoisomers), 4.03 – 4.02 (m, 1H, both diastereoisomers), 3.99 – 3.98 (m, 1H, both diastereoisomers), 2.36 – 2.22 (m, 5H, both diastereoisomers), 0.69 (t, *J* = 7.3 Hz, 3H, both diastereoisomers).

^{13}C NMR (75 MHz, $CDCl_3$, 300 K): δ_C (ppm) = 173.7 (C_q) , 143.8 (C_q), 143.2 (C_q), 140.8 (C_q), 140.6 (C_q), 140.0 (C_q), 138.7 (C_q), 137.7 (C_q), 136.3 (C_q), 136.2 (C_q), 129.3 (*C*H), 129.2 (*C*H), 129.0 (2 x *C*H), 128.9 (2 x *C*H), 128.4 (2 x *C*H), 128.2 (3 x *C*H), 128.1 (*C*H), 128.0 (*C*H), 127.9 (*C*H), 127.8 (*C*H), 127.4 (*C*H), 127.2 (*C*H), 127.1 (*C*H), 127.0 (*C*H), 126.8 (*C*H), 126.7 (*C*H), 126.6 (2 x *C*H), 83.4 (*C*H), 70.9 (2 x *C*H_2), 60.7 (C_q), 59.2 (*C*H), 31.5 (*C*H_2), 31.4 (*C*H_2), 21.0 (2 x *C*H_3), 10.0 (*C*H_3).[9]

HRMS (ESI) *m/z* = 562.2717 calcd. for $[C_{38}H_{37}NO_2Na]^+$ $[M+Na]^+$, found: 562.2714.

[8] Some signals of the diastereoisomers are superimposed.
[9] Some signals of the diastereoisomers are superimposed.

***N*-((1S,2S)-2-(methoxymethoxy)-1,2-diphenylethyl)-2-phenyl-2-(p-tolyl)butanamide (3an):**

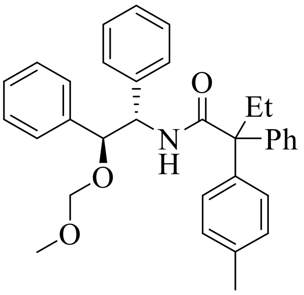

The title compound was prepared following the general procedure **GP3** using sulfonamide **1n** (82.3 mg, 0.200 mmol, 1.0 eq.), *n*-BuLi (1.6 M-solution in hexanes, 0.138 mL, 0.220 mmol, 1.1 eq.), lithium chloride (50.9 mg, 1.20 mmol, 6.0 eq.), ferrocenium tetrafluoroborate (54.7 mg, 0.200 mmol, 1.0 eq.) and ethylphenylketene **2a** (39 µL, 0.30 mmol) in Et_2O (20 mL). Purification by RP-MPLC (MeOH/H_2O, gradient from 50% to 90%) gave the title compound as a colourless oil (1:1 mixture of diastereoisomers, 68.3 mg, 0.138 mmol, 69%).

FT-IR (neat): ν (cm^{-1}) = 3429, 3062, 3029, 3968, 2929, 2888, 1729, 1671, 1601, 1492, 1452, 1380, 1266, 1205, 1151, 1102, 1077, 1022, 922, 842, 814, 766, 736, 699, 607.

^{1}H NMR (300 MHz, $CDCl_3$, 300 K): δ_H (ppm) = 7.35 – 7.03 (m, 19H, both diastereoisomers), 6.47 (d, *J* = 8.2 Hz, 1H, both diastereoisomers), 5.21 – 5.16 (m, 1H, both diastereoisomers), 4.77 (t, *J* = 2.6 Hz, 1H, both diastereoisomers), 4.32 – 4.21 (m, 2H, both diastereoisomers), 2.76 (s, 3H, both diastereoisomers), 2.42 – 2.25 (m, 5H, both diastereoisomers), 0.71 (t, *J* = 7.3 Hz, 3H, both diastereoisomers).

^{13}C NMR (75 MHz, $CDCl_3$, 300 K): δ_C (ppm) = 173.7 (C_q), 143.7 (C_q), 143.1 (C_q), 140.5 (C_q), 140.4 (C_q), 139.9 (C_q), 138.4 (C_q), 136.4 (C_q), 136.3 (C_q), 129.3 (*C*H), 129.2 (*C*H), 129.1 (*C*H), 129.0 (*C*H), 128.9 (*C*H), 128.3 (3 x *C*H), 128.2 (*C*H), 128.0 (*C*H), 127.8 (*C*H), 127.1 (*C*H), 126.9 (*C*H), 126.8 (2 x *C*H), 126.7 (*C*H), 126.6 (*C*H), 94.1 (CH_2), 79.7 (*C*H), 60.7 (C_q), 58.9 (*C*H), 55.4 (CH_3), 31.5 (2 x CH_2), 21.1 (CH_3), 21.0 (CH_3), 10.0 (2 x CH_3).[10]

HRMS (ESI) *m/z* = 516.2509 calcd. for $[C_{33}H_{35}NO_3Na]^+$ $[M+Na]^+$, found: 516.2521.

***N*-Isopropyl-2-phenyl-2-(p-tolyl)butanamide (3ao):**

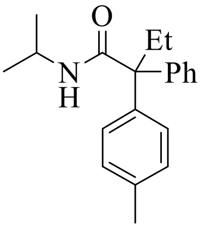

The title compound was prepared following the general procedure **GP3** using sulfonamide **1o** (42.7 mg, 0.200 mmol, 1.0 eq.), *n*-BuLi (1.6 M-solution in hexanes, 0.138 mL, 0.220 mmol, 1.1 eq.), ferrocenium tetrafluoroborate (54.7 mg, 0.200 mmol, 1.0 eq.) and ethylphenylketene **2a** (39 µL, 0.30 mmol,

[10] Some signals of the diastereoisomers are superimposed.

1.5 eq.) in Et_2O (20 mL). Purification by RP-MPLC ($MeOH/H_2O$, gradient from 20% to 90%) gave the title compound as a colourless oil (41.1 mg, 0.139 mmol, 70%).

FT-IR (neat): ν (cm^{-1}) = 3425, 3058, 3026, 2970, 2934, 2877, 1745, 1693, 1600, 1510, 1455, 1385, 1367, 1329, 1265, 1235, 1170, 1120, 1099, 1076, 1034, 1022, 973, 935, 812, 766, 735, 700, 585, 556.

^{1}H NMR (300 MHz, $CDCl_3$, 300 K): δ_H (ppm) = 7.28 – 7.08 (m, 9H), 5.23 (d, *J* = 8.3 Hz, 1H), 4.10 – 3.98 (m, 1H), 2.39 (q, *J* = 7.3 Hz, 2H), 2.31 (s, 3H), 0.98 (d, *J* = 6.6 Hz, 6H), 0.79 (t, *J* = 7.3 Hz, 3H).

^{13}C NMR (75 MHz, $CDCl_3$, 300 K): δ_C (ppm) = 173.6 (C_q), 143.4 (C_q), 140.2 (C_q), 136.3 (C_q), 129.0 (*C*H), 128.9 (2 x *C*H), 128.2 (*C*H), 126.6 (*C*H), 60.6 (C_q), 41.6 (*C*H), 31.4 (CH_2), 22.4 (CH_3), 21.0 (CH_3), 10.1 (CH_3).

HRMS (ESI) *m/z* = 318.1828 calcd. for $[C_{20}H_{25}NONa]^+$ $[M+Na]^+$, found: 318.1838.

2-(4-Fluorophenyl)-*N*-isopropyl-2-phenylbutanamide (3ap):

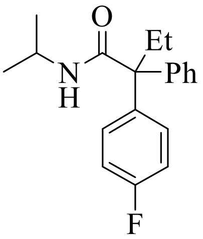

The title compound was prepared following the general procedure **GP3** using sulfonamide **1p** (43.5 mg, 0.200 mmol, 1.0 eq.), *n*-BuLi (1.6 M-solution in hexanes, 0.138 mL, 0.220 mmol, 1.1 eq.), ferrocenium tetrafluoroborate (54.7 mg, 0.200 mmol, 1.0 eq.) and ethylphenylketene **2a** (39 µL, 0.30 mmol, 1.5 eq.) in Et_2O (20 mL). Purification by RP-MPLC ($MeOH/H_2O$, gradient from 20% to 90%) gave the title compound as a colourless oil (37.7 mg, 0.126 mmol, 63%).

FT-IR (neat): ν (cm^{-1}) = 3429, 2972, 2937, 1658, 1605, 1508, 1458, 1233, 1164, 1129, 1085, 906, 835, 725, 648, 578.

^{1}H NMR (300 MHz, $CDCl_3$, 300 K): δ_H (ppm) = 7.34 – 7.22 (m, 7H), 7.02 – 6.96 (m, 2H), 5.20 (d, *J* = 7.7 Hz, 1H), 4.11 – 4.00 (m, 1H), 2.40 (q, *J* = 7.3 Hz, 2H), 1.00 (d, *J* = 6.6 Hz, 6H), 0.81 (t, *J* = 7.3 Hz, 3H).

^{13}C NMR (75 MHz, $CDCl_3$, 300 K): δ_C (ppm) = 173.3 (C_q), 161.5 (d, *J* = 246.2 Hz, C_q), 143.1 (C_q), 139.0 (d, *J* = 3.5 Hz, C_q), 130.6 (d, *J* = 7.8 Hz, *C*H), 128.6 (d, *J* = 40.1 Hz, *C*H), 126.9 (*C*H), 115.0 (d, J = 21.1 Hz), 60.4 (C_q), 41.7 (*C*H), 31.5 (CH_2), 22.4 (CH_3), 10.1 (CH_3).

^{19}F NMR (283 MHz, $CDCl_3$, 300 K): δ_F (ppm) = −116.1.

HRMS (ESI) *m/z* = 322.1578 calcd. for $[C_{19}H_{22}NOFNa]^+$ $[M+Na]^+$, found: 322.1577.

2-(4-Chlorophenyl)-*N*-isopropyl-2-phenylbutanamide (3aq):

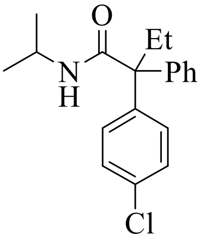

The title compound was prepared following the general procedure **GP3** using sulfonamide **1q** (46.7 mg, 0.200 mmol, 1.0 eq.), *n*-BuLi (1.6 M-solution in hexanes, 0.138 mL, 0.220 mmol, 1.1 eq.), ferrocenium tetrafluoroborate (54.7 mg, 0.200 mmol, 1.0 eq.) and ethylphenylketene **2a** (39 µL, 0.30 mmol, 1.5 eq.) in Et_2O (20 mL). Purification by RP-MPLC (MeOH/H_2O, gradient from 20% to 90%) gave the title compound as a colourless oil (52.9 mg, 0.168 mmol, 84%).

FT-IR (neat): ν (cm^{-1}) = 3431, 2972, 2938, 2879, 2250, 1658, 1493, 1455, 1237, 1170, 1129, 1097, 1014, 905, 828, 725, 648.

^{1}H NMR (300 MHz, $CDCl_3$, 300 K): δ_H (ppm) = 7.36 – 7.22 (m, 9H), 5.21 (d, *J* = 7.8 Hz, 1H), 4.14 – 3.98 (m, 1H), 2.40 (q, *J* = 7.3 Hz, 2H), 1.01 (d, *J* = 6.5 Hz, 6H), 0.82 (t, *J* = 7.3 Hz, 3H).

^{13}C NMR (75 MHz, $CDCl_3$, 300 K): δ_C (ppm) = 173.0 (C_q), 142.9 (C_q), 141.9 (C_q) , 132.6(C_q), 130.4 (*C*H), 128.9 (*C*H), 128.4 (*C*H), 128.3 (*C*H), 127.0 (*C*H), 60.5 (C_q), 41.7 (*C*H), 31.4 (CH_2), 22.4 (CH_3), 10.0 (CH_3).

HRMS (ESI) *m/z* = 338.1282 calcd. for $[C_{19}H_{22}NOClNa]^+$ $[M+Na]^+$, found: 338.1282.

***N*-Isopropyl-2-(4-methoxyphenyl)-2-phenylbutanamide (3ar):**

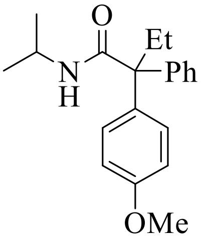

The title compound was prepared following the general procedure **GP3** using sulfonamide **1r** (45.9 mg, 0.200 mmol, 1.0 eq.), *n*-BuLi (1.6 M-solution in hexanes, 0.138 mL, 0.220 mmol, 1.1 eq.), ferrocenium tetrafluoroborate (54.7 mg, 0.200 mmol, 1.0 eq.) and ethylphenylketene **2a** (39 µL, 0.30 mmol, 1.5 eq.) in Et_2O (20 mL). Purification by RP-MPLC (MeOH/H_2O, gradient from 20% to 90%) gave the title compound as a colourless oil (42.8 mg, 0.137 mmol, 69%).

FT-IR (neat): ν (cm^{-1}) = 3421, 2972, 2938, 1657, 1510, 1384, 1295, 1251, 1194, 1160, 1129, 1088, 1034, 906, 831, 807, 724, 675, 647, 590, 560.

^{1}H NMR (300 MHz, $CDCl_3$, 300 K): δ_H (ppm) = 7.30 – 7.16 (m, 7H), 6.82 (d, *J* = 8.8 Hz, 2H), 5.22 (d, *J* = 7.9 Hz, 1H), 4.10 – 3.98 (m, 1H), 3.78 (s, 3H), 2.36 (q, *J* = 7.3 Hz, 2H), 0.98 (d, *J* = 6.5 Hz, 5H), 0.80 (t, *J* = 7.3 Hz, 3H).

^{13}C NMR (75 MHz, $CDCl_3$, 300 K): δ_C (ppm) = 173.8 (C_q), 158.2 (C_q), 143.5 (C_q), 135.2 (C_q), 130.1 (*C*H), 128.9 (*C*H), 128.2 (*C*H), 126.7 (*C*H), 113.5 (*C*H), 60.3 (C_q), 55.2 (CH_3), 41.6 (*C*H), 31.5 (CH_2), 22.5 (CH_3), 22.4 (CH_3), 10.1 (CH_3).

HRMS (ESI) *m/z* = 334.1778 calcd. for $[C_{20}H_{25}NO_2Na]^+$ $[M+Na]^+$, found: 334.1774.

***N*-Isopropyl-2-phenyl-2-(4-(trifluoromethyl)phenyl)butanamide (3as):**

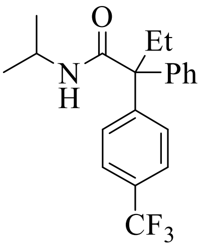

The title compound was prepared following the general procedure **GP3** using sulfonamide **1s** (53.5 mg, 0.200 mmol, 1.0 eq.), *n*-BuLi (1.6 M-solution in hexanes, 0.138 mL, 0.220 mmol, 1.1 eq.), ferrocenium tetrafluoroborate (54.7 mg, 0.200 mmol, 1.0 eq.) and ethylphenylketene **2a** (39 µL, 0.30 mmol, 1.5 eq.) in Et_2O (20 mL). Purification by RP-MPLC ($MeOH/H_2O$, gradient from 20% to 90%) gave the title compound as a colourless oil (60.7 mg, 0.174 mmol, 87%).

FT-IR (neat): ν (cm^{-1}) = 3428, 3359, 2974, 2938, 2881, 1657, 1650, 1619, 1510, 1504, 1455, 1411, 1386, 1366, 1324, 1240, 1165, 1117, 1069, 1017, 910, 836, 812, 772, 730, 700, 669, 602.

^{1}H NMR (300 MHz, $CDCl_3$, 300 K): δ_H (ppm) = 7.55 – 7.52 (m, 2H), 7.42 – 7.40 (m, 2H), 7.34 – 7.22 (m, 5H), 5.18 (d, *J* = 7.8 Hz, 1H), 4.10 – 3.99 (m, 1H), 2.48 – 2.32 (m, 2H), 0.99 (d, *J* = 6.4 Hz, 6H), 0.81 (t, *J* = 7.3 Hz, 3H).

^{13}C{^{1}H,^{19}F} NMR (151 MHz, $CDCl_3$, 300 K): δ_C (ppm) = 172.6 (C_q), 147.5 (C_q) , 142.6 (C_q), 129.4 (*C*H), 128.9 (*C*H), 128.5 (*C*H), 127.2 (*C*H), 125.0 (*C*H), 124.1 (C_q), 60.9 (C_q), 41.8 (*C*H), 31.4 (CH_2), 22.4 (2 x CH_3), 10.0 (CH_3).

^{19}F{^{1}H,^{13}C} NMR (564 MHz, $CDCl_3$, 300 K): δ_F (ppm) = −62.5.

HRMS (ESI) *m/z* = 372.1546 calcd. for $[C_{20}H_{22}NOF_3Na]^+$ $[M+Na]^+$, found: 372.1543.

***N*-Isopropyl-2-(4-nitrophenyl)-2-phenylbutanamide (3at):**

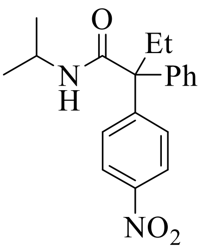

The title compound was prepared following the general procedure **GP3** using sulfonamide **1t** (48.9 mg, 0.200 mmol, 1.0 eq.), *n*-BuLi (1.6 M-solution in hexanes, 0.138 mL, 0.220 mmol, 1.1 eq.), ferrocenium tetrafluoroborate (54.7 mg, 0.200 mmol, 1.0 eq.) and ethylphenylketene **2a** (39 µL, 0.30 mmol, 1.5 eq.) in Et_2O (20 mL). Purification by RP-MPLC ($MeOH/H_2O$, gradient from 20% to 90%) gave the title compound as a colourless oil (25.0 mg, 76.6 µmol, 38%).

FT-IR (neat): ν (cm^{-1}) = 3422, 3052, 2971, 2932, 2876, 1734, 1662, 1599, 1576, 1559,1507, 1491, 1457, 1399, 1387, 13667, 1325, 1230, 1188, 1171, 1130, 1057, 1032, 1008, 912,782, 761, 734, 701, 667, 646, 617.

^{1}H NMR (300 MHz, $CDCl_3$, 300 K): δ_H (ppm) = 8.18 – 8.15 (m, 2H), 7.54 – 7.51 (m, 2H), 7.40– 7.24 (m, 5H), 5.22 (d, J = 7.7 Hz, 1H), 4.15 – 4.03 (m, 1H), 2.54 – 2.40 (m, 2H), 1.06 – 1.03 (m, 6H), 0.88 (t, J = 7.3 Hz, 3H).

^{13}C NMR (75 MHz, $CDCl_3$, 300 K): δ_C (ppm) = 172.2 (C_q), 151.1 (C_q), 146.4 (C_q), 142.1 (C_q), 130.0 (CH), 128.8 (2 x CH), 127.6 (CH), 123.1 (CH), 61.0 (C_q), 41.9 (CH), 31.5 (CH_2), 22.4 (2 x CH_3), 10.0 (CH_3).

HRMS (ESI) *m/z* = 349.1523 calcd. for $[C_{19}H_{22}N_2O_3Na]^+$ $[M+Na]^+$, found: 349.1521.

2-(4-Cyanophenyl)-*N*-isopropyl-2-phenylbutanamide (3au):

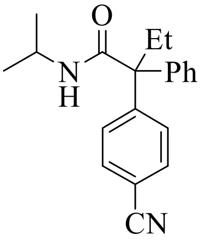

The title compound was prepared following the general procedure **GP3** using sulfonamide **1u** (44.9 mg, 0.200 mmol, 1.0 eq.), *n*-BuLi (1.6 M-solution in hexanes, 0.138 mL, 0.220 mmol, 1.1 eq.), ferrocenium tetrafluoroborate (54.7 mg, 0.200 mmol, 1.0 eq.) and ethylphenylketene **2a** (39 µL, 0.30 mmol, 1.5 eq.) in Et_2O (20 mL). Purification by RP-MPLC (MeOH/H_2O, gradient from 20% to 90%) gave the title compound as a colourless oil (24.1 mg, 78.7 µmol, 39%).

FT-IR (neat): ν (cm^{-1}) = 3419, 2972, 2937, 2879, 2230, 1657, 1606, 1503, 1455, 1386, 1331, 1241, 1202, 1169, 1129, 1093, 907, 727, 700, 647.

^{1}H NMR (300 MHz, $CDCl_3$, 300 K): δ_H (ppm) = 7.61 (d, J = 8.5 Hz, 2H), 7.45 (d, J = 8.5 Hz, 2H), 7.39 – 7.23 (m, 5H), 5.21 (d, J = 7.9 Hz, 1H), 4.14 – 4.02 (m, 1H), 2.51 – 2.37 (m, 2H), 1.05 – 1.02 (m, 6H), 0.86 (t, J = 7.3 Hz, 3H).

^{13}C NMR (75 MHz, $CDCl_3$, 300 K): δ_C (ppm) = 172.3, 149.0, 142.1, 131.8 (CH), 129.8 (CH), 128.8 (CH) , 128. (CH) 7, 127.5 (CH), 118.8 (C_q), 110.5 (C_q), 61.1 (C_q), 41.9 (CH), 31.3 (CH_2), 22.4 (2 x CH_3), 10.0 (CH_3).

HRMS (ESI) *m/z* = 329.1624 calcd. for $[C_{20}H_{22}N_2ONa]^+$ $[M+Na]^+$, found: 329.1624.

***N*-Isopropyl-4-(1-(isopropylamino)-1-oxo-2-phenylbutan-2-yl)benzamide (3av):**

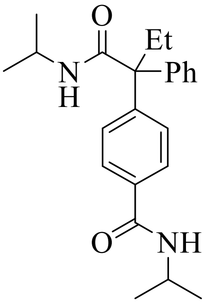

The title compound was prepared following the general procedure **GP3** using sulfonamide **1v** (56.9 mg, 0.200 mmol, 1.0 eq.), *n*-BuLi (1.6 M-solution in hexanes, 0.138 mL, 0.220 mmol, 1.1 eq.), ferrocenium tetrafluoroborate (54.7 mg, 0.200 mmol, 1.0 eq.) and ethylphenylketene **2a** (39 µL, 0.30 mmol, 1.5 eq.) in Et_2O (20 mL). Purification by RP-MPLC (MeOH/H_2O, gradient from 20% to 90%) gave the title compound as a colourless solid (38.8 mg, 0.106 mmol, 53%).

MP: 173-175 °C.

FT-IR (neat): ν (cm^{-1}) = 3320, 2972, 2938, 2880, 1728, 1712, 1693, 1656, 1639, 1611,1567, 1544, 1536, 1502, 1460, 1454, 1385, 1366, 1352, 1327, 1265, 1172, 1129, 1089, 850,811, 733, 701, 560.

^{1}H NMR (300 MHz, $CDCl_3$, 300 K): δ_H (ppm) = 7.72 – 7.69 (m, 2H), 7.40 – 7.25 (m, 7H), 5.95 (d, *J* = 8.0 Hz, 1H), 5.22 (d, *J* = 7.4 Hz, 1H), 4.33 – 4.24 (m, 1H), 4.14 – 4.02 (m, 1H),

2.45 (q, *J* = 7.7 Hz, 2H), 1.27 (d, *J* = 6.5 Hz, 6H), 1.02 (d, *J* = 6.6 Hz, 6H), 0.83 (t, *J* = 7.3 Hz, 3H)

^{13}C NMR (75 MHz, $CDCl_3$, 300 K): δ_C (ppm) = 172.9 (C_q), 166.4 (C_q), 146.8 (C_q), 142.8 (C_q), 133.3 (C_q), 129.2 (*C*H), 128.9 (*C*H), 128.4 (*C*H), 127.0 (*C*H), 126.7 (*C*H), 60.9 (C_q), 41.9 (*C*H), 41.7 (*C*H), 31.4 (CH_2), 22.9 (CH_3), 22.4 (CH_3), 10.0 (CH_3).

HRMS (ESI) *m/z* = 389.2200 calcd. for $[C_{23}H_{30}N_2O_2Na]^+$ $[M+Na]^+$, found: 389.2198.

***N*-Isopropyl-2-phenyl-2-(o-tolyl)butanamide (3aw):**

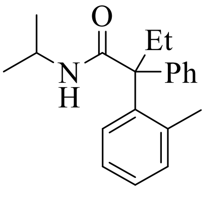

The title compound was prepared following the general procedure **GP3** using sulfonamide **1w** (42.7 mg, 0.200 mmol, 1.0 eq.), *n*-BuLi (1.6 M-solution in hexanes, 0.138 mL, 0.220 mmol, 1.1 eq.), ferrocenium tetrafluoroborate (54.7 mg, 0.200 mmol, 1.0 eq.) and ethylphenylketene **2a** (39 µL, 0.30 mmol, 1.5 eq.) in Et_2O (20 mL). Purification by RP-MPLC (MeOH/H_2O, gradient from 20% to 90%) gave the title compound as a yellowish oil (39.5 mg, 0.134 mmol, 67%).

FT-IR (neat): ν (cm^{-1}) = 3420, 2972, 2938, 2249, 1658, 1503, 1455, 1386, 1236, 1172, 1129, 904, 724, 648, 628.

^{1}H NMR (300 MHz, $CDCl_3$, 300 K): δ_H (ppm) = 7.44 – 7.39 (m, 3H), 7.24 – 7.04 (m, 6H), 5.04 (d, *J* = 8.0 Hz, 1H), 4.07 – 3.92 (m, 1H), 2.36 – 2.25 (m, 2H), 1.83 (s, 3H), 0.97 – 0.78 (m, 9H).

^{13}C NMR (75 MHz, $CDCl_3$, 300 K): δ_C (ppm) = 173.8 (C_q), 143.8 (C_q), 141.4 (C_q), 138.6 (C_q), 132.5 (*C*H), 128.6 (*C*H), 128.4 (*C*H), 127.8 (*C*H), 127.5 (*C*H), 126.1 (*C*H), 125.9 (*C*H), 59.2 (C_q), 41.3 (*C*H), 33.6 (CH_2), 22.5 (CH_3), 22.2 (CH_3), 21.4 (CH_3), 10.5. (CH_3)

HRMS (ESI) *m/z* = 318.1828 calcd. for $[C_{20}H_{25}NONa]^+$ $[M+Na]^+$, found: 318.1827.

N-Isopropyl-2-phenyl-2-(m-tolyl)butanamide (3ax):

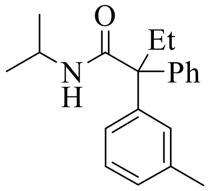

The title compound was prepared following the general procedure **GP3** using sulfonamide **1x** (42.7 mg, 0.200 mmol, 1.0 eq.), *n*-BuLi (1.6 M-solution in hexanes, 0.138 mL, 0.220 mmol, 1.1 eq.), ferrocenium tetrafluoroborate (54.7 mg, 0.200 mmol, 1.0 eq.) and ethylphenylketene **2a** (39 µL, 0.30 mmol, 1.5 eq.) in Et_2O (20 mL). Purification by RP-MPLC (MeOH/H_2O, gradient from 20% to 90%) gave the title compound as a colourless oil (44.9 mg, 0.152 mmol ,76%).

FT-IR (neat): ν (cm^{-1}) = 3423, 2972, 2936, 2879, 2448, 1658, 1603, 1456, 1386, 1324, 1171, 1128, 905, 768, 724, 647.

^{1}H NMR (300 MHz, $CDCl_3$, 300 K): δ_H (ppm) = 7.26 – 7.15 (m, 6H), 7.09 – 7.03 (m, 3H), 5.23 (d, *J* = 7.9 Hz, 1H), 4.12 – 3.96 (m, 1H), 2.39 (q, *J* = 7.3 Hz, 2H), 2.29 (s, 3H), 0.97 (d, *J* = 6.2 Hz, 6H), 0.79 (t, *J* = 7.3 Hz, 3H).

^{13}C NMR (75 MHz, $CDCl_3$, 300 K): δ_C (ppm) = 173.7 (C_q), 143.4 (C_q), 143.2 (C_q), 137.7 (C_q), 129.6 (*C*H), 129.0 (*C*H), 128.2 (*C*H), 128.0 (*C*H), 127.5 (*C*H), 126.7 (*C*H), 126.2 (*C*H), 60.9 (C_q), 41.6 (*C*H), 31.4 (CH_2), 22.4 (CH_3), 21.7 (CH_3), 10.2 (CH_3).

HRMS (ESI) *m/z* = 318.1828 calcd. for $[C_{20}H_{25}NONa]^+$ $[M+Na]^+$, found: 318.1826.

2-(3,5-Bis(trifluoromethyl)phenyl)-N-isopropyl-2-phenylbutanamide (3ay):

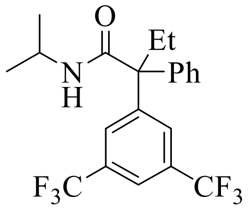

The title compound was prepared following the general procedure **GP3** using sulfonamide **1y** (67.1 mg, 0.200 mmol, 1.0 eq.), *n*-BuLi (1.6 M-solution in hexanes, 0.138 mL, 0.220 mmol, 1.1 eq.), ferrocenium tetrafluoroborate (54.7 mg, 0.200 mmol, 1.0 eq.) and ethylphenylketene **2**[a] (39 µL, 0.30 mmol, 1.5 eq.) in Et_2O (20 mL). Purification by RP-MPLC (MeOH/H_2O, gradient from 20% to 90%) gave the title compound as a yellowish oil (41.1 mg, 98.5 µmol, 49%).

FT-IR (neat): ν (cm^{-1}) = 3420, 2976, 2938, 2880, 1815, 1661, 1459, 1370, 1227, 1172, 1137, 1033, 906, 727, 692, 649.

^{1}H NMR (600 MHz, $CDCl_3$, 300 K): δ_H (ppm) = 7.82 (s, 2H), 7.78 (s, 1H), 7.41 – 7.38 (m, 2H), 7.35 – 7.33 (m, 1H), 7.26 – 7.24 (m, 2H), 5.22 (d, *J* = 7.9 Hz, 1H), 4.13 – 4.07 (m, 1H), 2.54 – 2.40 (m, 2H), 1.07 – 1.01 (m, 6H), 0.88 (t, *J* = 7.3 Hz, 3H).

$^{13}C\{^1H,^{19}F\}$ NMR (151 MHz, $CDCl_3$, 300 K): δ_C (ppm) = 171.9 (C_q), 146.1 (C_q), 141.7 (C_q), 131.1 (C_q), 129.3 (*C*H), 128.9 (*C*H), 128.6 (*C*H), 127.8 (*C*H), 123.3 (C_q), 120.6 (*C*H), 60.7 (C_q), 41.9 (*C*H), 31.4 (CH_2), 22.3 (CH_3), 22.2 (CH_3), 9.8 (CH_3).

$^{19}F\{^1H,^{13}C\}$ NMR (564 MHz, $CDCl_3$, 300 K): δ_F (ppm) = −62.8.

HRMS (ESI) *m/z* = 440.1420 calcd. for $[C_{21}H_{21}NOF_6Na]^+$ $[M+Na]^+$, found: 440.1415.

***N*-Isopropyl-2-(naphthalen-1-yl)-2-phenylbutanamide (3az):**

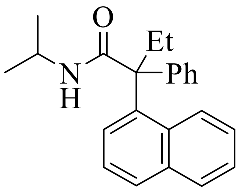

The title compound was prepared following the general procedure **GP3** using sulfonamide **1z** (49.9 mg, 0.200 mmol, 1.0 eq.), *n*-BuLi (1.6 M-solution in hexanes, 0.138 mL, 0.220 mmol, 1.1 eq.), ferrocenium tetrafluoroborate (54.7 mg, 0.200 mmol, 1.0 eq.) and ethylphenylketene **2a** (39 µL, 0.30 mmol, 1.5 eq.) in Et_2O (20 mL). Purification by RP-MPLC (MeOH/H_2O, gradient from 20% to 90%) gave the title compound as a colourless oil (12.6 mg, 38.0 µmol, 19%).

FT-IR (neat): ν (cm^{-1}) = 3420, 3053, 2971, 2933, 2877, 1732, 1658, 1598, 1492, 1455, 1365, 1337, 11331, 1228, 1187, 1171, 1128, 1076, 1032, 910, 782, 762, 728, 700, 646, 570.

^1H NMR (300 MHz, $CDCl_3$, 300 K): δ_H (ppm) = 7.87 – 7.82 (m, 2H), 7.68 – 7.66 (m, 2H), 7.55 – 7.48 (m, 3H), 7.41– 7.36 (m, 1H), 7.25 – 7.15 (m, 4H), 5.01 (d, *J* = 8.2 Hz, 1H), 4.08 – 3.97 (m, 1H), 2.63 – 2.55 (m, 2H), 0.96 (d, *J* = 6.6 Hz, 3H), 0.86 (t, *J* = 7.3 Hz, 3H), 0.69 (d, *J* = 6.6 Hz, 3H).

^{13}C NMR (75 MHz, $CDCl_3$, 300 K): δ_C (ppm) = 174.2 (C_q), 143.8 (C_q), 138.8 (C_q), 134.6 (C_q), 132.0 (C_q), 129.0 (*C*H), 128.9 (*C*H), 128.8 (*C*H), 127.8 (*C*H), 126.9 (*C*H), 126.8 (*C*H), 126.3 (*C*H), 125.6 (*C*H), 125.5 (*C*H), 124.9 (*C*H), 59.4 (C_q), 41.4 (*C*H), 33.6 (CH_2), 22.4 (CH_3), 21.9 (CH_3), 10.4 (CH_3).

HRMS (ESI) *m/z* = 354.1828 calcd. for $[C_{23}H_{25}NONa]^+$ $[M+Na]^+$, found: 354.1827.

***N*-Isopropyl-2-(naphthalen-2-yl)-2-phenylbutanamide (3aaa):**

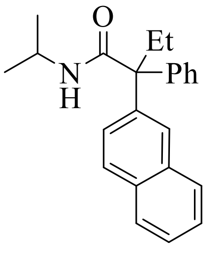

The title compound was prepared following the general procedure **GP3** using sulfonamide **1aa** (49.9 mg, 0.200 mmol, 1.0 eq.), *n*-BuLi (1.6 M-solution in hexanes, 0.138 mL, 0.220 mmol, 1.1 eq.), ferrocenium tetrafluoroborate (54.7 mg, 0.200 mmol, 1.0 eq.) and ethylphenylketene **2a** (39 µL, 0.30 mmol, 1.5 eq.) in Et_2O (20 mL). Purification by RP-MPLC (MeOH/H_2O, gradient from 20% to 90%) gave the title compound as a colourless oil (48.6 mg, 0.147 mmol, 73%).

FT-IR (neat): ν (cm^{-1}) = 3423, 3056, 2971, 2935, 2880, 1726, 1712, 1680, 1599, 1504, 1454, 1386, 1367, 1326, 1234, 1173, 1131, 904, 857, 819, 728, 649.

^{1}H NMR (300 MHz, $CDCl_3$, 300 K): δ_H (ppm) = 7.84 – 7.78 (m, 4H), 7.52 – 7.47 (m, 2H), 7.39 – 7.27 (m, 6H), 5.31 (d, *J* = 7.9 Hz, 1H), 4.19 – 4.07 (m, 1H), 2.56 (q, *J* = 7.3 Hz, 2H), 1.08 – 0.98 (m, 6H), 0.90 (t, *J* = 7.3 Hz, 3H).

^{13}C NMR (75 MHz, $CDCl_3$, 300 K): δ_C (ppm) = 173.4 (C_q), 143.3 (C_q), 140.6 (C_q), 133.1 (C_q), 132.1 (*C*H), 129.1 (*C*H), 128.3 (*C*H), 128.2 (*C*H), 127.7 (*C*H), 127.6 (*C*H), 127.4 (*C*H), 126.8 (*C*H), 126.2 (2 x *C*H), 61.0 (C_q), 41.7 (*C*H), 31.5 (CH_2), 22.5 (CH_3), 22.4 (CH_3), 10.3 (CH_3).

HRMS (ESI) *m/z* = 354.1828 calcd. for $[C_{23}H_{25}NONa]^+$ $[M+Na]^+$, found: 354.1828.

***N*-Isopropyl-2-phenyl-2-(thiophen-2-yl)butanamide (3aab):**

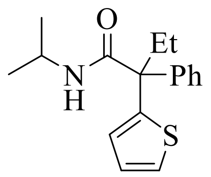

The title compound was prepared following the general procedure **GP3** using sulfonamide **1ab** (41.1 mg, 0.200 mmol, 1.0 eq.), *n*-BuLi (1.6 M-solution in hexanes, 0.138 mL, 0.220 mmol, 1.1 eq.), ferrocenium tetrafluoroborate (54.7 mg, 0.200 mmol, 1.0 eq.) and ethylphenylketene **2a** (39 µL, 0.30 mmol, 1.5 eq.) in Et_2O (20 mL). Purification by RP-MPLC (MeOH/H_2O, gradient from 20% to 90%) gave the title compound as a colourless oil (15.3 mg, 53.2 µmol, 27%).

FT-IR (neat): ν (cm^{-1}) = 3420, 2971, 2937, 2879, 1661, 1600, 1509, 1455, 1386, 1368, 1278, 1234, 1171, 1133, 1086, 1033,841, 809, 767, 697, 578.

^{1}H NMR (300 MHz, $CDCl_3$, 300 K): δ_H (ppm) = 7.36 – 7.25 (m, 6H), 6.95 (dd, *J* = 5.1, 3.6 Hz, 1H), 6.82 (dd, *J* = 3.6, 1.2 Hz, 1H), 5.36 (bs, 1H), 4.15 – 4.08 (m, 1H), 2.49 – 2.38 (m, 2H), 1.08 (d, *J* = 6.6 Hz, 3H), 1.03 (d, *J* = 6.6 Hz, 3H), 0.94 (t, *J* = 7.3 Hz, 3H).

^{13}C NMR (75 MHz, $CDCl_3$, 300 K): δ_C (ppm) = 172.7 (C_q), 148.1 (C_q), 143.1 (C_q), 128.4 (*C*H), 128.3 (*C*H), 127.3 (*C*H), 126.9 (*C*H), 126.1 (*C*H), 125.2 (*C*H), 58.9 (C_q), 41.8 (*C*H), 33.5 (CH_2), 22.5 (CH_3), 22.4 (CH_3), 10.1 (CH_3).

HRMS (ESI) *m/z* = 310.1236 calcd. for $[C_{17}H_{21}NOSNa]^+$ $[M+Na]^+$, found: 310.1234.

2-(Benzo[b]thiophen-2-yl)-*N*-isopropyl-2-phenylbutanamide (3aac):

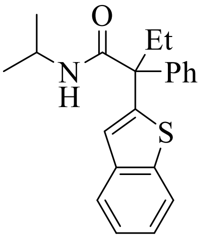

The title compound was prepared following the general procedure **GP3** using sulfonamide **1ac** (51.1 mg, 0.200 mmol, 1.0 eq.), *n*-BuLi (1.6 M-solution in hexanes, 0.138 mL, 0.220 mmol, 1.1 eq.), ferrocenium tetrafluoroborate (54.7 mg, 0.200 mmol, 1.0 eq.) and ethylphenylketene **2a** (39 µL, 0.30 mmol, 1.5 eq.) in Et_2O (20 mL). Purification by RP-MPLC (MeOH/H_2O, gradient from 5% to 70%) gave the title compound as a colourless oil (29.3 mg, 86.8 µmol, 43%).

FT-IR (neat): ν (cm^{-1}) = 3417, 3058, 2971, 2934, 2877, 2359, 2246, 1659, 1599, 1504, 1456, 1433, 1385, 1367, 1307, 1236, 1171, 1130, 1072, 1033, 1014, 907, 859, 829, 725, 699, 646, 564.

^{1}H NMR (300 MHz, $CDCl_3$, 300 K): δ_H (ppm) = 7.79 – 7.76 (m, 1H), 7.70 – 7.66 (m, 1H), 7.45 – 7.29 (m, 7H), 7.13 (s, 1H), 5.46 (d, *J* = 8.0 Hz, 1H), 4.20 – 4.09 (m, 1H), 2.59 – 2.45 (m, 2H), 1.11 – 0.98 (m, 9H).

^{13}C NMR (75 MHz, $CDCl_3$, 300 K): δ_C (ppm) = 172.1 (C_q), 149.2 (C_q), 142.6 (C_q), 139.1 (C_q), 128.6 (*C*H), 128.4 (*C*H), 127.5 (*C*H), 124.2 (*C*H), 123.8 (*C*H), 123.4 (*C*H), 122.0 (*C*H), 59.4 (C_q), 41.9 (*C*H), 33.3 (CH_2), 22.5 (CH_3), 22.4 (CH_3), 10.2 (CH_3).[11]

HRMS (ESI) *m/z* = 360.1393 calcd. for $[C_{21}H_{23}NOSNa]^+$ $[M+Na]^+$, found: 360.1388.

2-(Benzofuran-2-yl)-*N*-isopropyl-2-phenylbutanamide (3aad):

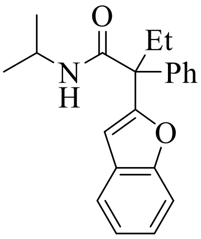

The title compound was prepared following the general procedure **GP3** using sulfonamide **1ad** (47.9 mg, 0.200 mmol, 1.0 eq.), *n*-BuLi (1.6 M-solution in hexanes, 0.138 mL, 0.220 mmol, 1.1 eq.), ferrocenium tetrafluoroborate (54.7 mg, 0.200 mmol, 1.0 eq.) and ethylphenylketene **2a** (39 µL, 0.30 mmol, 1.5 eq.) in Et_2O (20 mL). Purification by RP-MPLC (MeOH/H_2O, gradient from 5% to 70%) gave the title compound as a colourless oil (12.9 mg, 40.1 µmol, 20%).

FT-IR (neat): ν (cm^{-1}) = 3427, 2974, 2359, 1668, 1514, 1454, 1276, 1173, 913, 764, 749, 702, 651, 586.

^{1}H NMR (300 MHz, $CDCl_3$, 300 K): δ_H (ppm) = 7.59 – 7.56 (m, 1H), 7.44 – 7.41 (m, 1H), 7.35 – 7.22 (m, 7H), 6.77 (d, *J* = 0.7 Hz, 1H), 5.76 (d, *J* = 7.9 Hz, 1H), 4.17 – 4.06 (m, 1H), 2.54 – 2.37 (m, 2H), 1.12 (d, *J* = 6.5 Hz, 3H), 1.04 (d, *J* = 6.6 Hz, 3H), 0.91 (t, *J* = 7.4 Hz, 3H).

[11] A signal of a quaternary carbon atom and one of an aromatic C−H unit are not resolved.

^{13}C NMR (126 MHz, $CDCl_3$, 300 K): δ_C (ppm) = 170.8 (C_q), 158.4 (C_q), 154.7 (C_q), 141.0 (C_q), 128.3 (*C*H), 128.1 (*C*H and C_q), 127.2 (*C*H), 124.2 (*C*H), 122.9 (*C*H), 121.0 (*C*H), 111.0 (*C*H), 106.4 (*C*H), 58.2 (C_q), 41.9 (*C*H), 30.3 (CH_2), 22.4 (2 x CH_3), 9.8 (CH_3).

HRMS (ESI) *m/z* = x344.1621 calcd. for $[C_{21}H_{21}NO_2Na]^+$ $[M+Na]^+$, found: 344.1613.

***N*-Isopropyl-2,2-diphenylpropanamide (3ba):**

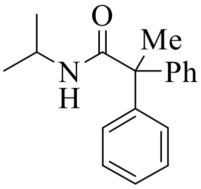

The title compound was prepared following the general procedure **GP3** using sulfonamide **1a** (39.9 mg, 0.200 mmol, 1.0 eq.), *n*-BuLi (1.6 M-solution in hexanes, 0.138 mL, 0.220 mmol, 1.1 eq.), ferrocenium tetrafluoroborate (54.6 mg, 0.200 mmol, 1.0 eq.) and methylphenylketene **2b** (38 µL, 0.30 mmol, 1.5 eq.) in Et_2O (20 mL). Purification by RP-MPLC (MeOH/H_2O, gradient from 20% to 90%) gave the title compound as a colourless oil (33.5 mg, 0.125 mmol, 63%).

FT-IR (neat): ν (cm^{-1}) = 3420. 3086, 3023, 2972, 2935, 1732, 1662, 1600, 1510, 1456, 1386, 1368, 1241, 1222, 1190, 1170, 1130, 1068, 1029, 964, 901, 850, 817, 773, 724, 699, 665, 635, 560.

^{1}H NMR (300 MHz, $CDCl_3$, 300 K): δ_H (ppm) = 7.00 – 6.86 (m, 10H), 4.86 (d, *J* = 6.7 Hz, 1H), 3.84 – 3.69 (m, 1H), 1.63 (s, 3H), 0.70 (d, *J* = 6.6 Hz, 6H).

^{13}C NMR (75 MHz, $CDCl_3$, 300 K): δ_C (ppm) = 174.1 (C_q), 145.1 (C_q), 128.5 (*C*H), 128.1 (*C*H), 126.9 (*C*H), 56.8 (C_q), 41.8 (*C*H), 27.1 (CH_2), 22.5 (CH_3).

HRMS (ESI) *m/z* = 290.1515 calcd. for $[C_{18}H_{21}NONa]^+$ $[M+Na]^+$, found: 290.1516.

***N*-Isopropyl-3-methyl-2,2-diphenylbutanamide (3ca):**

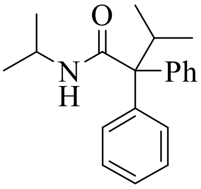

The title compound was prepared following the general procedure **GP3** using sulfonamide **1a** (39.9 mg, 0.200 mmol, 1.0 eq.), *n*-BuLi (1.6 M-solution in hexanes, 0.138 mL, 0.220 mmol, 1.1 eq.), ferrocenium tetrafluoroborate (54.6 mg, 0.200 mmol, 1.0 eq.) and isopropylphenylketene **2c** (46 µL, 0.30 mmol, 1.5 eq.) in Et_2O (20 mL). Purification by RP-MPLC (MeOH/H_2O, gradient from 20% to 90%) gave the title compound as a colourless oil (17.3 mg, 58.6 µmol, 29%).

FT-IR (neat): ν (cm^{-1}) = 3431, 2972, 3878, 1658, 1600, 1504, 1386, 1366, 1235, 1167, 1129, 1085, 1035, 906, 770, 725, 647, 577.

^{1}H NMR (300 MHz, $CDCl_3$, 300 K): δ_H (ppm) = 7.33 – 7.15 (m, 10H), 5.21 (d, J = 8.4 Hz, 1H), 4.05 – 3.84 (m, 1H), 3.29 (hept, J = 6.6 Hz, 1H), 0.87 (d, J = 6.5 Hz, 6H), 0.73 (d, J = 6.7 Hz, 6H).

^{13}C NMR (75 MHz, $CDCl_3$, 300 K): δ_C (ppm) = 172.8 (C_q), 141.3 (C_q), 130.2 (CH), 127.7 (CH), 126.6 (CH), 65.6 (C_q), 41.6 (CH), 31.2 (CH$_2$), 22.3 (CH$_3$), 19.1 (CH$_3$).

HRMS (ESI) *m/z* = 318.1828 calcd. for $[C_{20}H_{25}NONa]^+$ $[M+Na]^+$, found: 318.1828.

2-Cyclopentyl-*N*-isopropyl-2,2-diphenylacetamide (3da):

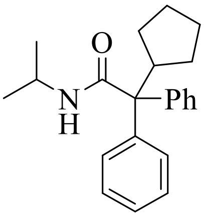

The title compound was prepared following the general procedure **GP3** using sulfonamide **1a** (39.9 mg, 0.200 mmol, 1.0 eq.), *n*-BuLi (1.6 M-solution in hexanes, 0.138 mL, 0.220 mmol, 1.1 eq.), ferrocenium tetrafluoroborate (54.6 mg, 0.200 mmol, 1.0 eq.) and cyclopentylphenylketene **2d** (~1:1 mixture with the corresponding acid chloride[12], 104 µL, ~0.300 mmol, 1.5 eq.) in Et_2O (20 mL). Purification by RP-MPLC (MeOH/H_2O, gradient from 20% to 90%) gave the title compound as a colourless oil (35.8 mg, 0.111 mmol, 56%).

FT-IR (neat): ν (cm^{-1}) = 3429, 3059, 2964, 2873, 1657, 1599, 1494, 1447, 1386, 1367, 1233, 1170, 1129, 1085, 1036, 906, 726, 700, 647, 601, 578.

^{1}H NMR (300 MHz, $CDCl_3$, 300 K): δ_H (ppm) = 7.41 – 7.13 (m, 10H), 5.21 (d, J = 8.0 Hz, 1H), 4.16 – 3.98 (m, 1H), 3.31 (p, J = 8.1 Hz, 1H), 1.92 – 1.77 (m, 2H), 1.61 – 1.25 (m, 6H), 1.00 (d, J = 6.5 Hz, 6H).

^{13}C NMR (75 MHz, $CDCl_3$, 300 K): δ_C (ppm) = 173.1 (C_q), 143.4 (C_q), 129.9 (CH), 127.8 (CH), 126.6 (CH), 63.9 (C_q), 45.7 (CH), 41.5 (CH), 29.5 (CH$_2$), 25.3 (CH$_2$), 22.4 (CH$_3$).

HRMS (ESI) *m/z* = 344.1985 calcd. for $[C_{22}H_{27}NONa]^+$ $[M+Na]^+$, found: 344.1985.

***N*-Isopropyl-1-phenyl-1,2,3,4-tetrahydronaphthalene-1-carboxamide (3ea):**

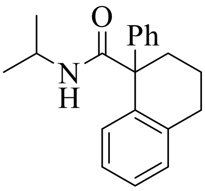

The title compound was prepared following the general procedure **GP3** using sulfonamide **1a** (39.9 mg, 0.200 mmol, 1.0 eq.), *n*-BuLi (1.6 M-solution in hexanes, 0.138 mL, 0.220 mmol, 1.1 eq.), ferrocenium tetrafluoroborate (54.6 mg, 0.200 mmol, 1.0 eq.) and ketene **2e** (52 µL, 0.30 mmol, 1.5 eq.) in

[12] Determined by ^{1}H NMR spectroscopy.

Et_2O (20 mL). Purification by RP-MPLC (MeOH/H_2O, gradient from 20% to 90%) gave the title compound as a colourless oil (24.4 mg, 83.2 µmol, 42%).

FT-IR (neat): ν (cm^{-1}) = 3423, 2971, 2941, 2874, 2245, 1726, 1712, 1692, 1658, 1599, 1552, 1527, 1514, 1494, 1461, 1445, 1366, 1367, 1326, 1237, 1169, 1129, 1035, 909, 754, 730, 699, 647.

^{1}H NMR (300 MHz, $CDCl_3$, 300 K): δ_H (ppm) = 7.32 – 7.17 (m, 7H), 7.11 – 7.06 (m, 1H), 6.88 – 6.86 (m, 1H), 5.44 (d, *J* = 7.3 Hz, 1H), 4.22 – 4.10 (m, 1H), 2.93 – 2.81 (m, 3H), 2.16 – 2.10 (m, 1H), 1.78 – 1.72 (m, 2H), 1.10 (d, *J* = 6.5 Hz, 3H), 1.03 (d, *J* = 6.5 Hz, 3H).

^{13}C NMR (75 MHz, $CDCl_3$, 300 K): δ_C (ppm) = 173.5 (C_q), 145.6 (C_q), 139.1 (C_q), 138.1 (C_q), 129.9 (*C*H), 128.6 (*C*H), 128.2 (*C*H), 127.2 (*C*H), 126.5 (*C*H), 125.9 (*C*H), 58.0 (C_q), 41.8 (*C*H), 36.2 (CH_2), 29.5 (CH_2), 22.6 (2 x CH_3), 19.9 (CH_2).

HRMS (ESI) *m/z* = 316.1672 calcd. for $[C_{20}H_{23}NONa]^+$ $[M+Na]^+$, found: 316.1672.

2-(4-Bromophenyl)-*N*-isopropyl-2-phenylbutanamide (3fa):

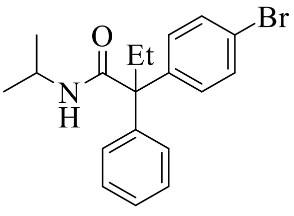

The title compound was prepared following the general procedure **GP3** using sulfonamide **1a** (39.9 mg, 0.200 mmol, 1.0 eq.), *n*-BuLi (1.6 M-solution in hexanes, 0.138 mL, 0.220 mmol, 1.1 eq.), ferrocenium tetrafluoroborate (54.6 mg, 0.200 mmol, 1.0 eq.) and ketene **2f** (43 µL, 0.30 mmol, 1.5 eq.) in Et_2O (20 mL). Purification by RP-MPLC (MeOH/H_2O, gradient from 20% to 90%) gave the title compound as a colourless oil (32.9 mg, 91.3 µmol, 46%).

FT-IR (neat): ν (cm^{-1}) = 3421, 2971, 2937, 2879, 1657, 1488, 1455, 1386, 1367 1235, 1168, 1129, 1077, 1033, 1009, 905, 824, 727, 702, 648, 620, 577.

^{1}H NMR (300 MHz, $CDCl_3$, 300 K): δ_H (ppm) = 7.46 – 7.18 (m, 9H), 5.21 (d, *J* = 7.7 Hz, 1H), 4.13 – 4.02 (m, 1H), 2.41 (q, *J* = 7.3 Hz, 2H), 1.02 (d, *J* = 6.5 Hz, 6H), 0.83 (t, *J* = 7.3 Hz, 3H).

^{13}C NMR (75 MHz, $CDCl_3$, 300 K): δ_C (ppm) = 142.8 (C_q), 142.4 (C_q), 131.2 (*C*H), 130.8 (*C*H), 128.9 (*C*H), 128.4 (*C*H), 127.1 (*C*H), 120.8 (C_q), 60.6 (C_q), 41.7 (*C*H), 31.4 (CH_2), 22.4 (CH_3), 10.0 (CH_3).[13]

HRMS (ESI) *m/z* = 382.0777 calcd. for [C19H22NOBrNa]$^+$ $[M+Na]^+$, found: 382.0777.

2-(4-Iodophenyl)-*N*-isopropyl-2-phenylbutanamide (3ga):

[13] The signal of the carbonyl carbon atom is not resolved.

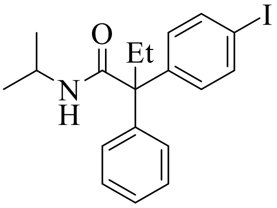

The title compound was prepared following the general procedure **GP3** using sulfonamide **1a** (39.9 mg, 0.200 mmol, 1.0 eq.), *n*-BuLi (1.6 M-solution in hexanes, 0.138 mL, 0.220 mmol, 1.1 eq.), ferrocenium tetrafluoroborate (54.6 mg, 0.200 mmol, 1.0 eq.) and ketene **2g** (49 µL, 0.30 mmol, 1.5 eq.) in Et_2O (20 mL). Purification by RP-MPLC ($MeOH/H_2O$, gradient from 20% to 90%) gave the title compound as a colourless oil (52.4 mg, 0.129 mmol, 65%).

FT-IR (neat): ν (cm^{-1}) = 3421, 2971, 2937, 2879, 1728, 1658, 1504, 1485, 1455, 1389, 1236, 1168, 1129, 1085, 1032, 1005, 905, 820, 725, 647, 599.

^{1}H NMR (300 MHz, $CDCl_3$, 300 K): δ_H (ppm) = 7.64 – 7.61 (m, 2H), 7.34 – 7.24 (m, 5H), 7.06 – 7.03 (m, 2H), 5.20 (d, *J* = 7.9 Hz, 1H), 4.11 – 3.99 (m, 1H), 2.42 – 2.35 (m, 2H), 1.03 – 0.99 (m, 6H), 0.81 (t, *J* = 7.3 Hz, 3H).

^{13}C NMR (75 MHz, $CDCl_3$, 300 K): δ_C (ppm) = 172.9 (C_q), 143.1 (C_q), 142.7 (C_q), 137.2 (*C*H), 131.1 (*C*H), 128.9 (*C*H), 128.4 (*C*H), 127.1 (*C*H), 92.4 (C_q), 60.7 (C_q), 41.7 (*C*H), 31.3 (CH_2), 22.4 (CH_3), 10.0 (CH_3).

HRMS (ESI) *m/z* = 430.0638 calcd. for $[C19H22NOINa]^+$ $[M+Na]^+$, found: 430.0634.

2-(4-Isobutylphenyl)-*N*-isopropyl-2-phenylpropanamide (3ha):

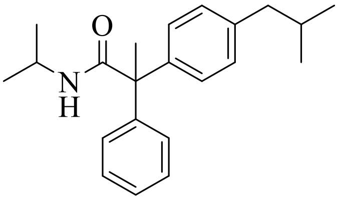

The title compound was prepared following the general procedure **GP3** using sulfonamide **1a** (39.9 mg, 0.200 mmol, 1.0 eq.), *n*-BuLi (1.6 M-solution in hexanes, 0.138 mL, 0.220 mmol, 1.1 eq.), ferrocenium tetrafluoroborate (54.6 mg, 0.200 mmol, 1.0 eq.) and ketene **2h** (52 µL, 0.30 mmol, 1.5 eq.) in Et_2O (20 mL). Purification by RP-MPLC ($MeOH/H_2O$, gradient from 20% to 90%) gave the title compound as a colourless oil (48.2 mg, 0.149 mmol, 75%).

^{1}H NMR (300 MHz, $CDCl_3$, 300 K): δ_H (ppm) = 7.35 – 7.21 (m, 5H), 7.14 – 7.07 (m, 4H), 5.22 (d, *J* = 7.8 Hz, 1H), 4.17 – 4.05 (m, 1H), 2.46 (d, *J* = 7.2 Hz, 2H), 1.97 (s, 3H), 1.86 (hept, *J* = 6.8 Hz, 1H), 1.06 – 1.03 (m, 6H), 0.90 (d, *J* = 6.8 Hz, 6H).

^{13}C NMR (75 MHz, $CDCl_3$, 300 K): δ_C (ppm) = 174.4 (C_q), 145.3 (C_q), 142.3 (C_q), 140.3 (C_q), 129.2 (*C*H), 128.4 (*C*H), 128.1 (*C*H), 127.8 (*C*H), 126.8 (*C*H), 56.5 (C_q), 44.9 (CH_2), 41.7 (*C*H), 30.2 (*C*H), 27.0 (CH_3), 22.5 (2 x CH_3), 22.4 (CH_3).

HRMS (ESI) *m/z* = 346.2141 calcd. for $[C_{22}H_{29}NONa]^+$ $[M+Na]^+$, found: 346.2143.

The analytical data are consistent with those reported in the literature.[34]

***N*-Isopropyl-2-methyl-2,3-diphenylpropanamide (3ja):**

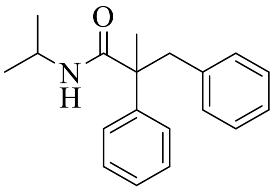

The title compound was prepared following the general procedure **GP3** using sulfonamide **1a** (39.9 mg, 0.200 mmol, 1.0 eq.), *n*-BuLi (1.6 M-solution in hexanes, 0.138 mL, 0.220 mmol, 1.1 eq.), ferrocenium tetrafluoroborate (54.6 mg, 0.200 mmol, 1.0 eq.) and ketene **2j** (38 µL, 0.30 mmol, 1.5 eq.) in Et_2O (20 mL). Purification by RP-MPLC ($MeOH/H_2O$, gradient from 20% to 90%) gave the title compound as a colourless solid (27.4 mg, 97.5 µmol, 49%).

MP: 95-97 °C.

FT-IR (neat): ν (cm^{-1}) = 3347, 3060, 3028, 2972, 2933, 2877, 1657, 1643, 1602, 1547, 1527, 1514, 1498, 1461, 1385, 1367, 1322, 1264, 1172, 1131, 1077, 1031, 967, 849, 746, 699.

^{1}H NMR (600 MHz, $CDCl_3$, 300 K): δ_H (ppm) = 7.34 – 7.23 (m, 5H), 7.14 – 7.09 (m, 3H), 6.79 – 6.77 (m, 2H), 5.01 (d, *J* = 7.7 Hz, 1H), 4.12 – 4.05 (m, 1H), 3.43 (d, *J* = 13.4 Hz, 1H), 3.22 (d, *J* = 13.4 Hz, 1H), 1.44 (s, 3H), 1.05 – 1.03 (m, 6H).

^{13}C NMR (151 MHz, $CDCl_3$, 300 K): δ_C (ppm) = 175.9 (C_q), 143.4 (C_q), 137.7 (C_q), 130.6 (*C*H), 128.3 (*C*H), 127.5 (2 x *C*H), 127.0 (*C*H), 126.1 (*C*H), 51.4 (C_q), 45.2 (*C*H), 41.5 (CH_2), 23.1 (CH_3), 22.5 (CH_3), 22.4 (CH_3).

HRMS (ESI) *m/z* = 304.1672 calcd. for $[C_{19}H_{23}NONa]^+$ $[M+Na]^+$, found: 304.1670.

2-Cyclohexyl-*N*-isopropyl-2-phenylpropanamide (3ka):

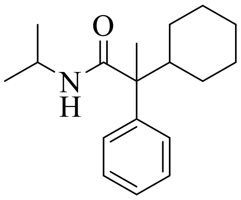

The title compound was prepared following the general procedure **GP3** using sulfonamide **1a** (39.9 mg, 0.200 mmol, 1.0 eq.), *n*-BuLi (1.6 M-solution in hexanes, 0.138 mL, 0.220 mmol, 1.1 eq.), ferrocenium tetrafluoroborate (54.6 mg, 0.200 mmol, 1.0 eq.) and ketene **2k** (38 µL, 0.30 mmol, 1.5 eq.) in Et_2O (20 mL). Purification by RP-MPLC ($MeOH/H_2O$, gradient from 20% to 90%) gave the title compound as a colourless solid (40.1 mg, 0.147 mmol, 74%).

MP: 125-126 °C.

FT-IR (neat): ν (cm^{-1}) = 3356, 3058, 2971, 2928, 2853, 1635, 1599, 1522, 1498, 1445, 1386, 1366, 1257, 1244, 1172, 1131, 1083, 1030, 1002, 963, 929, 898, 848, 777, 762, 736, 699, 671, 601, 576.

^{1}H NMR (300 MHz, $CDCl_3$, 300 K): δ_H (ppm) = 7.27 – 7.08 (m, 5H), 5.00 (d, J = 7.7 Hz, 1H), 3.99 – 3.83 (m, 1H), 2.16 – 2.08 (m, 1H), 1.65 – 1.55 (m, 4H), 1.36 (s, 2H), 1.26 – 0.72 (m, 12H).

^{13}C NMR (75 MHz, $CDCl_3$, 300 K): δ_C (ppm) = 175.0 (C_q), 143.4 (C_q), 128.2 (*C*H), 127.0 (*C*H), 126.5 (*C*H), 53.4 (C_q), 44.9 (*C*H), 41.3 (*C*H), 29.1 (CH_2), 28.1 (CH_2), 27.1 (CH_2), 27.0 (CH_2), 26.7 (CH_2), 22.6 (CH_3), 22.4 (CH_3), 18.9 (CH_3).

HRMS (ESI) *m/z* = 296.1985 calcd. for $[C_{18}H_{27}NONa]^+$ $[M+Na]^+$, found: 296.1983.

***N*-Isopropyl-1-phenylcycloheptane-1-carboxamide (3la):**

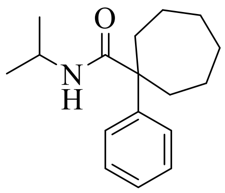

The title compound was prepared following the general procedure **GP3** using sulfonamide **1a** (39.9 mg, 0.200 mmol, 1.0 eq.), *n*-BuLi (1.6 M-solution in hexanes, 0.138 mL, 0.220 mmol, 1.1 eq.), ferrocenium tetrafluoroborate (54.6 mg, 0.200 mmol, 1.0 eq.) and ketene **2l** (34 µL, 0.30 mmol, 1.5 eq.) in Et_2O (20 mL). Purification by RP-MPLC (MeOH/H_2O, gradient from 20% to 90%) gave the title compound as a colourless oil (42.0 mg, 0.162 mmol, 81%).

FT-IR (neat): ν (cm^{-1}) = 3425, 3343, 3204, 2968, 2925, 2859, 1999, 1641, 1600, 1510, 1420, 1385, 1365, 1324, 1250, 1197, 1170, 1130, 1081, 1036, 952, 913, 846, 812, 760, 724, 698, 644.

^{1}H NMR (300 MHz, $CDCl_3$, 300 K): δ_H (ppm) = 7.32 – 7.16 (m, 5H), 4.67 (d, J = 7.6 Hz, 1H), 4.68 – 3.89 (m, 1H), 2.34 – 2.26 (m, 2H), 2.03 – 1.95 (m, 2H), 1.80 – 1.40 (m, 8H), 0.92 (d, J = 6.5 Hz, 6H).

^{13}C NMR (75 MHz, $CDCl_3$, 300 K): δ_C (ppm) = 176.8 (C_q), 145.8 176.8 (C_q), 128.6 (*C*H), 126.8 (*C*H), 126.6 (*C*H), 54.2 (C_q), 41.2 (*C*H), 37.3 (CH_2), 29.9 (CH_2), 24.1 (CH_2), 22.4 (CH_3).

HRMS (ESI) *m/z* = 282.1828 calcd. for $[C_{17}H_{25}NONa]^+$ $[M+Na]^+$, found: 282.1824.

1.4.4 Hydrolysis of α-quaternary amides

2,2-Diphenylbutanoic acid (4):

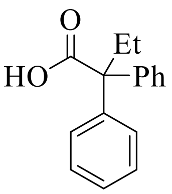

The title compound was prepared following a modified procedure from the literature.[12] Amide **3af** (25.1 mg, 71.0 µmol, 1.0 eq.) was dissolved in 1,4-dioxane (1.0 mL) and sulfuric acid (9 N (aq.), 1.0 mL) in a sealed tube and refluxed at 115 °C for 48 hours. After cooling to 0 °C, the pH of the mixture was adjusted to pH ≥ 10 by addition of sodium hydroxide solution (5 N aq.). The mixture was partitioned between water (5 mL) and CH_2Cl_2 (10 mL). The layers were separated and the aqueous layer was extracted with CH_2Cl_2 (3 x 10 mL). The aqueous layer was brought to pH ≤ 1 by slow addition of sulfuric acid (9 N aq.) at 0 °C and then extracted with CH_2Cl_2 (3 x 15 mL). The combined organic phases were dried over Na_2SO_4 and concentrated under reduced pressure. Purification by RP-MPLC (MeOH/H_2O, gradient from 10% to 90%) gave the title compound as a colourless oil (12.1 mg, 50.4 µmol, 71%).

^{1}H NMR (300 MHz, $CDCl_3$, 300 K): δ_H (ppm) = 7.33 – 7.27 (m, 10H), 2.43 (q, J = 7.3 Hz, 2H), 0.77 (t, J = 7.3 Hz, 3H).

^{13}C NMR (151 MHz, $CDCl_3$, 300 K): δ_C (ppm) = 179.0 (C_q), 142.3 (C_q), 129.1 (*C*H), 127.9 (*C*H), 126.9 (*C*H), 60.6 (C_q), 30.8 (CH_2), 9.8 (CH_3).

HRMS (ESI) *m/z* = 263.1043 calcd. for $[C_{16}H_{16}O_2Na]^+$ $[M+Na]^+$, found: 263.1041.

The analytical data are consistent with those reported in the literature.[35]

2. Supplementary Figures

2.1 NMR Spectra of compounds

^{1}H NMR (300 MHz, CDCl$_3$):

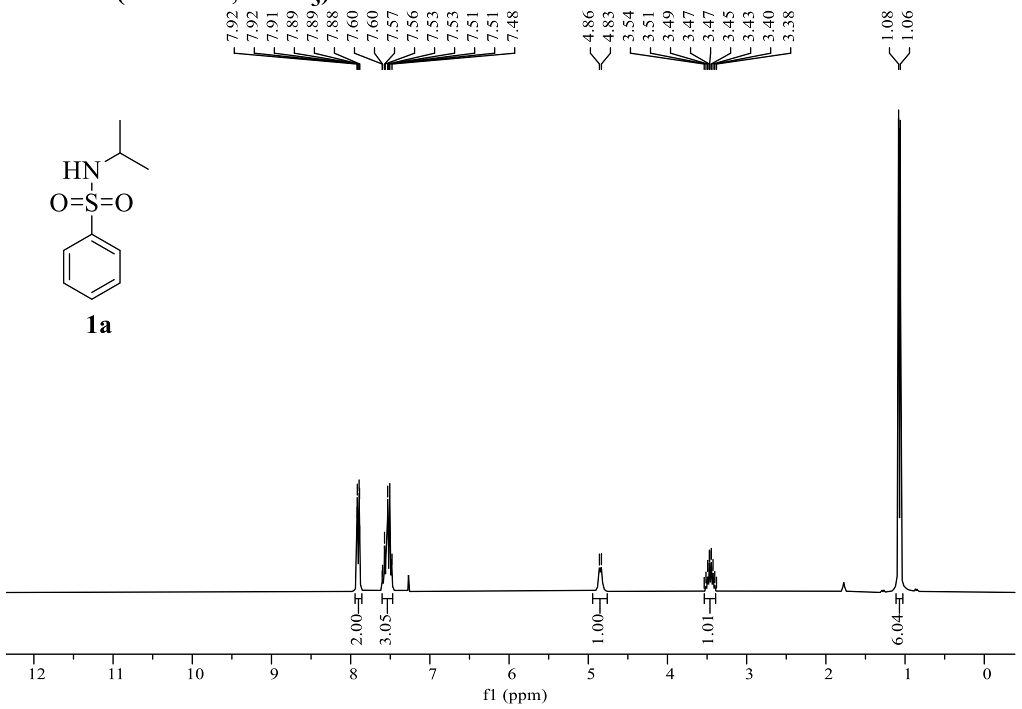

Supplementary Figure 2: ^{1}H NMR (300 MHz, CDCl$_3$) spectrum of compound **1a**.

^{13}C NMR (75 MHz, CDCl$_3$):

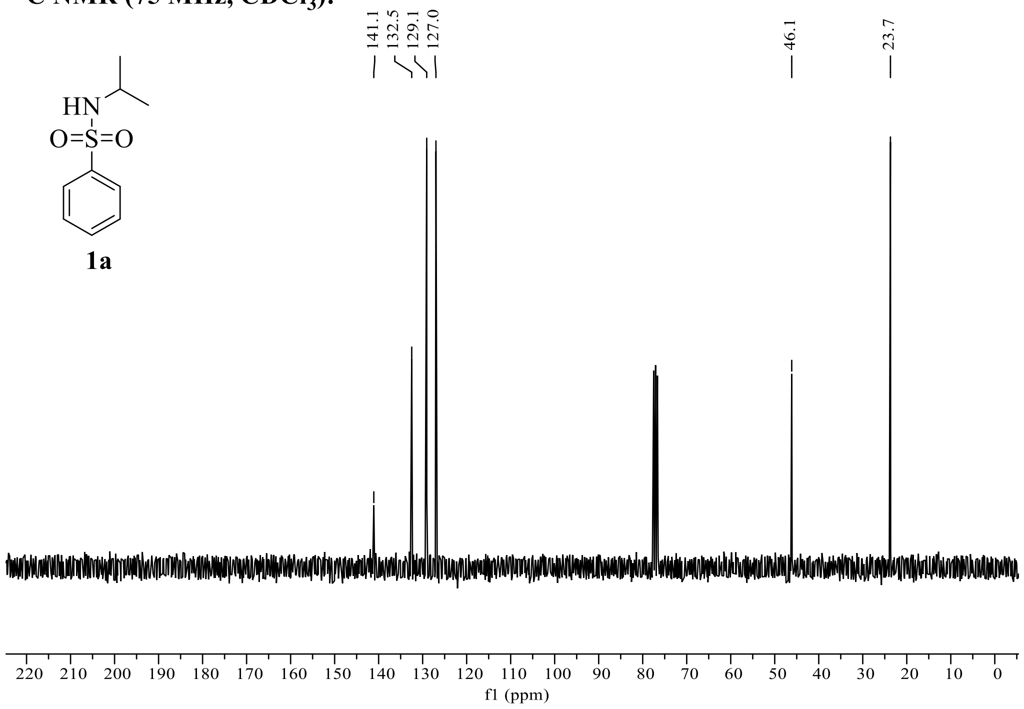

Supplementary Figure 3: ^{13}C NMR (75 MHz, CDCl$_3$) spectrum of compound **1a**.

^{1}H NMR (300 MHz, $CDCl_3$):

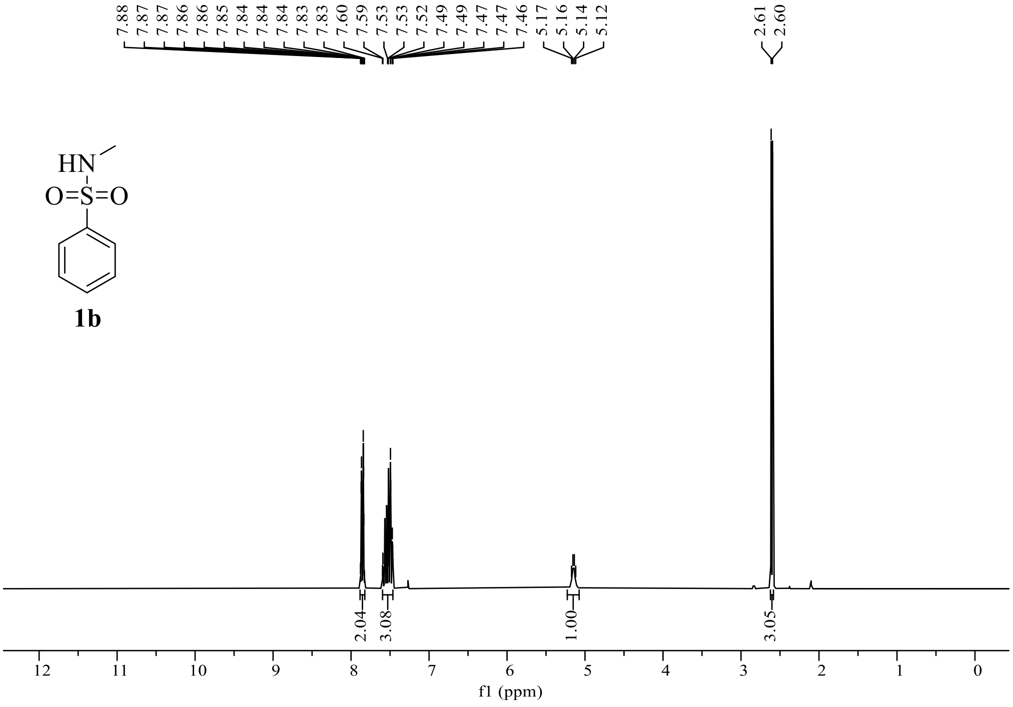

Supplementary Figure 4: ^{1}H NMR (300 MHz, $CDCl_3$) spectrum of compound **1b**.

^{13}C NMR (75 MHz, $CDCl_3$):

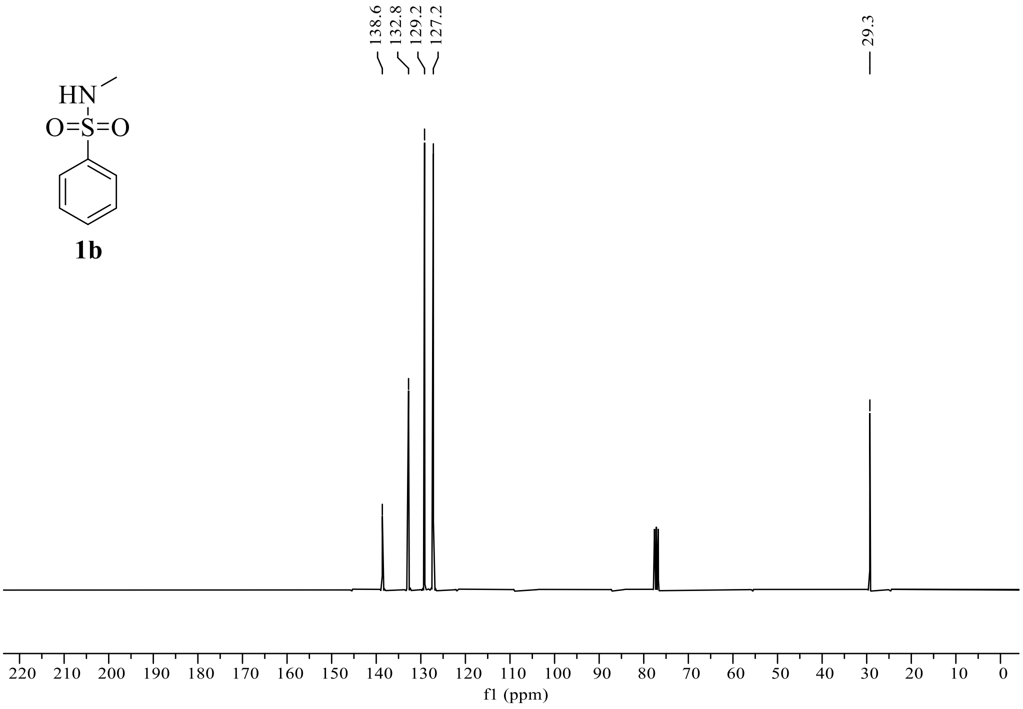

Supplementary Figure 5: ^{13}C NMR (75 MHz, $CDCl_3$) spectrum of compound **1b**.

^{1}H NMR (600 MHz, DMSO-d^{6}):

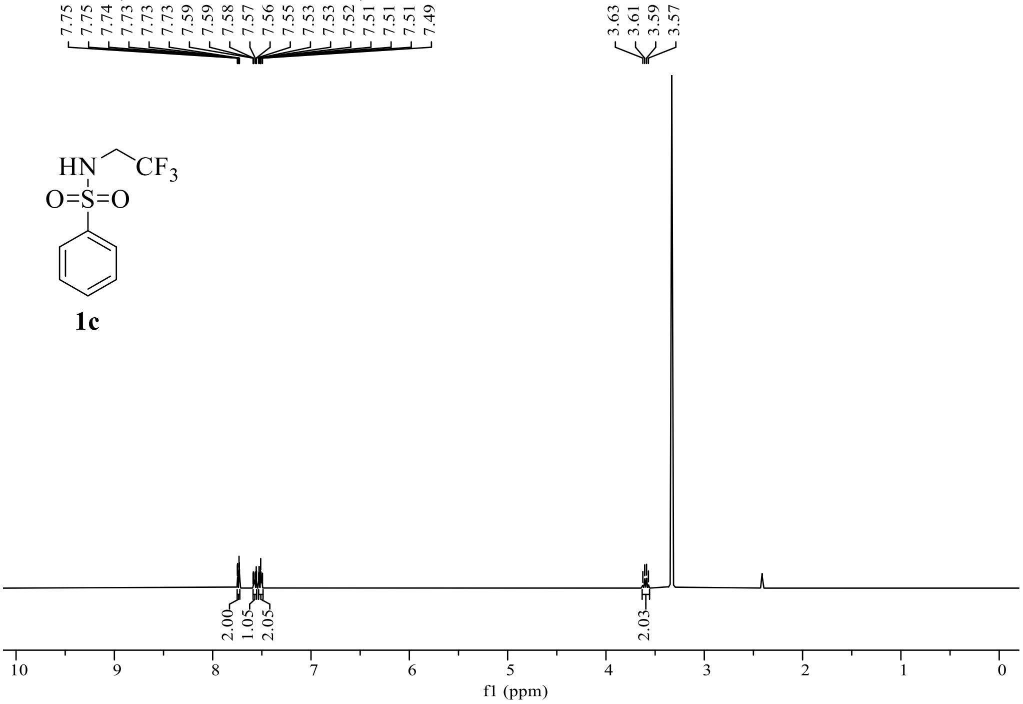

Supplementary Figure 6: ^{1}H NMR (600 MHz, DMSO-d^{6}) spectrum of compound **1c**.

^{13}C{^{1}H,^{19}F} NMR (151 MHz, DMSO-d^{6}):

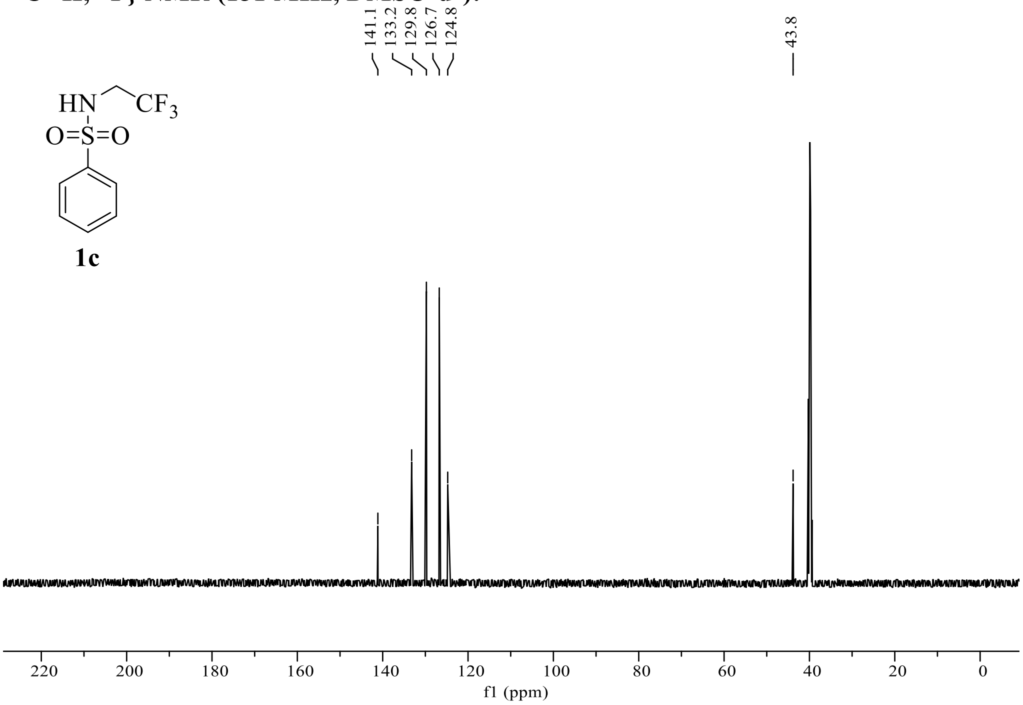

Supplementary Figure 7: ^{13}C NMR (151 MHz, DMSO-d^{6}) spectrum of compound **1c**.

$^{19}F\{^1H,^{13}C\}$ NMR (564 MHz, DMSO-d^6):

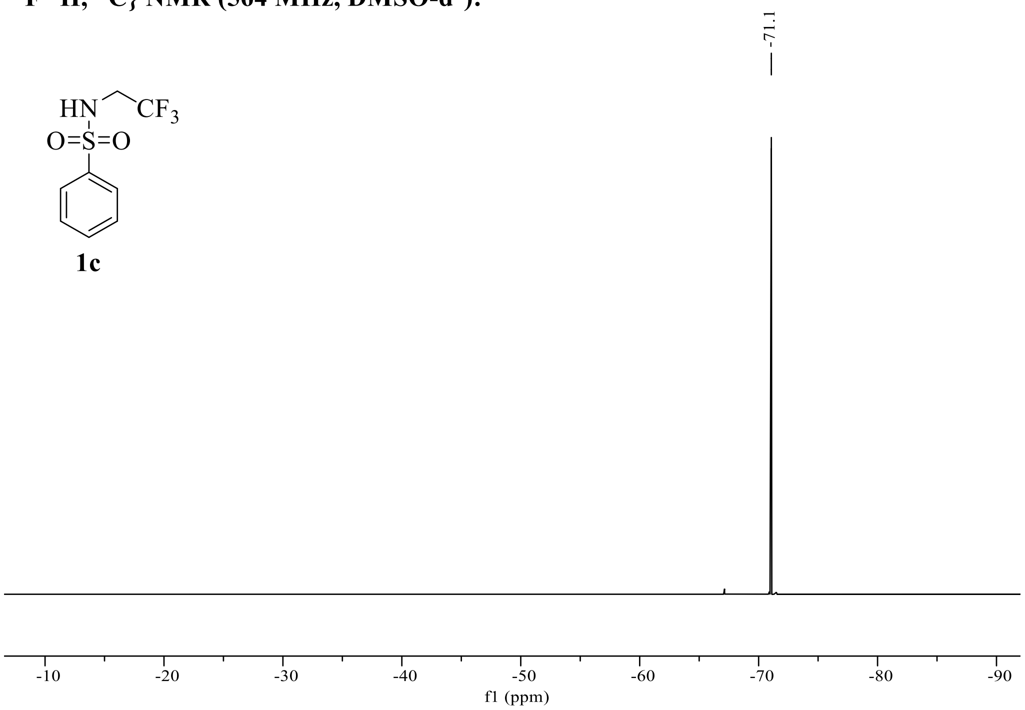

Supplementary Figure 8: ^{19}F NMR (564 MHz, $CDCl_3$) spectrum of compound **1c**.

^{1}H NMR (300 MHz, $CDCl_3$):

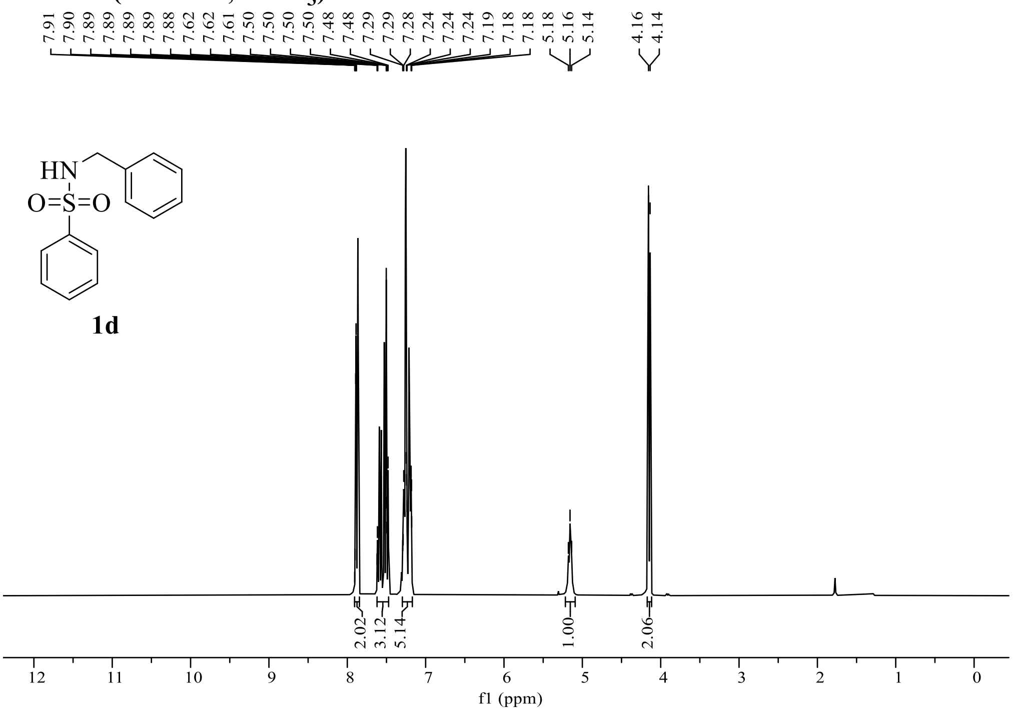

Supplementary Figure 9: ^{1}H NMR (300 MHz, $CDCl_3$) spectrum of compound **1d**.

^{13}C NMR (75 MHz, $CDCl_3$):

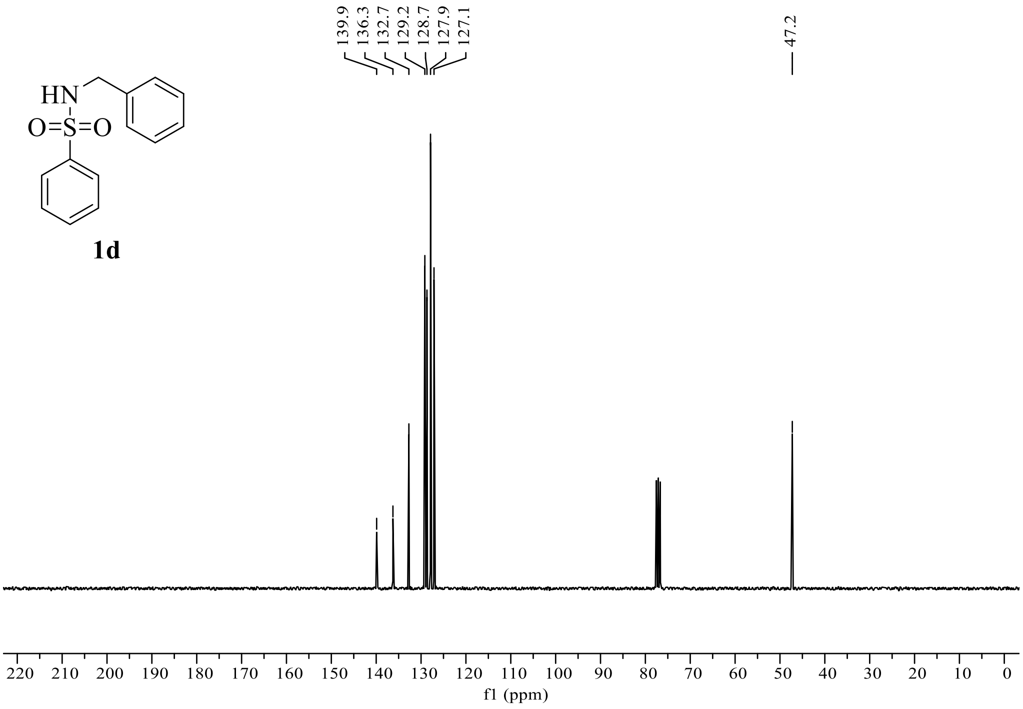

Supplementary Figure 10: ^{13}C NMR (75 MHz, $CDCl_3$) spectrum of compound **1d**.

^{1}H NMR (300 MHz, $CDCl_3$):

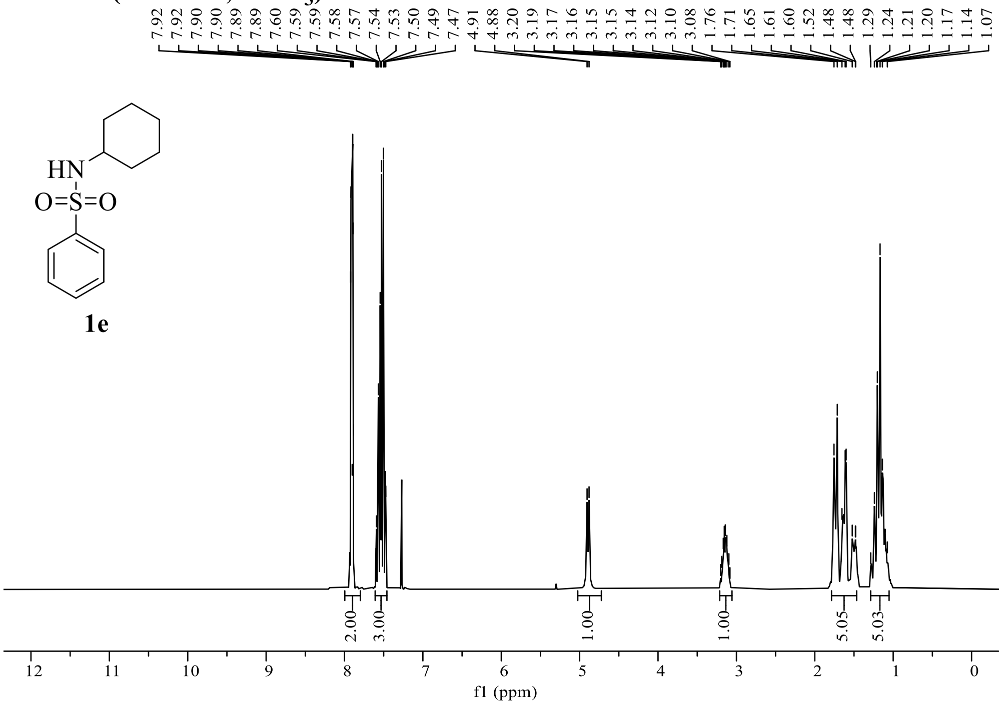

Supplementary Figure 11: ^{1}H NMR (300 MHz, $CDCl_3$) spectrum of compound **1e**.

^{13}C NMR (75 MHz, $CDCl_3$):

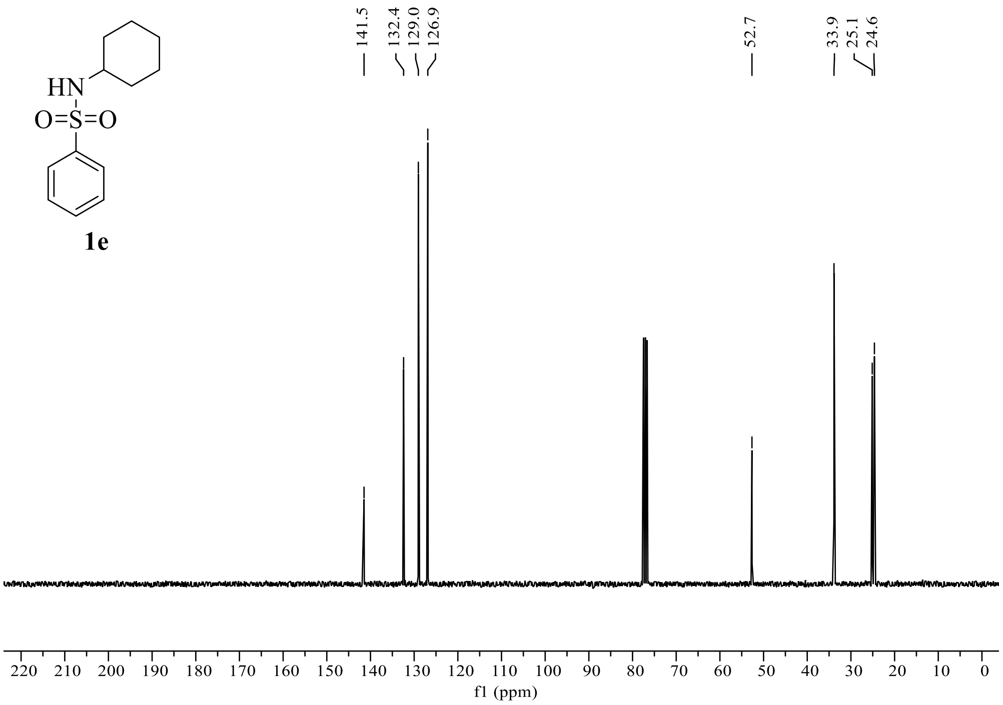

Supplementary Figure 12: ^{13}C NMR (75 MHz, $CDCl_3$) spectrum of compound **1e**.

^{1}H NMR (300 MHz, $CDCl_3$):

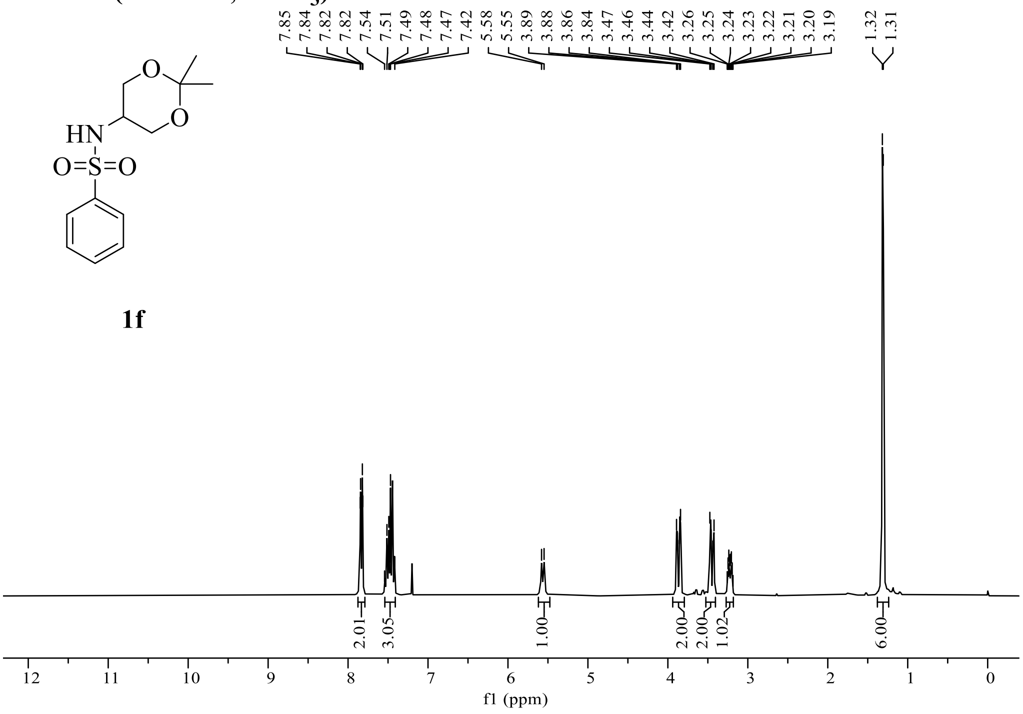

Supplementary Figure 13: ^{1}H NMR (300 MHz, $CDCl_3$) spectrum of compound **1f**.

^{13}C NMR (75 MHz, $CDCl_3$):

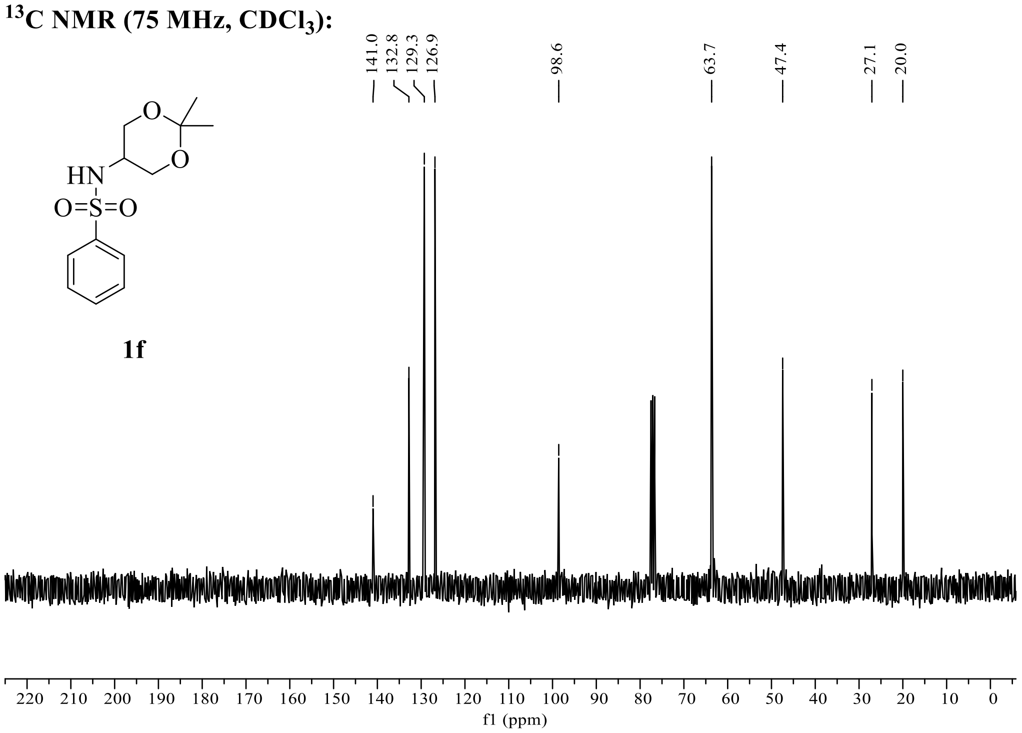

Supplementary Figure 14: ^{13}C NMR (75 MHz, $CDCl_3$) spectrum of compound **1f**.

^{1}H NMR (300 MHz, CDCl$_3$):

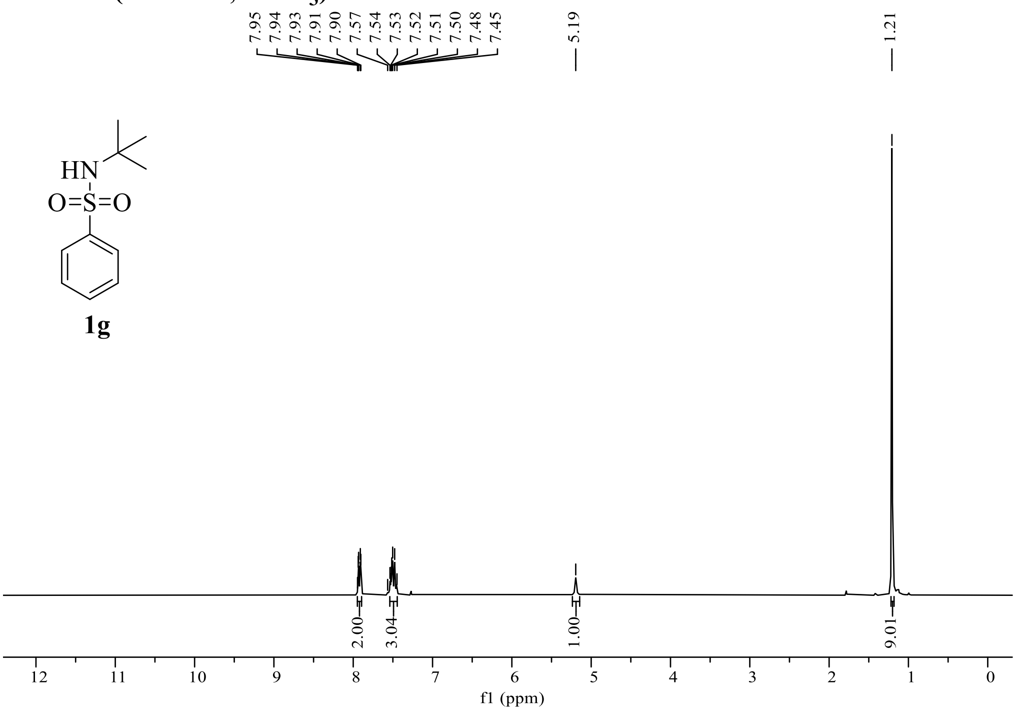

Supplementary Figure 15: ^{1}H NMR (300 MHz, CDCl$_3$) spectrum of compound **1g**.

^{13}C NMR (75 MHz, CDCl$_3$):

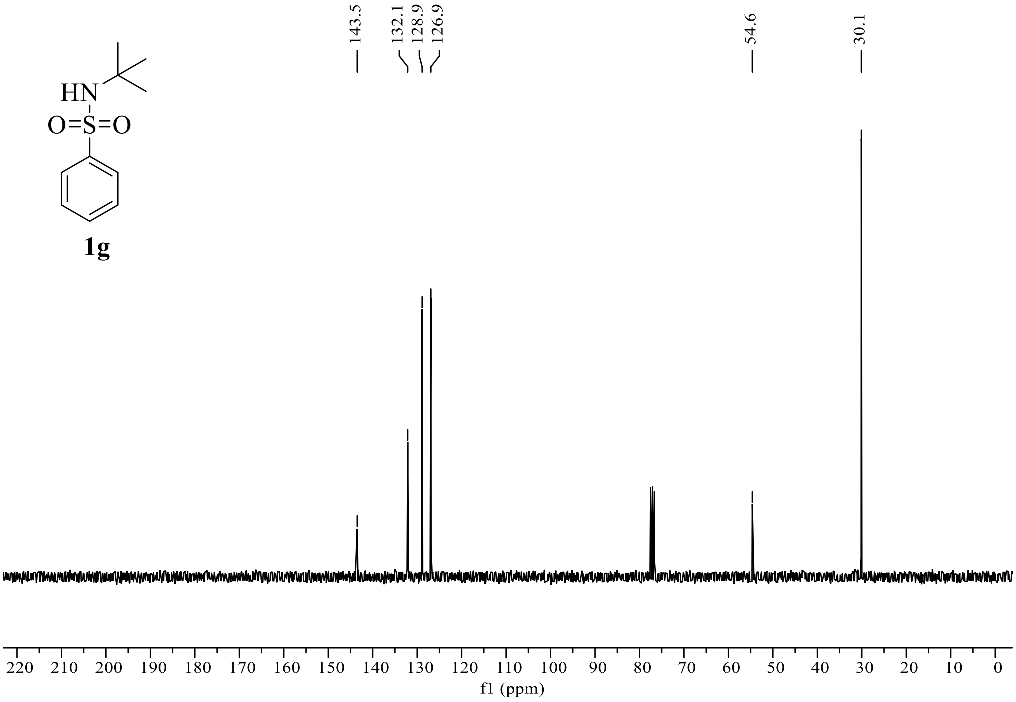

Supplementary Figure 16: ^{13}C NMR (75 MHz, CDCl$_3$) spectrum of compound **1g**.

^{1}H NMR (300 MHz, $CDCl_3$):

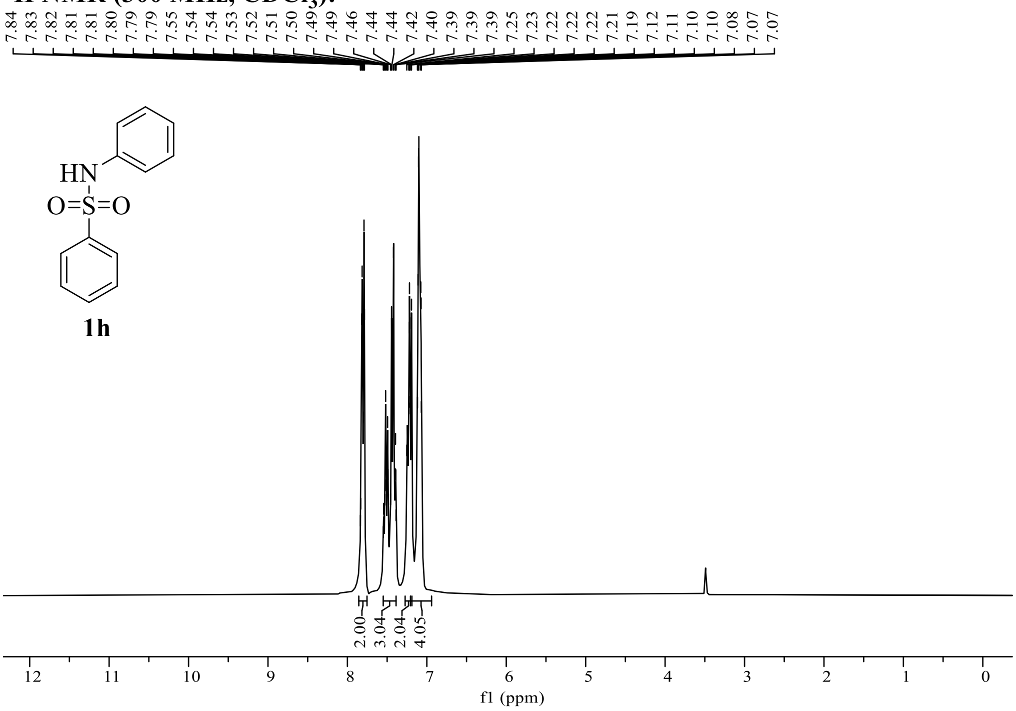

Supplementary Figure 17: ^{1}H NMR (300 MHz, $CDCl_3$) spectrum of compound **1h**.

^{13}C NMR (75 MHz, $CDCl_3$):

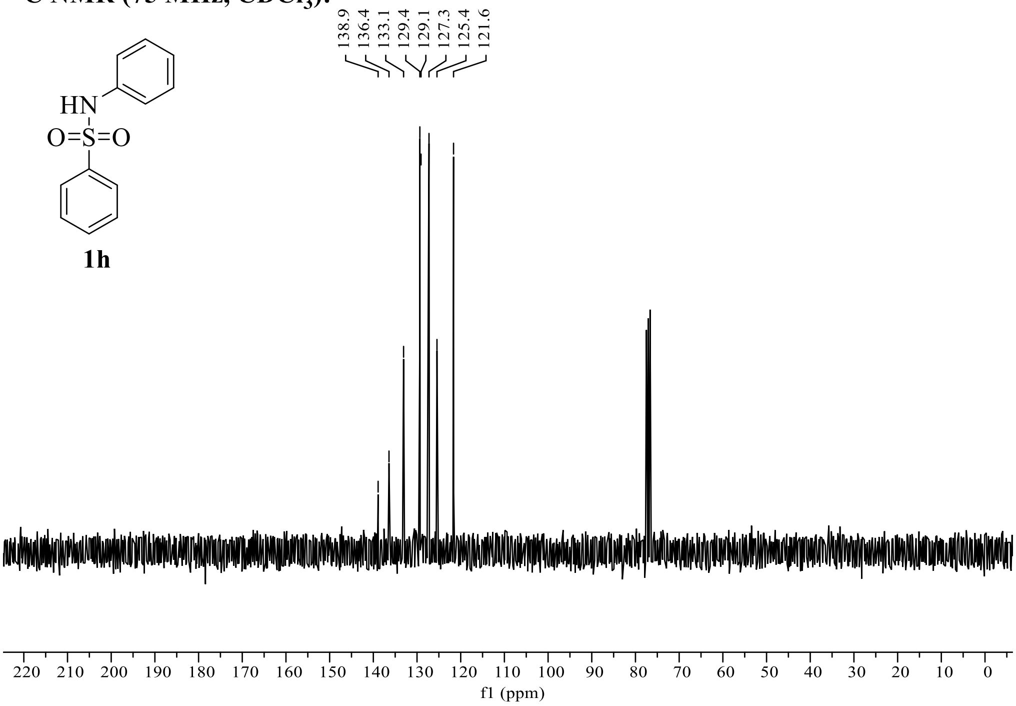

Supplementary Figure 18: ^{13}C NMR (75 MHz, $CDCl_3$) spectrum of compound **1h**.

^{1}H NMR (300 MHz, $CDCl_3$):

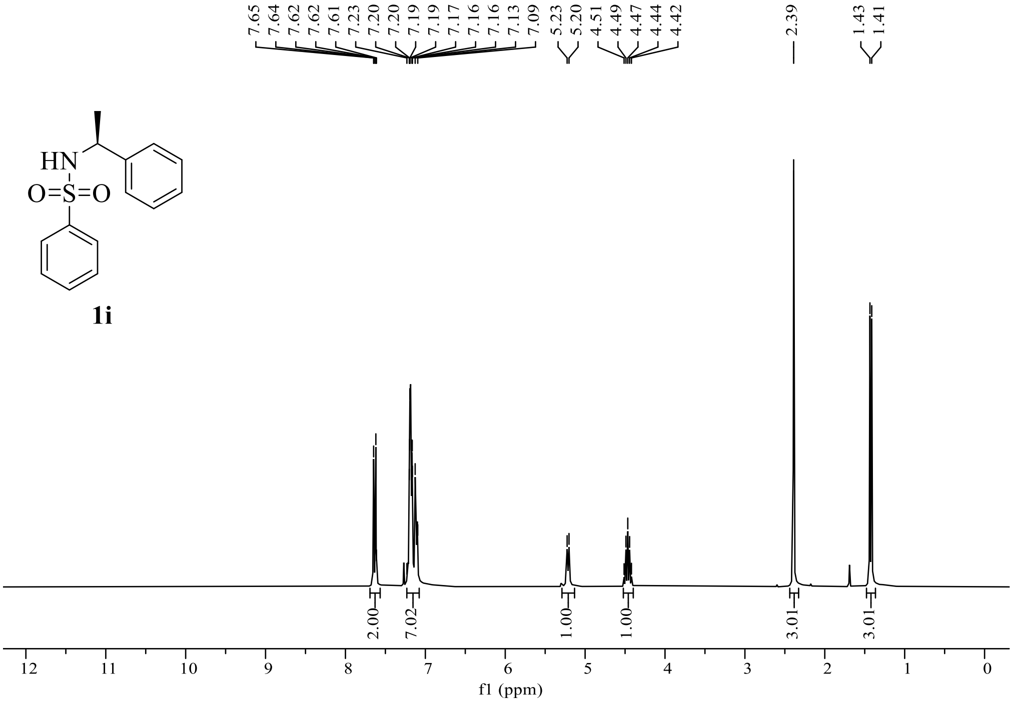

Supplementary Figure 19: ^{1}H NMR (300 MHz, $CDCl_3$) spectrum of compound **1i**.

^{13}C NMR (75 MHz, $CDCl_3$):

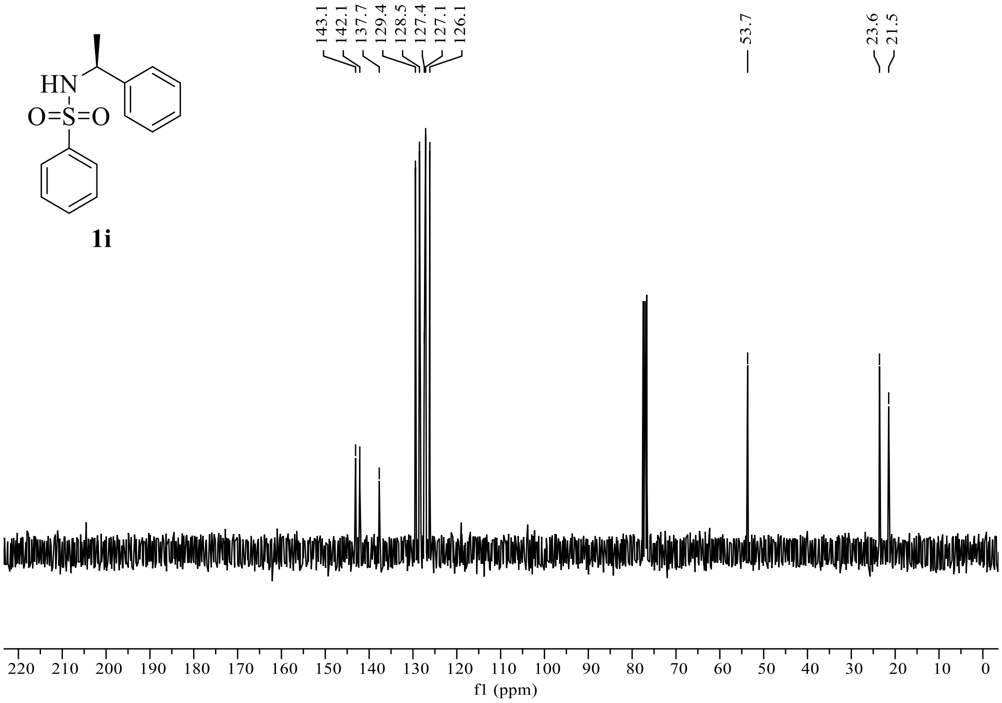

Supplementary Figure 20: ^{13}C NMR (75 MHz, $CDCl_3$) spectrum of compound **1i**.

^{1}H NMR (300 MHz, $CDCl_3$):

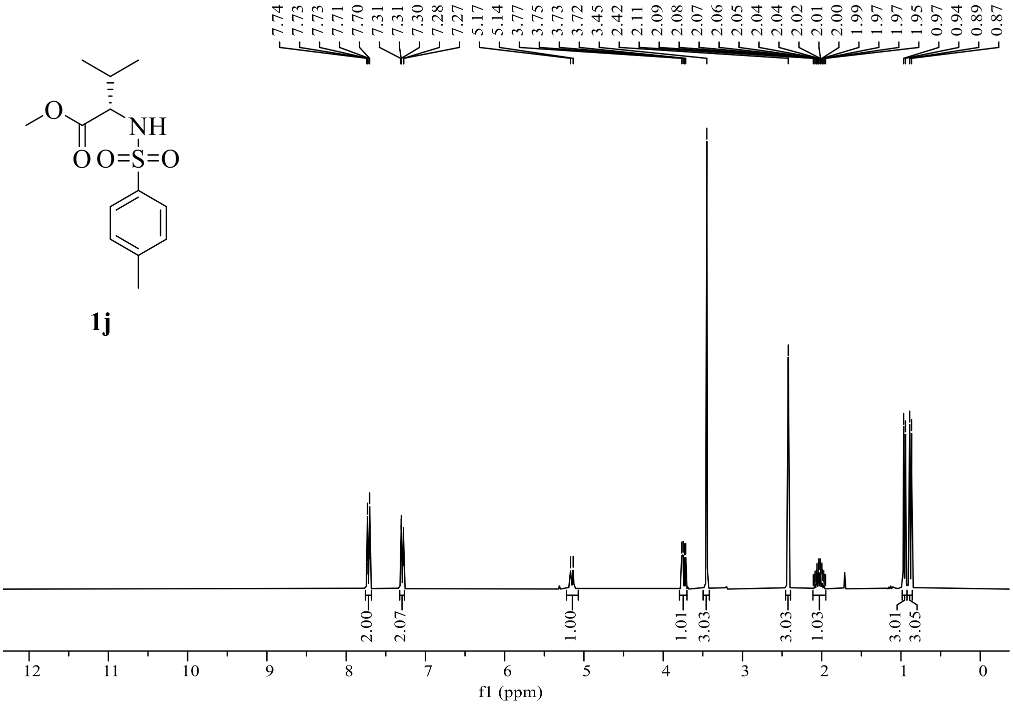

Supplementary Figure 21: ^{1}H NMR (300 MHz, $CDCl_3$) spectrum of compound **1j**.

^{13}C NMR (75 MHz, $CDCl_3$):

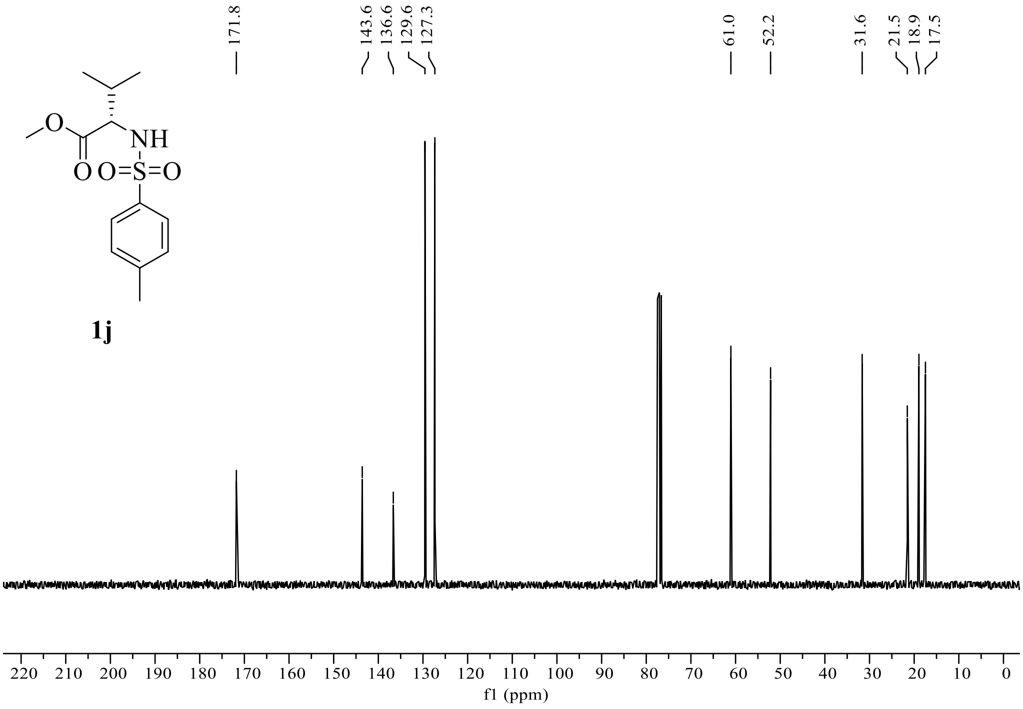

Supplementary Figure 22: ^{13}C NMR (75 MHz, $CDCl_3$) spectrum of compound **1j**.

^{1}H NMR (300 MHz, $CDCl_3$):

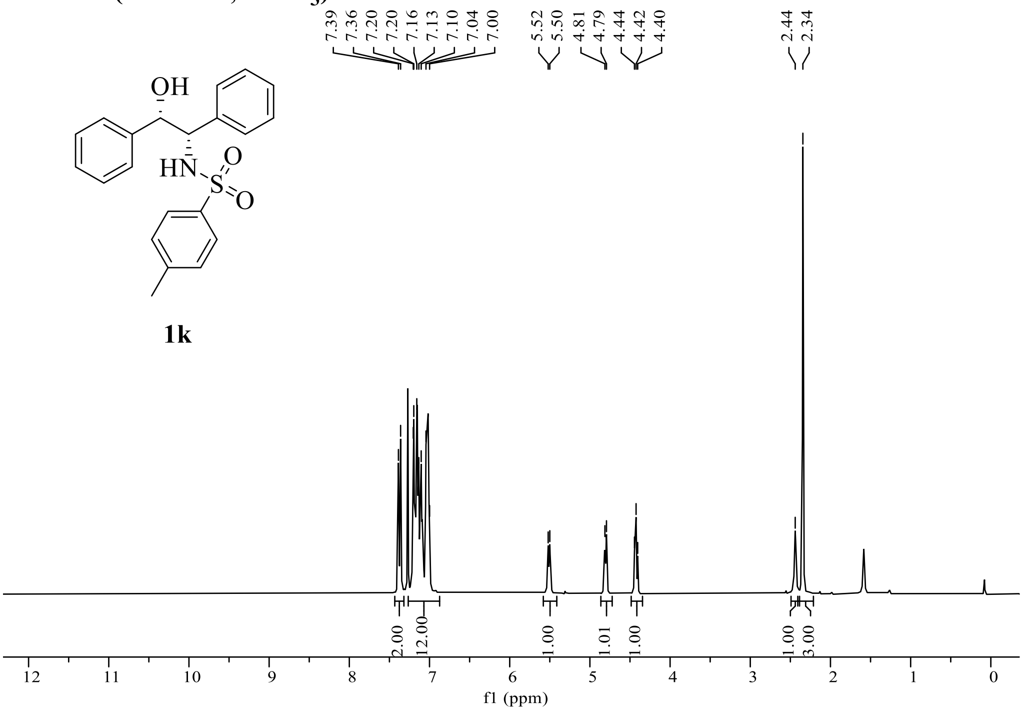

Supplementary Figure 23: ^{1}H NMR (300 MHz, $CDCl_3$) spectrum of compound **1k**.

^{13}C NMR (75 MHz, $CDCl_3$):

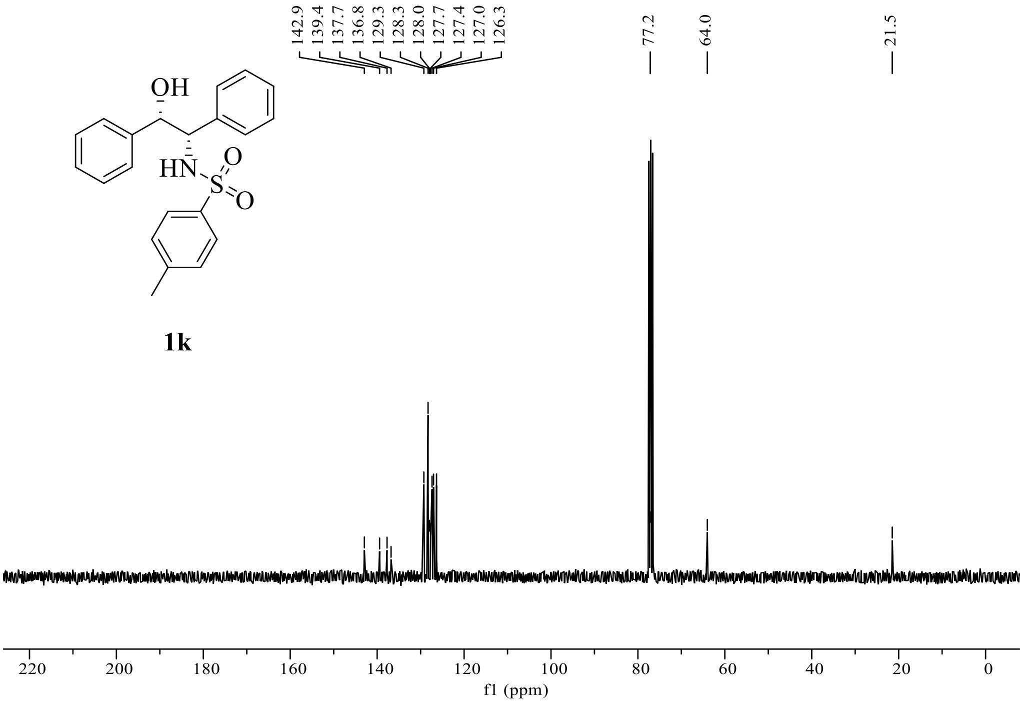

Supplementary Figure 24: ^{13}C NMR (75 MHz, $CDCl_3$) spectrum of compound **1k**.

^{1}H NMR (300 MHz, CDCl$_3$):

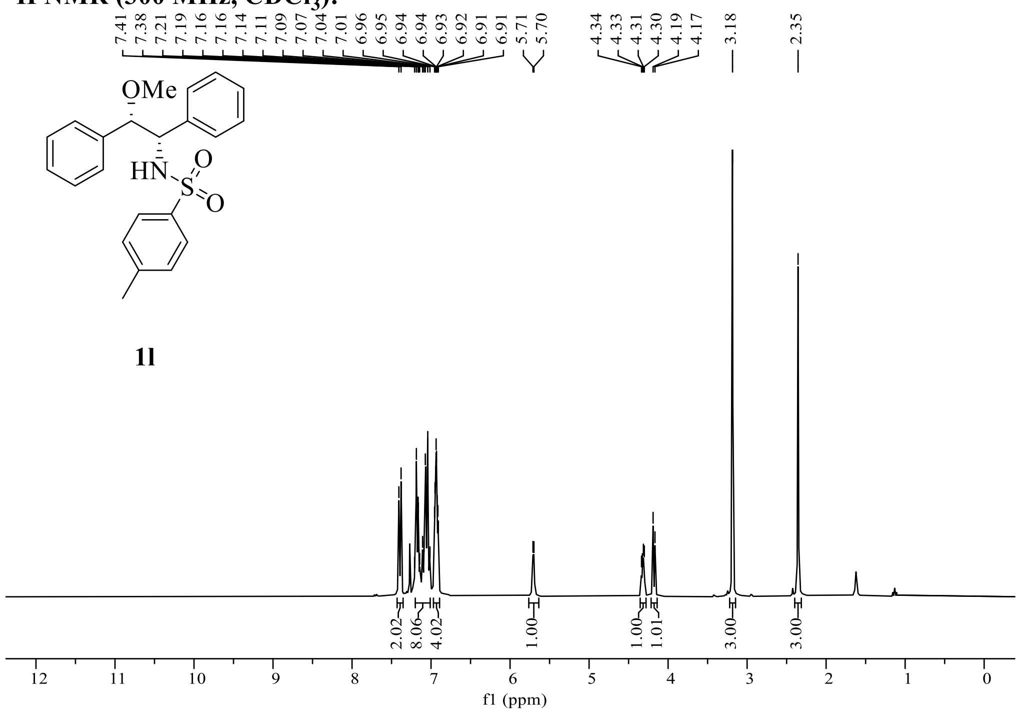

Supplementary Figure 25: ^{1}H NMR (300 MHz, CDCl$_3$) spectrum of compound **1l**.

^{13}C NMR (75 MHz, CDCl$_3$):

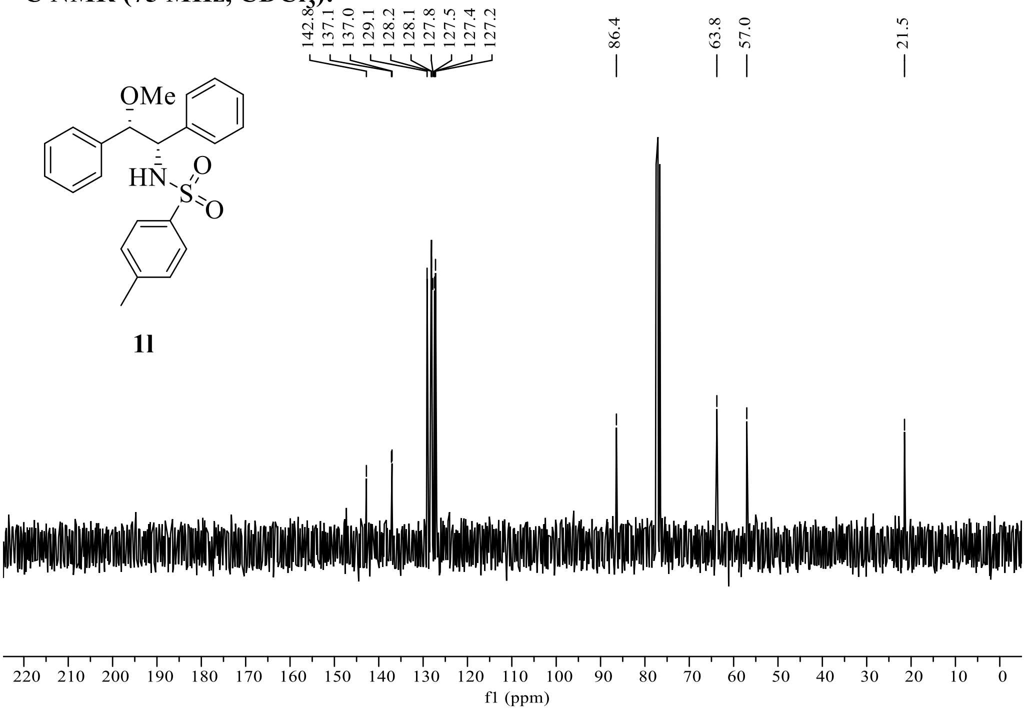

Supplementary Figure 26: ^{13}C NMR (75 MHz, CDCl$_3$) spectrum of compound **1l**.

^{1}H NMR (300 MHz, $CDCl_3$):

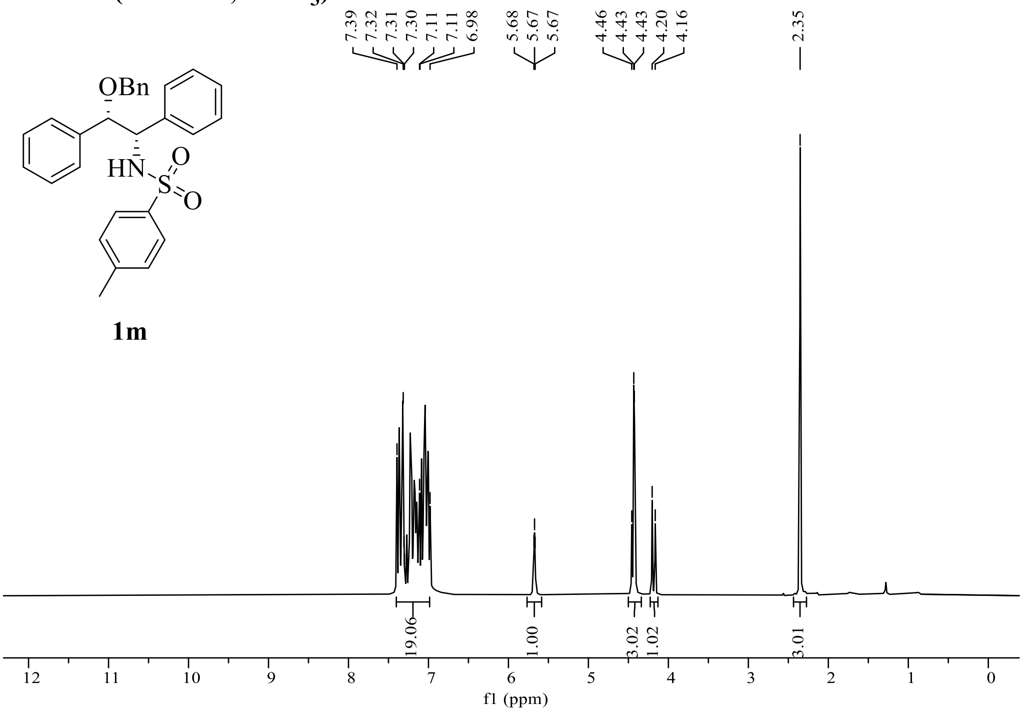

Supplementary Figure 27: ^{1}H NMR (300 MHz, $CDCl_3$) spectrum of compound **1m**.

^{13}C NMR (151 MHz, $CDCl_3$):

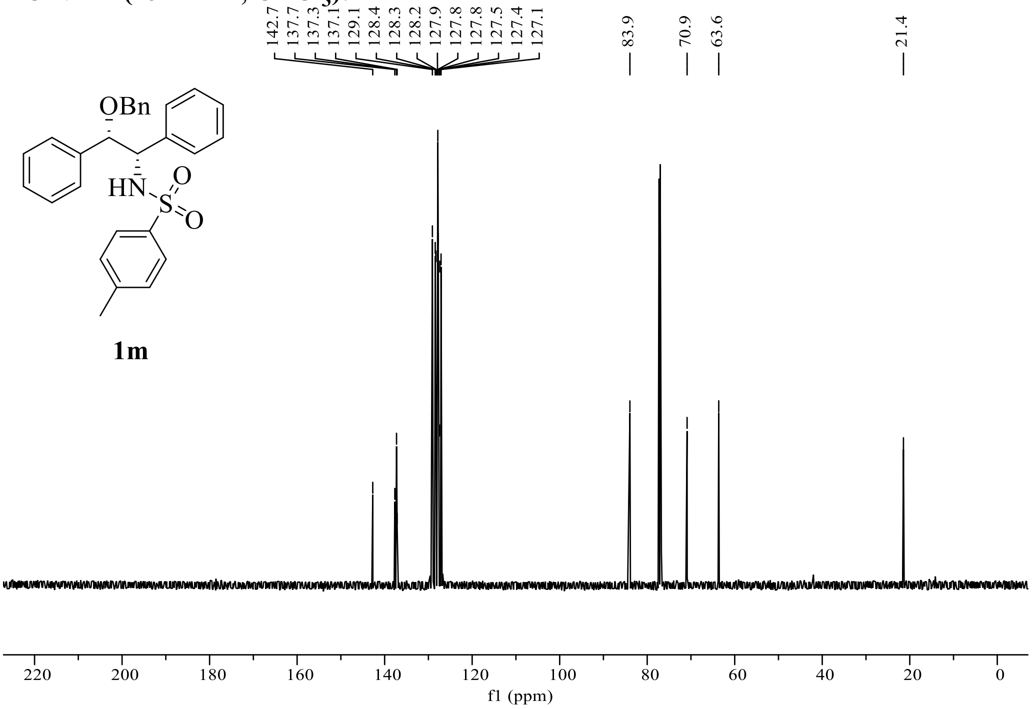

Supplementary Figure 28: ^{13}C NMR (75 MHz, $CDCl_3$) spectrum of compound **1m**.

^{1}H NMR (300 MHz, $CDCl_3$):

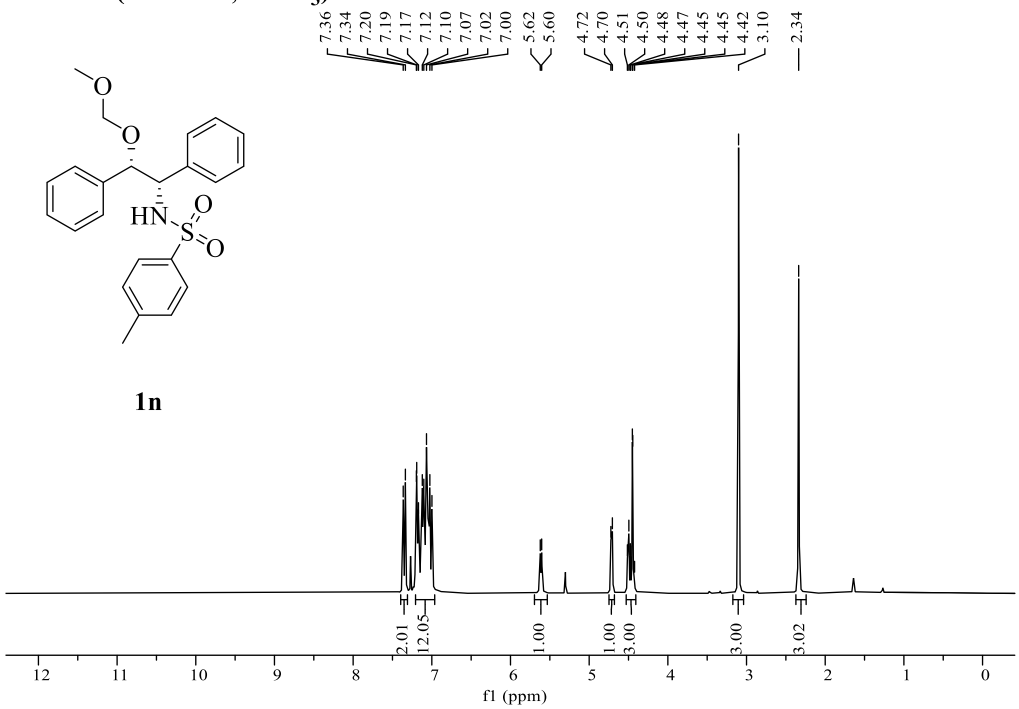

Supplementary Figure 29: ^{1}H NMR (300 MHz, $CDCl_3$) spectrum of compound **1n**.

^{13}C NMR (75 MHz, $CDCl_3$):

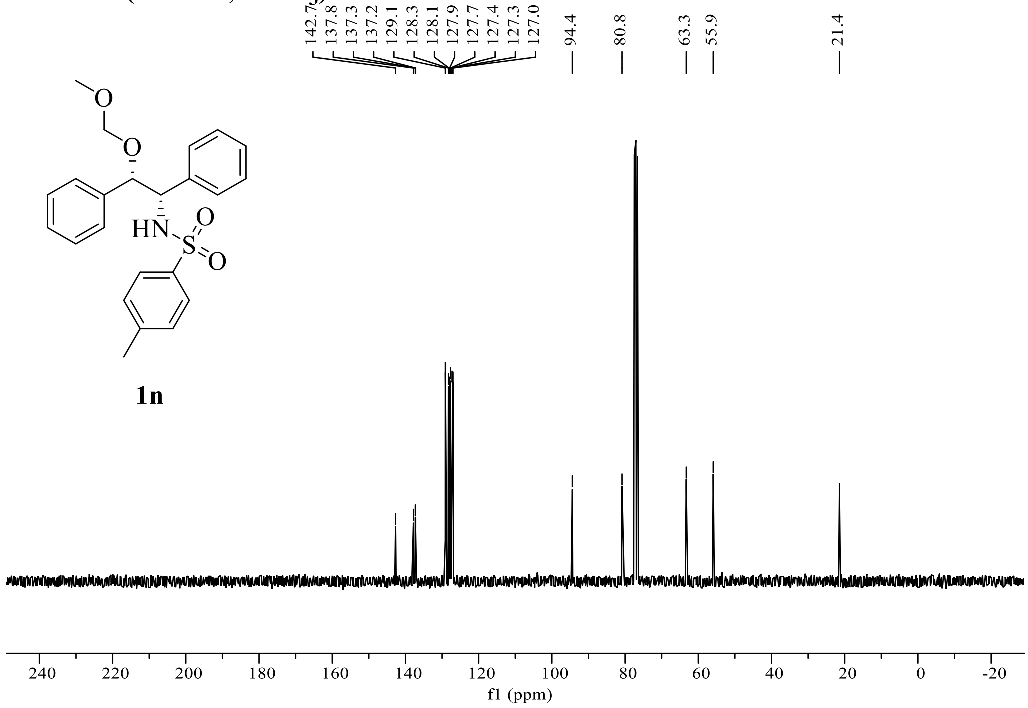

Supplementary Figure 30: ^{13}C NMR (75 MHz, $CDCl_3$) spectrum of compound **1n**.

^{1}H NMR (300 MHz, $CDCl_3$):

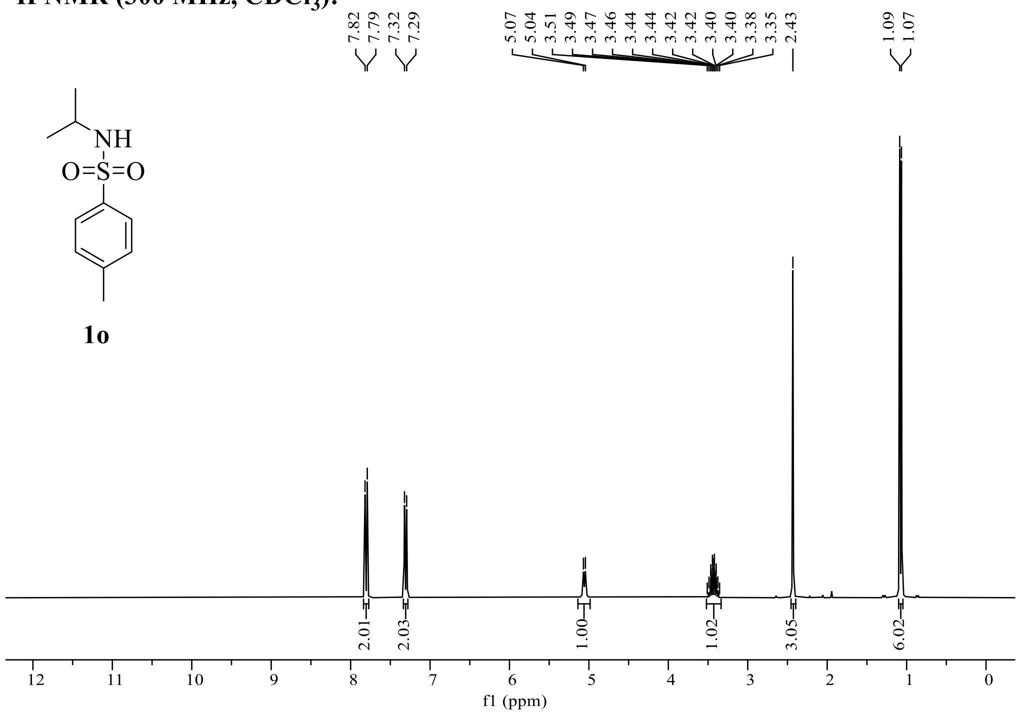

Supplementary Figure 31: ^{1}H NMR (300 MHz, $CDCl_3$) spectrum of compound **1o**.

^{13}C NMR (75 MHz, $CDCl_3$):

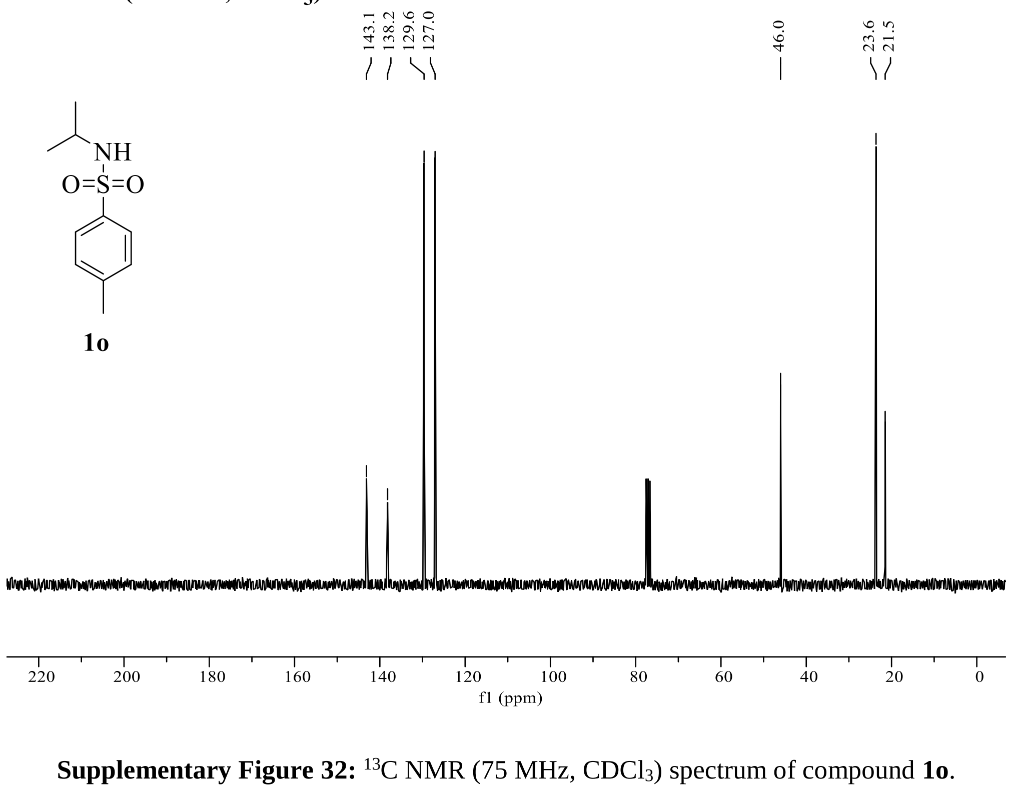

Supplementary Figure 32: ^{13}C NMR (75 MHz, $CDCl_3$) spectrum of compound **1o**.

^{1}H NMR (300 MHz, $CDCl_3$):

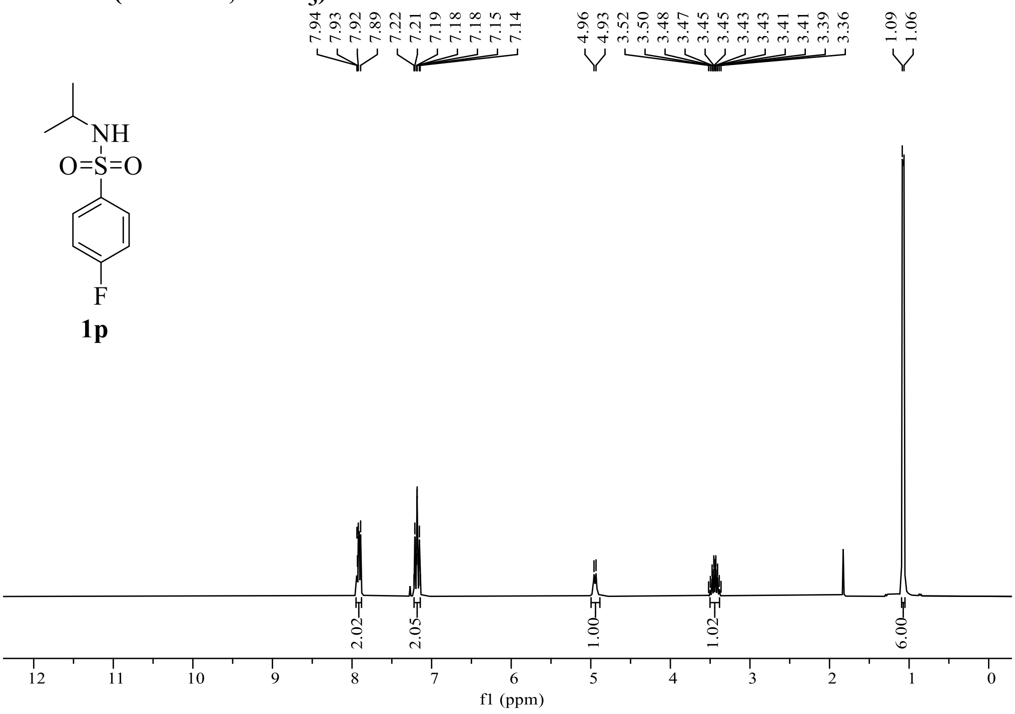

Supplementary Figure 33: ^{1}H NMR (300 MHz, $CDCl_3$) spectrum of compound **1p**.

^{13}C NMR (75 MHz, $CDCl_3$):

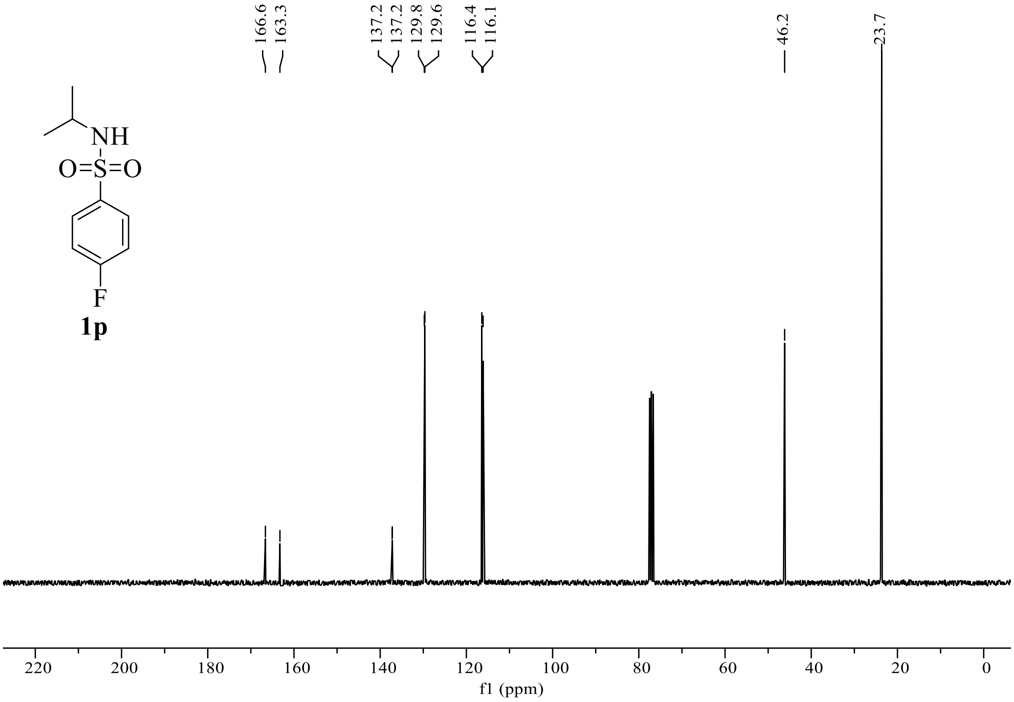

Supplementary Figure 34: ^{13}C NMR (75 MHz, $CDCl_3$) spectrum of compound **1p**.

^{19}F NMR (283 MHz, $CDCl_3$):

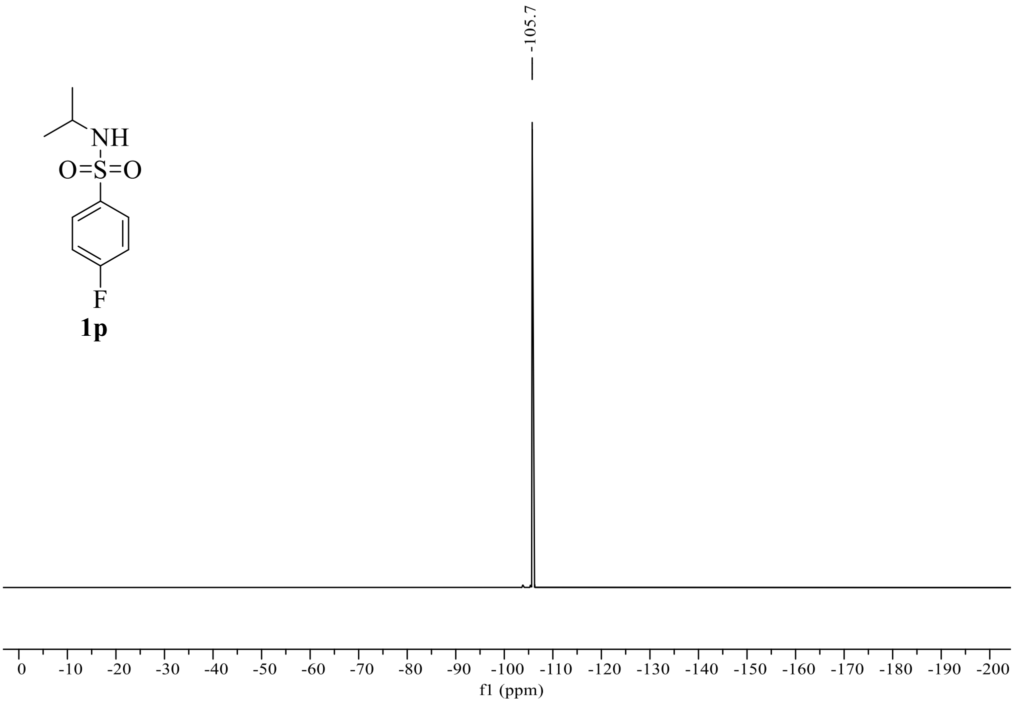

Supplementary Figure 35: ^{19}F NMR (283 MHz, $CDCl_3$) spectrum of compound **1p**.

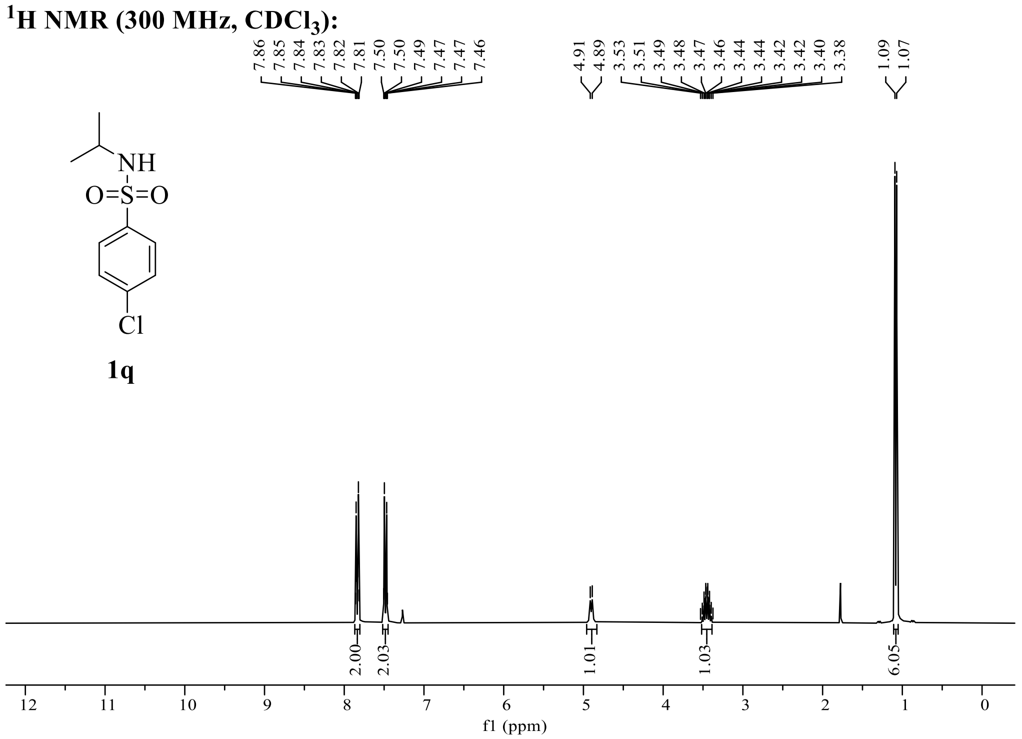

Supplementary Figure 36: ^{1}H NMR (300 MHz, $CDCl_3$) spectrum of compound **1q**.

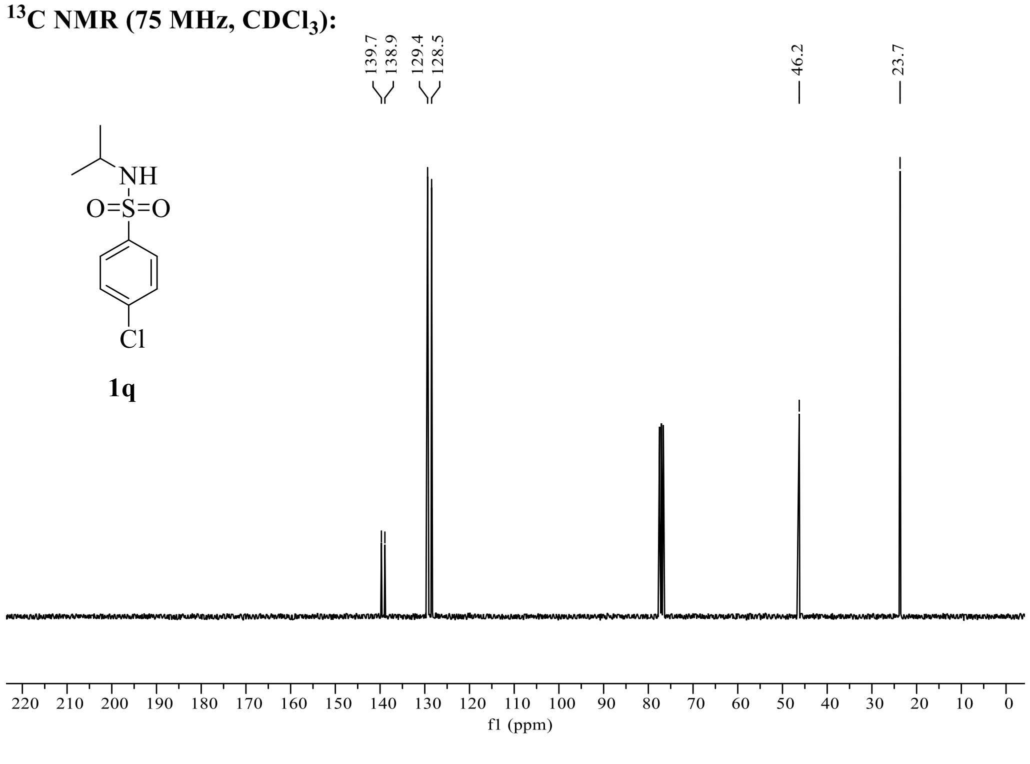

Supplementary Figure 37: ^{13}C NMR (75 MHz, $CDCl_3$) spectrum of compound **1q**.

^{1}H NMR (400 MHz, CDCl$_3$):

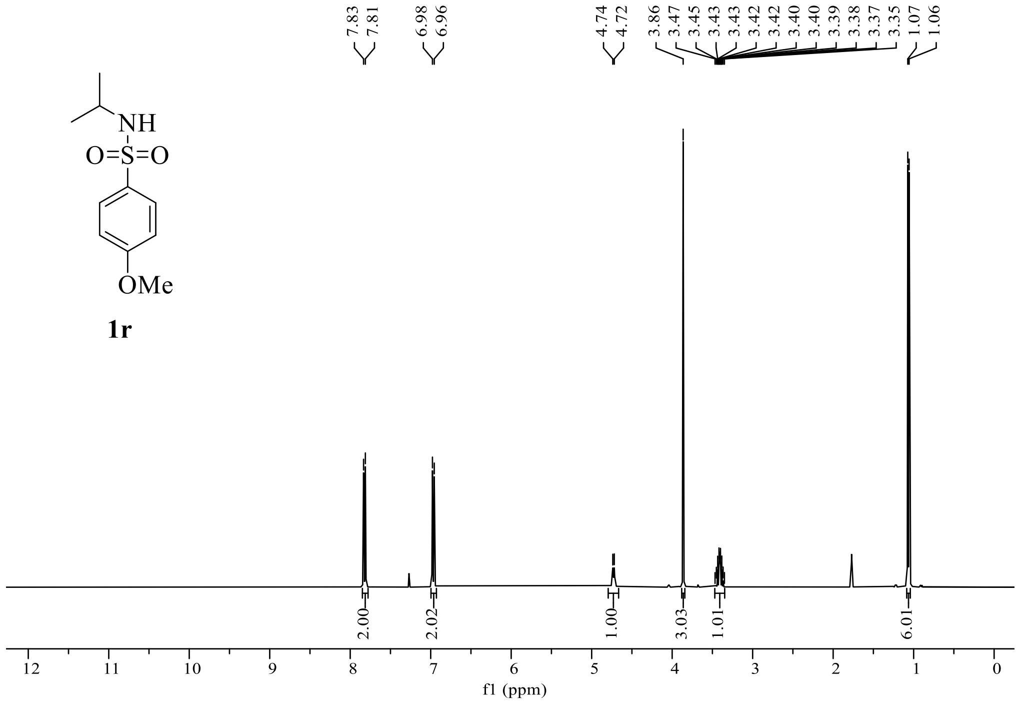

Supplementary Figure 38: ^{1}H NMR (400 MHz, CDCl$_3$) spectrum of compound **1r**.

^{13}C NMR (101 MHz, CDCl$_3$):

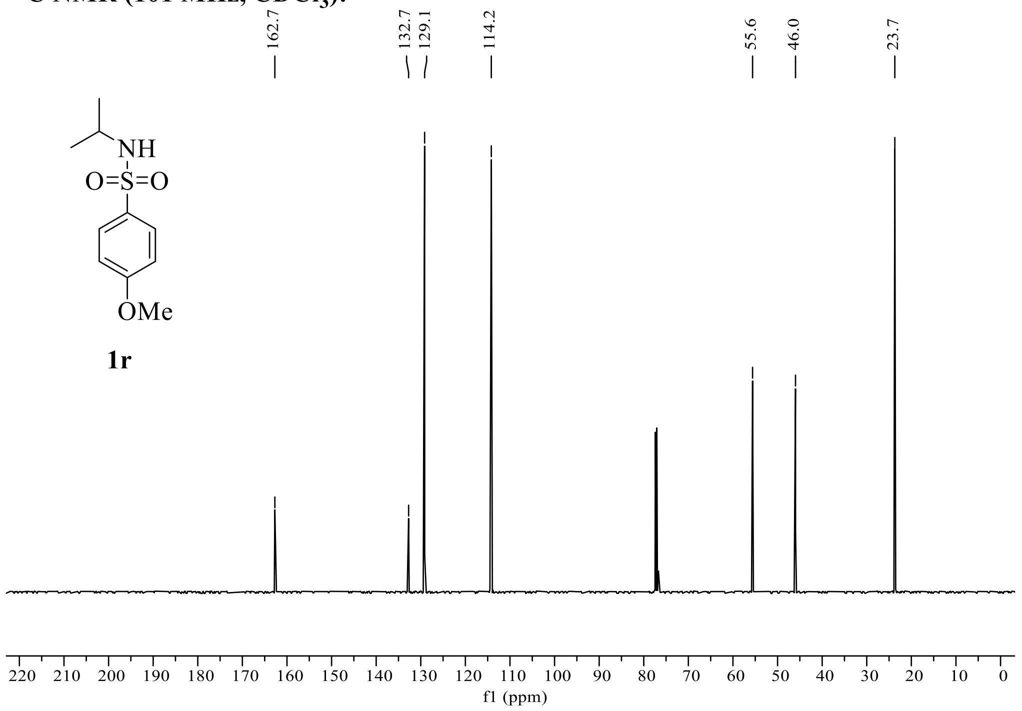

Supplementary Figure 39: ^{13}C NMR (101 MHz, CDCl$_3$) spectrum of compound **1r**.

^{1}H NMR (600 MHz, $CDCl_3$):

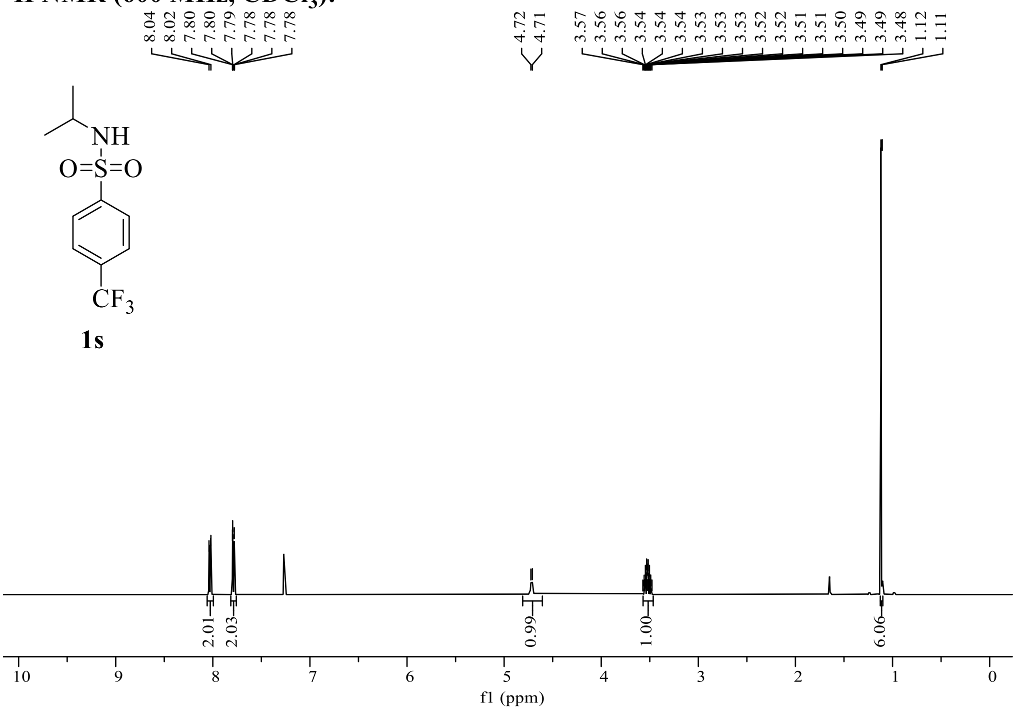

Supplementary Figure 40: ^{1}H NMR (600 MHz, $CDCl_3$) spectrum of compound **1s**.

$^{13}C\{^{1}H,^{19}F\}$ NMR (151 MHz, $CDCl_3$):

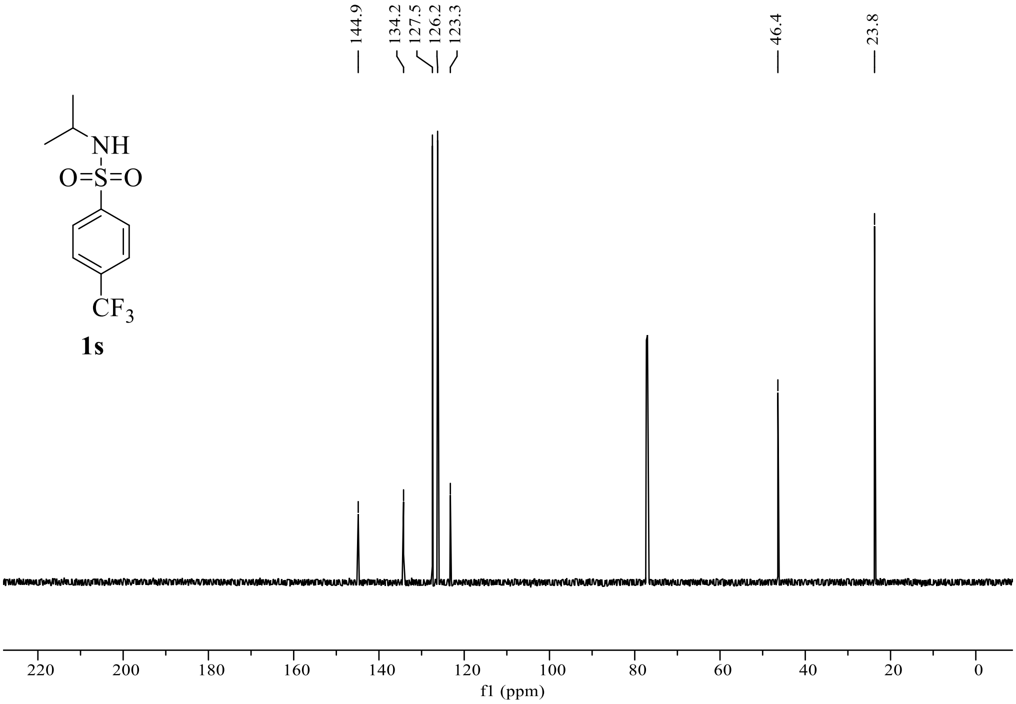

Supplementary Figure 41: $^{13}C\{^{1}H,^{19}F\}$ NMR (151 MHz, $CDCl_3$) spectrum of compound **1s**.

^{19}F {^{1}H, ^{13}C} NMR (564 MHz, CDCl$_3$):

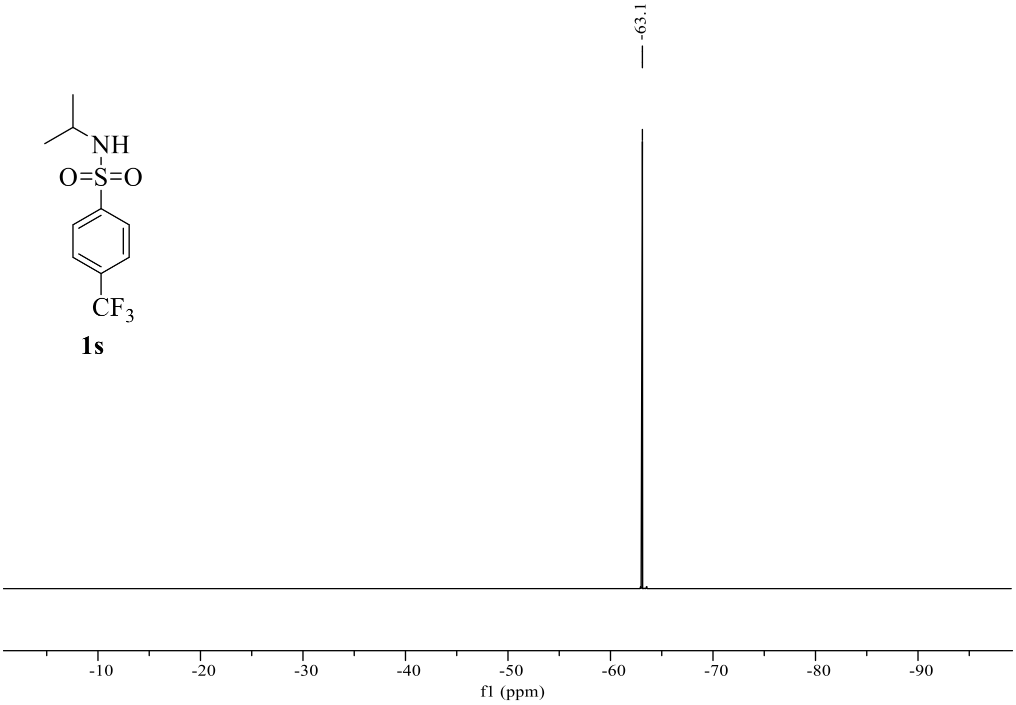

Supplementary Figure 42: $^{19}F\{^{1}H,^{13}C\}$ NMR (564 MHz, $CDCl_3$) spectrum of compound **1s**.

^{1}H NMR (300 MHz, $CDCl_3$):

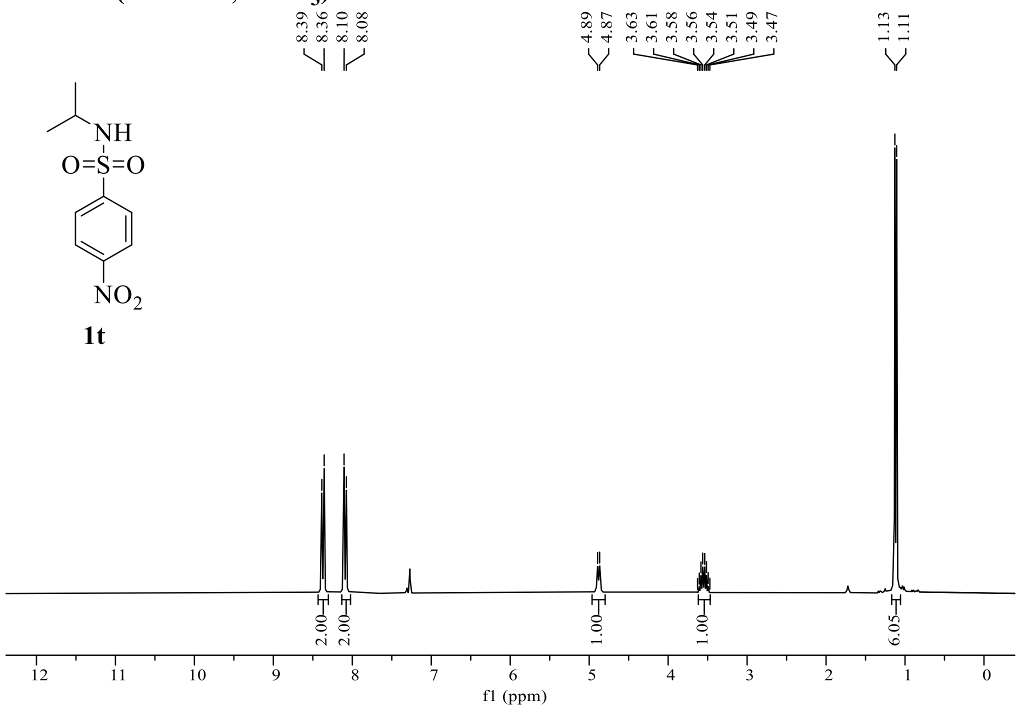

Supplementary Figure 43: ^{1}H NMR (300 MHz, $CDCl_3$) spectrum of compound **1t**.

^{13}C NMR (75 MHz, $CDCl_3$):

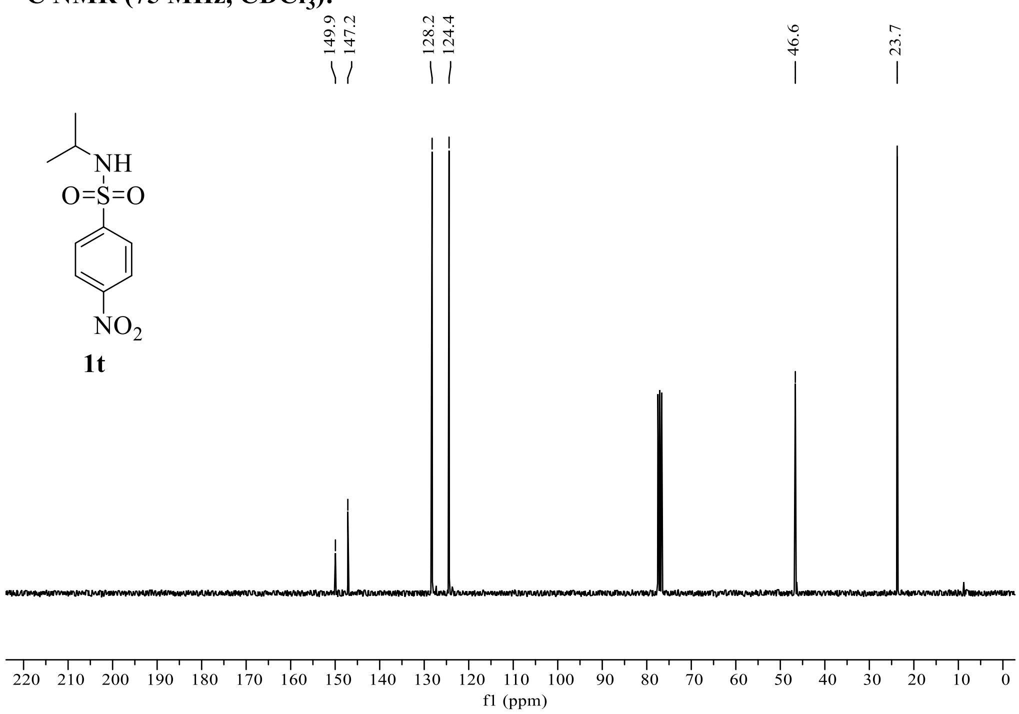

Supplementary Figure 44: ^{13}C NMR (76 MHz, $CDCl_3$) spectrum of compound **1t**.

^{1}H NMR (300 MHz, $CDCl_3$):

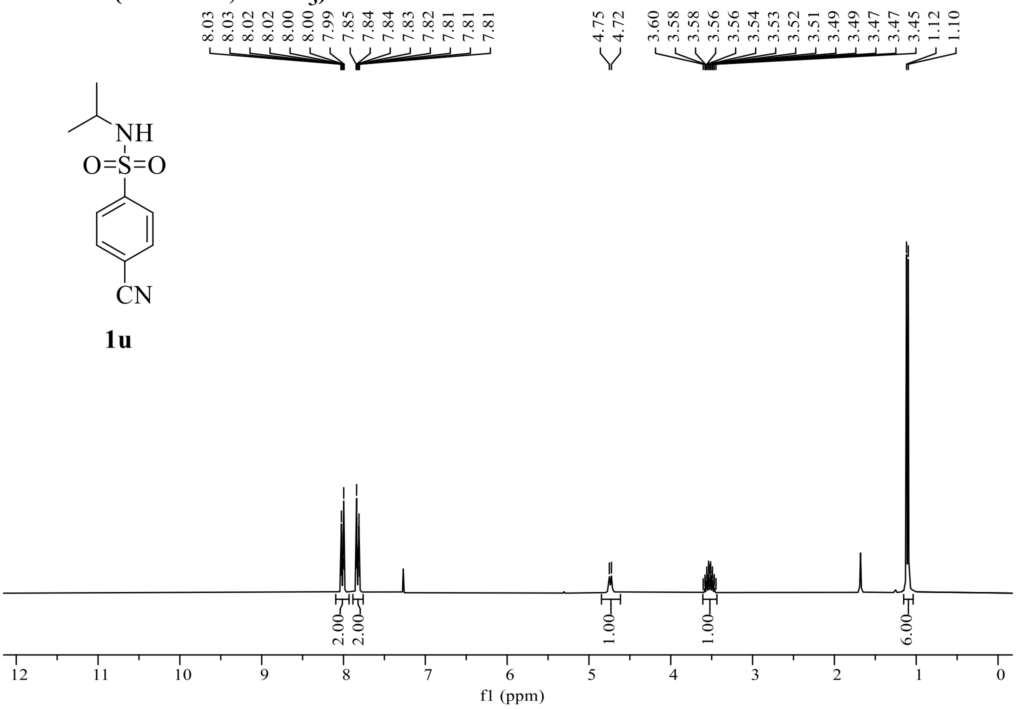

Supplementary Figure 45: ^{1}H NMR (300 MHz, $CDCl_3$) spectrum of compound **1u**.

^{13}C NMR (75 MHz, $CDCl_3$):

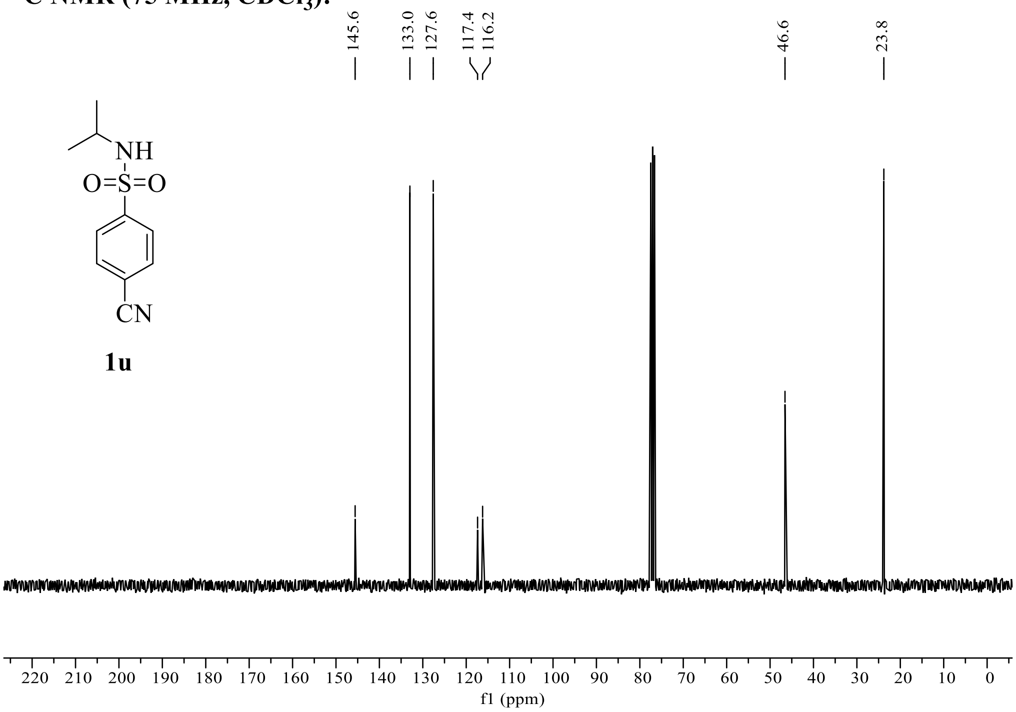

Supplementary Figure 46: ^{13}C NMR (75 MHz, $CDCl_3$) spectrum of compound **1u**.

^{1}H NMR (300 MHz, DMSO-d^{6}):

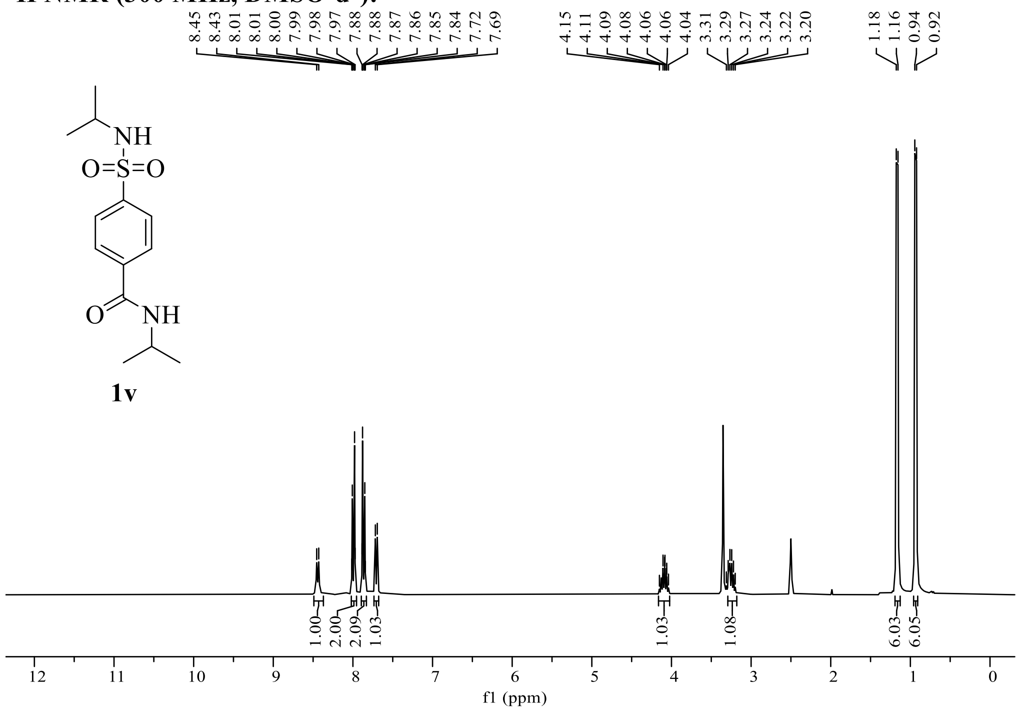

Supplementary Figure 47: ^{1}H NMR (300 MHz, DMSO-d^{6}) spectrum of compound **1v**.

^{13}C NMR (75 MHz, DMSO-d^{6}):

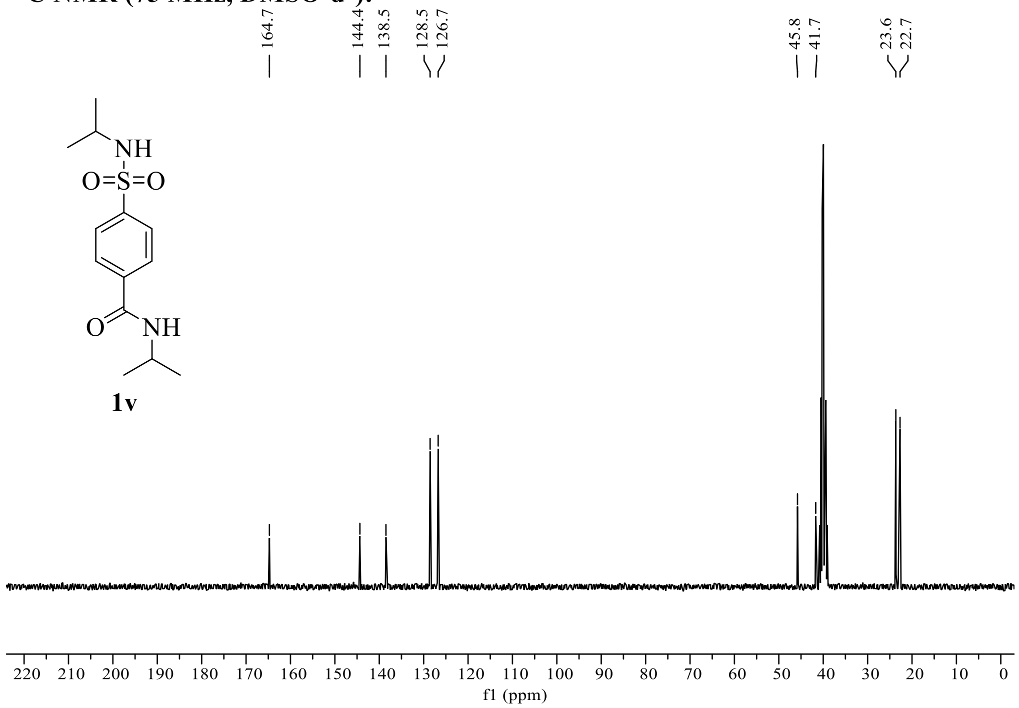

Supplementary Figure 48: ^{13}C NMR (75 MHz, DMSO-d^{6}) spectrum of compound **1v**.

^{1}H NMR (300 MHz, CDCl$_3$):

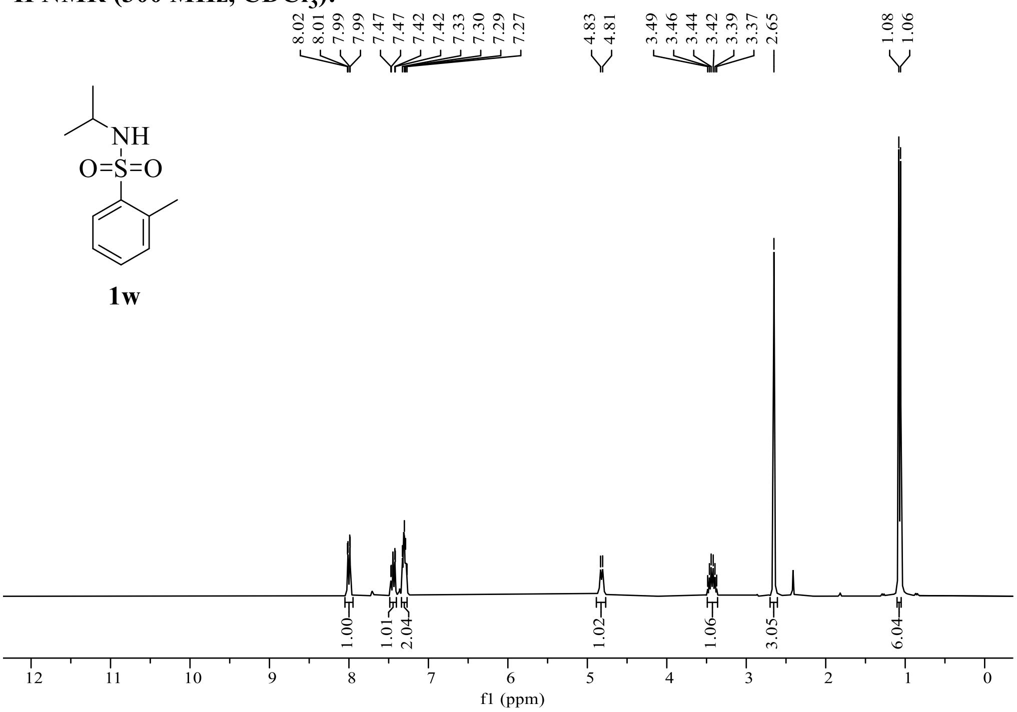

Supplementary Figure 49: ^{1}H NMR (300 MHz, CDCl$_3$) spectrum of compound **1w**.

^{13}C NMR (75 MHz, CDCl$_3$):

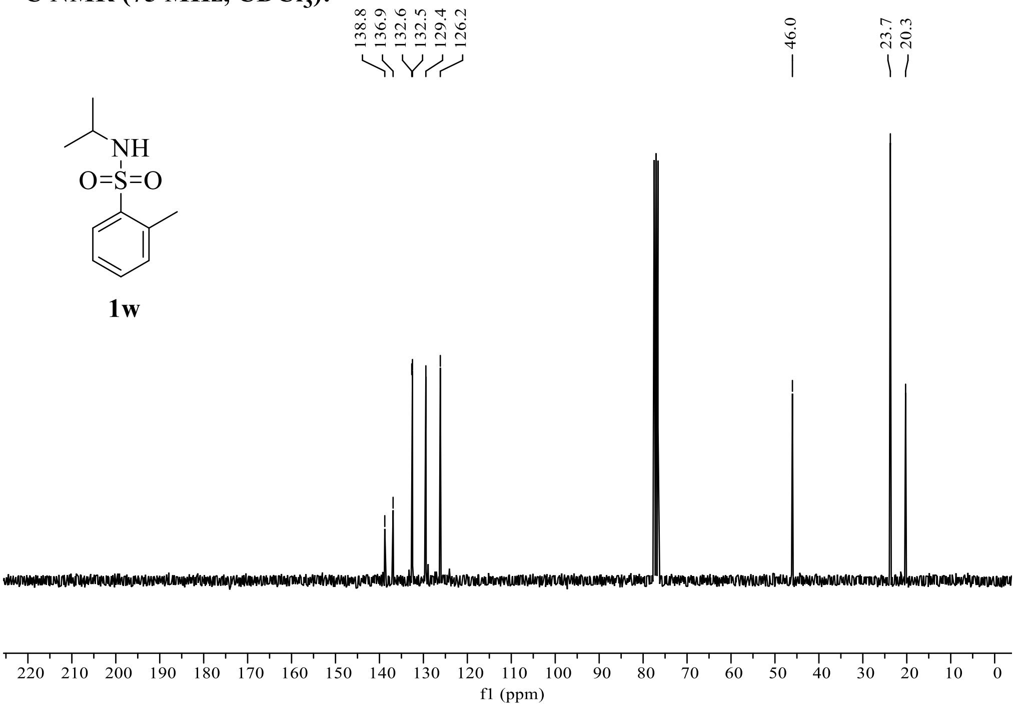

Supplementary Figure 50: ^{13}C NMR (75 MHz, CDCl$_3$) spectrum of compound **1w**.

^{1}H NMR (300 MHz, $CDCl_3$):

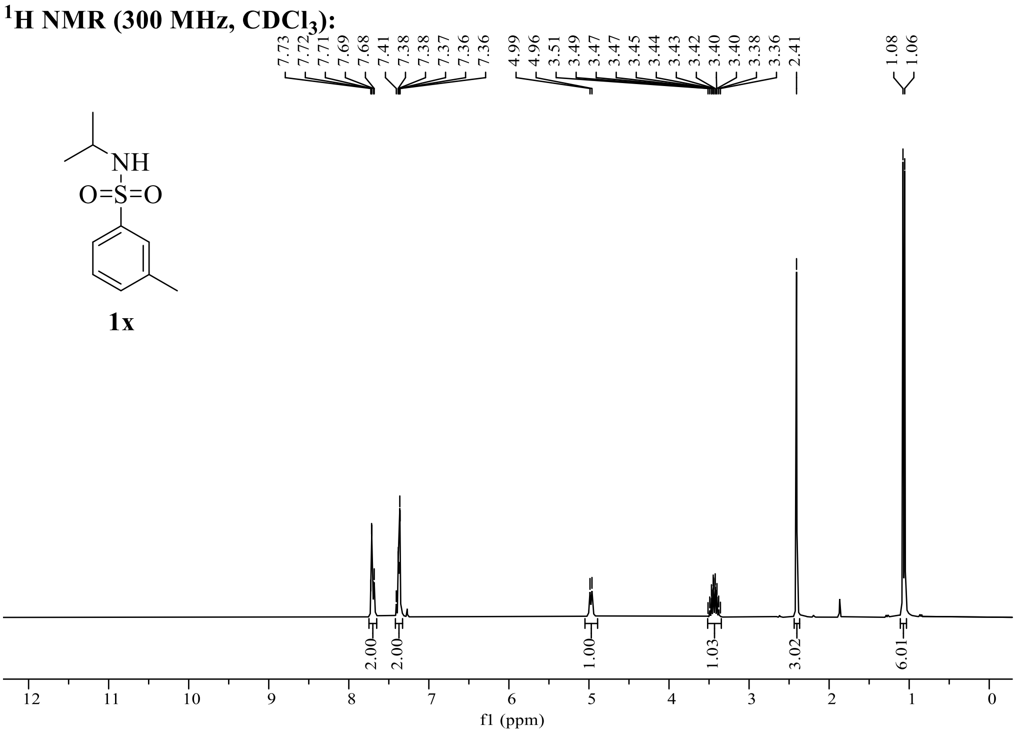

Supplementary Figure 51: ^{1}H NMR (300 MHz, $CDCl_3$) spectrum of compound **1x**.

^{13}C NMR (75 MHz, $CDCl_3$):

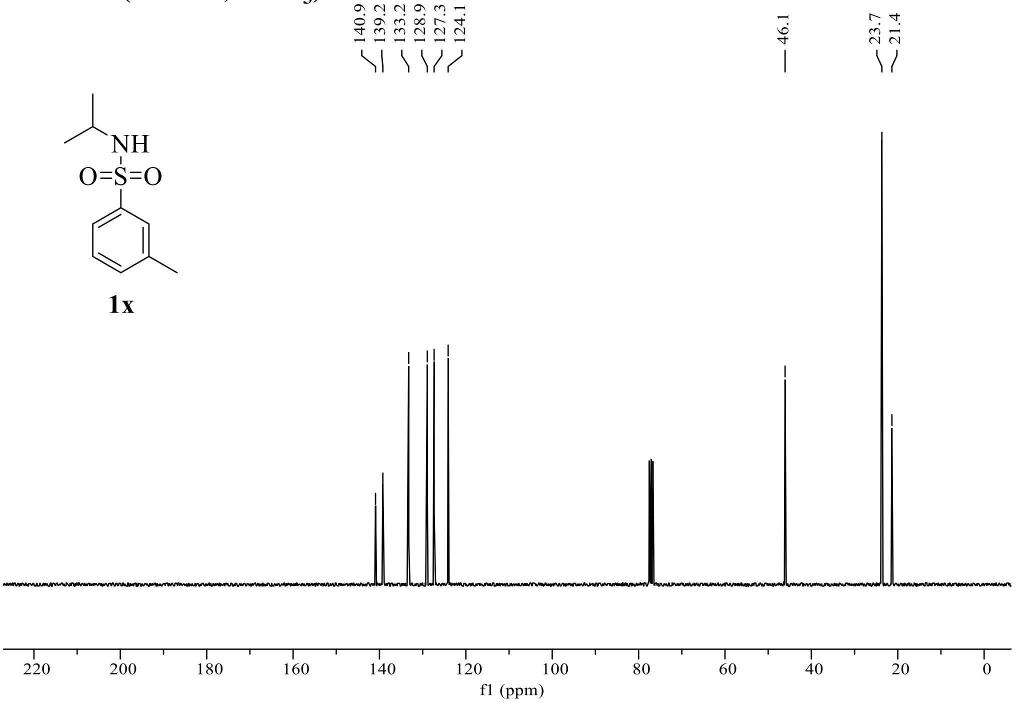

Supplementary Figure 52: ^{13}C NMR (75 MHz, $CDCl_3$) spectrum of compound **1x**.

^{1}H NMR (600 MHz, $CDCl_3$):

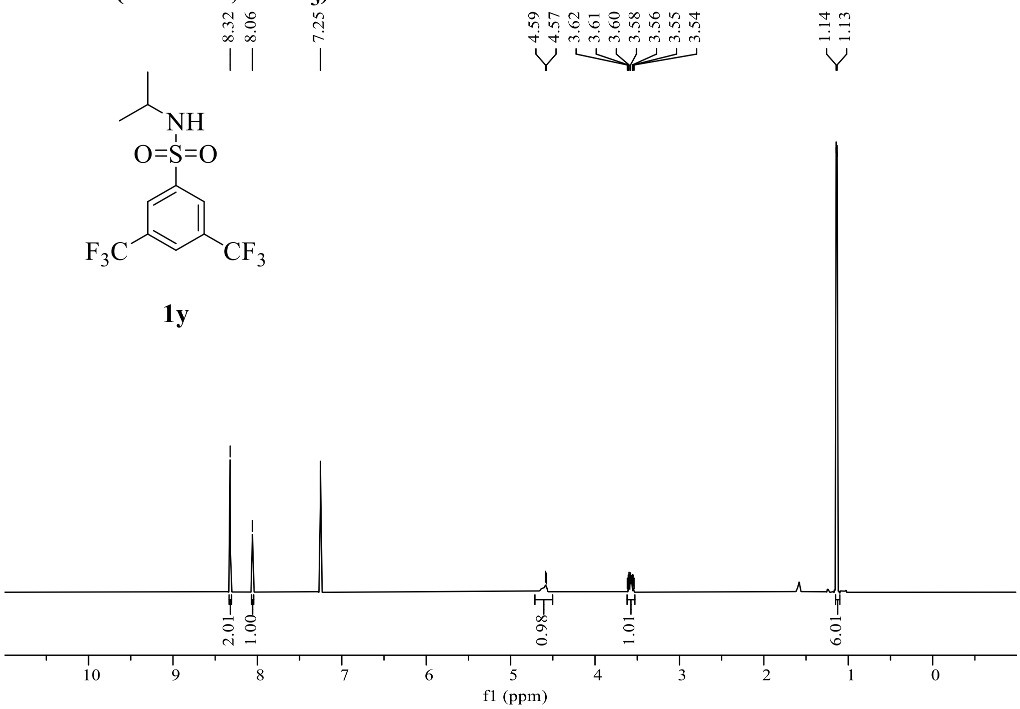

Supplementary Figure 53: ^{1}H NMR (600 MHz, $CDCl_3$) spectrum of compound **1y**.

$^{13}C\{^1H,^{19}F\}$ NMR (151 MHz, $CDCl_3$):

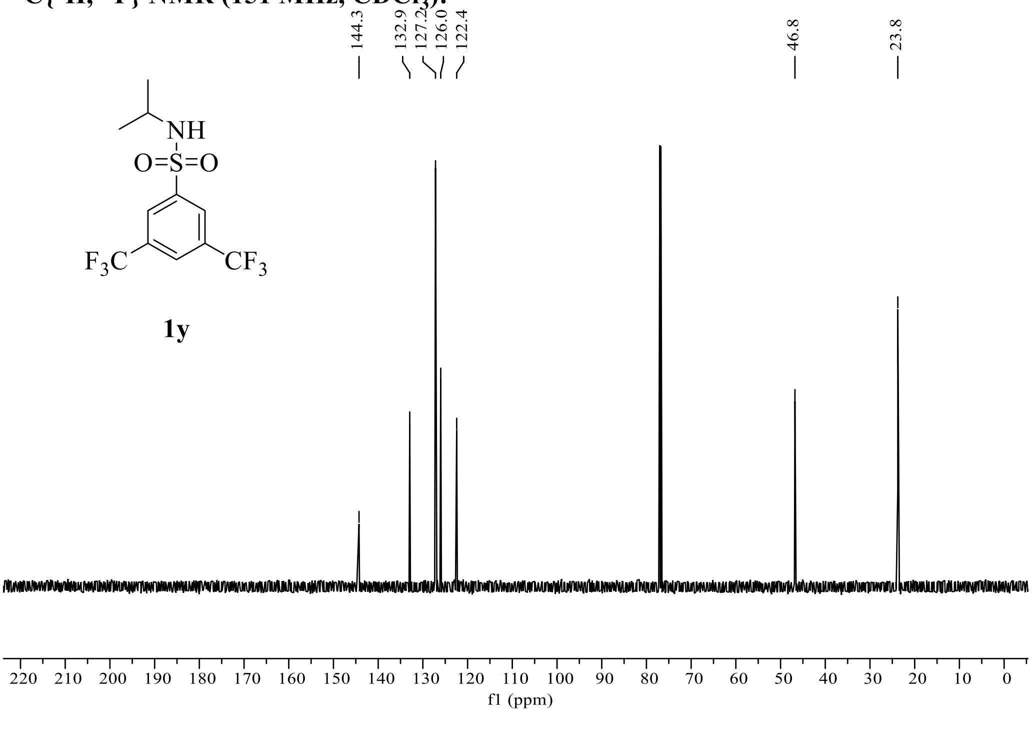

Supplementary Figure 54: $^{13}C\{^1H,^{19}F\}$ NMR (151 MHz, $CDCl_3$) spectrum of compound **1y**.

$^{19}F\{^{1}H,^{13}C\}$ NMR (564 MHz, $CDCl_3$):

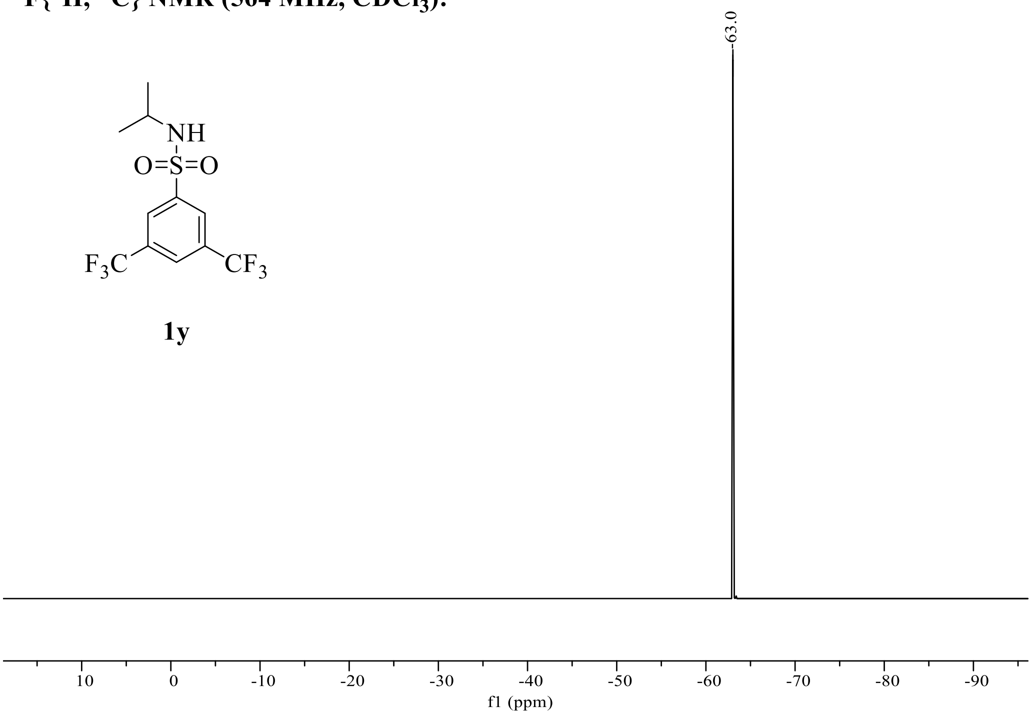

Supplementary Figure 55: $^{19}F\{^{1}H,^{13}C\}$ NMR (564 MHz, $CDCl_3$) spectrum of compound **1y**.

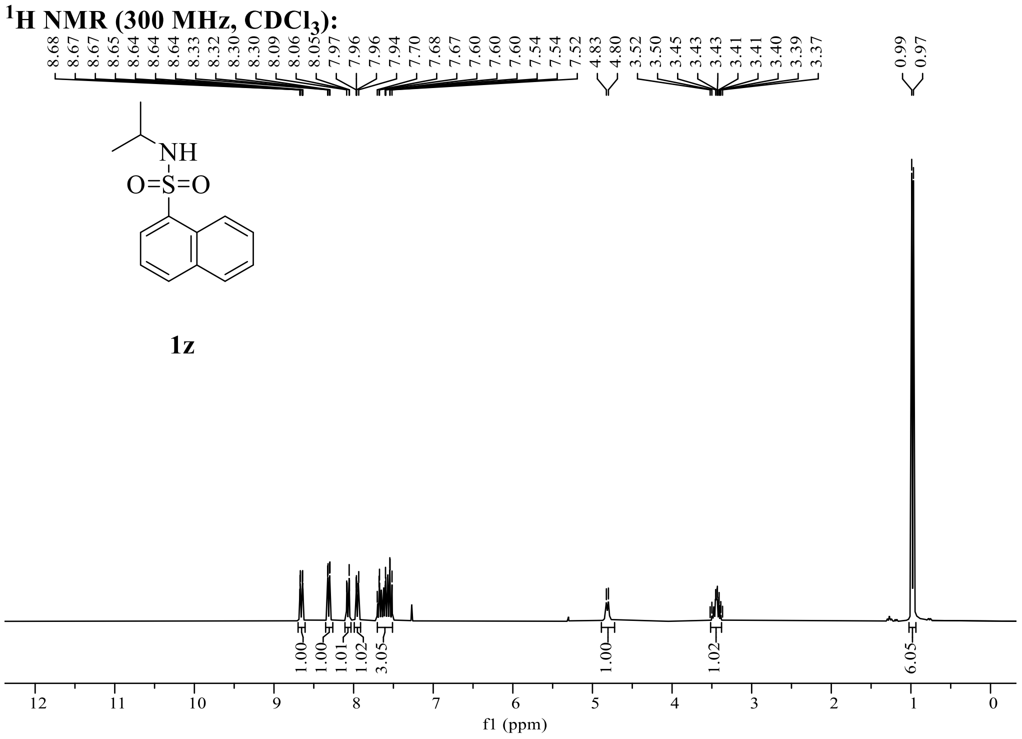

Supplementary Figure 56: ^{1}H NMR (300 MHz, $CDCl_3$) spectrum of compound **1z**.

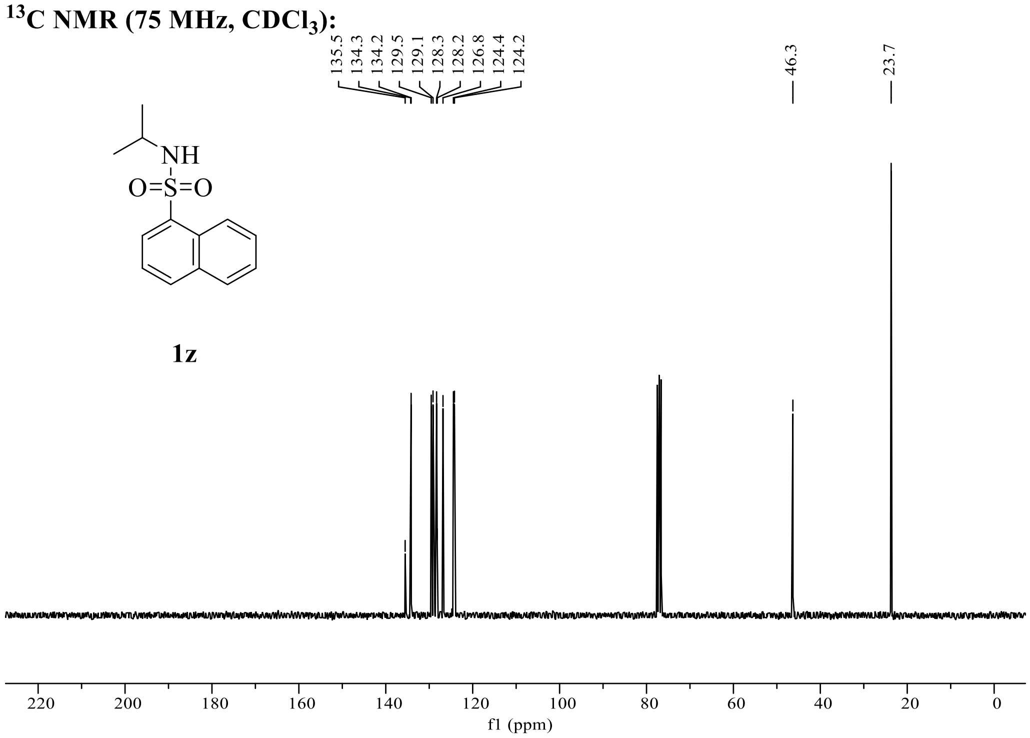

Supplementary Figure 57: ^{13}C NMR (75 MHz, $CDCl_3$) spectrum of compound **1z**.

^{1}H NMR (300 MHz, $CDCl_3$):

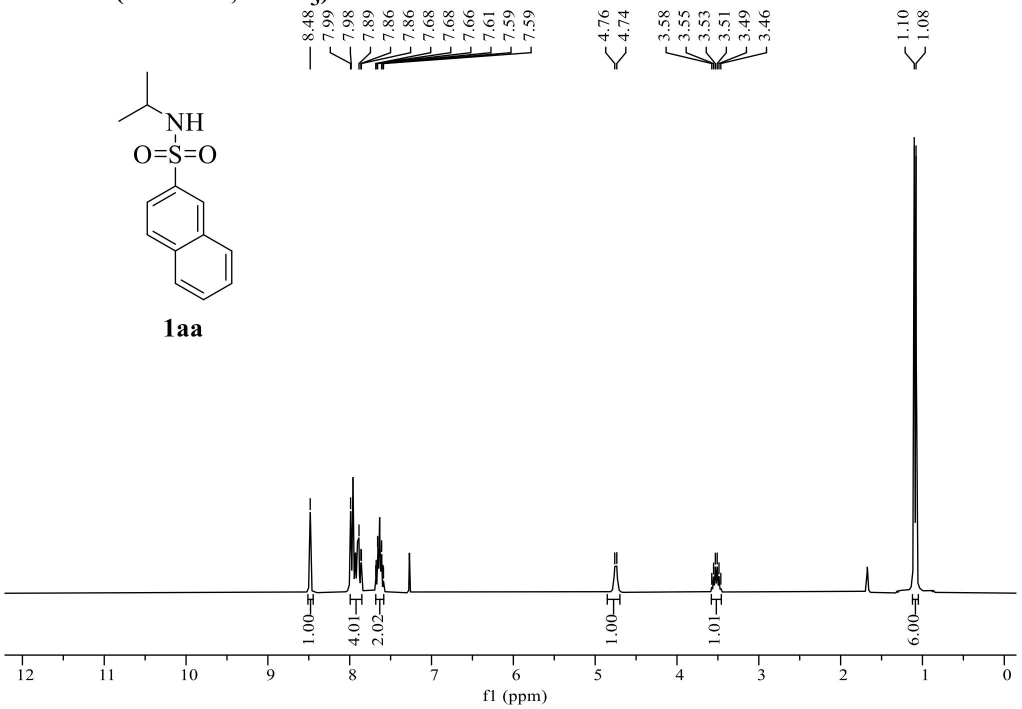

Supplementary Figure 58: ^{1}H NMR (300 MHz, $CDCl_3$) spectrum of compound **1aa**.

^{13}C NMR (75 MHz, $CDCl_3$):

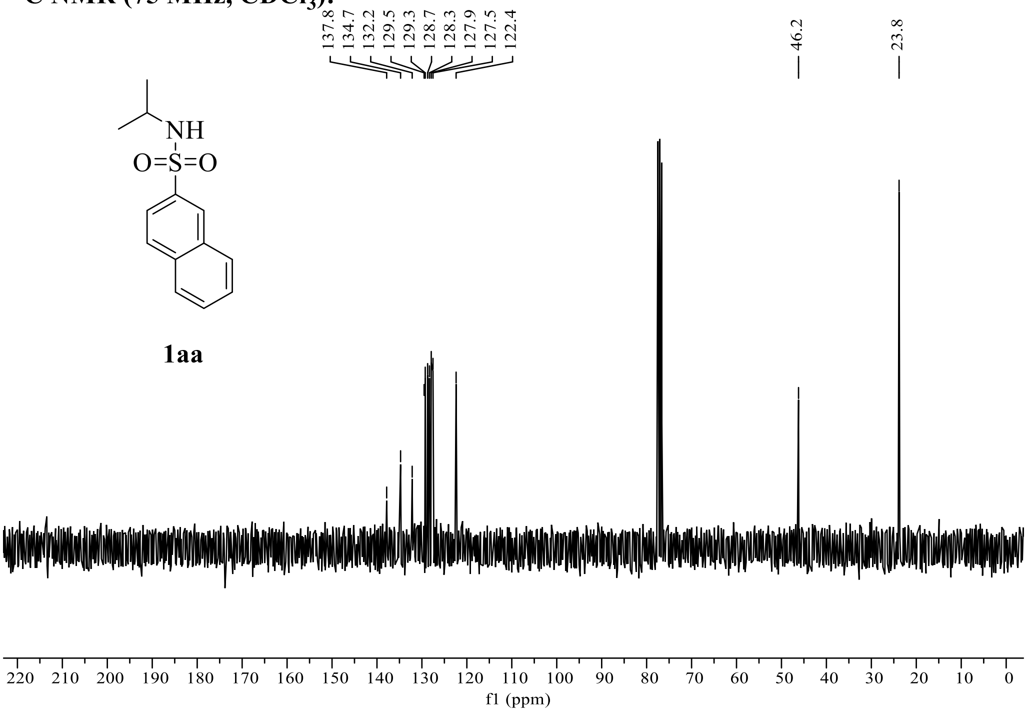

Supplementary Figure 59: ^{13}C NMR (75 MHz, $CDCl_3$) spectrum of compound **1aa**.

^{1}H NMR (300 MHz, $CDCl_3$):

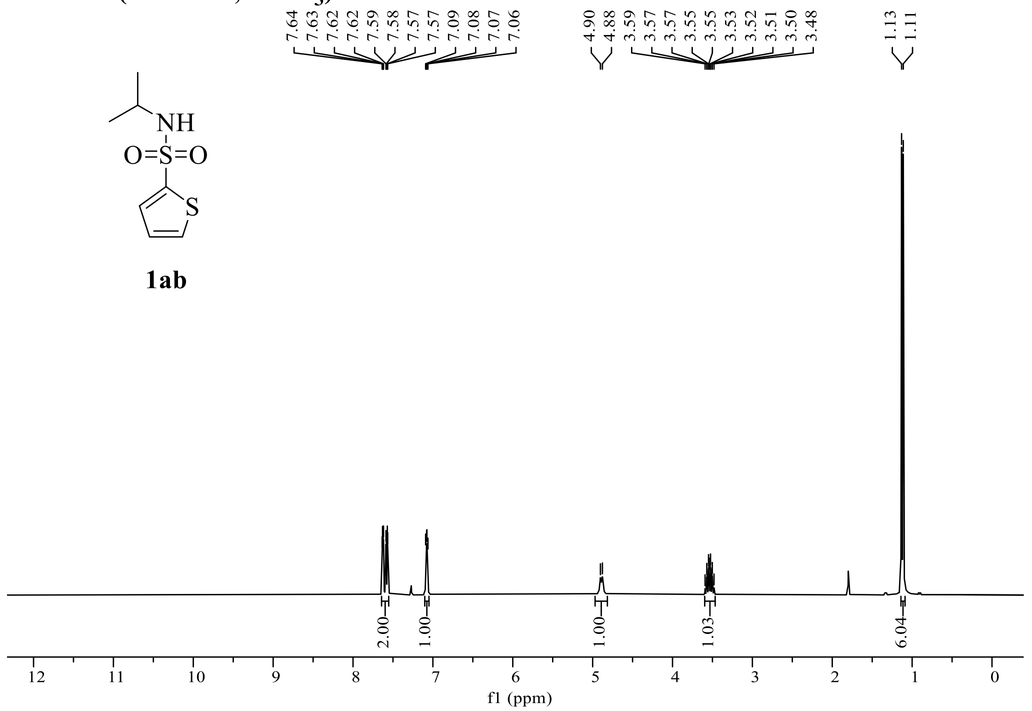

Supplementary Figure 60: ^{1}H NMR (300 MHz, $CDCl_3$) spectrum of compound **1ab**.

^{13}C NMR (75 MHz, $CDCl_3$):

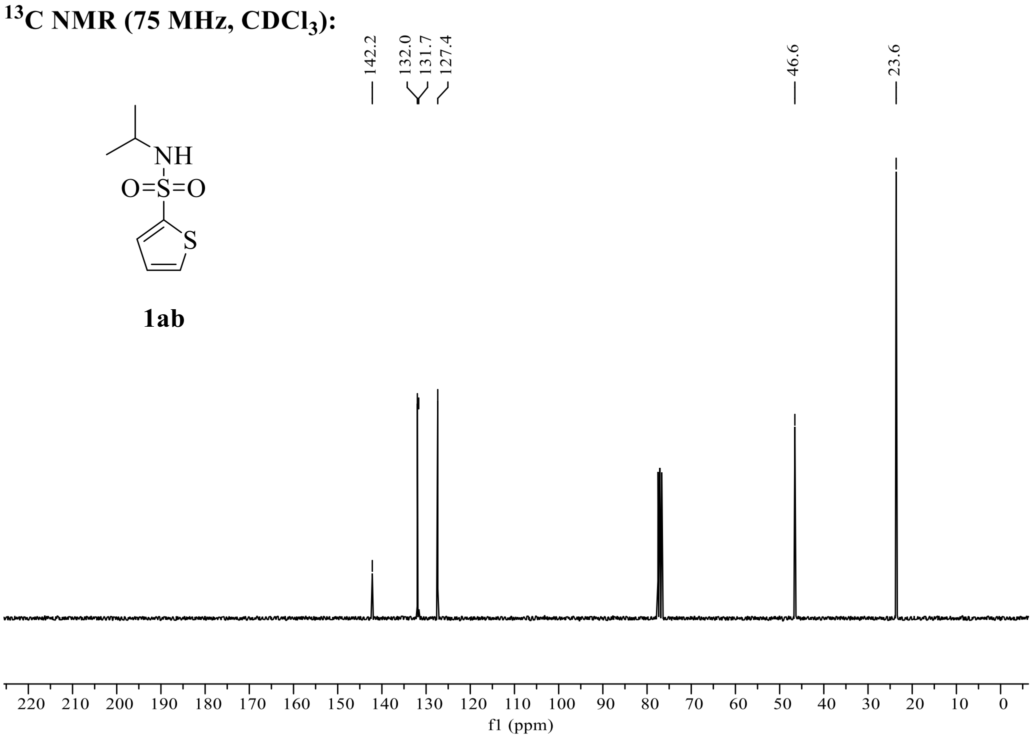

Supplementary Figure 61: ^{13}C NMR (75 MHz, $CDCl_3$) spectrum of compound **1ab**.

^{1}H NMR (300 MHz, $CDCl_3$):

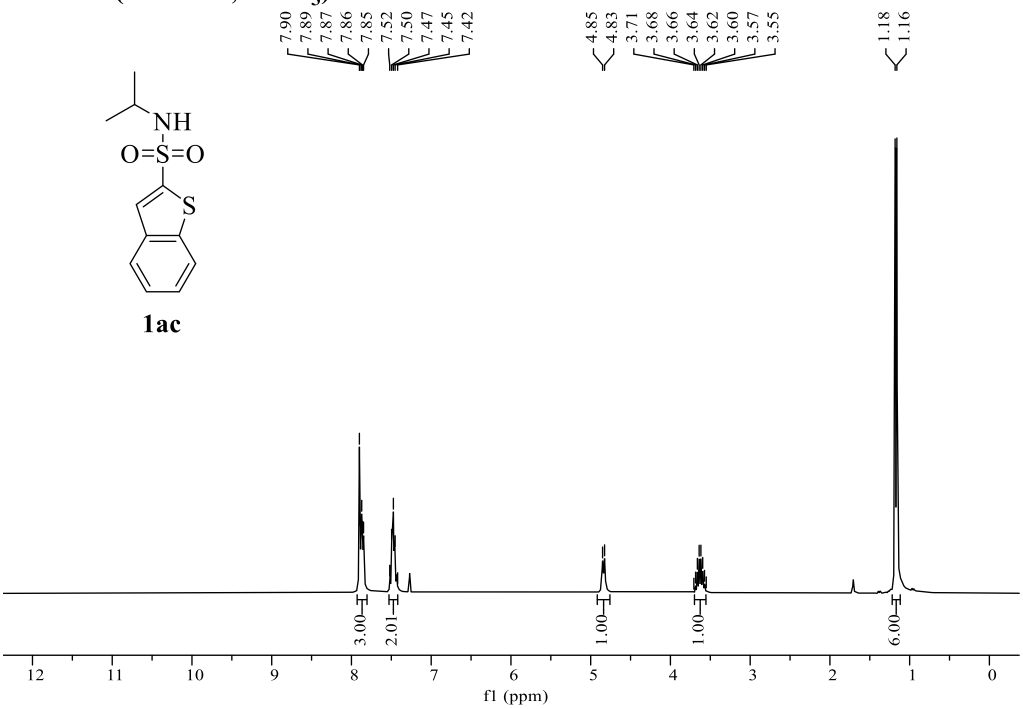

Supplementary Figure 62: ^{1}H NMR (300 MHz, $CDCl_3$) spectrum of compound **1ac**.

^{13}C NMR (75 MHz, $CDCl_3$):

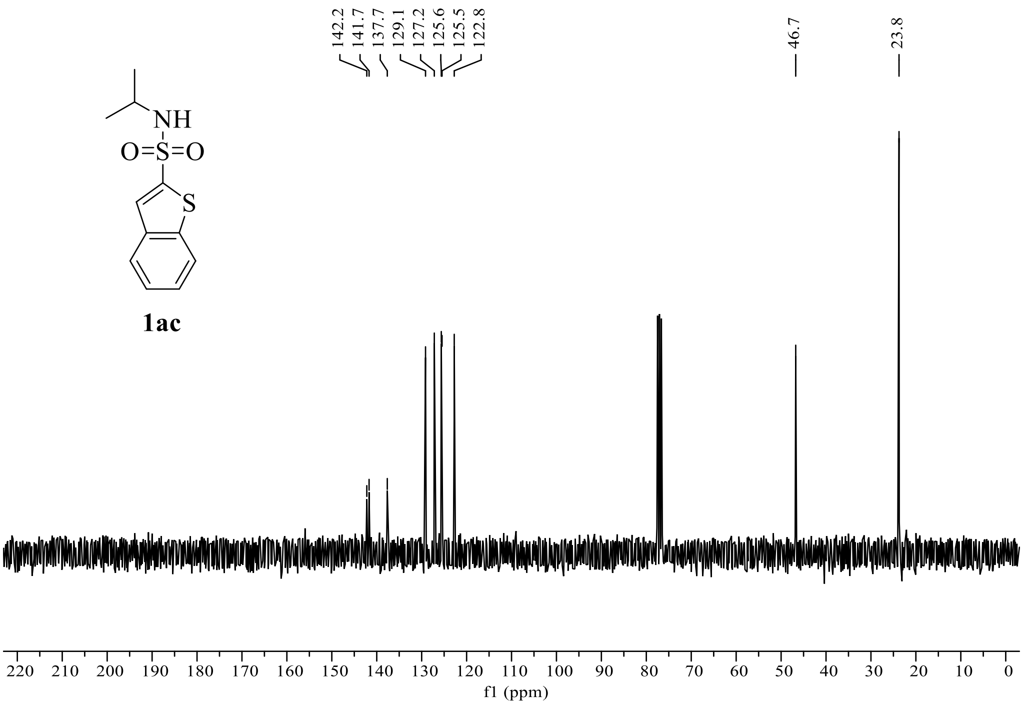

Supplementary Figure 63: ^{13}C NMR (75 MHz, $CDCl_3$) spectrum of compound **1ac**.

^{1}H NMR (300 MHz, $CDCl_3$):

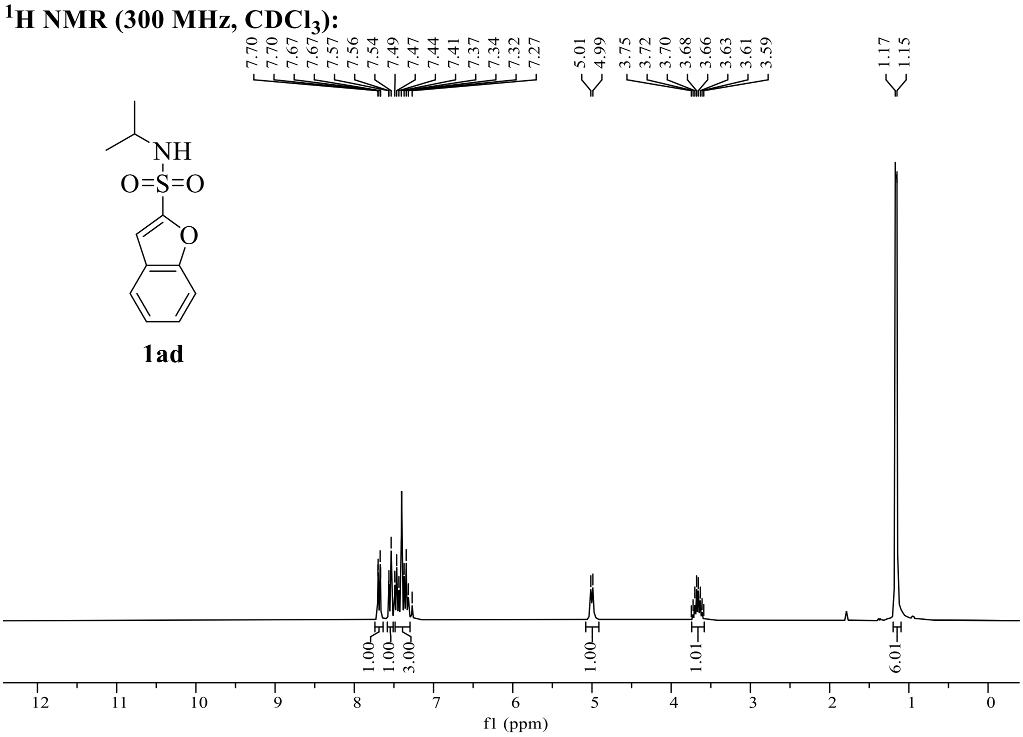

Supplementary Figure 64: ^{1}H NMR (300 MHz, $CDCl_3$) spectrum of compound **1ad**.

^{13}C NMR (75 MHz, $CDCl_3$):

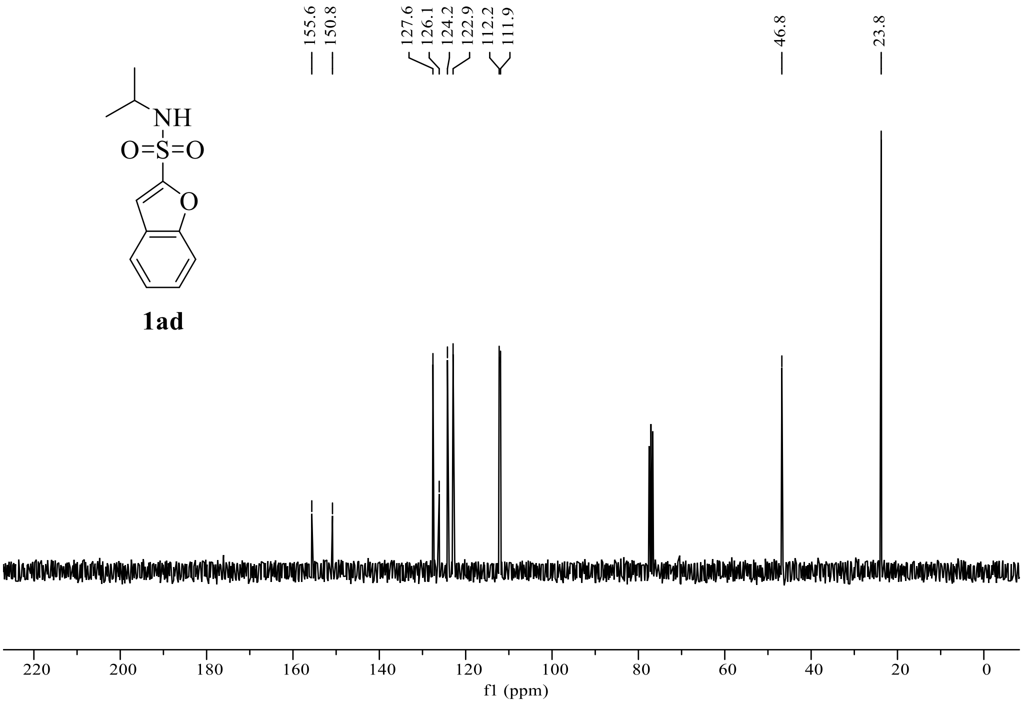

Supplementary Figure 65: ^{13}C NMR (75 MHz, $CDCl_3$) spectrum of compound **1ad**.

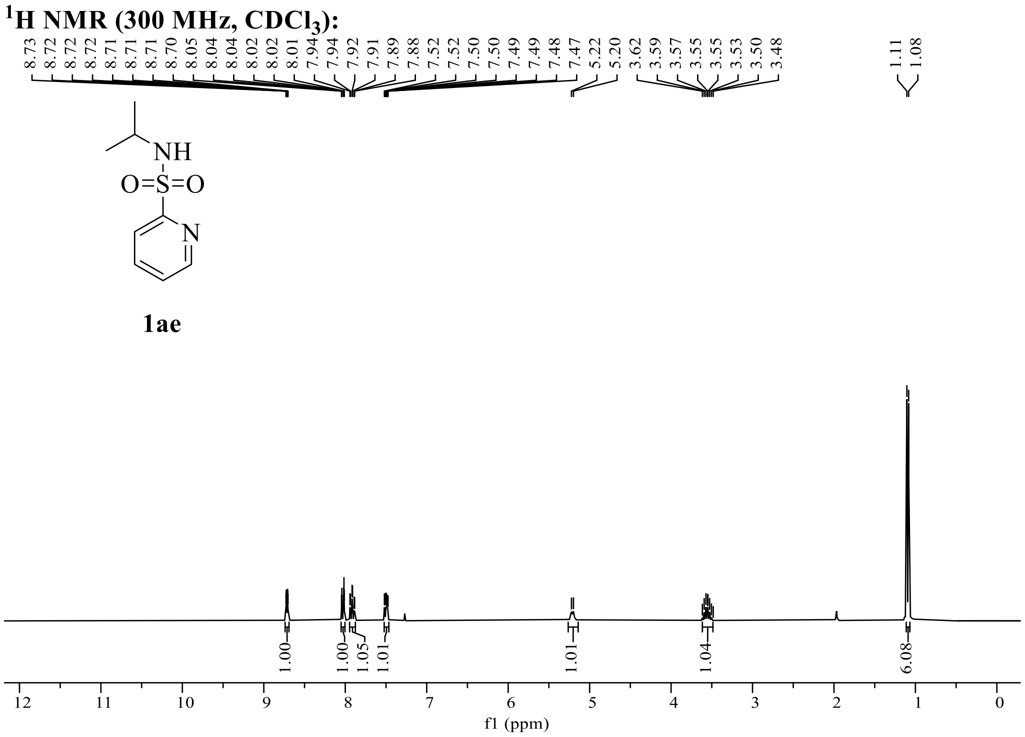

Supplementary Figure 66: ^{1}H NMR (300 MHz, $CDCl_3$) spectrum of compound **1ae**.

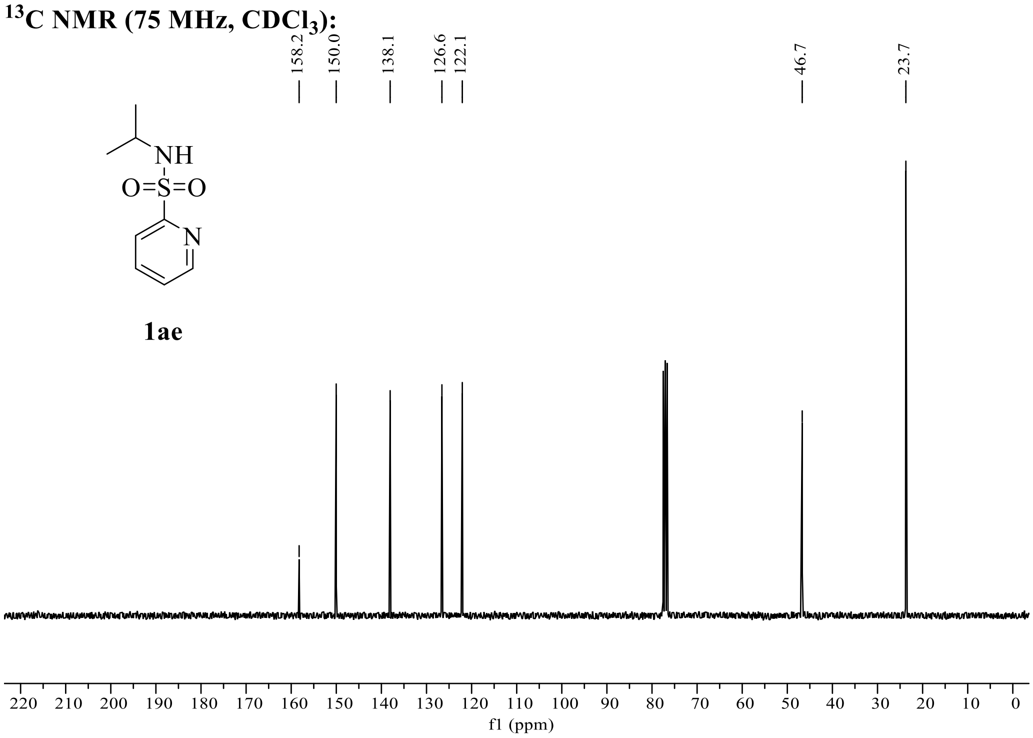

Supplementary Figure 67: ^{13}C NMR (75 MHz, $CDCl_3$) spectrum of compound **1ae**.

^{1}H NMR (300 MHz, $CDCl_3$):

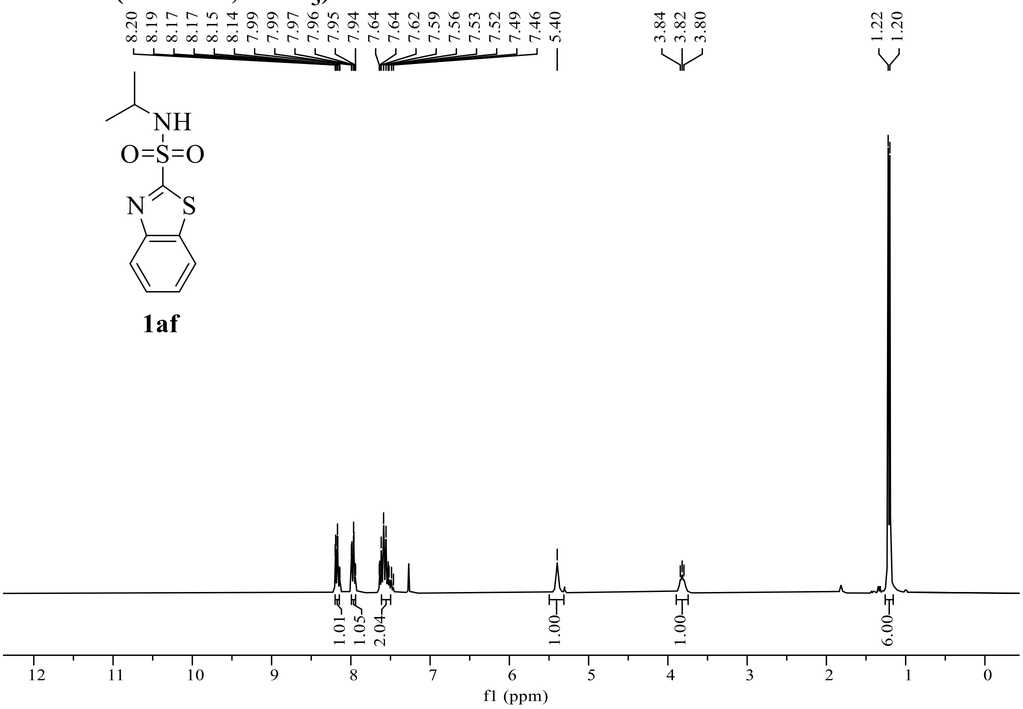

Supplementary Figure 68: ^{1}H NMR (300 MHz, $CDCl_3$) spectrum of compound **1af**.

^{13}C NMR (75 MHz, $CDCl_3$):

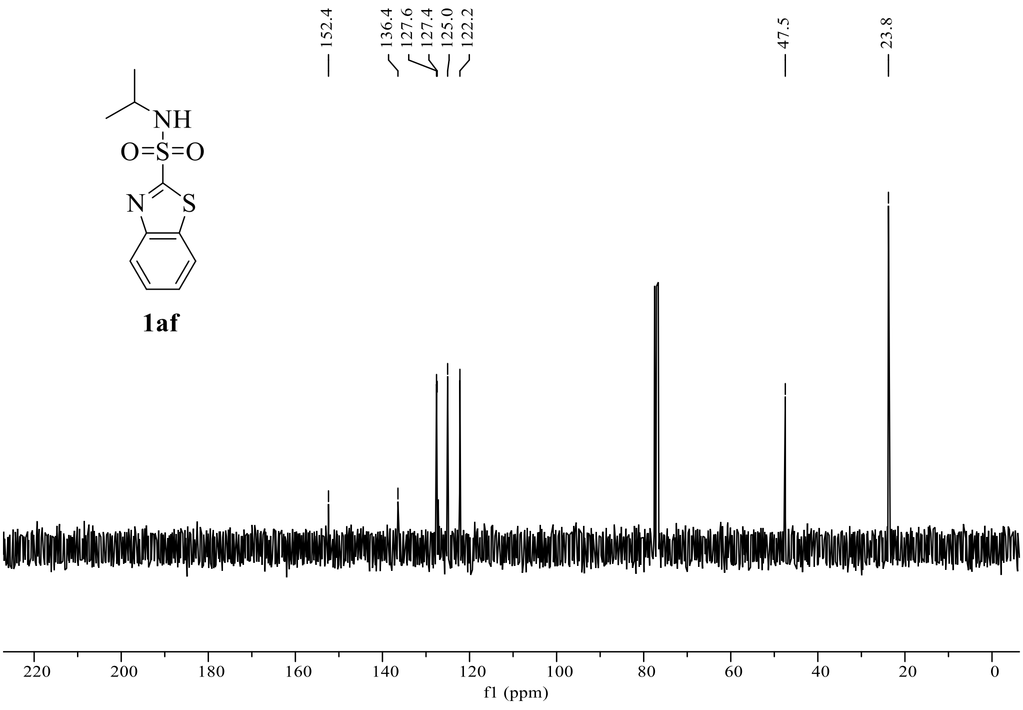

Supplementary Figure 69: ^{13}C NMR (75 MHz, $CDCl_3$) spectrum of compound **1af**.

^{1}H NMR (300 MHz, CDCl$_3$):

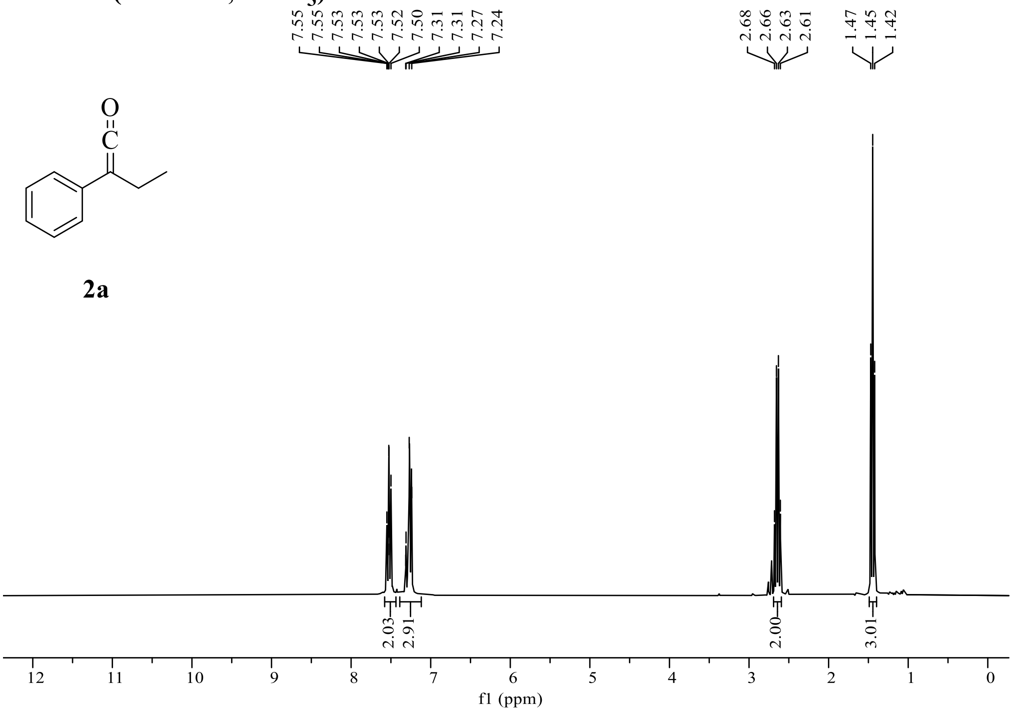

Supplementary Figure 70: ^{1}H NMR (300 MHz, CDCl$_3$) spectrum of compound **2a**.

^{13}C NMR (75 MHz, CDCl$_3$):

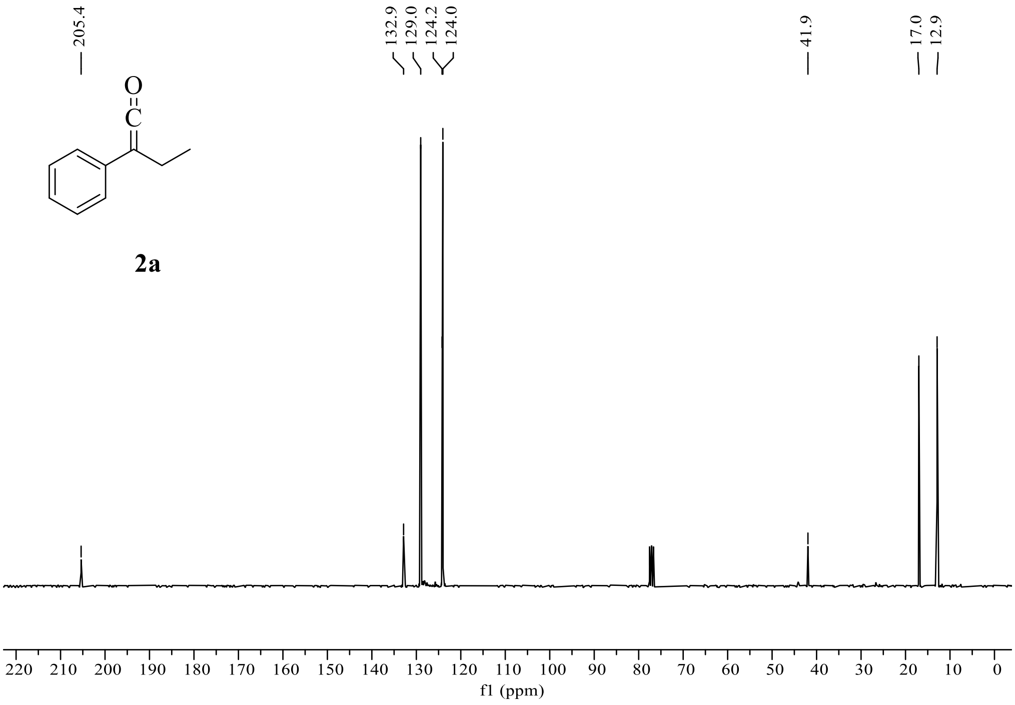

Supplementary Figure 71: ^{13}C NMR (75 MHz, CDCl$_3$) spectrum of compound **2a**.

^{1}H NMR (300 MHz, CDCl$_3$):

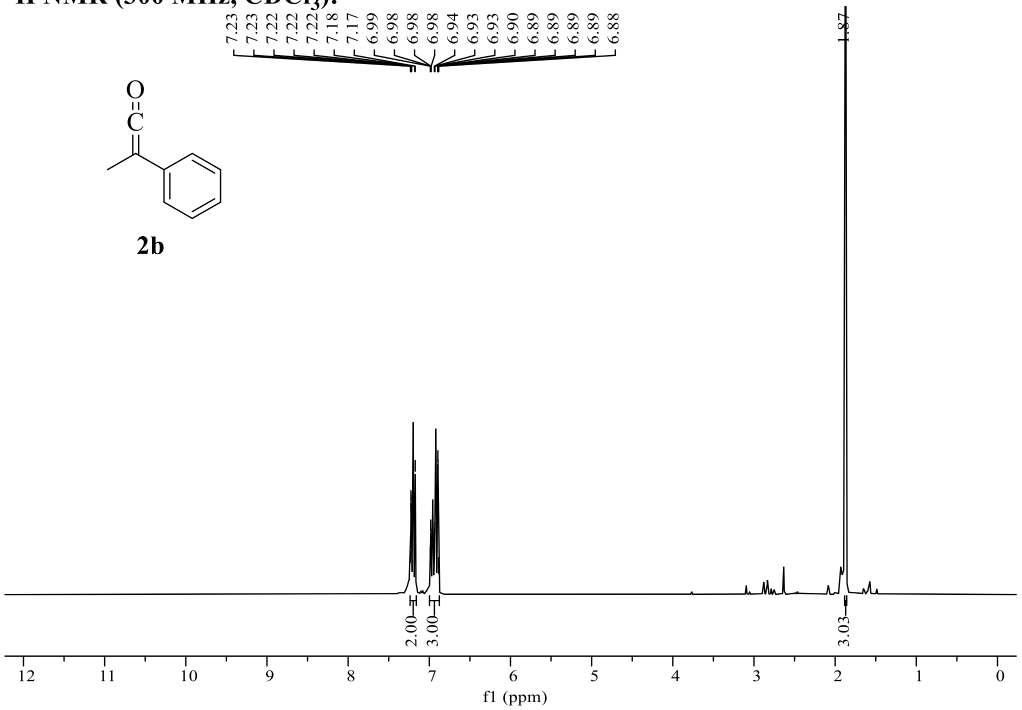

Supplementary Figure 72: ^{1}H NMR (300 MHz, CDCl$_3$) spectrum of compound **2b**.

^{13}C NMR (75 MHz, CDCl$_3$):

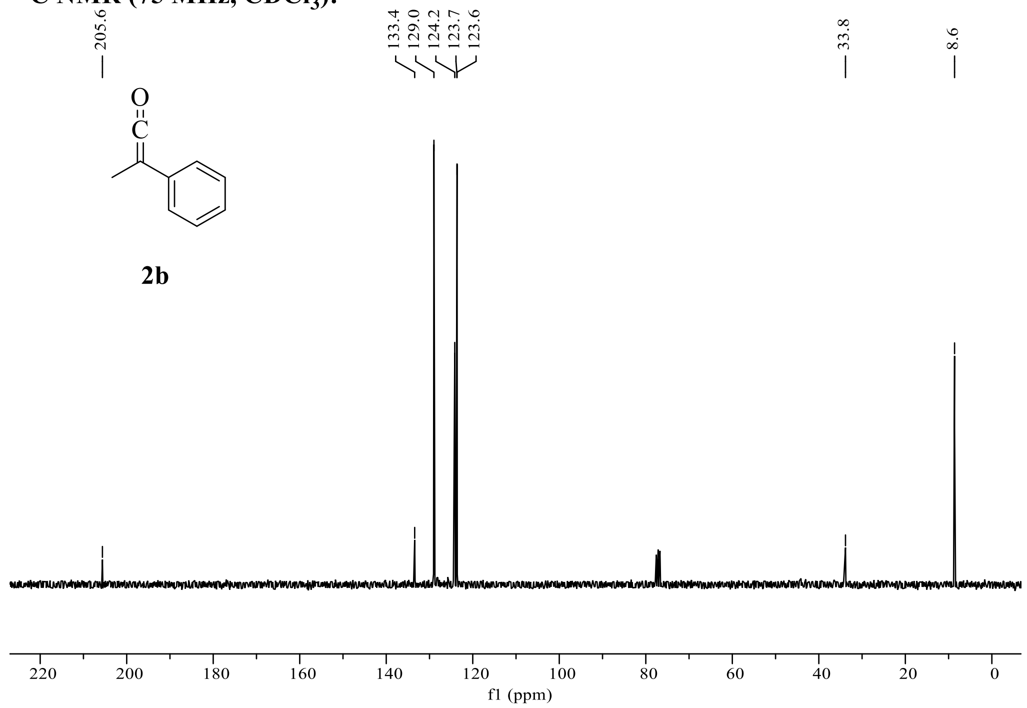

Supplementary Figure 73: ^{13}C NMR (75 MHz, CDCl$_3$) spectrum of compound **2b**.

^{1}H NMR (300 MHz, $CDCl_3$):

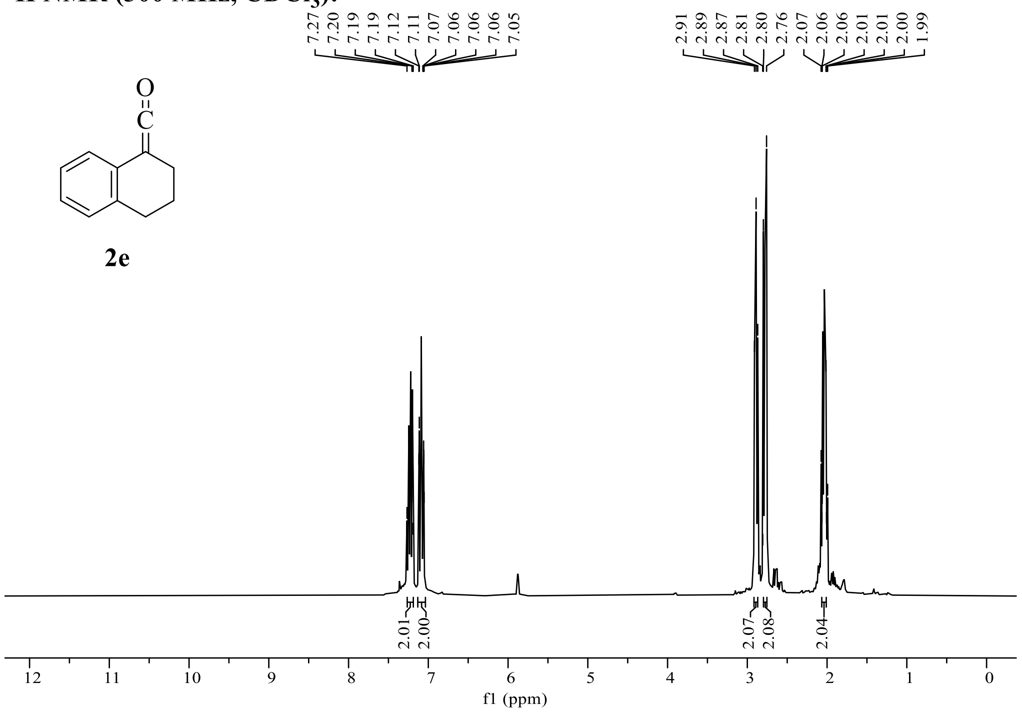

Supplementary Figure 74: ^{1}H NMR (300 MHz, $CDCl_3$) spectrum of compound **2e**.

^{13}C NMR (75 MHz, $CDCl_3$):

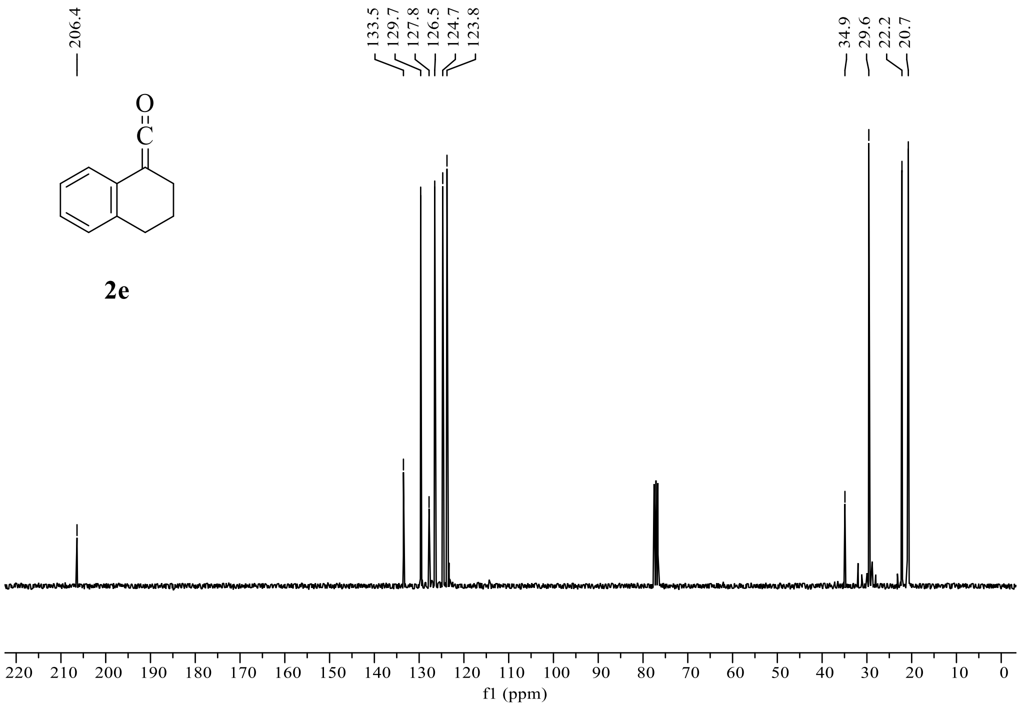

Supplementary Figure 75: ^{13}C NMR (75 MHz, $CDCl_3$) spectrum of compound **2e**.

^{1}H NMR (300 MHz, $CDCl_3$):

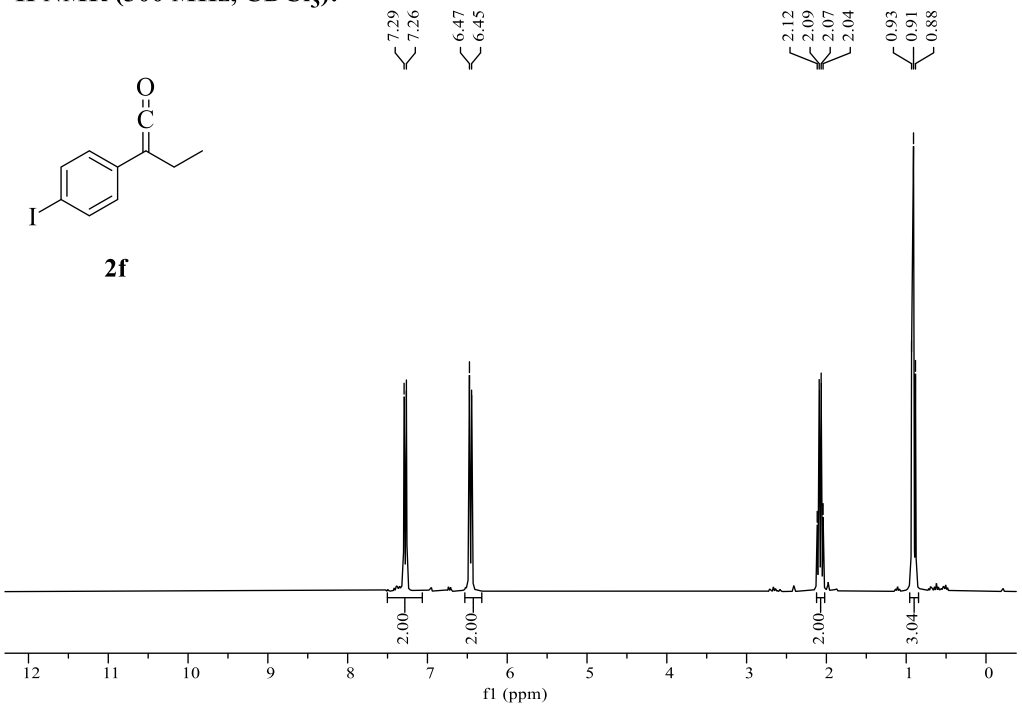

Supplementary Figure 76: ^{1}H NMR (300 MHz, $CDCl_3$) spectrum of compound **2f**.

^{13}C NMR (75 MHz, $CDCl_3$):

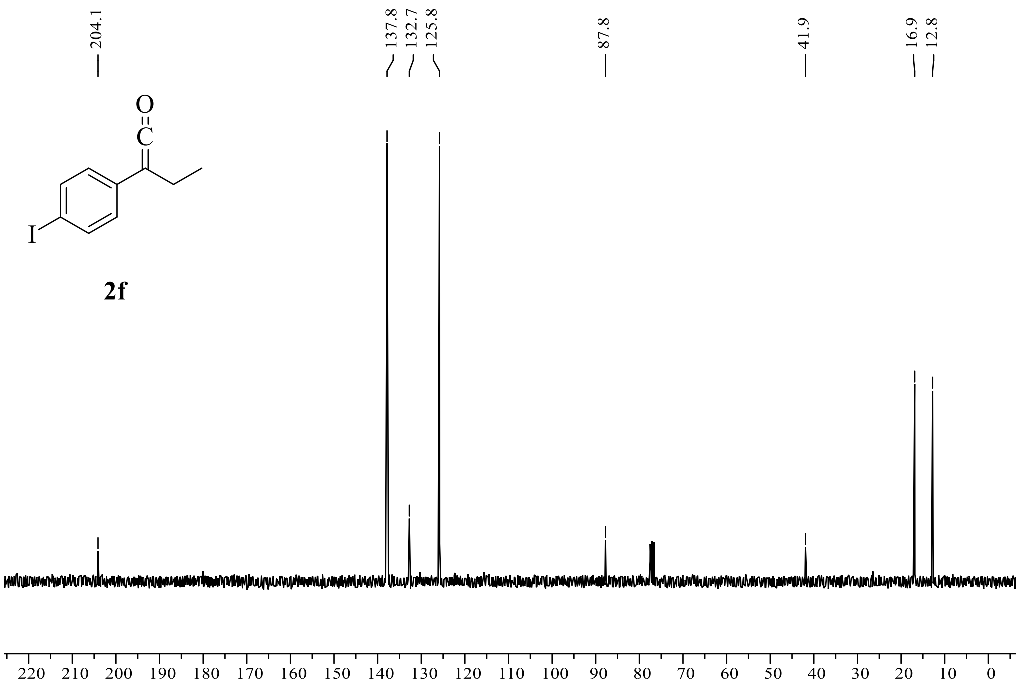

Supplementary Figure 77: ^{13}C NMR (75 MHz, $CDCl_3$) spectrum of compound **2f**.

^{1}H NMR (300 MHz, $CDCl_3$):

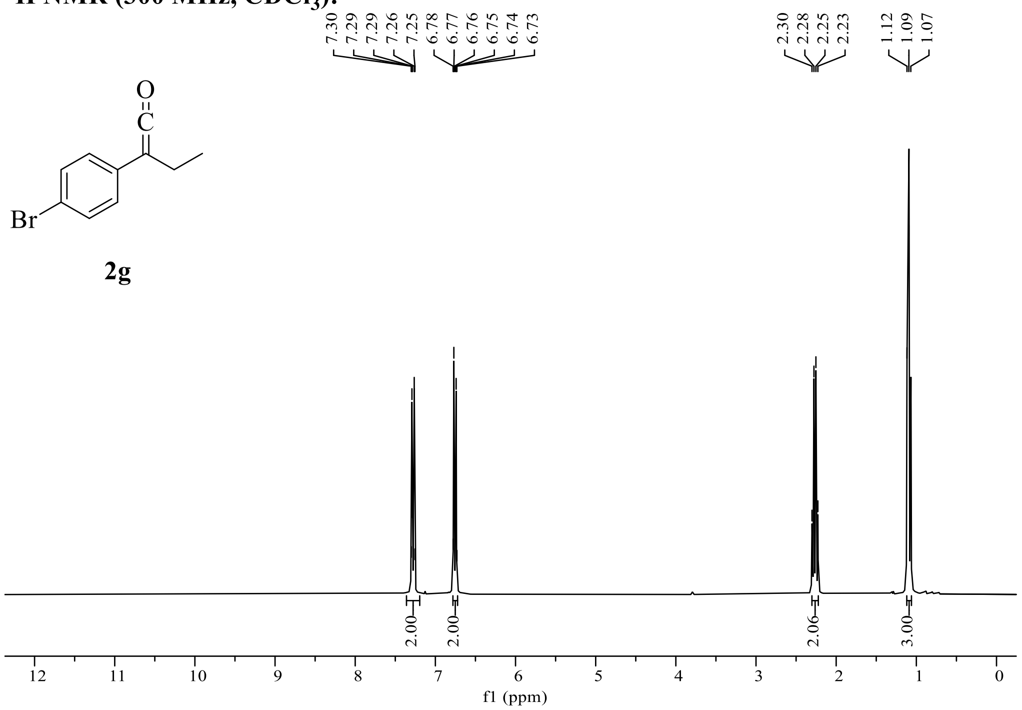

Supplementary Figure 78: ^{1}H NMR (300 MHz, $CDCl_3$) spectrum of compound **2g**.

^{13}C NMR (75 MHz, $CDCl_3$):

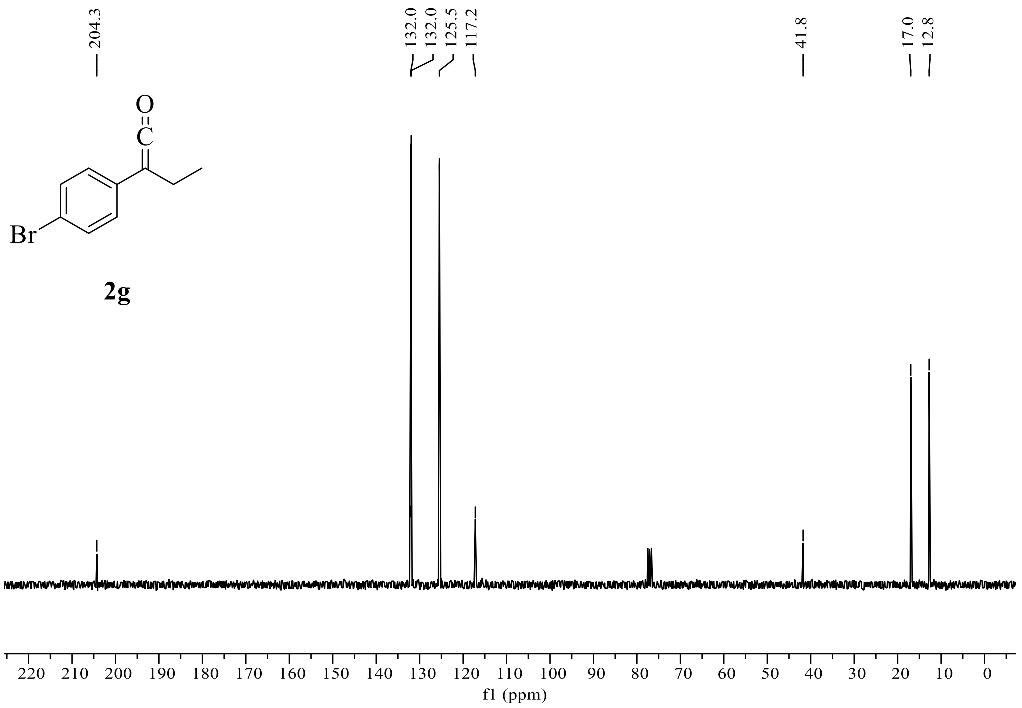

Supplementary Figure 79: ^{13}C NMR (75 MHz, $CDCl_3$) spectrum of compound **2g**.

^{1}H NMR (300 MHz, $CDCl_3$):

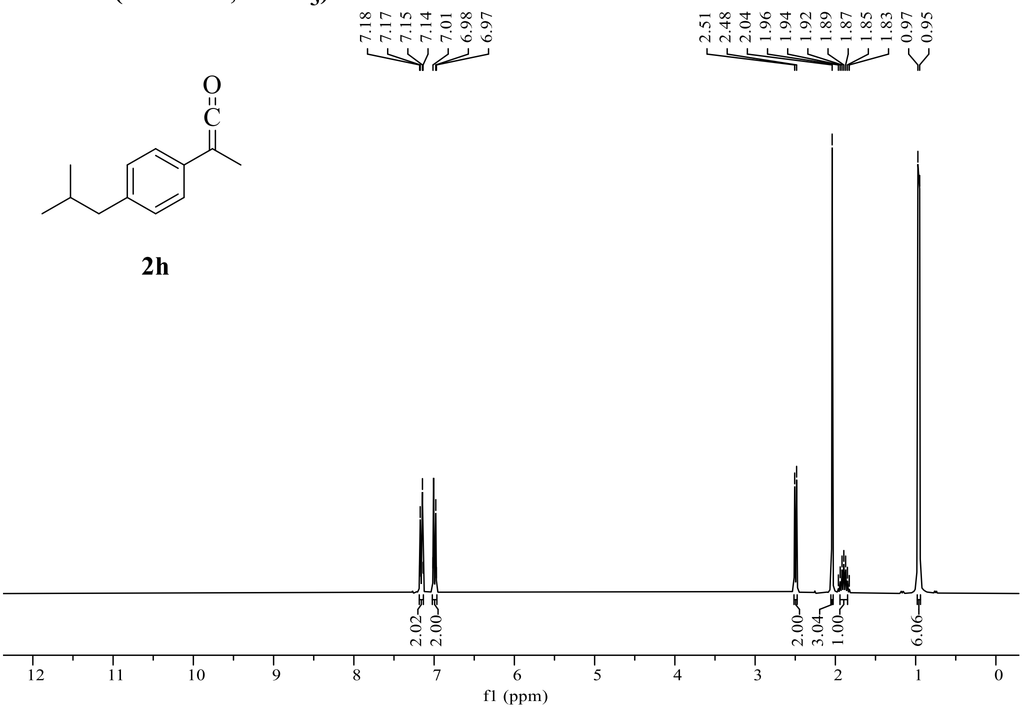

Supplementary Figure 80: ^{1}H NMR (300 MHz, $CDCl_3$) spectrum of compound **2h**.

^{13}C NMR (75 MHz, $CDCl_3$):

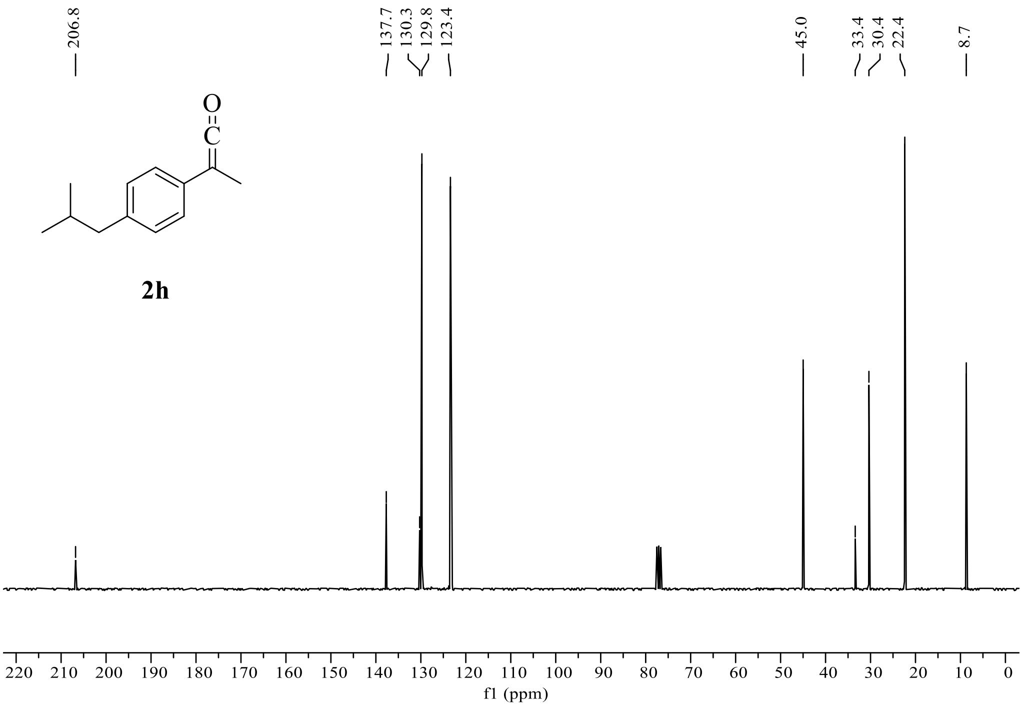

Supplementary Figure 81: ^{13}C NMR (75 MHz, $CDCl_3$) spectrum of compound **2h**.

^{1}H NMR (300 MHz, $CDCl_3$):

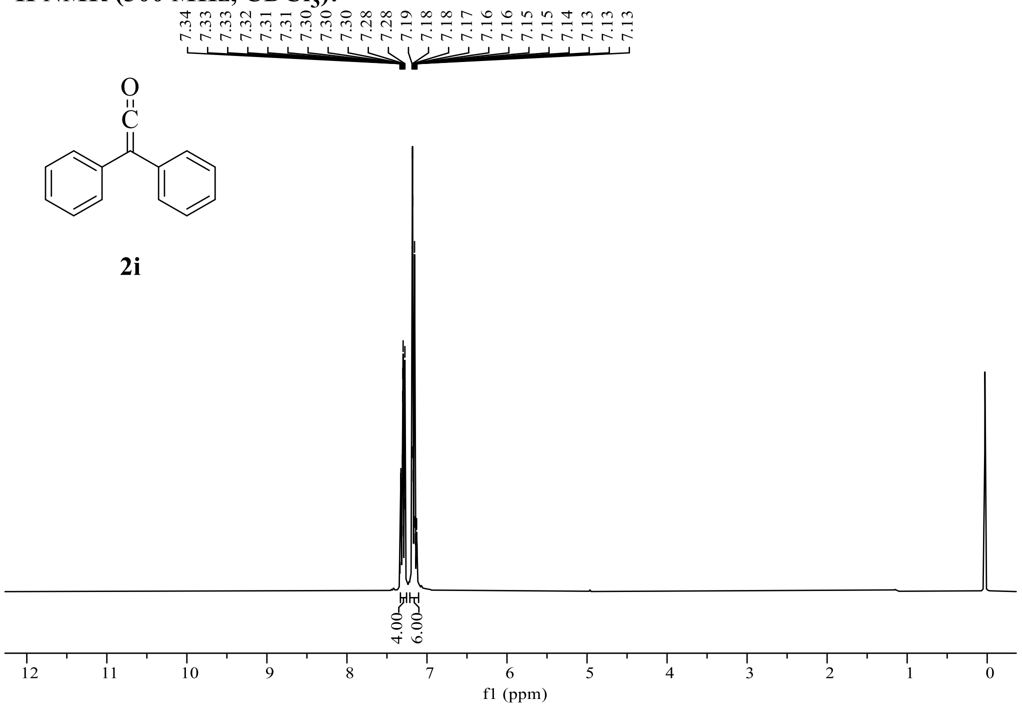

Supplementary Figure 82: ^{1}H NMR (300 MHz, $CDCl_3$) spectrum of compound **2i**.

^{13}C NMR (75 MHz, $CDCl_3$):

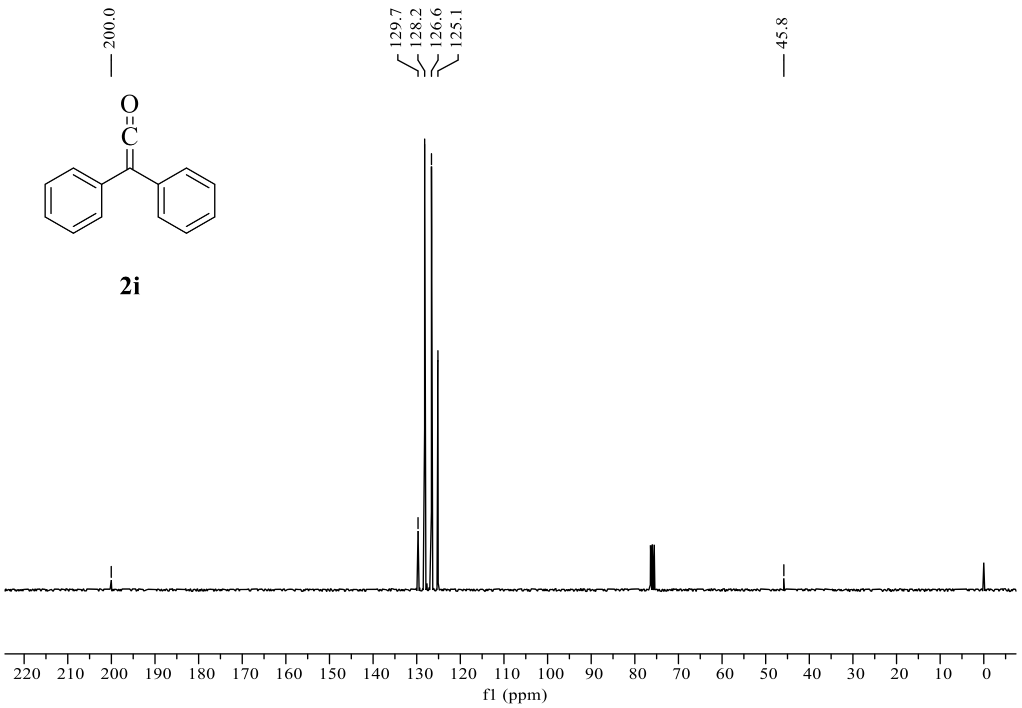

Supplementary Figure 83: ^{13}C NMR (75 MHz, $CDCl_3$) spectrum of compound **2i**.

¹H NMR (300 MHz, $CDCl_3$):

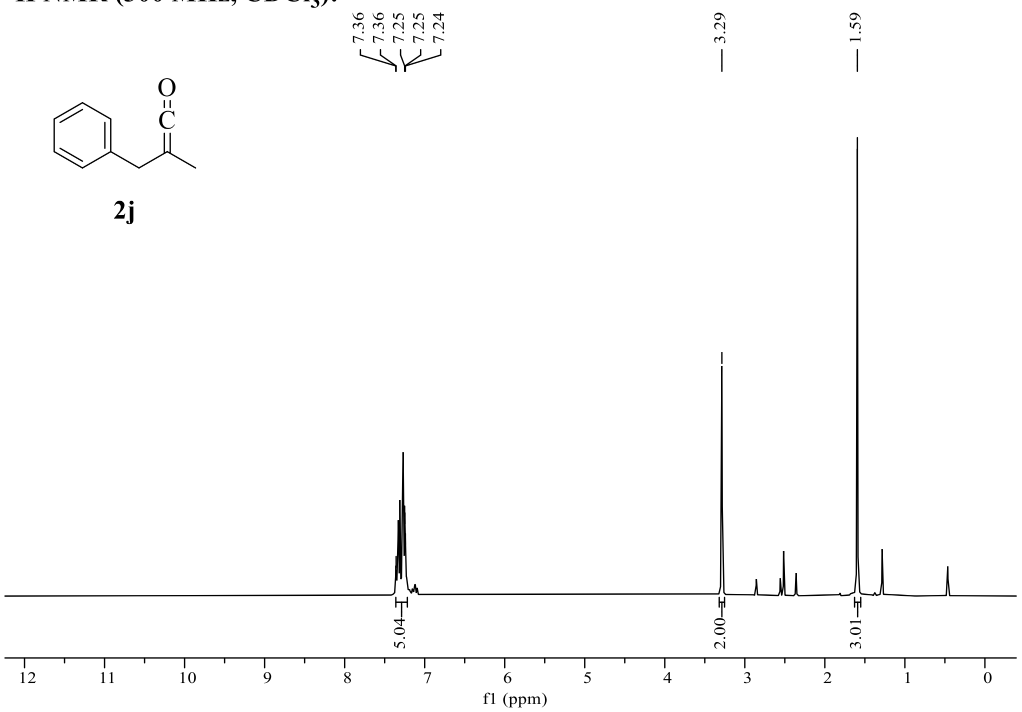

Supplementary Figure 84: ¹H NMR (300 MHz, $CDCl_3$) spectrum of compound **2j**.

¹H NMR (300 MHz, $CDCl_3$):

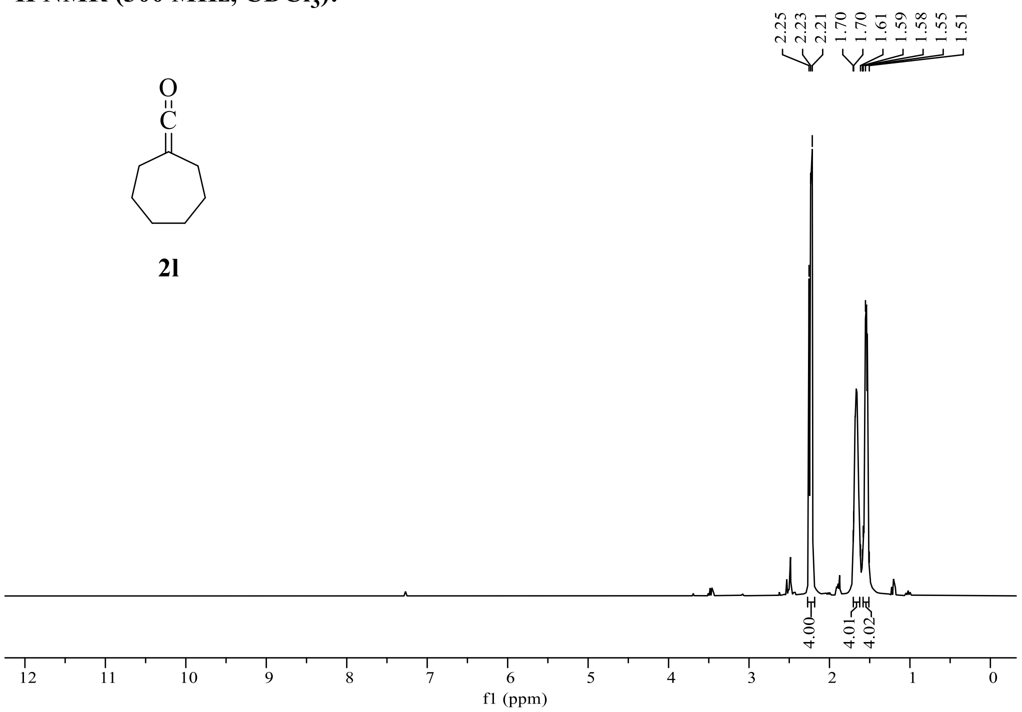

Supplementary Figure 85: ¹H NMR (300 MHz, $CDCl_3$) spectrum of compound **2l**.

^{1}H NMR (300 MHz, $CDCl_3$):

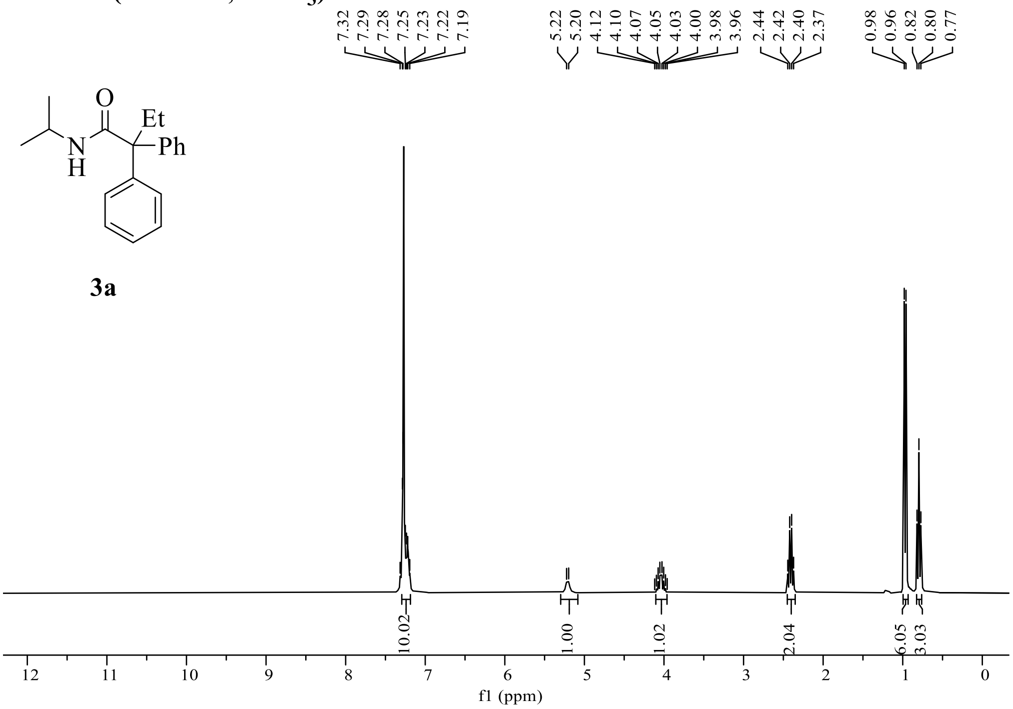

Supplementary Figure 86: ^{1}H NMR (300 MHz, $CDCl_3$) spectrum of compound **3a**.

^{13}C NMR (75 MHz, $CDCl_3$):

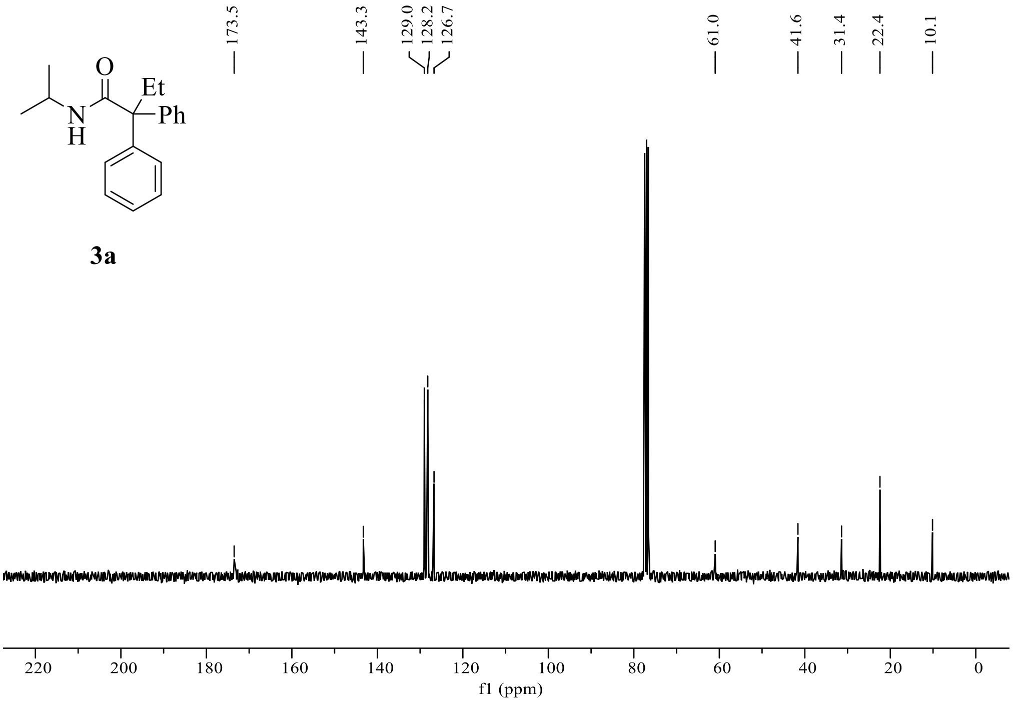

Supplementary Figure 87: ^{13}C NMR (75 MHz, $CDCl_3$) spectrum of compound **3a**.

^{1}H NMR (300 MHz, $CDCl_3$):

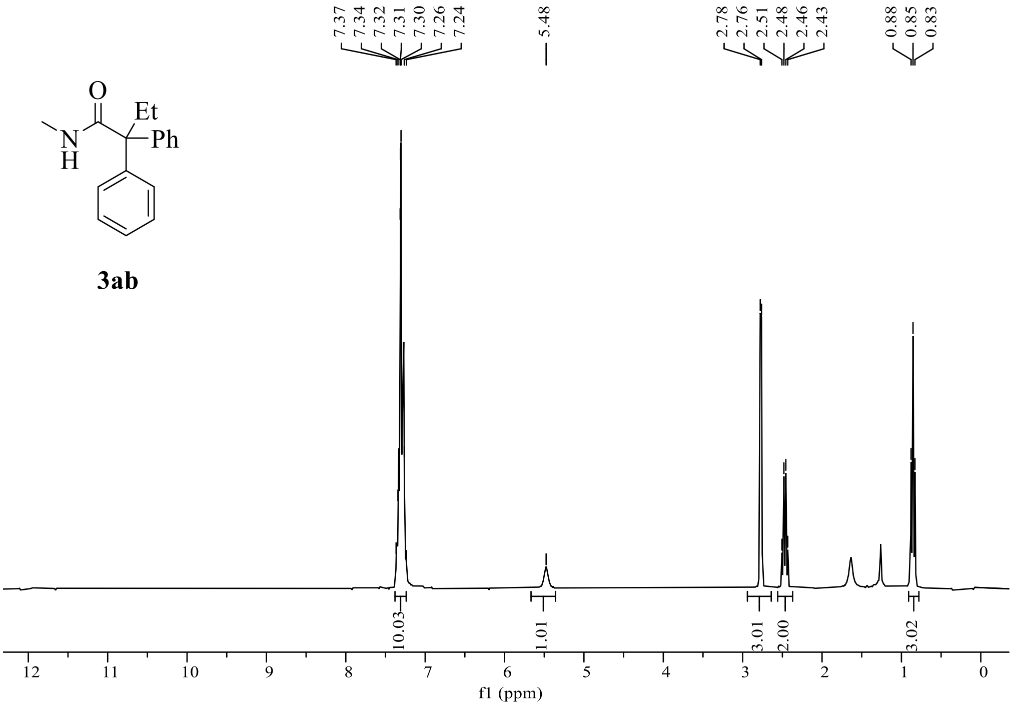

Supplementary Figure 88: ^{1}H NMR (300 MHz, $CDCl_3$) spectrum of compound **3ab**.

^{13}C NMR (75 MHz, $CDCl_3$):

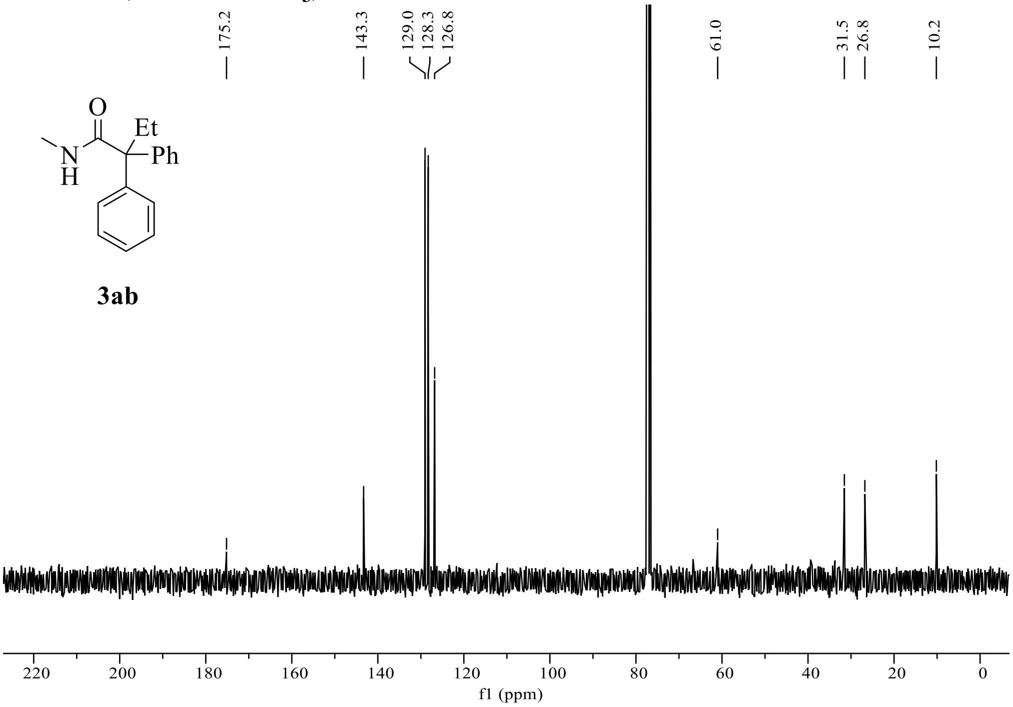

Supplementary Figure 89: ^{13}C NMR (75 MHz, $CDCl_3$) spectrum of compound **3ab**.

^{1}H NMR (300 MHz, $CDCl_3$):

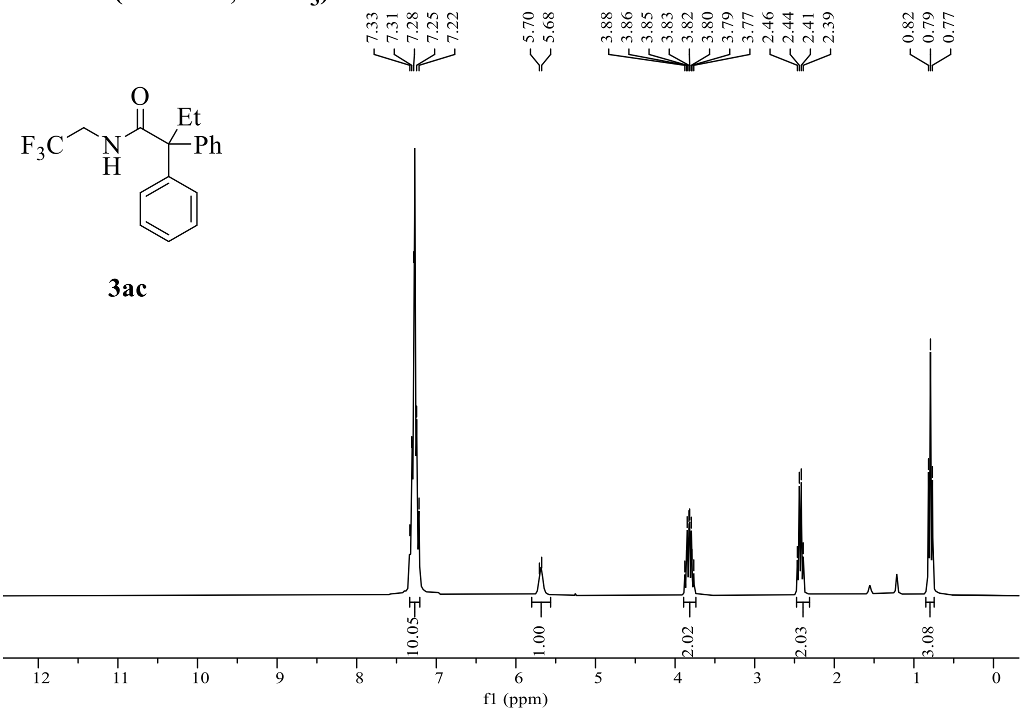

Supplementary Figure 90: ^{1}H NMR (300 MHz, $CDCl_3$) spectrum of compound **3ac**.

$^{13}C\{^1H,^{19}F\}$ NMR (151 MHz, $CDCl_3$):

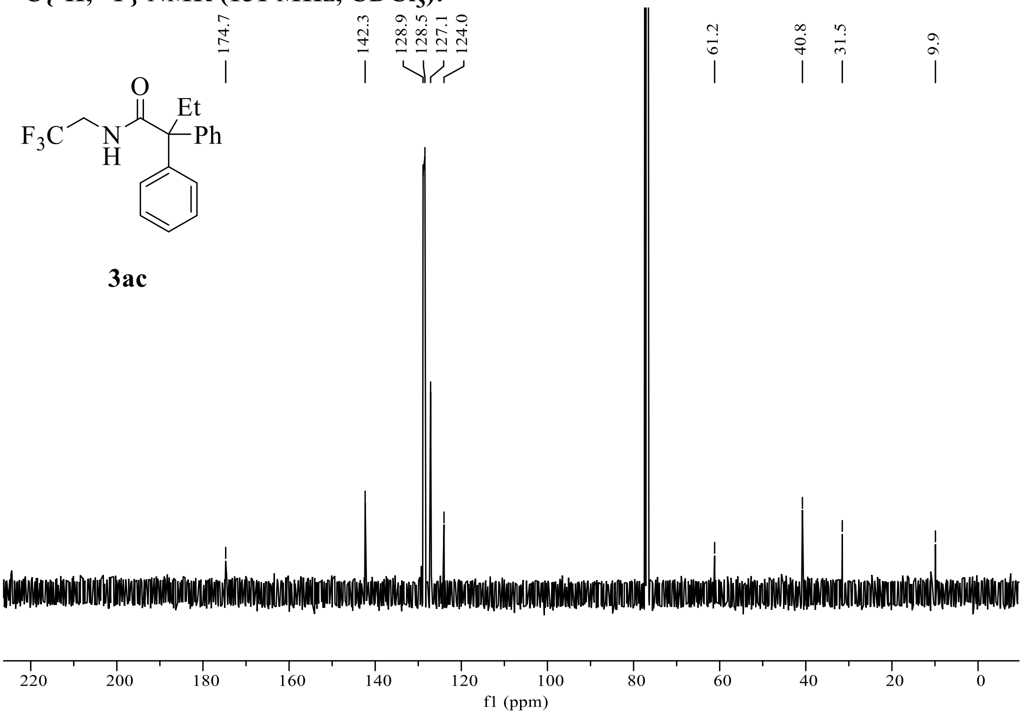

Supplementary Figure 91: $^{13}C\{^1H,^{19}F\}$ NMR (151 MHz, $CDCl_3$) spectrum of compound **3ac**.

$^{19}F\{^{1}H,^{13}C\}$ NMR (564 MHz, $CDCl_3$):

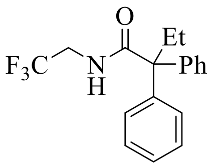

3ac

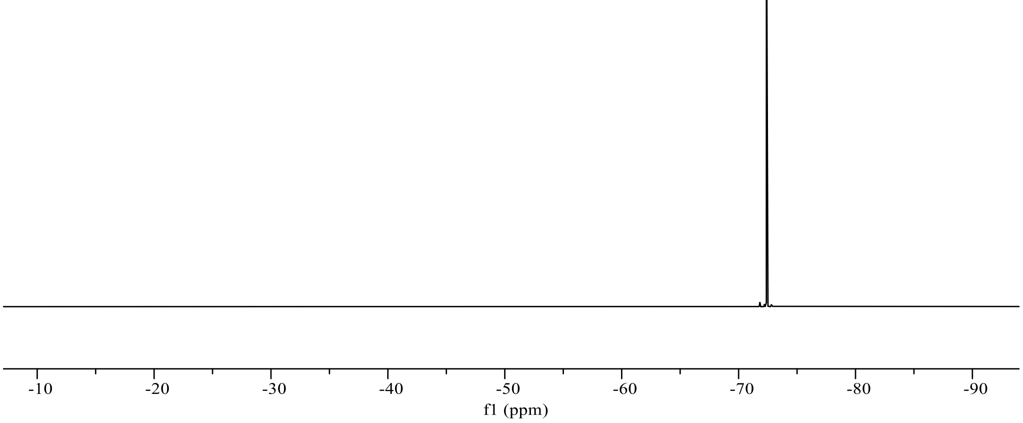

Supplementary Figure 92: $^{19}F\{^{1}H,^{13}C\}$ NMR (564 MHz, $CDCl_3$) spectrum of compound **3ac**.

^{1}H NMR (300 MHz, $CDCl_3$):

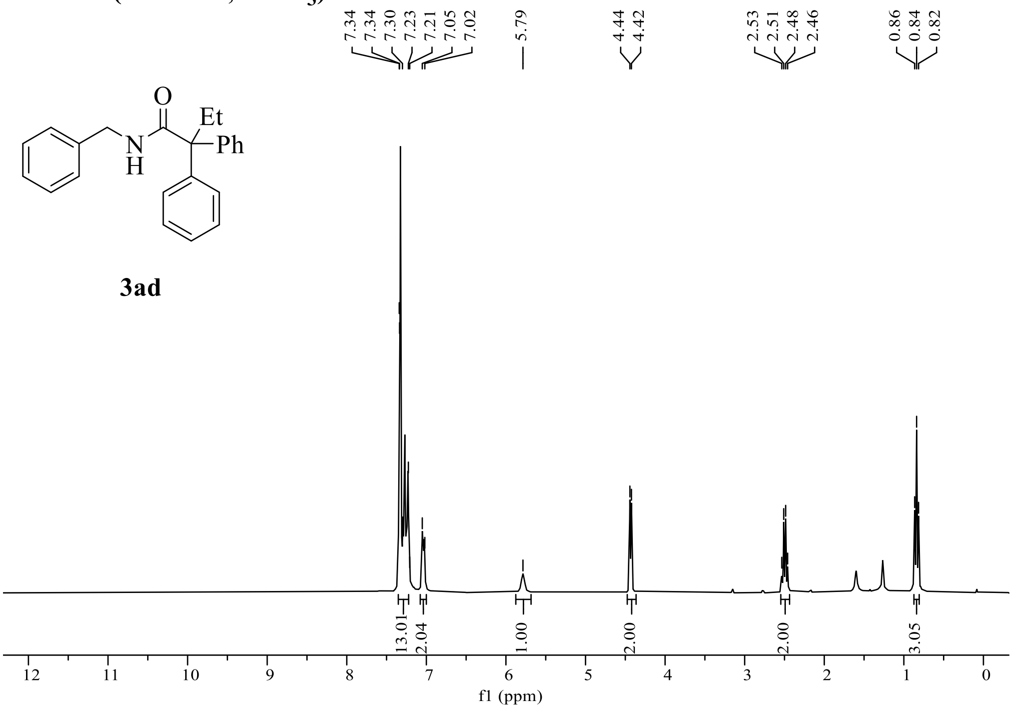

Supplementary Figure 93: ^{1}H NMR (300 MHz, $CDCl_3$) spectrum of compound **3ad**.

^{13}C NMR (75 MHz, $CDCl_3$):

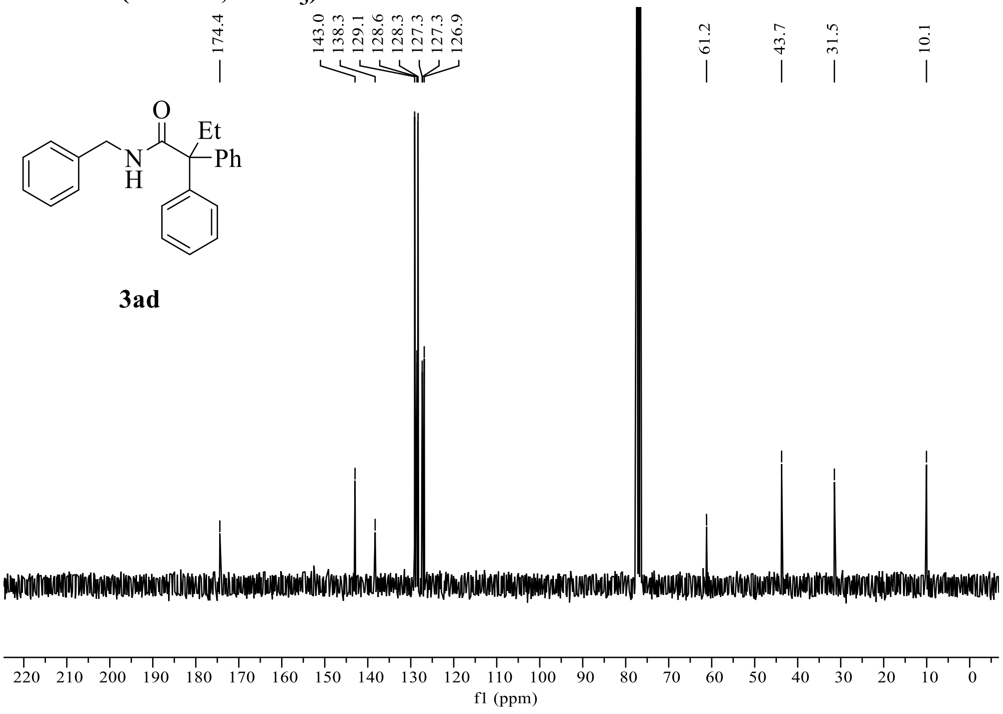

Supplementary Figure 94: ^{13}C NMR (75 MHz, $CDCl_3$) spectrum of compound **3ad**.

^{1}H NMR (300 MHz, CDCl$_3$):

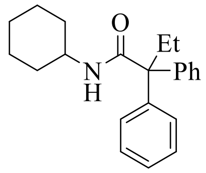

3ae

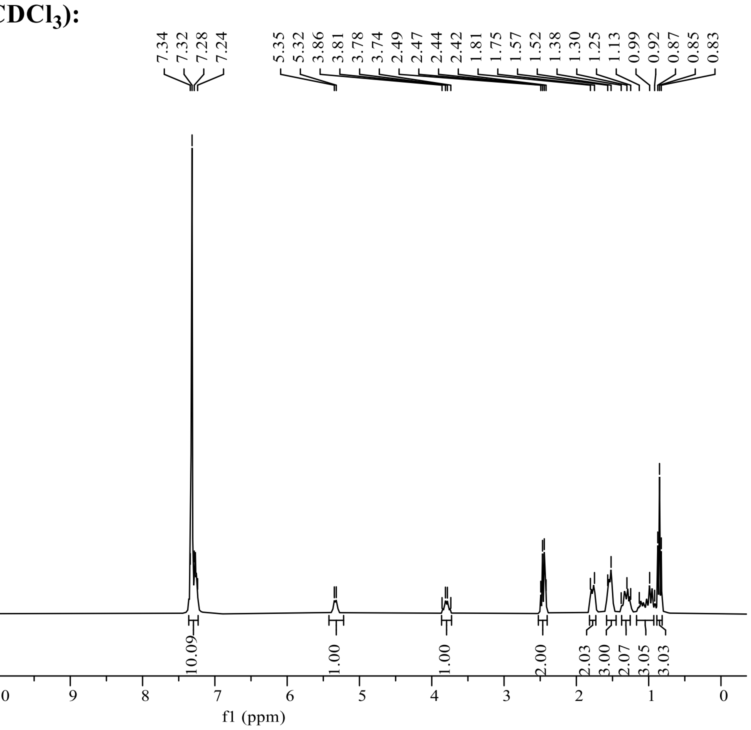

Supplementary Figure 95: ^{1}H NMR (300 MHz, CDCl$_3$) spectrum of compound **3ae**.

^{13}C NMR (75 MHz, CDCl$_3$):

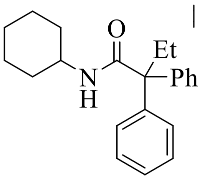

3ae

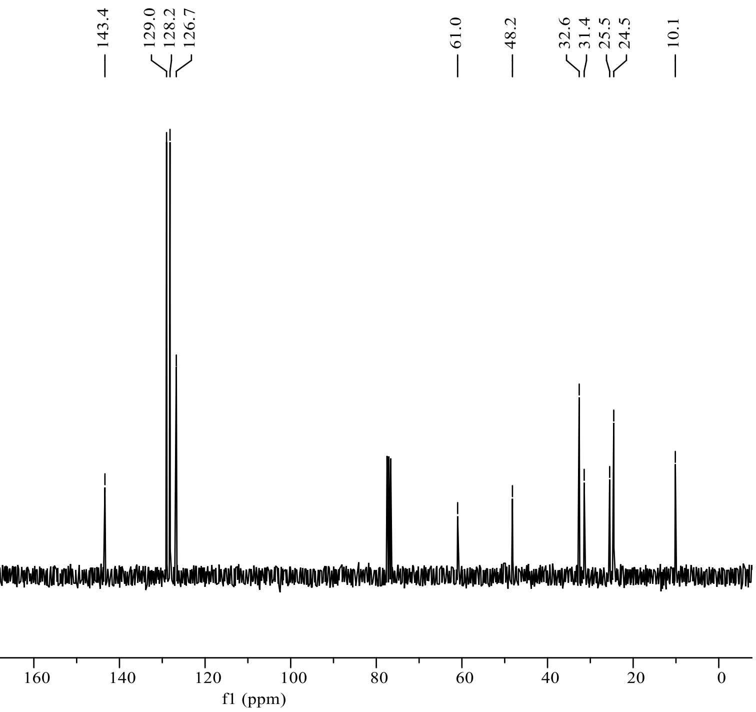

Supplementary Figure 96: ^{13}C NMR (75 MHz, CDCl$_3$) spectrum of compound **3ae**.

^{1}H NMR (300 MHz, $CDCl_3$):

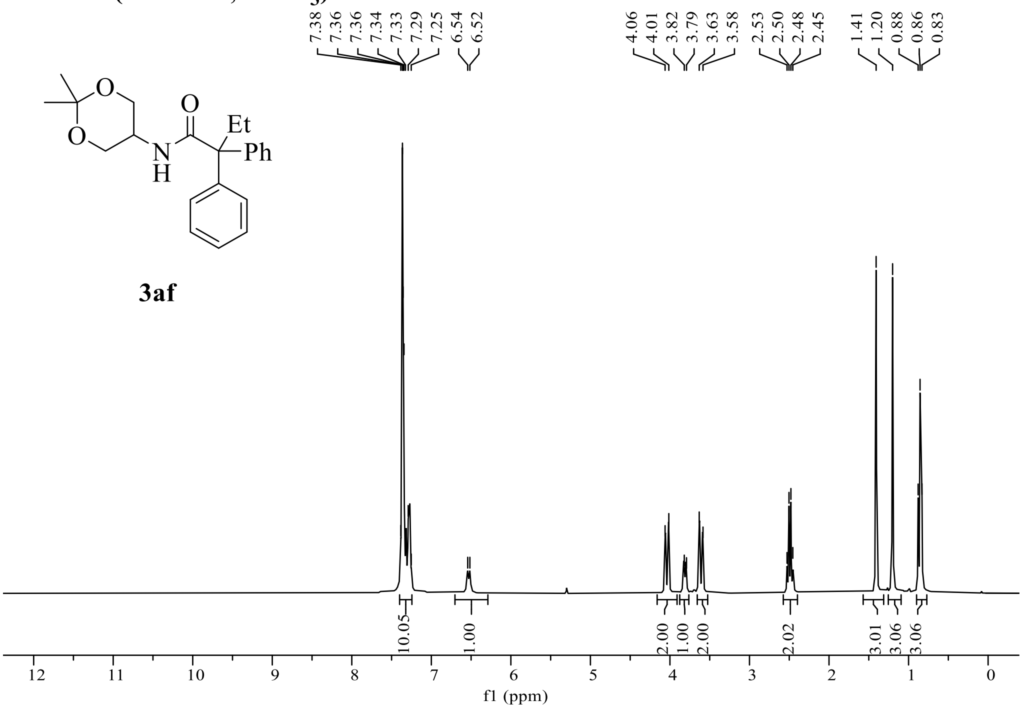

Supplementary Figure 97: ^{1}H NMR (300 MHz, $CDCl_3$) spectrum of compound **3af**.

^{13}C NMR (75 MHz, $CDCl_3$):

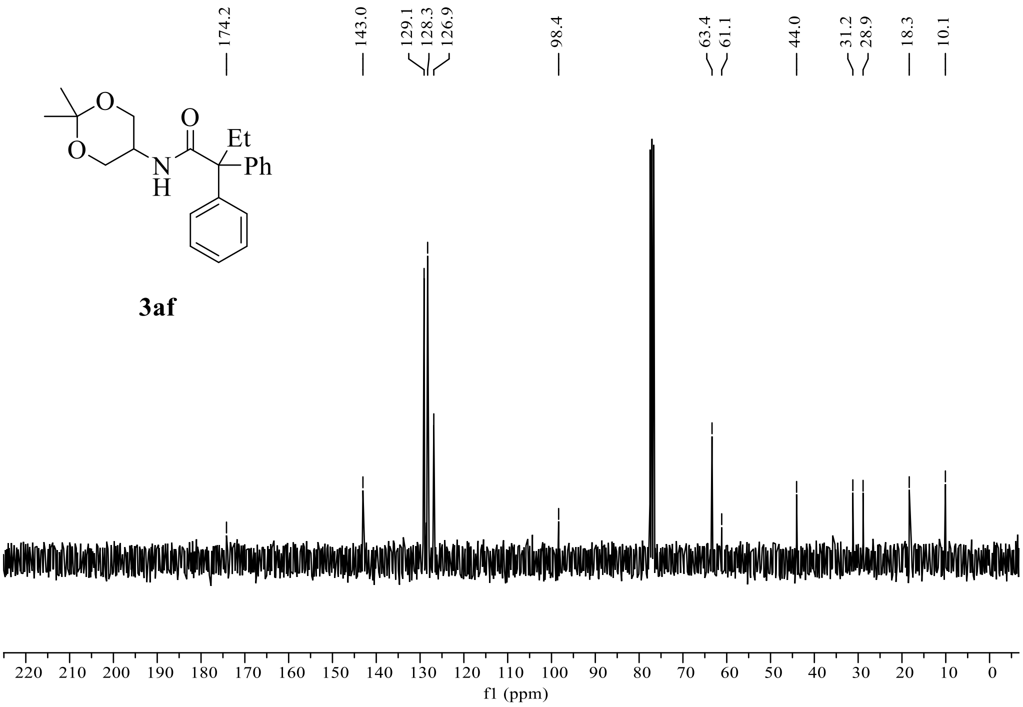

Supplementary Figure 98: ^{13}C NMR (75 MHz, $CDCl_3$) spectrum of compound **3af**.

^{1}H NMR (500 MHz, $CDCl_3$):

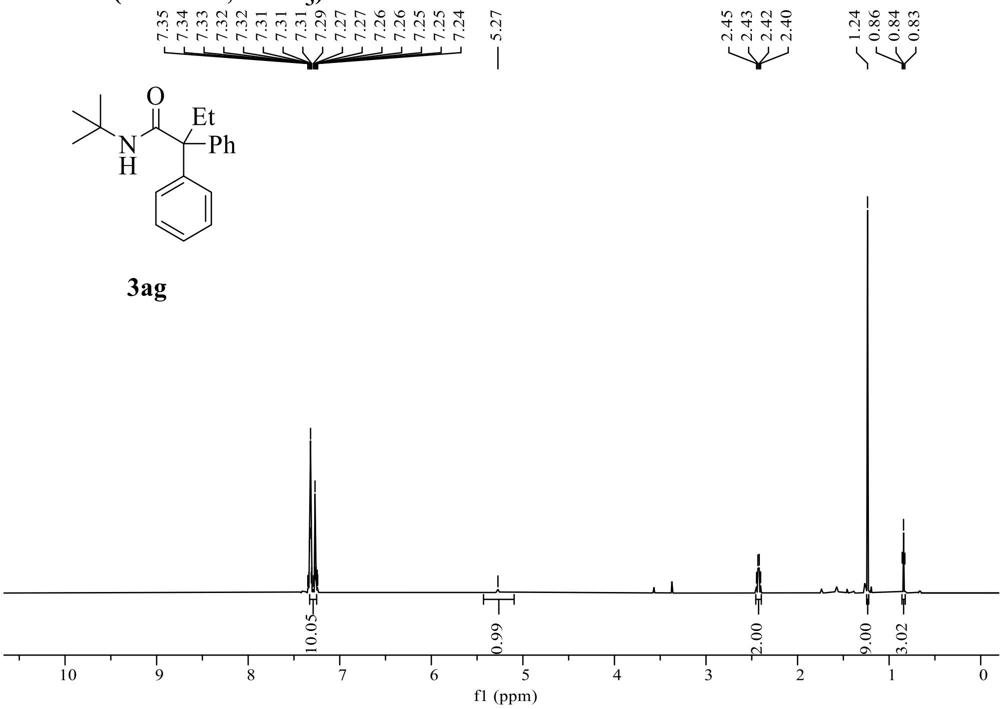

Supplementary Figure 99: ^{1}H NMR (500 MHz, $CDCl_3$) spectrum of compound **3ag**.

^{13}C NMR (126 MHz, $CDCl_3$):

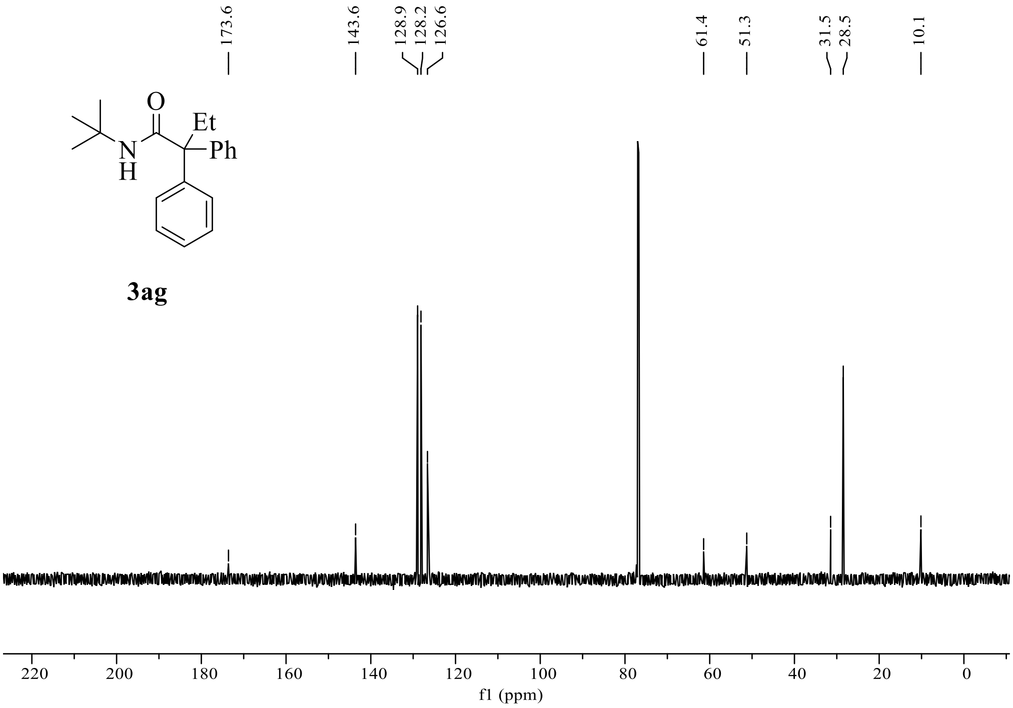

Supplementary Figure 100: ^{13}C NMR (126 MHz, $CDCl_3$) spectrum of compound **3ag**.

^{1}H NMR (300 MHz, CDCl$_3$):

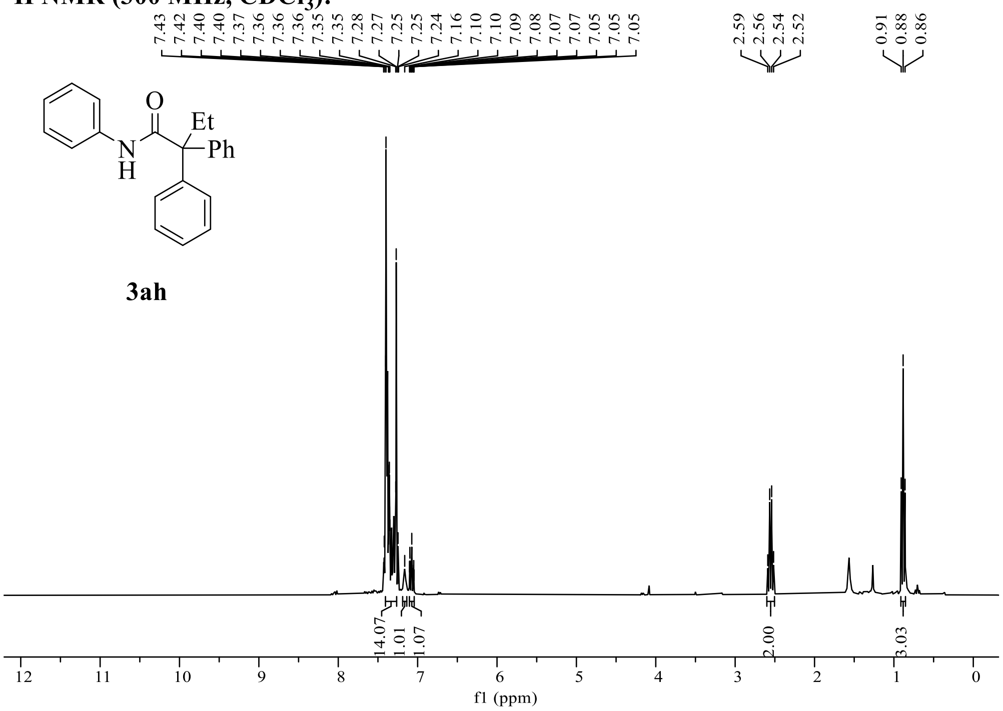

Supplementary Figure 101: ^{1}H NMR (300 MHz, CDCl$_3$) spectrum of compound **3ah**.

^{13}C NMR (75 MHz, CDCl$_3$):

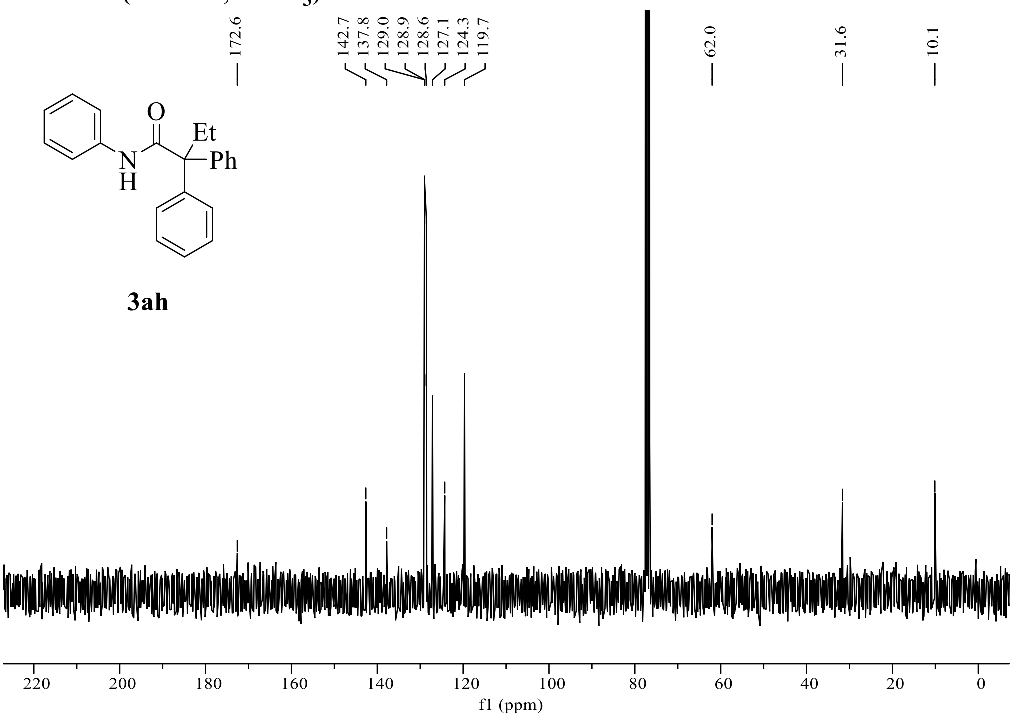

Supplementary Figure 102: ^{13}C NMR (75 MHz, CDCl$_3$) spectrum of compound **3ah**.

^{1}H NMR (300 MHz, $CDCl_3$):

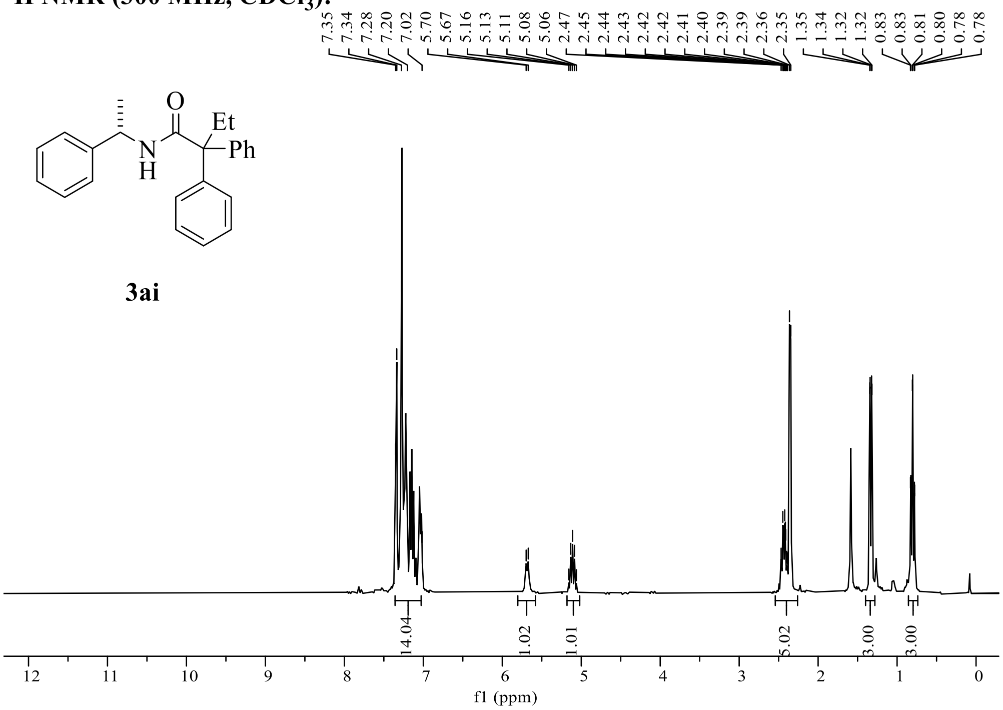

Supplementary Figure 103: ^{1}H NMR (300 MHz, $CDCl_3$) spectrum of compound **3ai**.

^{13}C NMR (151 MHz, $CDCl_3$):

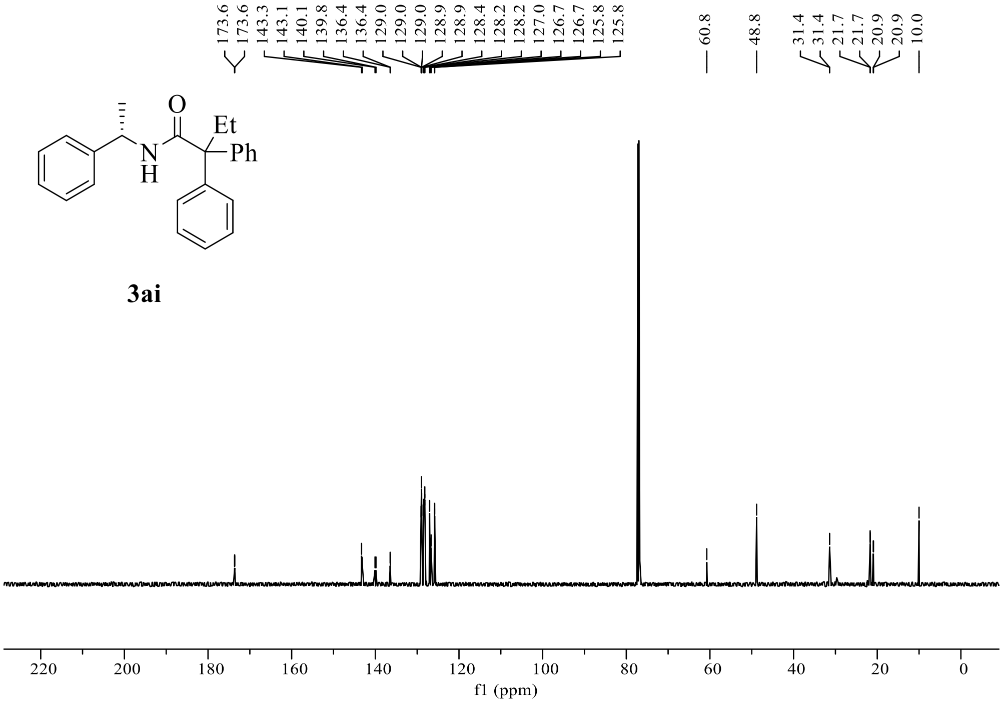

Supplementary Figure 104: ^{13}C NMR (151 MHz, $CDCl_3$) spectrum of compound **3ai**.

^{1}H NMR (300 MHz, CDCl$_3$):

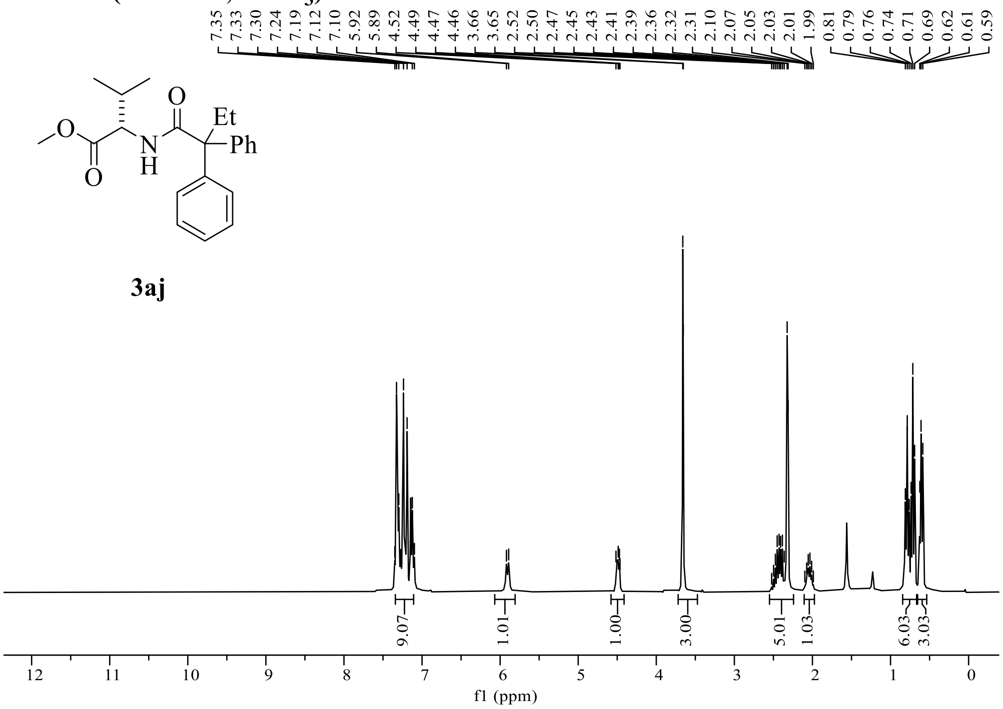

Supplementary Figure 105: ^{1}H NMR (300 MHz, CDCl$_3$) spectrum of compound **3aj**.

^{13}C NMR (151 MHz, CDCl$_3$):

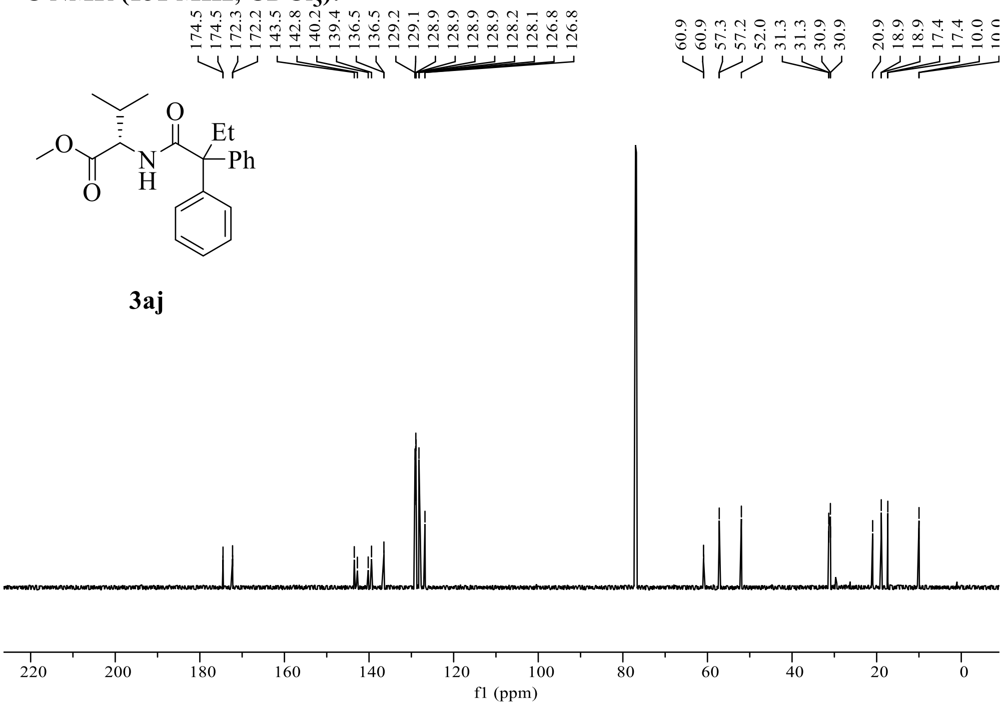

Supplementary Figure 106: ^{13}C NMR (151 MHz, CDCl$_3$) spectrum of compound **3aj**.

^{1}H NMR (300 MHz, CDCl$_3$):

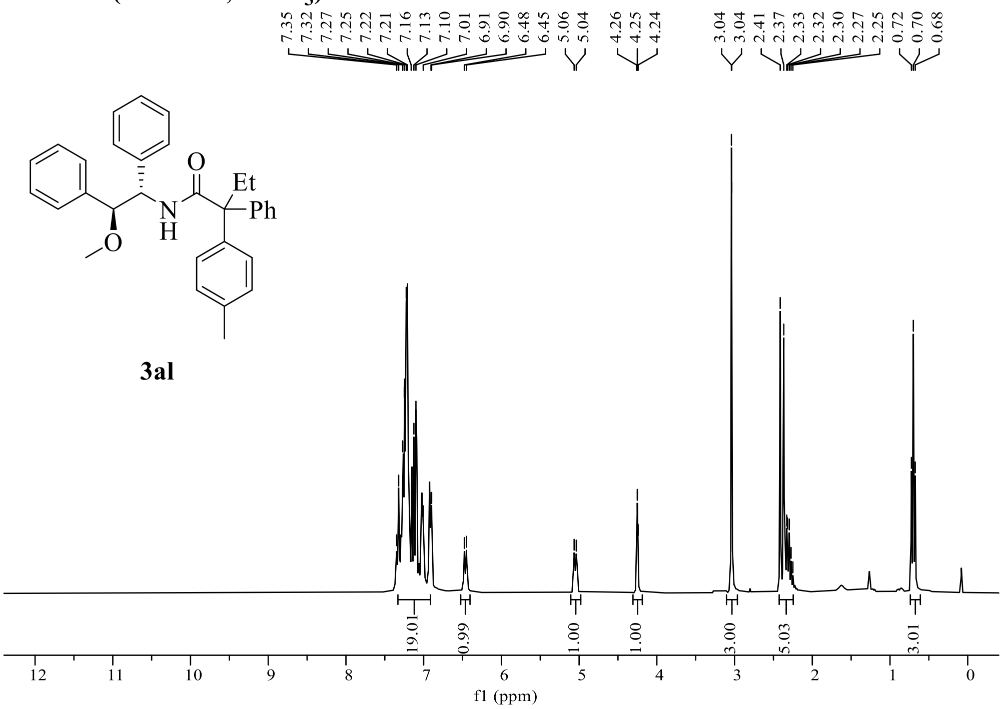

Supplementary Figure 107: ^{1}H NMR (300 MHz, CDCl$_3$) spectrum of compound **3al**.

^{13}C NMR (75 MHz, CDCl$_3$):

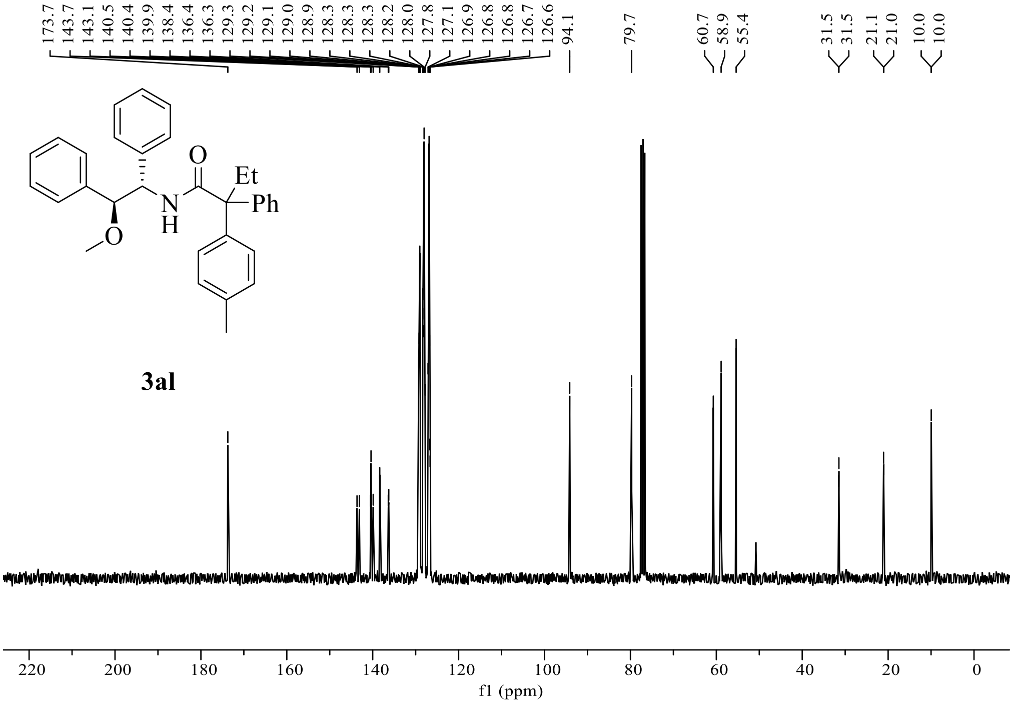

Supplementary Figure 108: ^{13}C NMR (75 MHz, CDCl$_3$) spectrum of compound **3al**.

^{1}H NMR (300 MHz, $CDCl_3$):

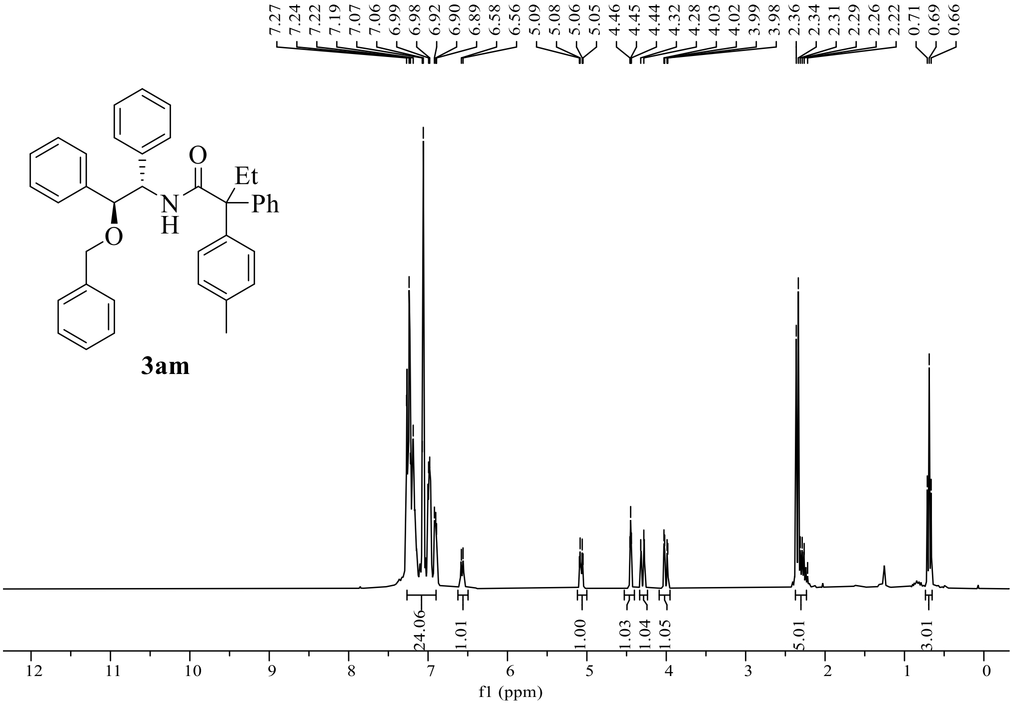

Supplementary Figure 109: ^{1}H NMR (300 MHz, $CDCl_3$) spectrum of compound **3am**.

^{13}C NMR (75 MHz, $CDCl_3$):

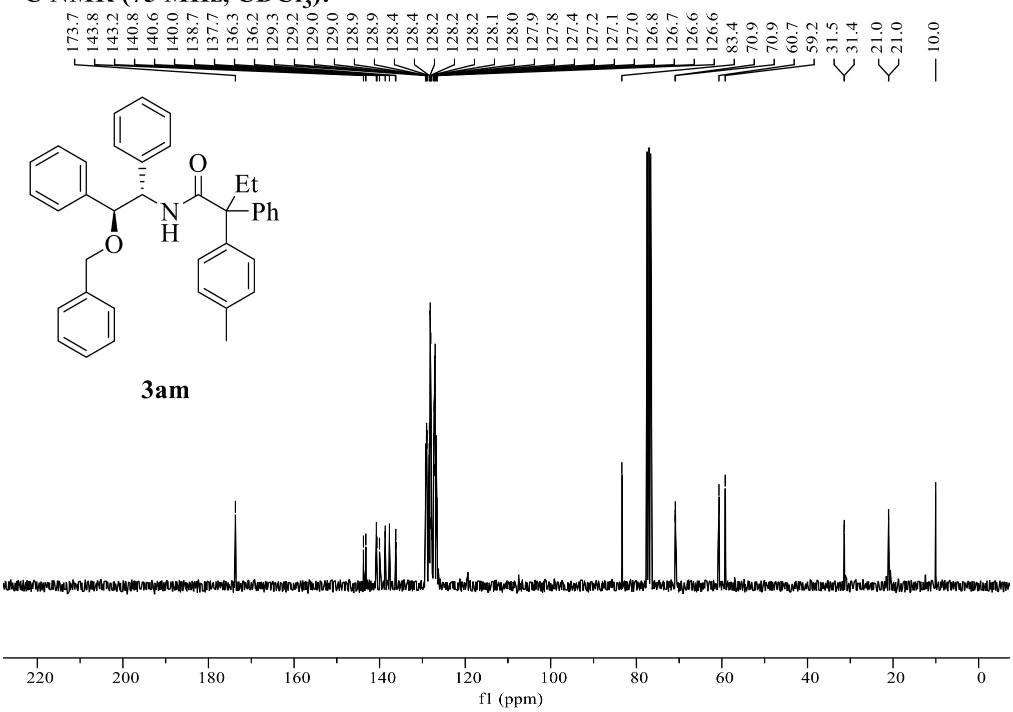

Supplementary Figure 110: ^{13}C NMR (75 MHz, $CDCl_3$) spectrum of compound **3am**.

^{1}H NMR (300 MHz, CDCl$_3$):

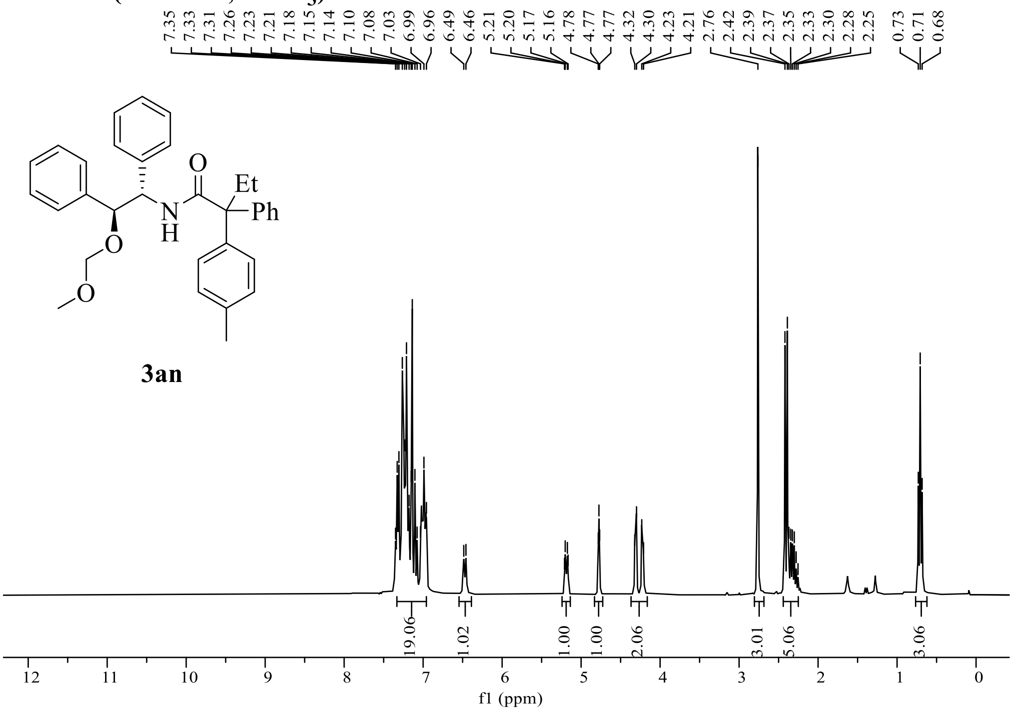

Supplementary Figure 111: ^{1}H NMR (300 MHz, CDCl$_3$) spectrum of compound **3an**.

^{13}C NMR (75 MHz, CDCl$_3$):

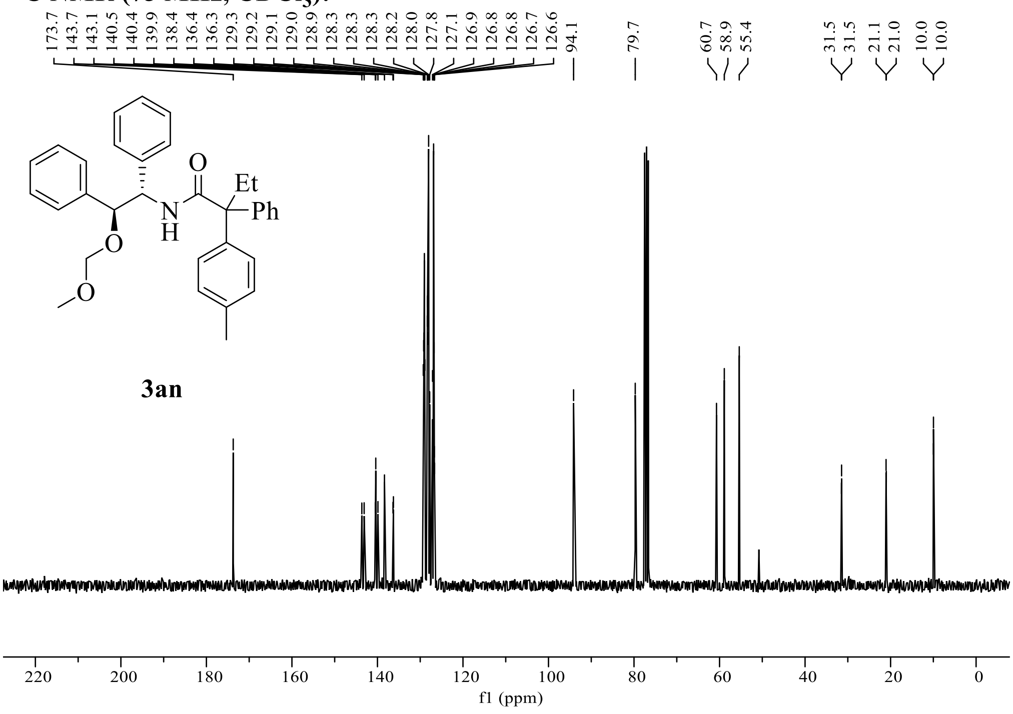

Supplementary Figure 112: ^{13}C NMR (75 MHz, CDCl$_3$) spectrum of compound **3an**.

^{1}H NMR (300 MHz, $CDCl_3$):

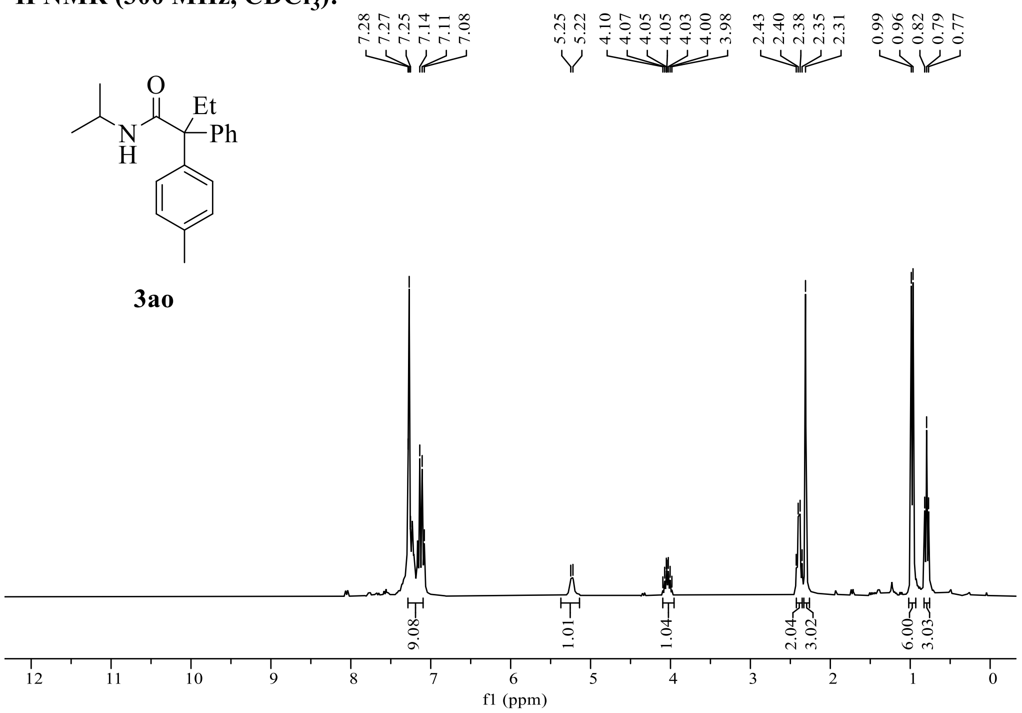

Supplementary Figure 113: ^{1}H NMR (300 MHz, $CDCl_3$) spectrum of compound **3ao**.

^{13}C NMR (75 MHz, $CDCl_3$):

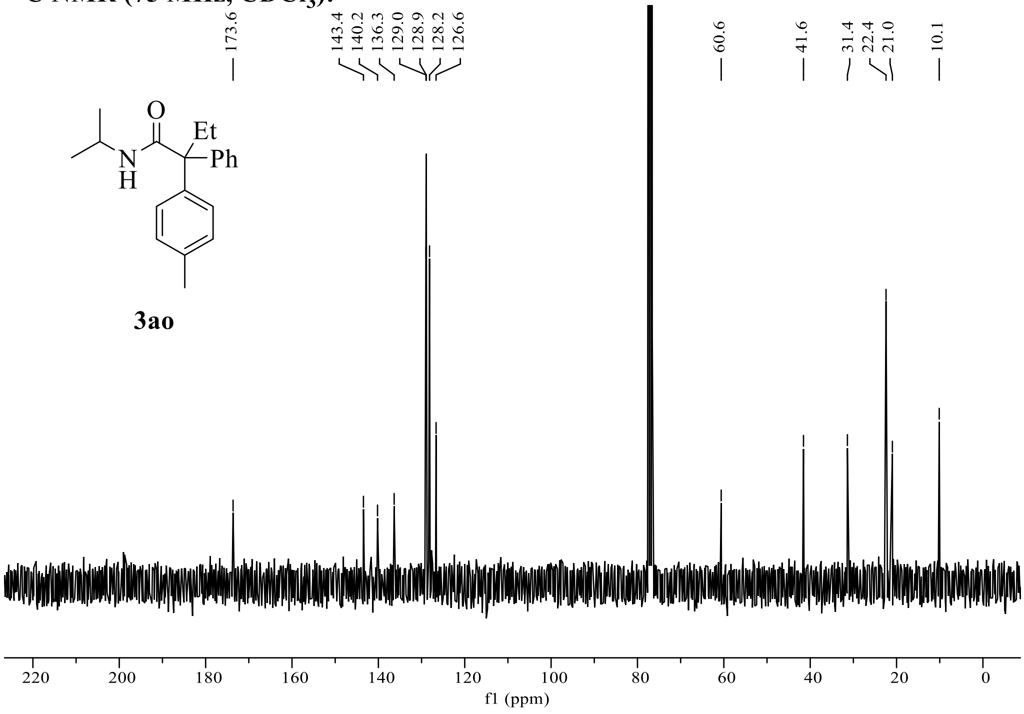

Supplementary Figure 114: ^{13}C NMR (75 MHz, $CDCl_3$) spectrum of compound **3ao**.

^{1}H NMR (300 MHz, $CDCl_3$):

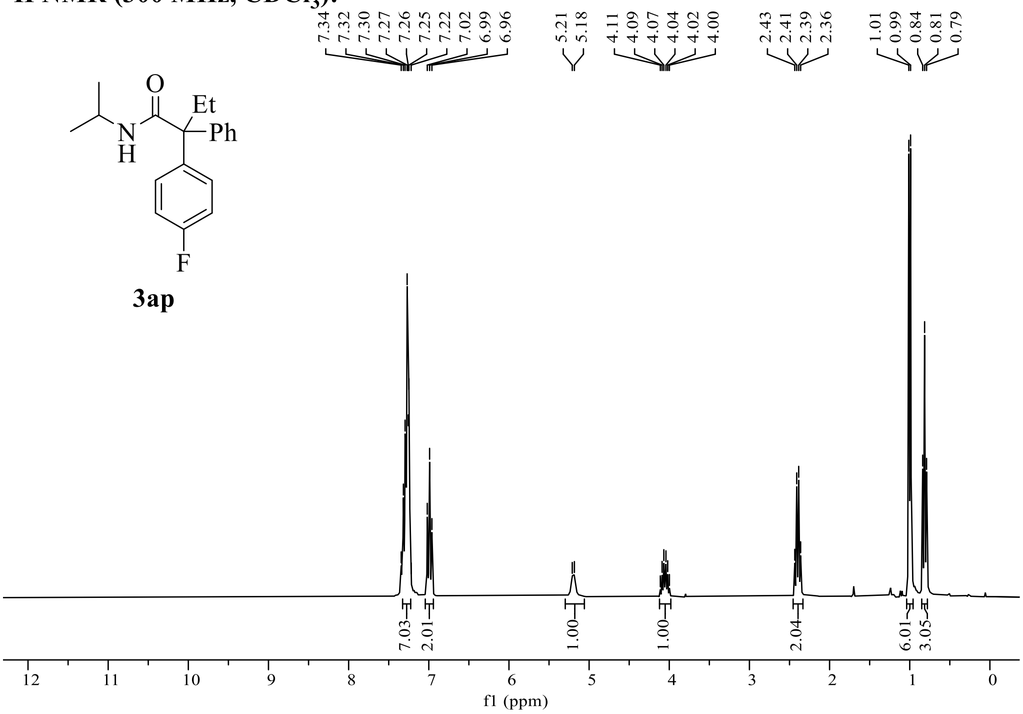

Supplementary Figure 115: ^{1}H NMR (300 MHz, $CDCl_3$) spectrum of compound **3ap**.

^{13}C NMR (75 MHz, $CDCl_3$):

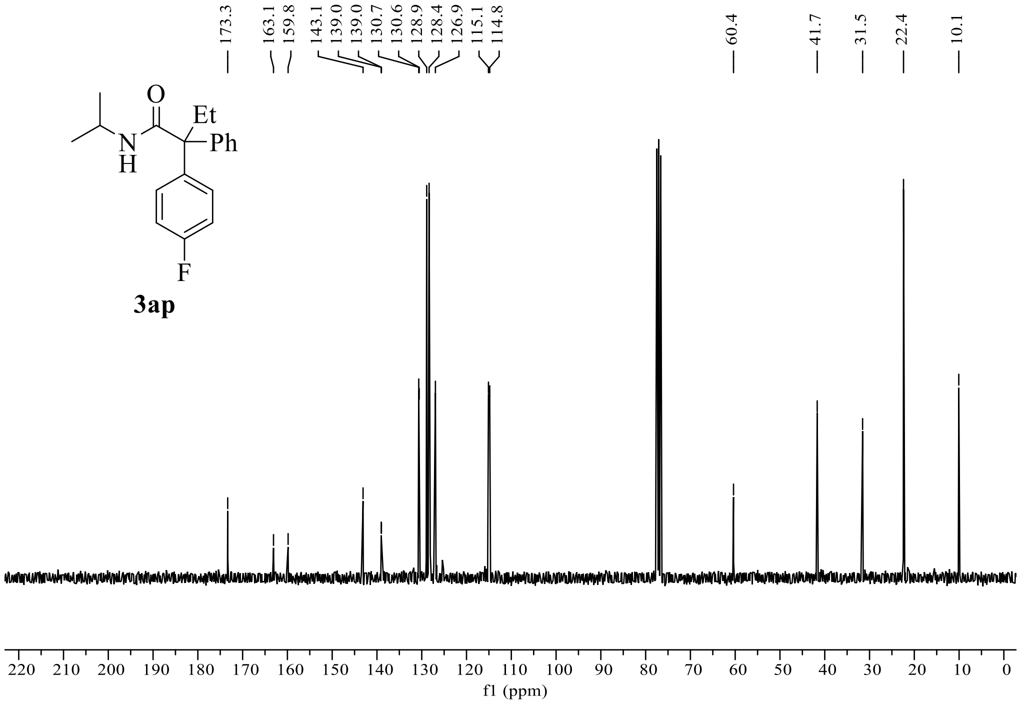

Supplementary Figure 116: ^{13}C NMR (750 MHz, $CDCl_3$) spectrum of compound **3ap**.

$^{19}F\{^1H,^{13}C\}$ NMR (283 MHz, $CDCl_3$):

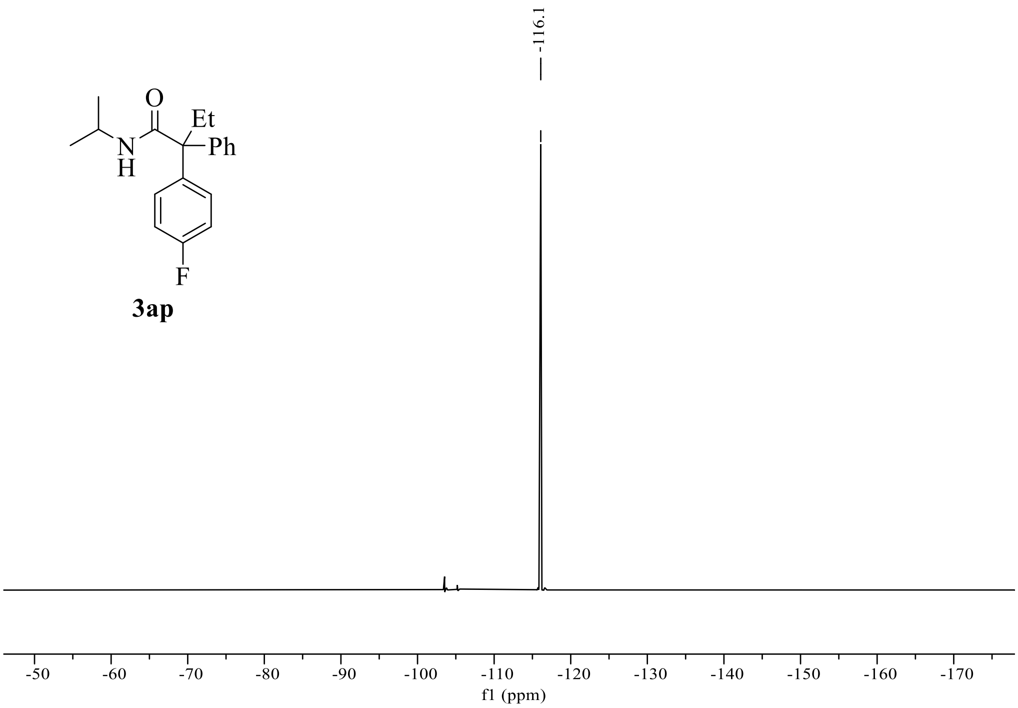

3ap

Supplementary Figure 117: ^{19}F NMR (283 MHz, $CDCl_3$) spectrum of compound **3ap**.

^{1}H NMR (300 MHz, CDCl$_3$):

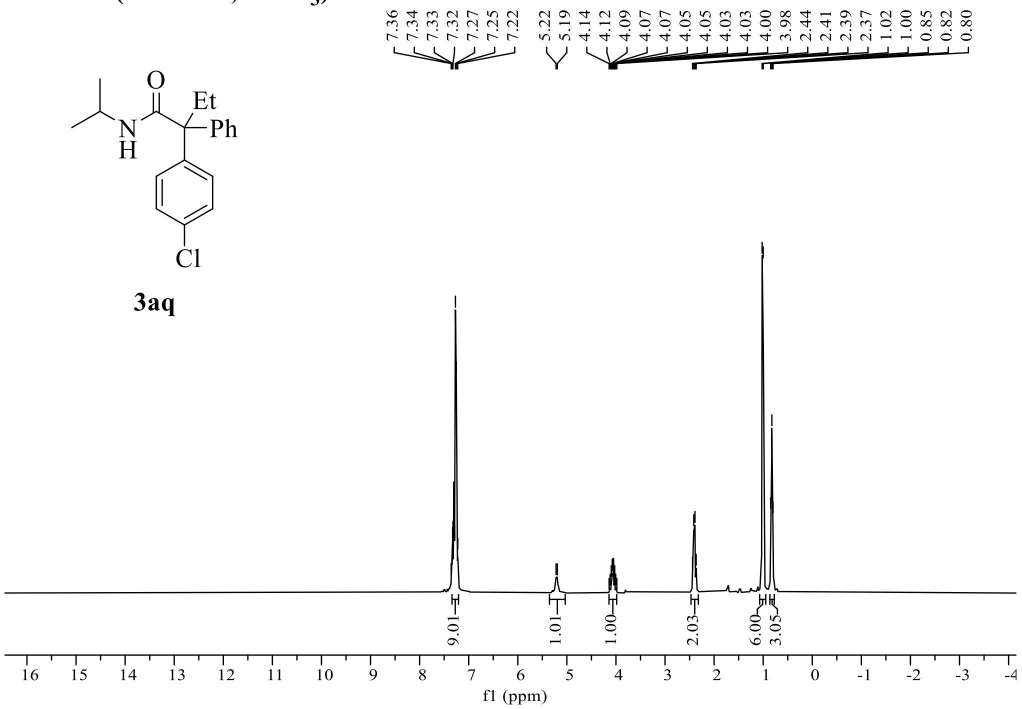

Supplementary Figure 118: ^{1}H NMR (300 MHz, CDCl$_3$) spectrum of compound **3aq**.

^{13}C NMR (75 MHz, CDCl$_3$):

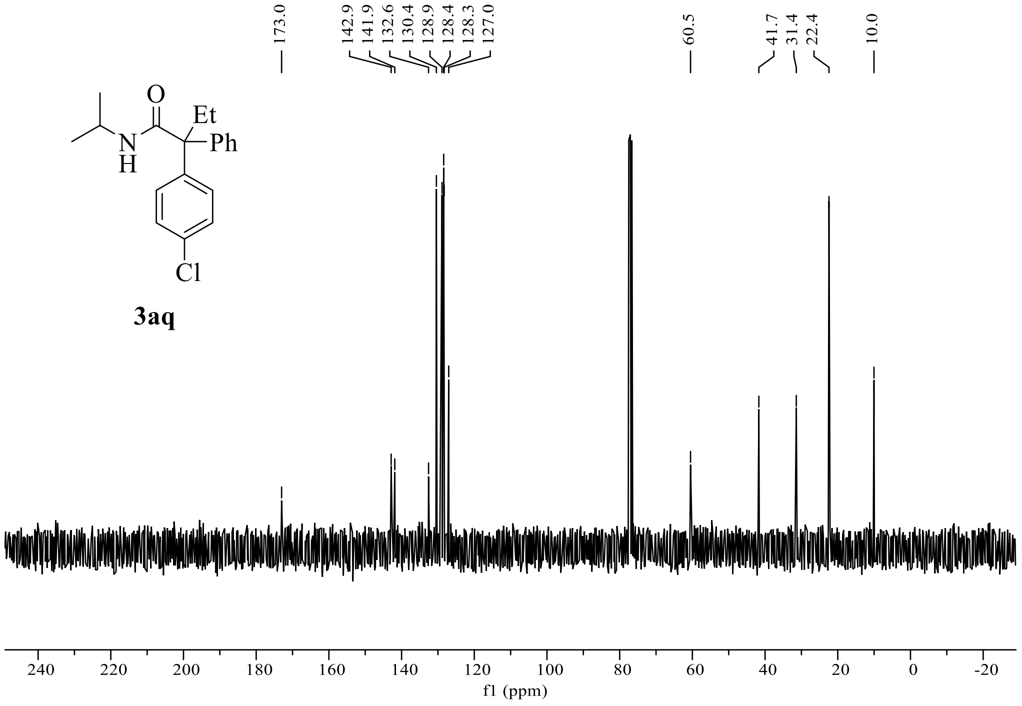

Supplementary Figure 119: ^{13}C NMR (75 MHz, CDCl$_3$) spectrum of compound **3aq**.

^{1}H NMR (300 MHz, $CDCl_3$):

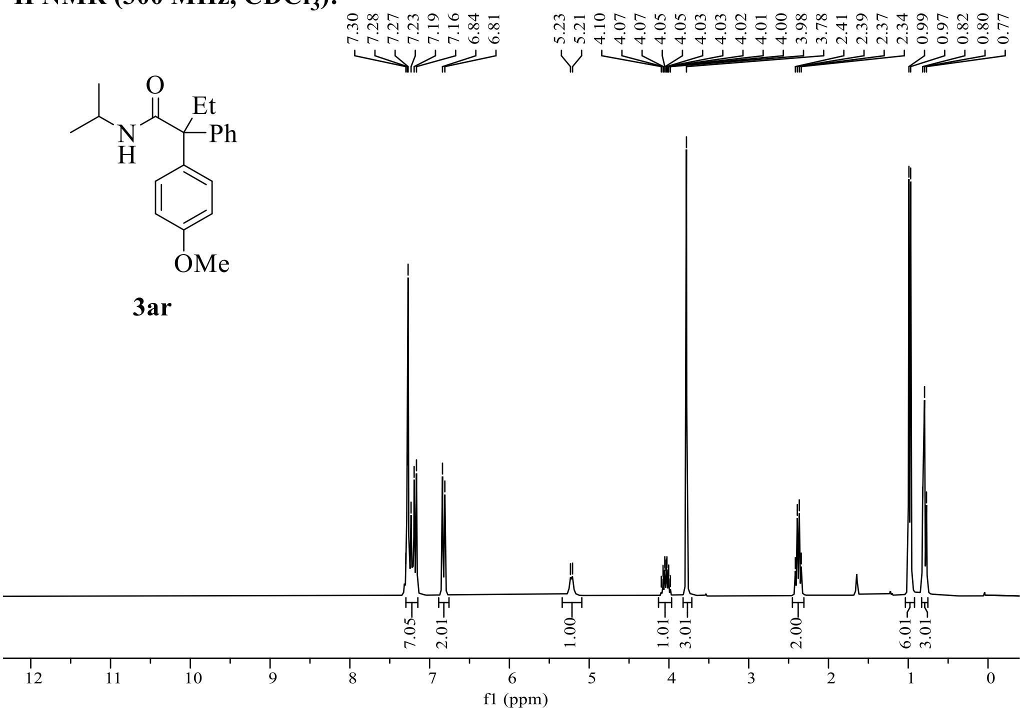

Supplementary Figure 120: ^{1}H NMR (300 MHz, $CDCl_3$) spectrum of compound **3ar**.

^{13}C NMR (75 MHz, $CDCl_3$):

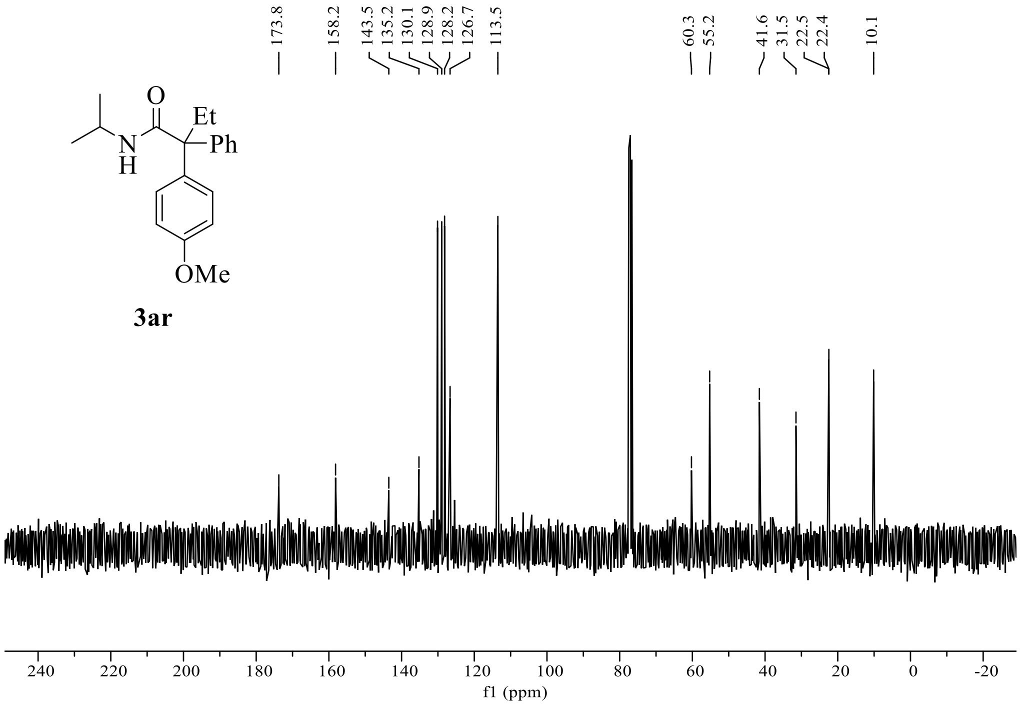

Supplementary Figure 121: ^{13}C NMR (75 MHz, $CDCl_3$) spectrum of compound **3ar**.

^{1}H NMR (300 MHz, $CDCl_3$):

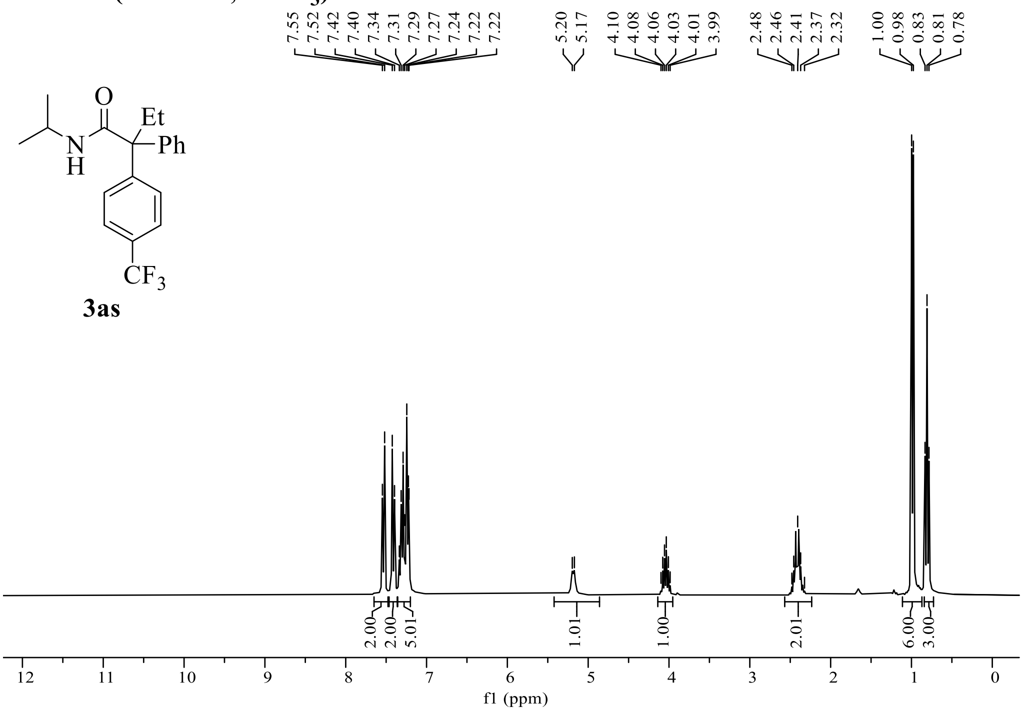

Supplementary Figure 122: ^{1}H NMR (300 MHz, $CDCl_3$) spectrum of compound **3as**.

$^{13}C\{^1H,^{19}F\}$ NMR (151 MHz, $CDCl_3$):

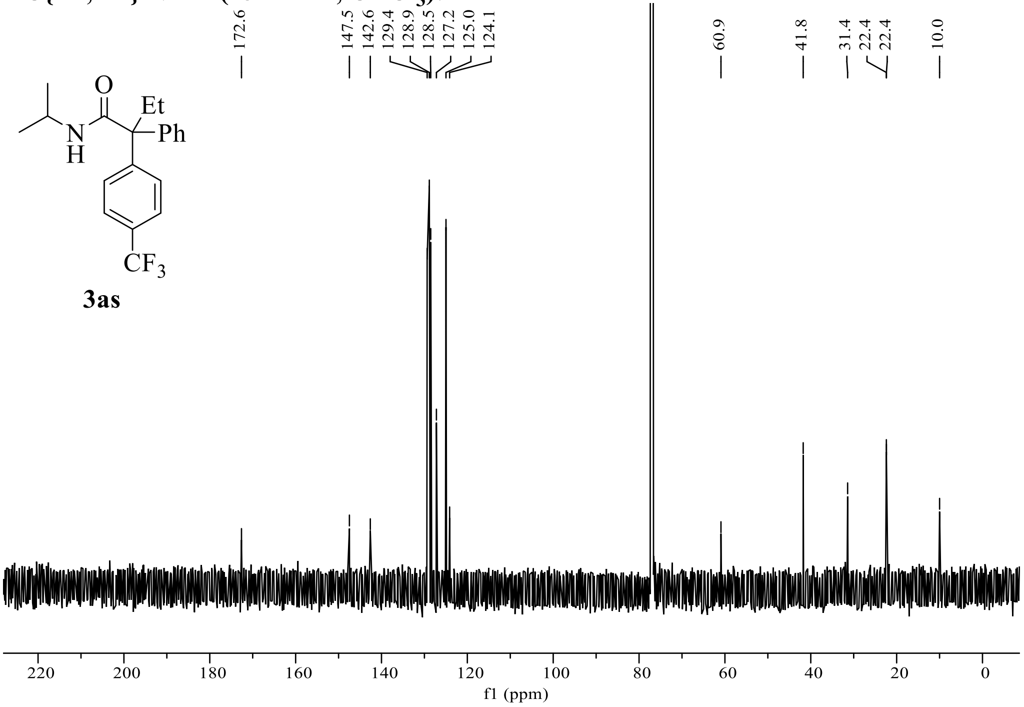

Supplementary Figure 123: $^{13}C\{^1H,^{19}F\}$ NMR (151 MHz, $CDCl_3$) spectrum of compound **3as**.

$^{19}F\{^{1}H,^{13}C\}$ NMR (564 MHz, $CDCl_3$):

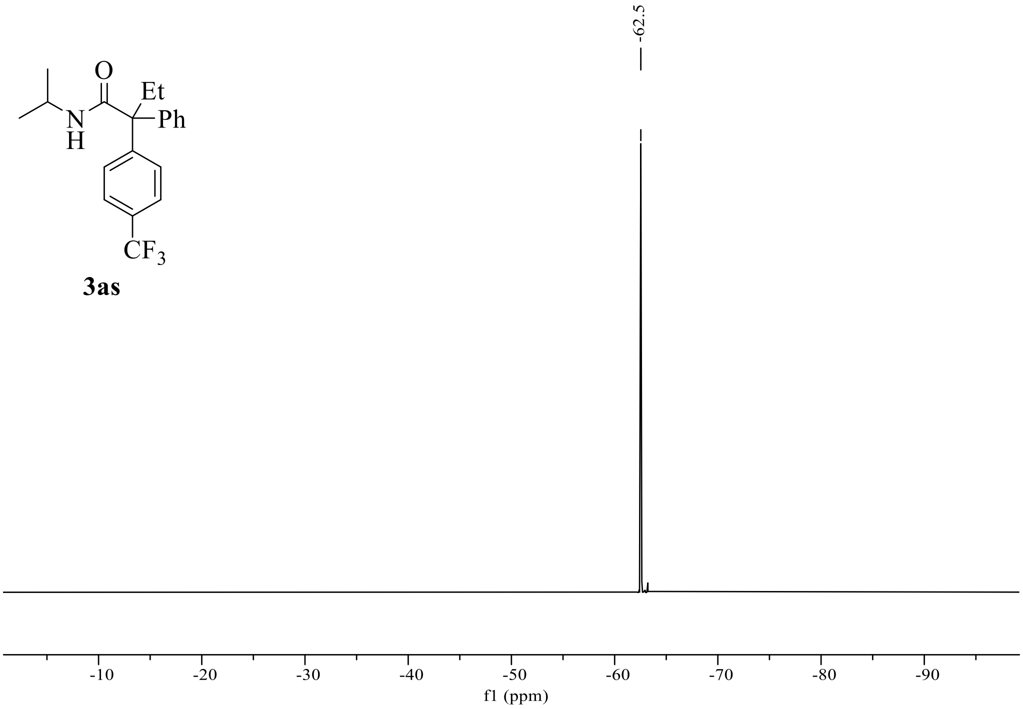

Supplementary Figure 124: $^{19}F\{^{1}H,^{13}C\}$ NMR (564 MHz, $CDCl_3$) spectrum of compound **3as**.

^{1}H NMR (300 MHz, $CDCl_3$):

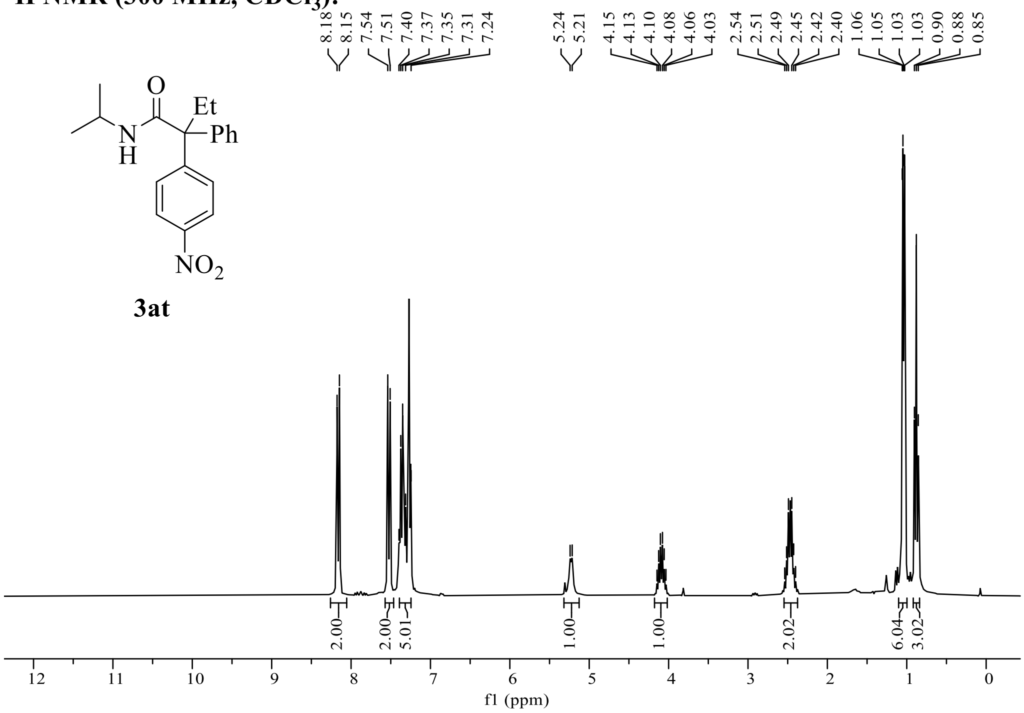

Supplementary Figure 125: ^{1}H NMR (300 MHz, $CDCl_3$) spectrum of compound **3at**.

^{13}C NMR (75 MHz, $CDCl_3$):

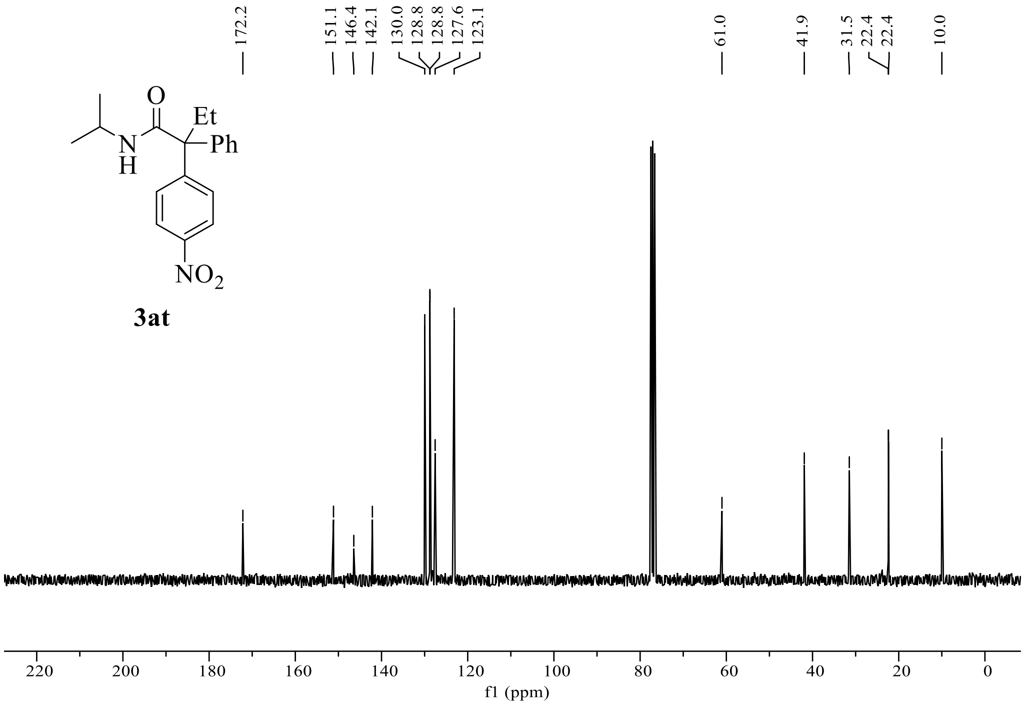

Supplementary Figure 126: ^{13}C NMR (75 MHz, $CDCl_3$) spectrum of compound **3at**.

^{1}H NMR (300 MHz, CDCl$_3$):

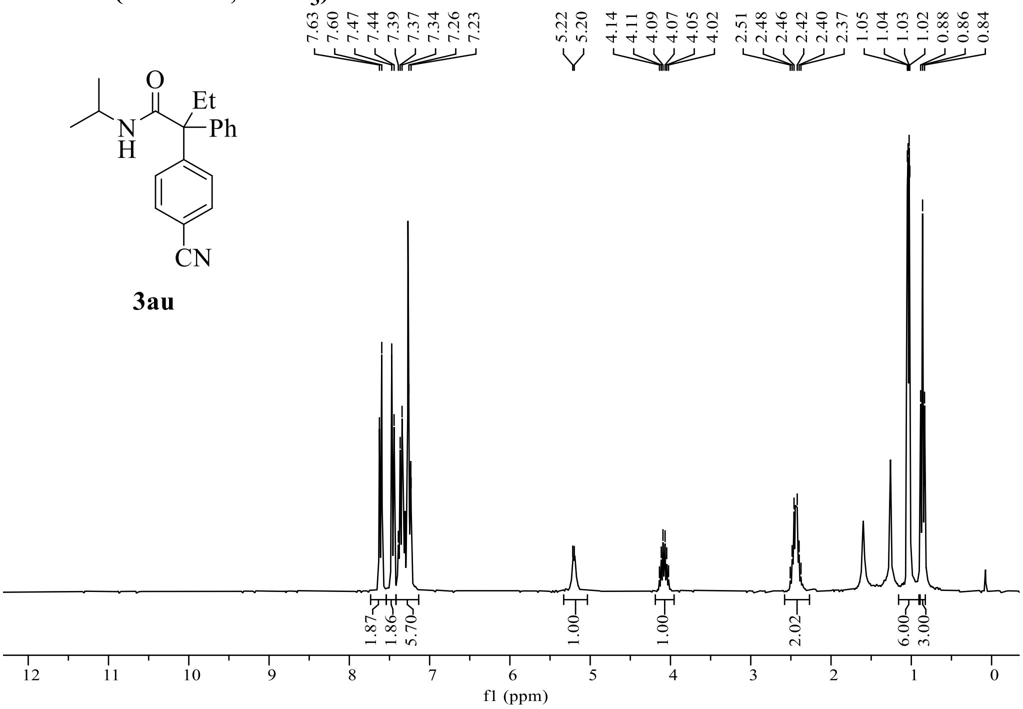

Supplementary Figure 127: ^{1}H NMR (300 MHz, CDCl$_3$) spectrum of compound **3au**.

^{13}C NMR (75 MHz, CDCl$_3$):

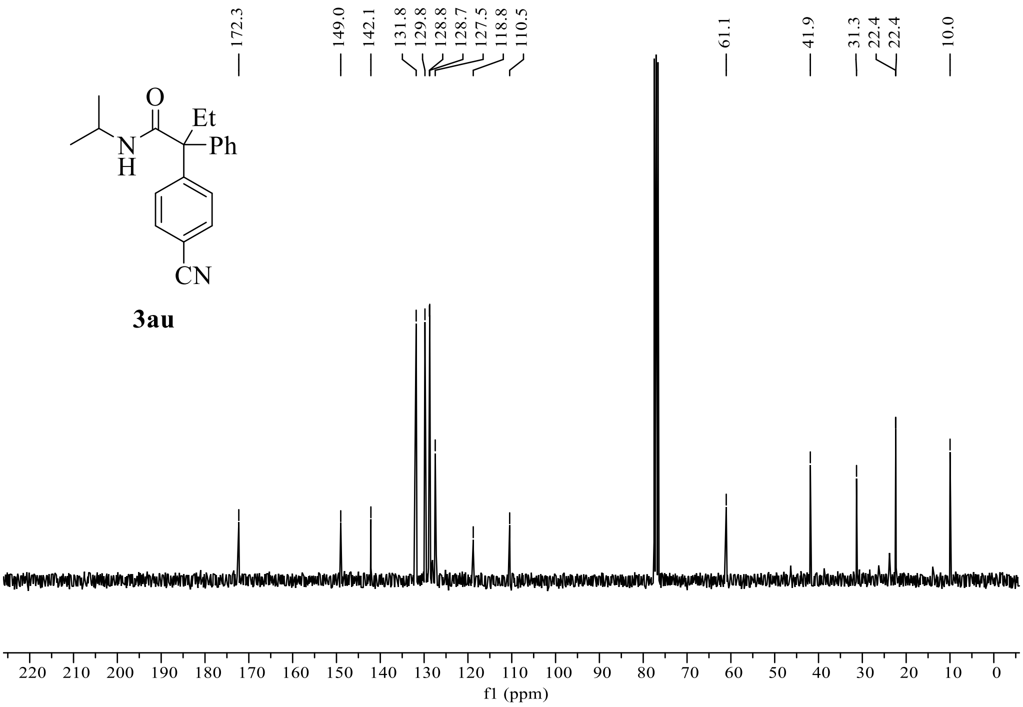

Supplementary Figure 128: ^{13}C NMR (75 MHz, CDCl$_3$) spectrum of compound **3au**.

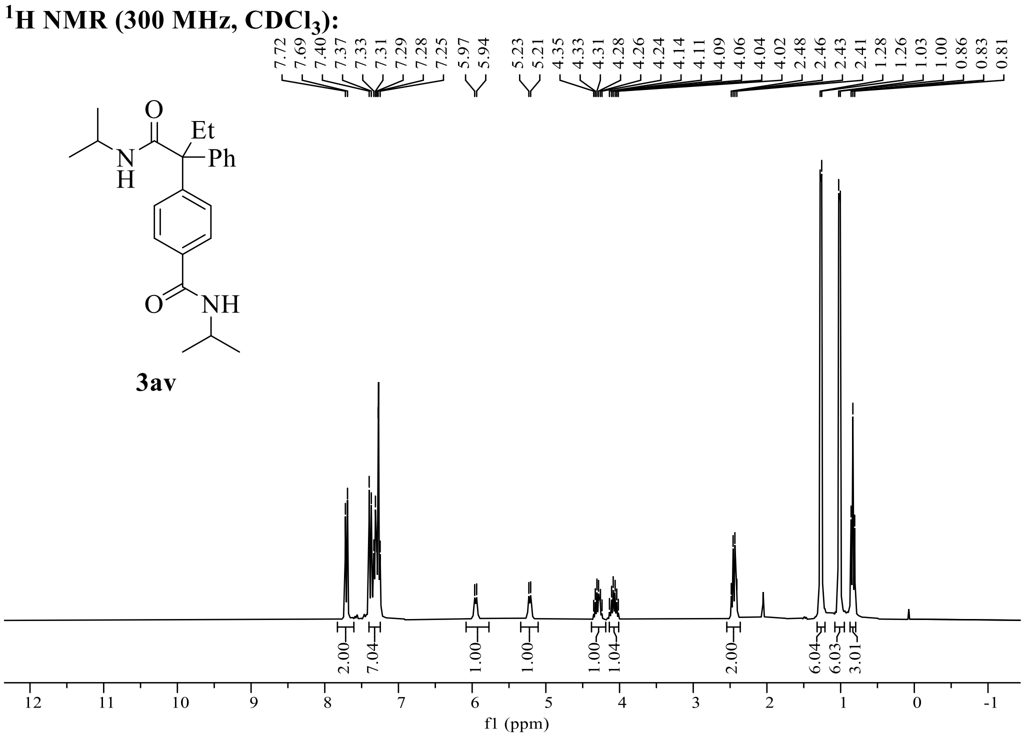

Supplementary Figure 129: ^{1}H NMR (300 MHz, $CDCl_3$) spectrum of compound **3av**.

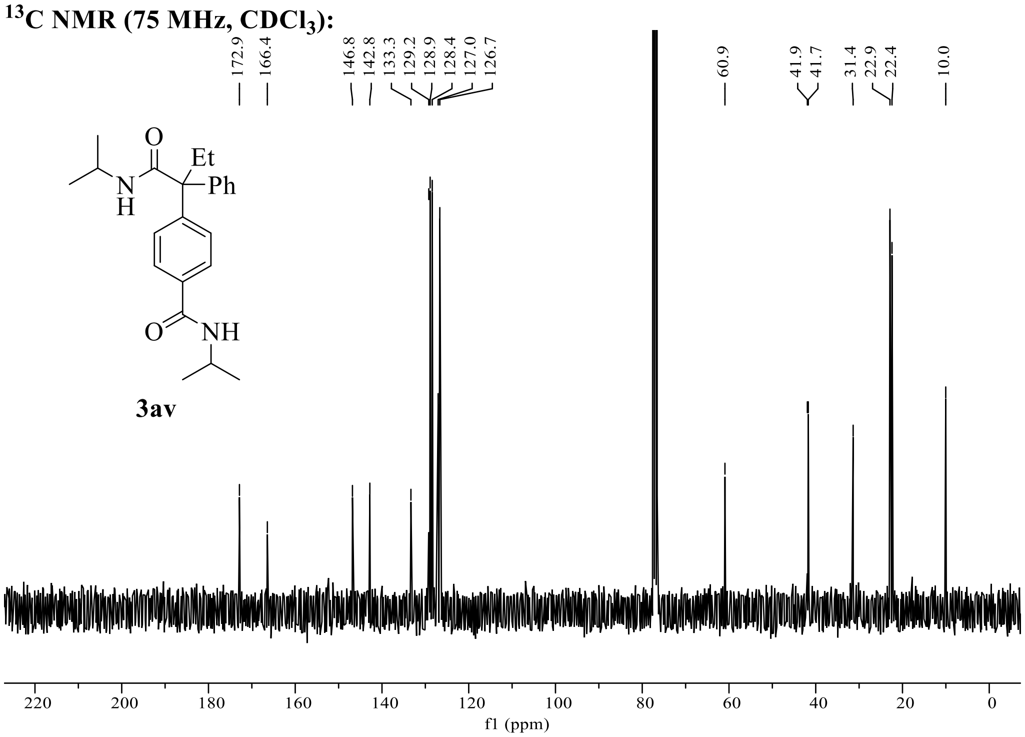

Supplementary Figure 130: ^{13}C NMR (75 MHz, $CDCl_3$) spectrum of compound **3av**.

^{1}H NMR (300 MHz, CDCl$_3$):

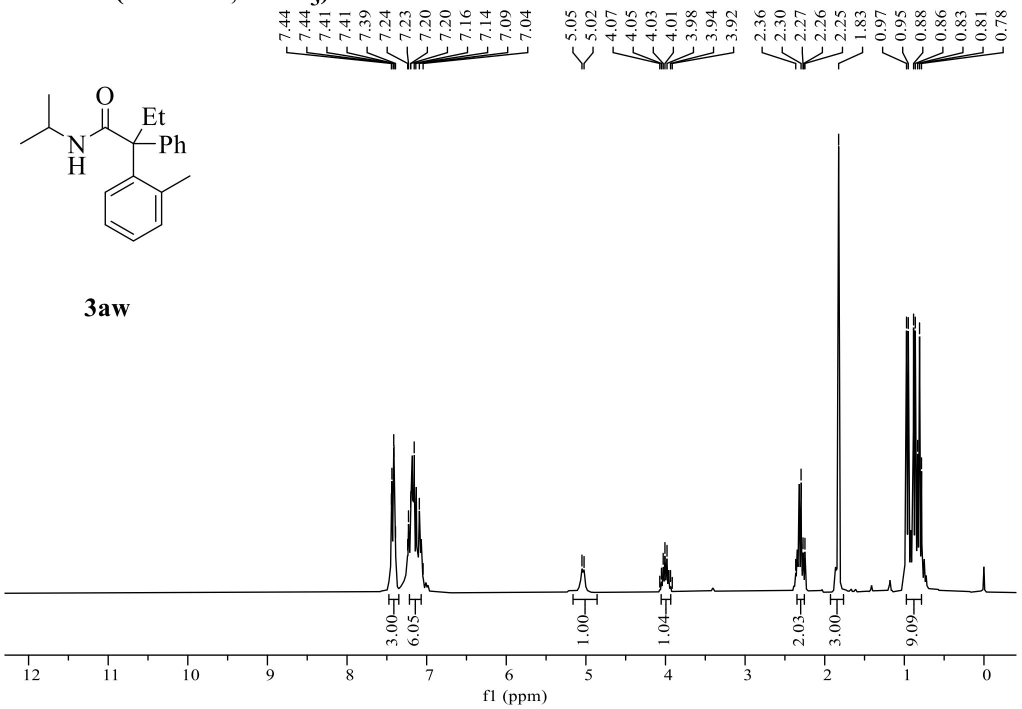

Supplementary Figure 131: ^{1}H NMR (300 MHz, CDCl$_3$) spectrum of compound **3aw**.

^{13}C NMR (75 MHz, CDCl$_3$):

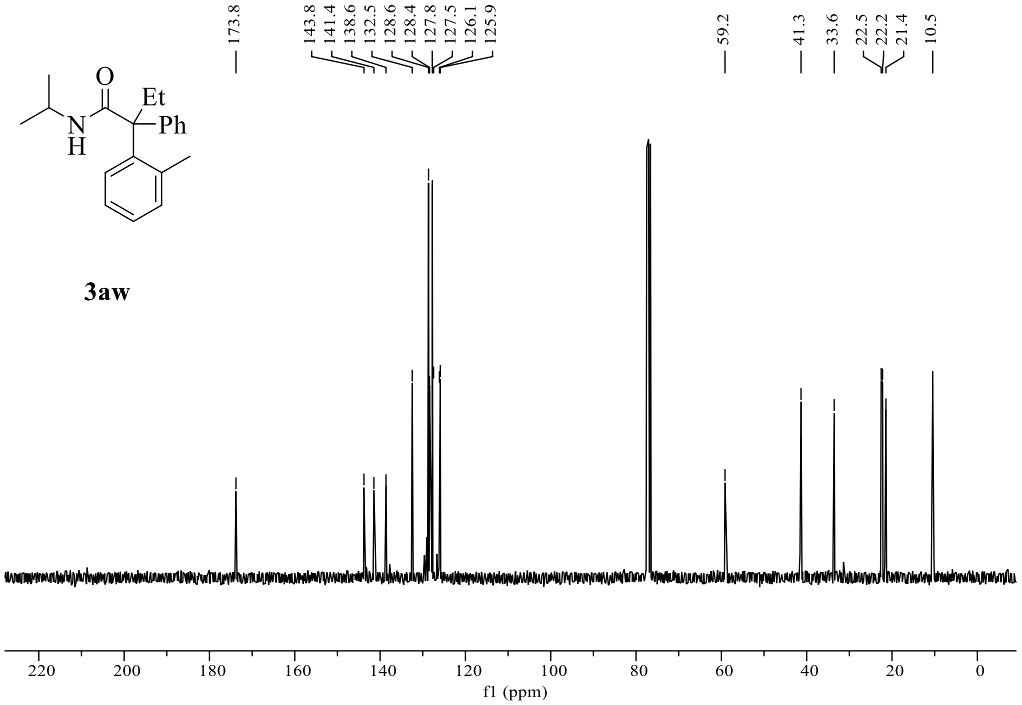

Supplementary Figure 132: ^{13}C NMR (75 MHz, CDCl$_3$) spectrum of compound **3aw**.

^{1}H NMR (300 MHz, CDCl$_3$):

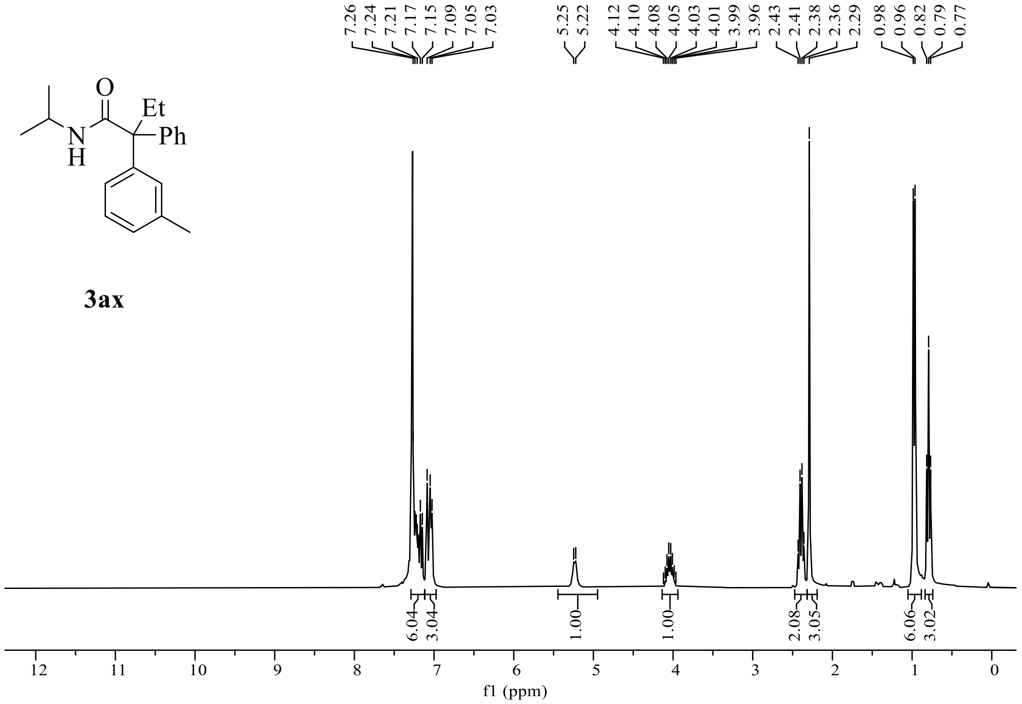

Supplementary Figure 133: ^{1}H NMR (300 MHz, CDCl$_3$) spectrum of compound **3ax**.

^{13}C NMR (75 MHz, CDCl$_3$):

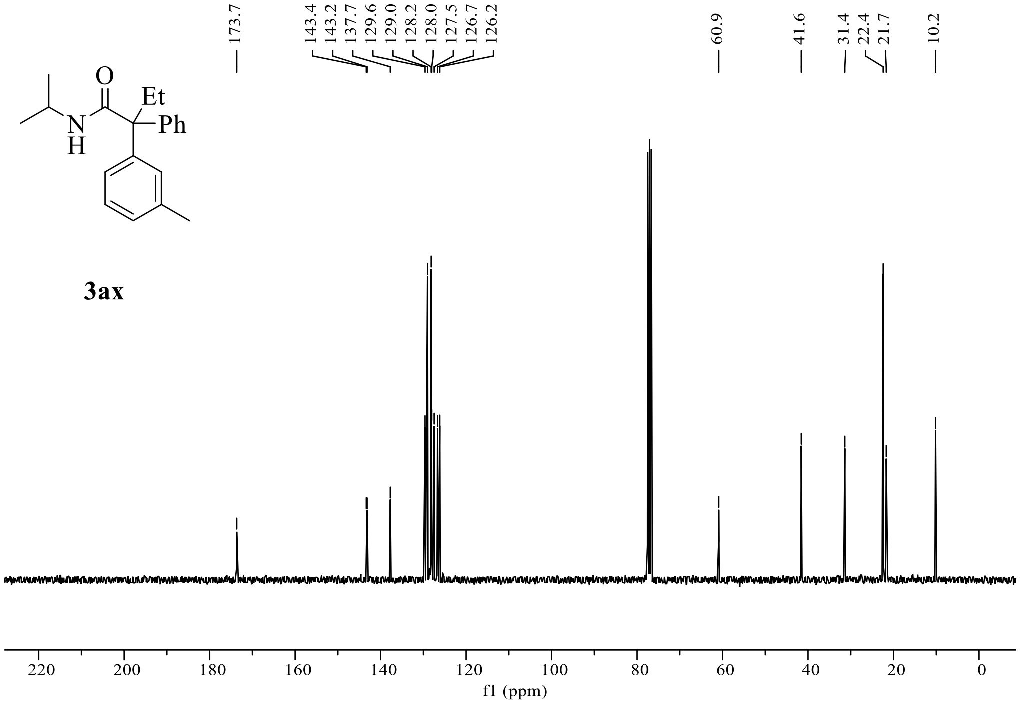

Supplementary Figure 134: ^{13}C NMR (75 MHz, CDCl$_3$) spectrum of compound **3ax**.

^{1}H NMR (600 MHz, CDCl$_3$):

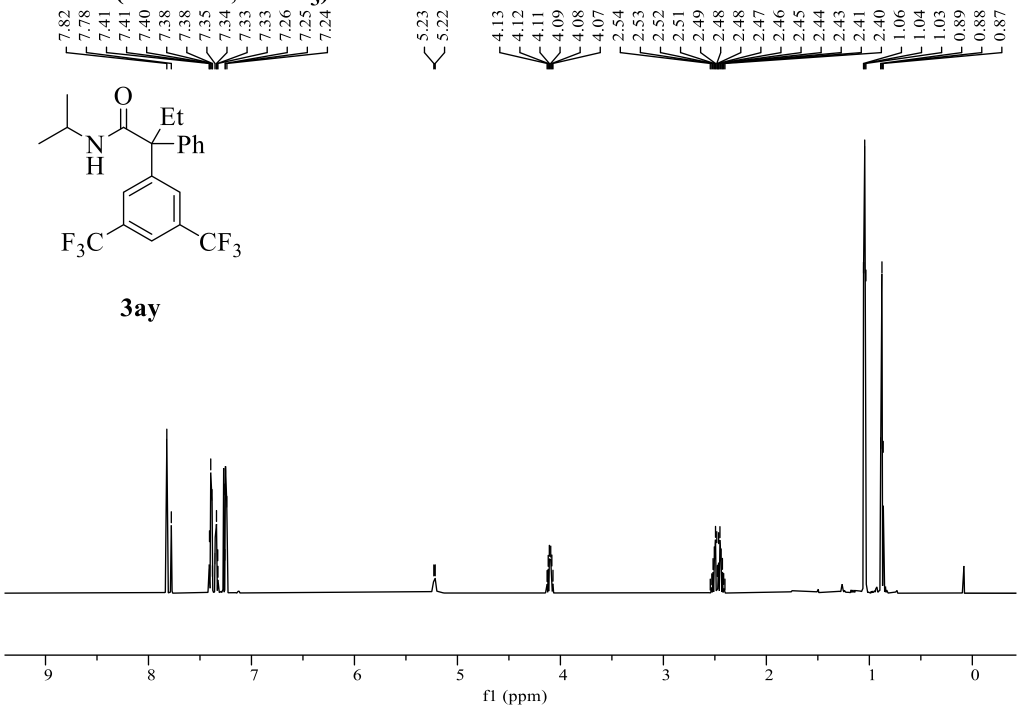

Supplementary Figure 135: ^{1}H NMR (600 MHz, CDCl$_3$) spectrum of compound **3ay**.

^{13}C{^{1}H,^{19}F} NMR (151 MHz, CDCl$_3$):

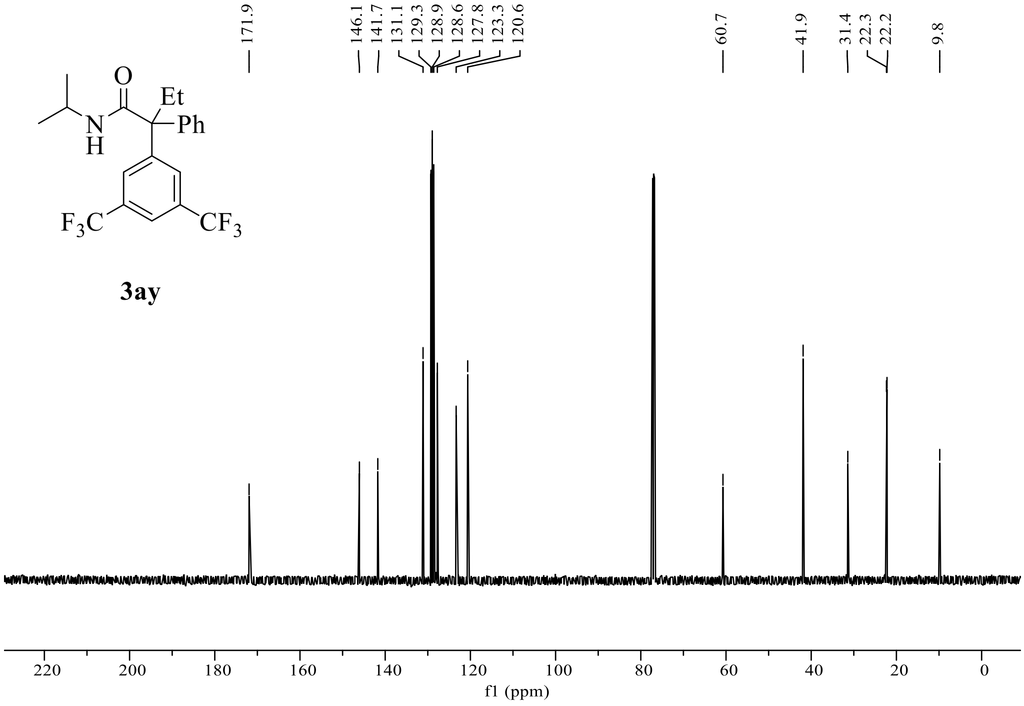

Supplementary Figure 136: ^{13}C{^{1}H,^{19}F} NMR (151 MHz, CDCl$_3$) spectrum of compound **3a**y

$^{19}F\{^{1}H,^{13}C\}$ NMR (564 MHz, $CDCl_3$):

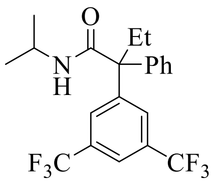

3ay

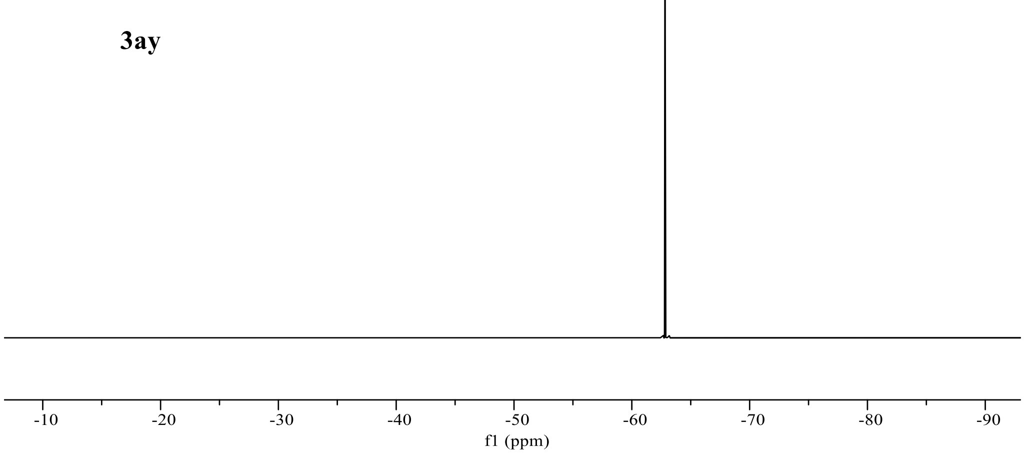

Supplementary Figure 137: $^{19}F\{^{1}H,^{13}C\}$ NMR (564 MHz, $CDCl_3$) spectrum of compound **3ay**.

^{1}H NMR (300 MHz, $CDCl_3$):

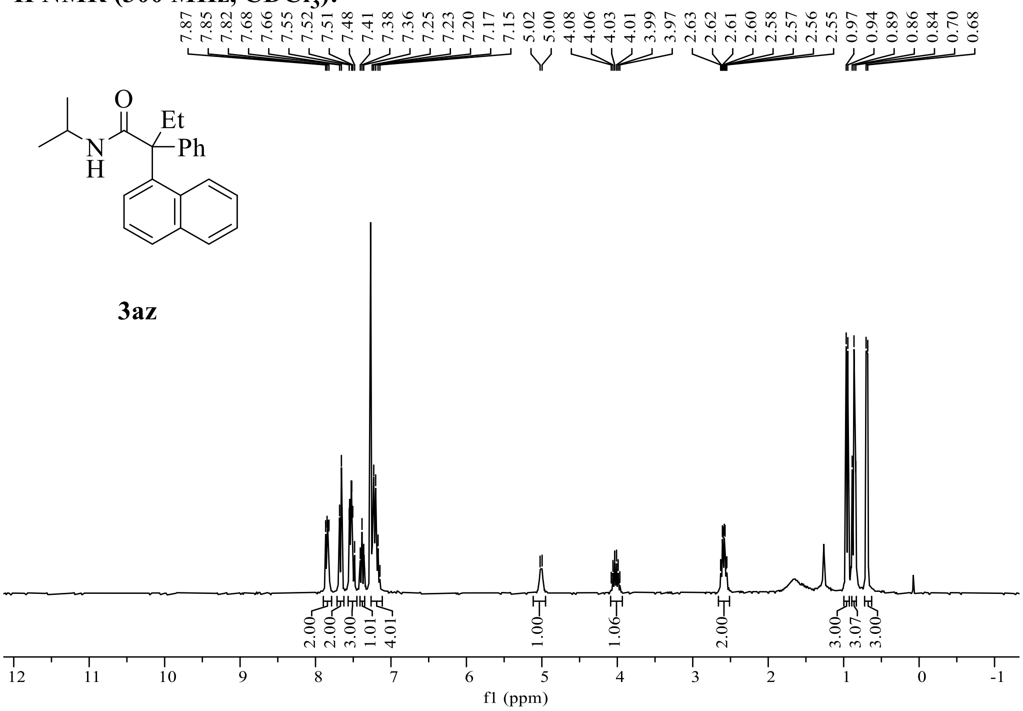

Supplementary Figure 138: ^{1}H NMR (300 MHz, $CDCl_3$) spectrum of compound **3az**.

^{13}C NMR (75 MHz, $CDCl_3$):

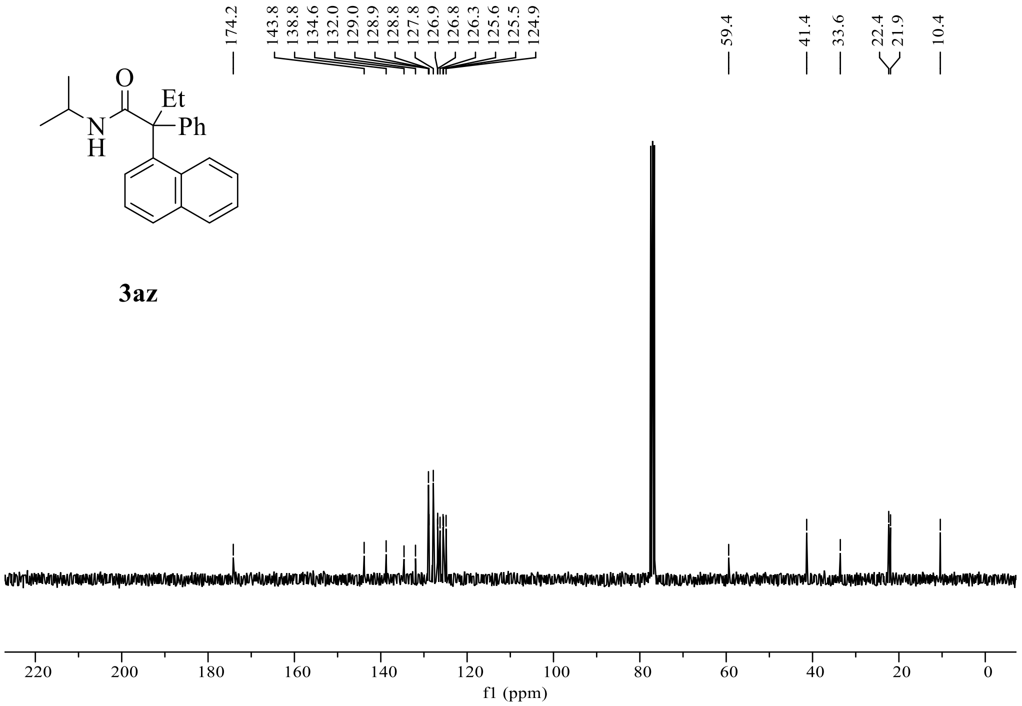

Supplementary Figure 139: ^{13}C NMR (75 MHz, $CDCl_3$) spectrum of compound **3az**.

^{1}H NMR (300 MHz, CDCl$_3$):

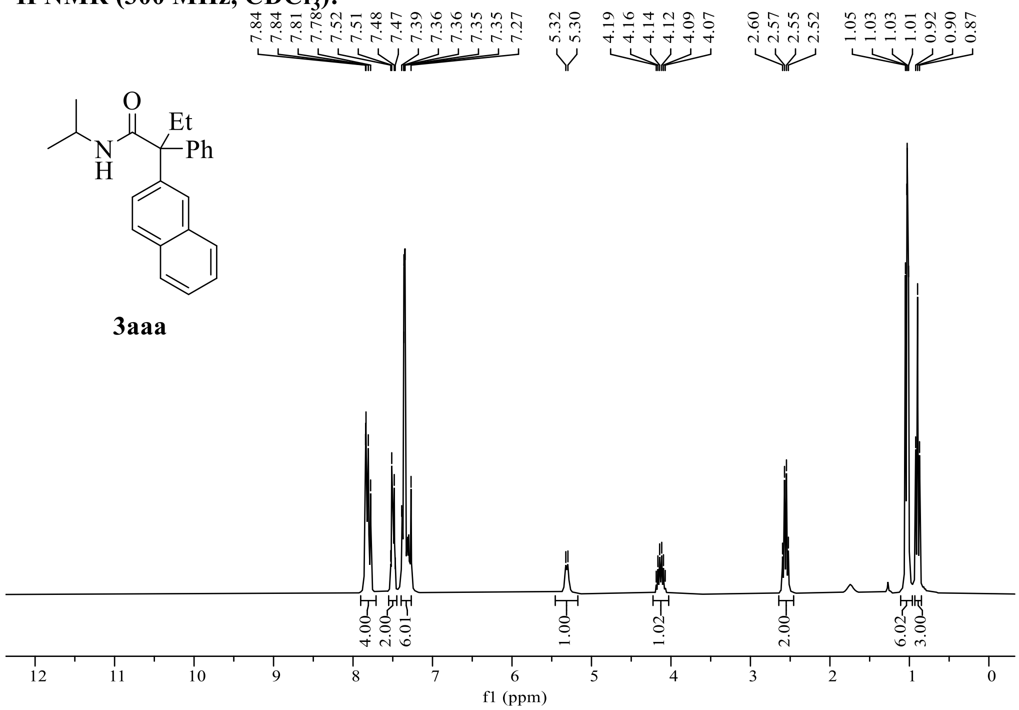

Supplementary Figure 140: ^{1}H NMR (300 MHz, CDCl$_3$) spectrum of compound **3aaa**.

^{13}C NMR (75 MHz, CDCl$_3$):

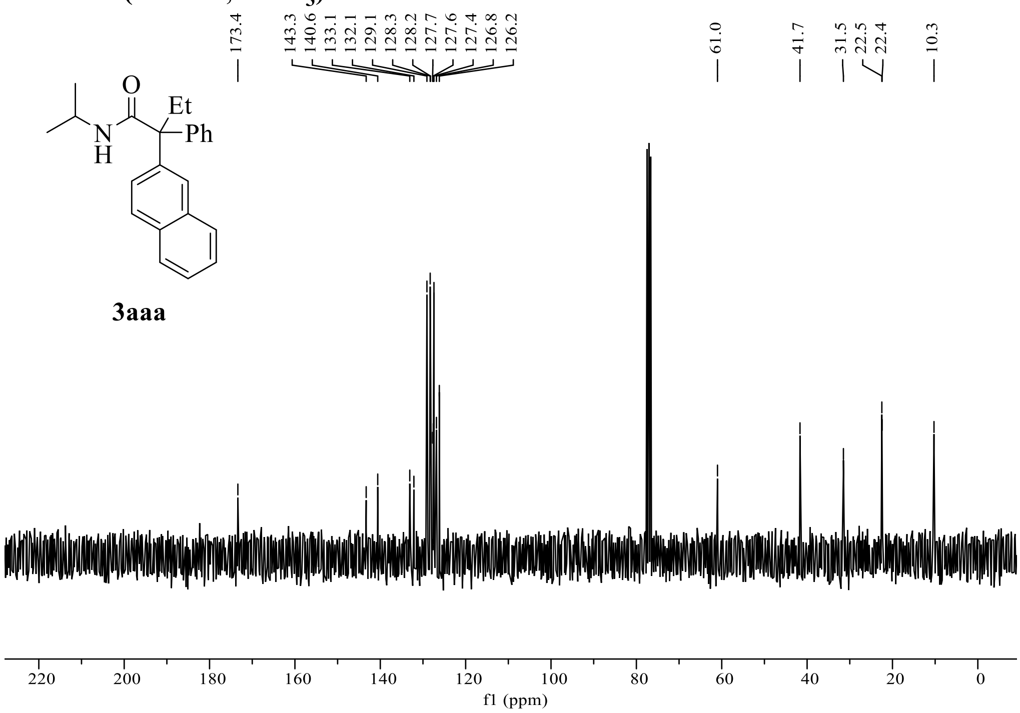

Supplementary Figure 141: ^{13}C NMR (75 MHz, CDCl$_3$) spectrum of compound **3aaa**.

^{1}H NMR (300 MHz, $CDCl_3$):

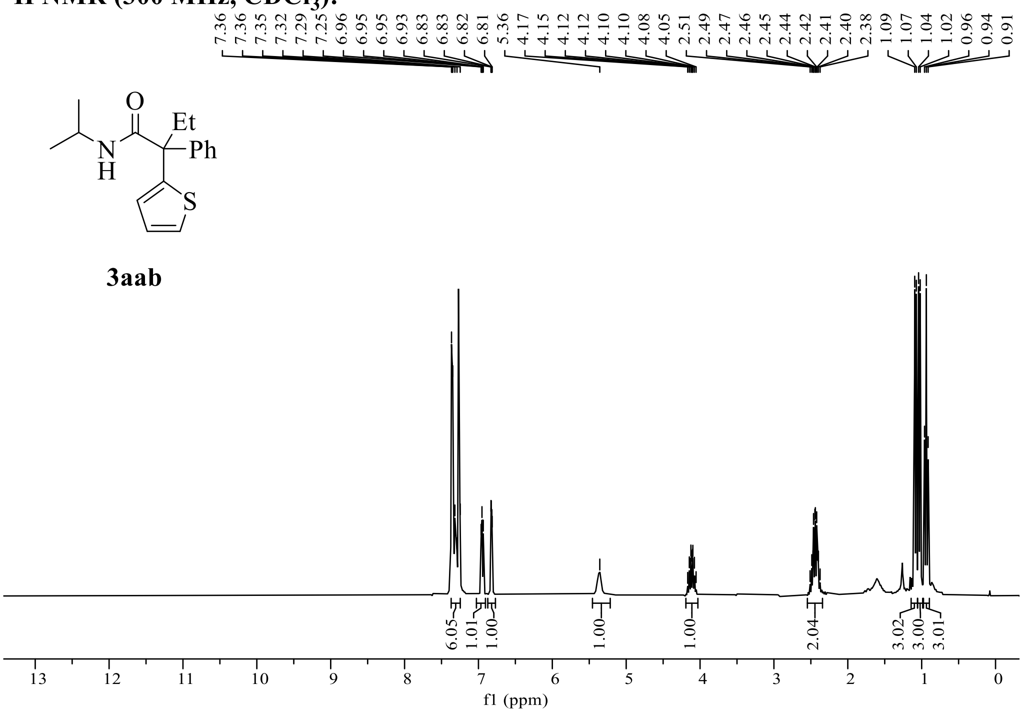

Supplementary Figure 142: ^{1}H NMR (300 MHz, $CDCl_3$) spectrum of compound **3aab**.

^{13}C NMR (75 MHz, $CDCl_3$):

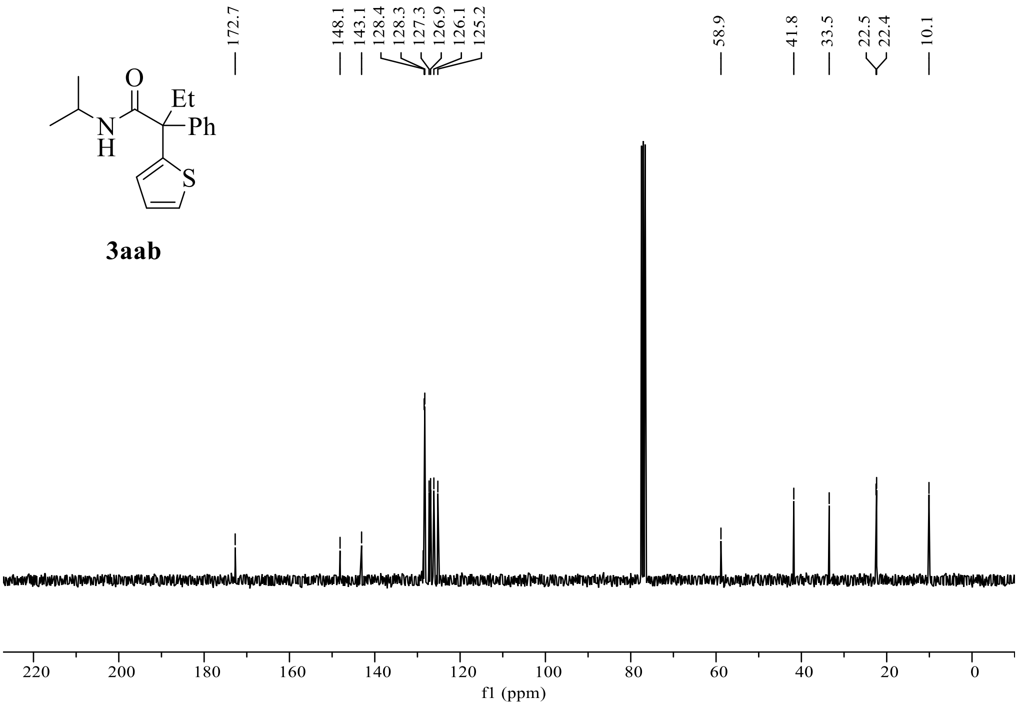

Supplementary Figure 143: ^{13}C NMR (75 MHz, $CDCl_3$) spectrum of compound **3aab**.

^{1}H NMR (300 MHz, $CDCl_3$):

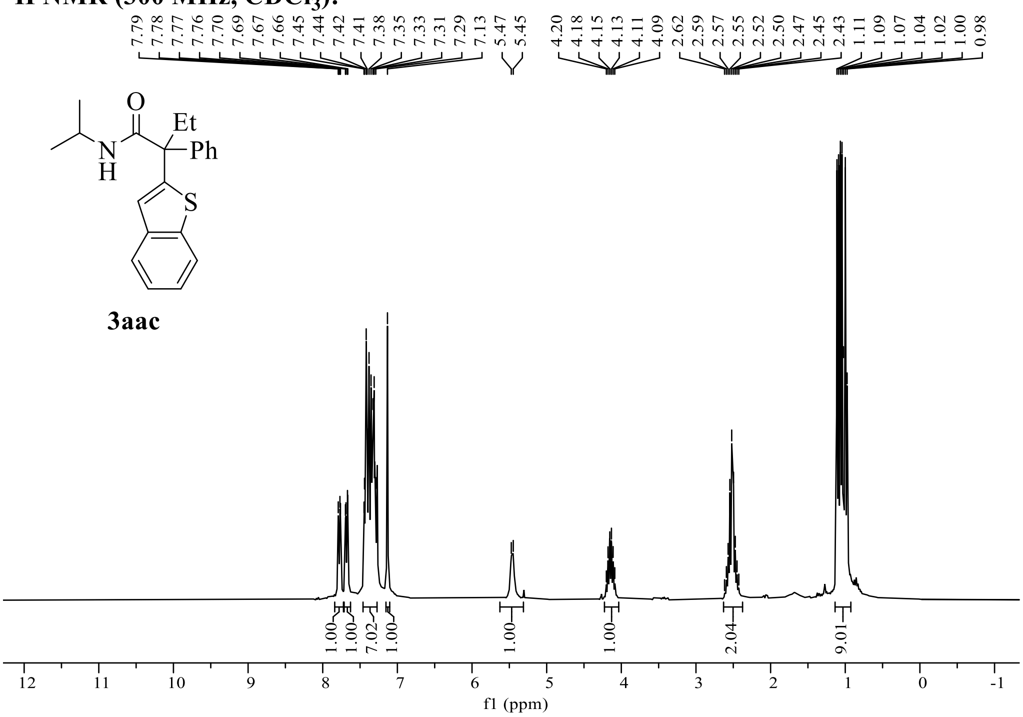

Supplementary Figure 144: ^{1}H NMR (300 MHz, $CDCl_3$) spectrum of compound **3aac**.

^{13}C NMR (75 MHz, $CDCl_3$):

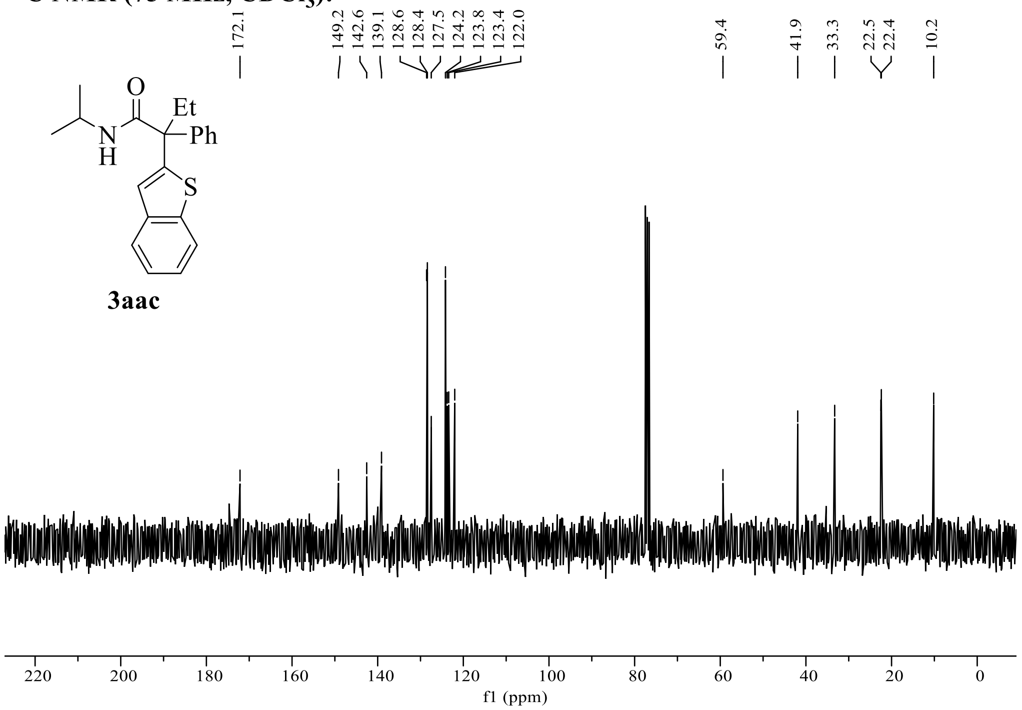

Supplementary Figure 145: ^{13}C NMR (75 MHz, $CDCl_3$) spectrum of compound **3aac**.

^{1}H NMR (300 MHz, $CDCl_3$):

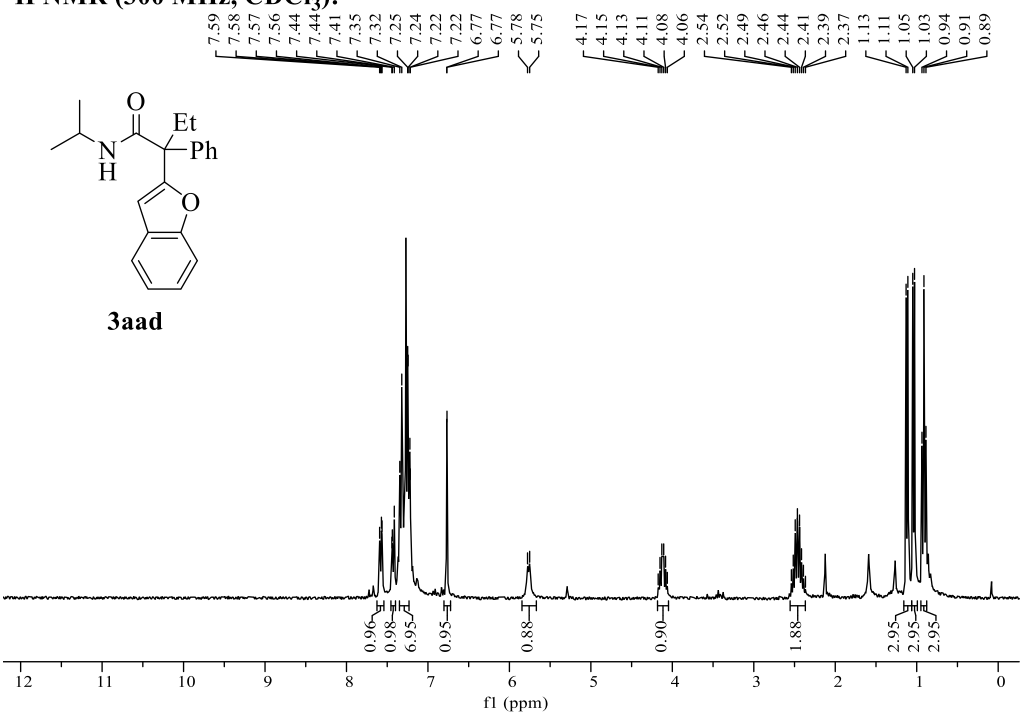

Supplementary Figure 146: ^{1}H NMR (300 MHz, $CDCl_3$) spectrum of compound **3aad**.

^{13}C NMR (126 MHz, $CDCl_3$):

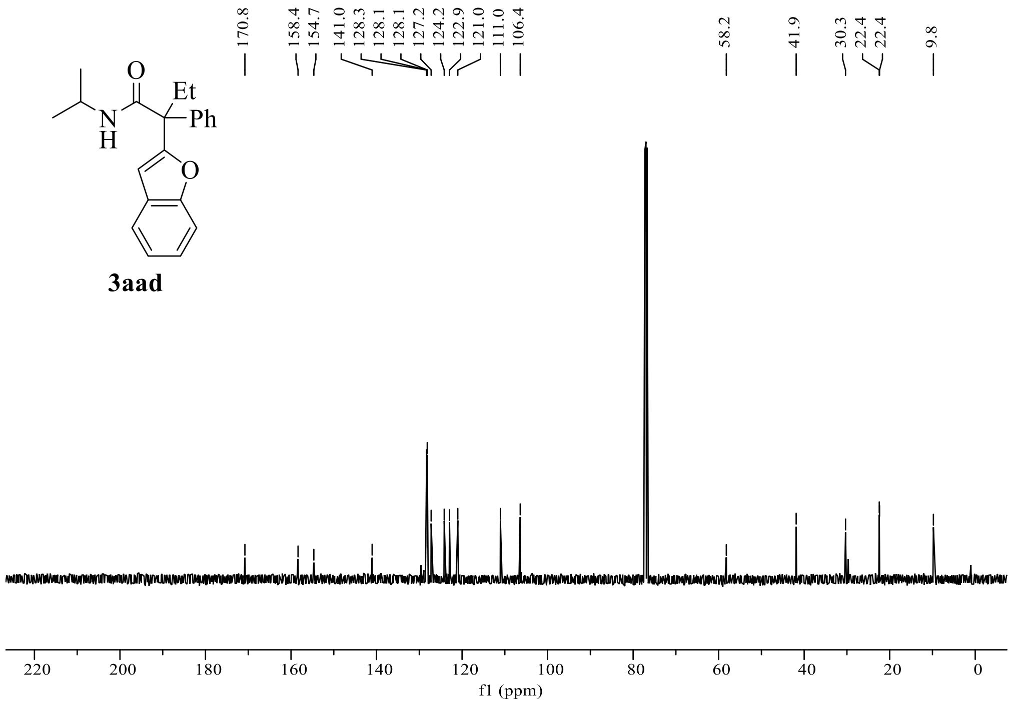

Supplementary Figure 147: ^{13}C NMR (126 MHz, $CDCl_3$) spectrum of compound **3aad**.

^{1}H NMR (300 MHz, $CDCl_3$):

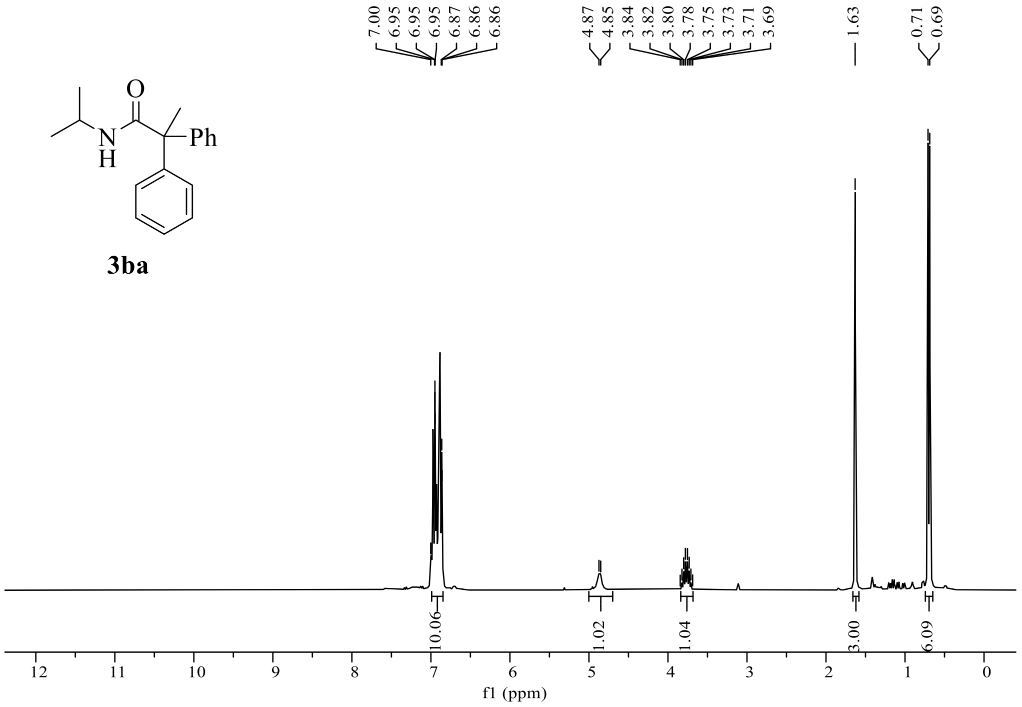

Supplementary Figure 148: ^{1}H NMR (300 MHz, $CDCl_3$) spectrum of compound **3ba**.

^{13}C NMR (75 MHz, $CDCl_3$):

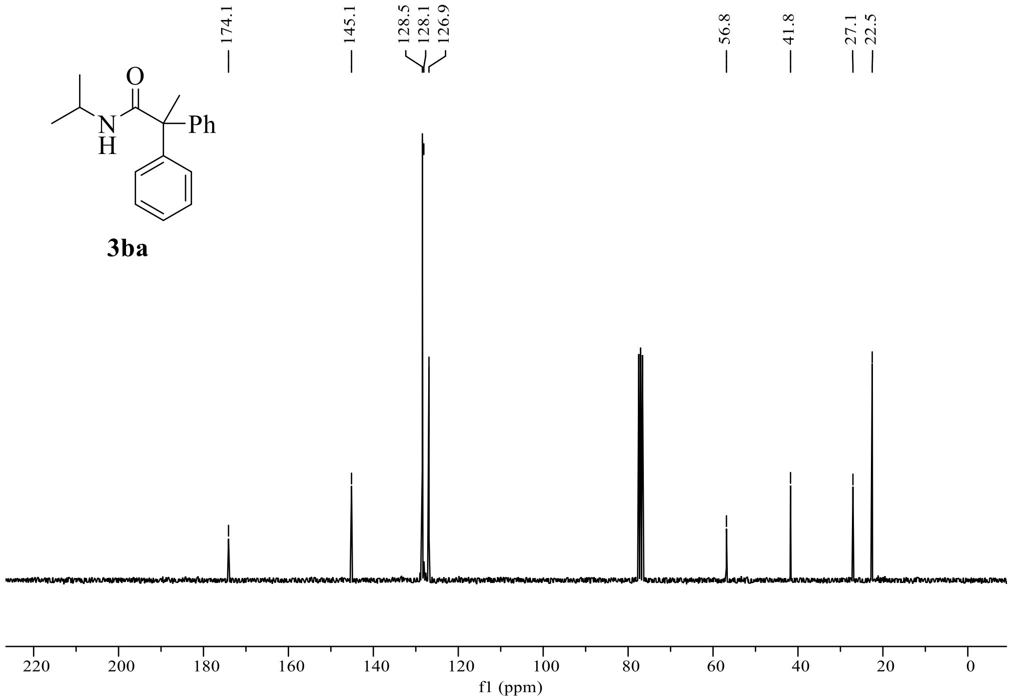

Supplementary Figure 149: ^{13}C NMR (75 MHz, $CDCl_3$) spectrum of compound **3ba**.

^{1}H NMR (300 MHz, CDCl$_3$):

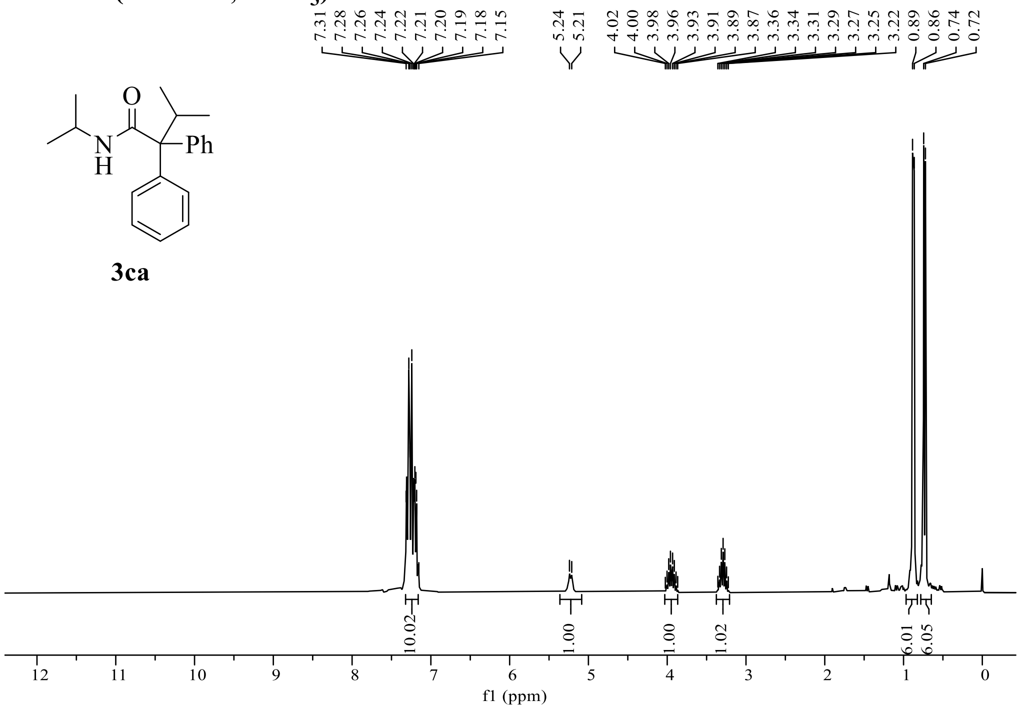

Supplementary Figure 150: ^{1}H NMR (300 MHz, CDCl$_3$) spectrum of compound **3ca**.

^{13}C NMR (75 MHz, CDCl$_3$):

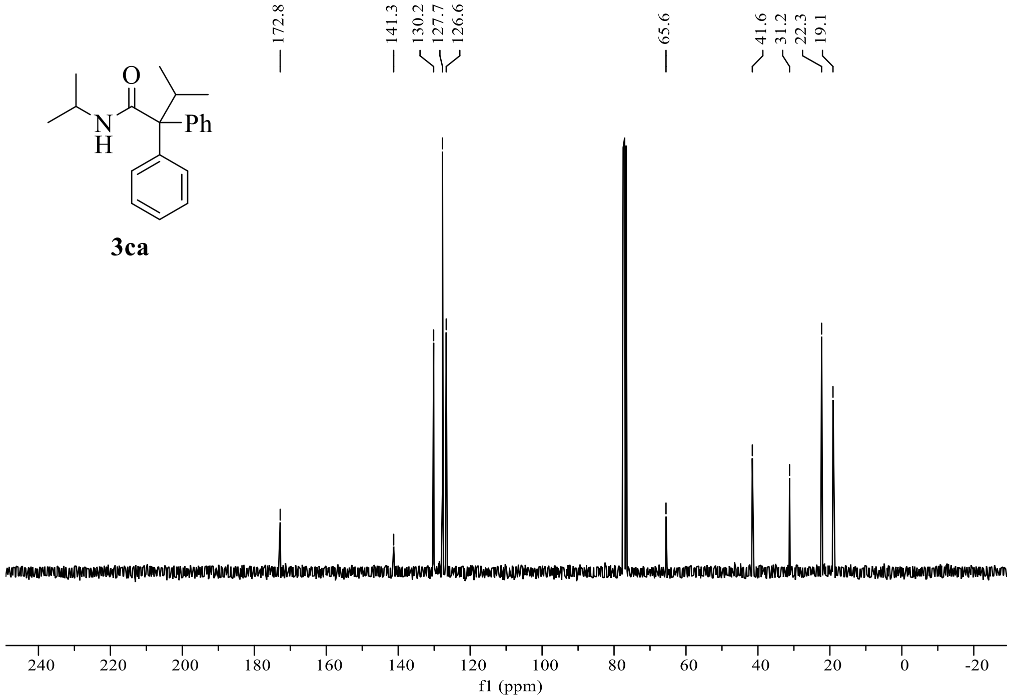

Supplementary Figure 151: ^{13}C NMR (75 MHz, CDCl$_3$) spectrum of compound **3ca**.

^{1}H NMR (300 MHz, $CDCl_3$):

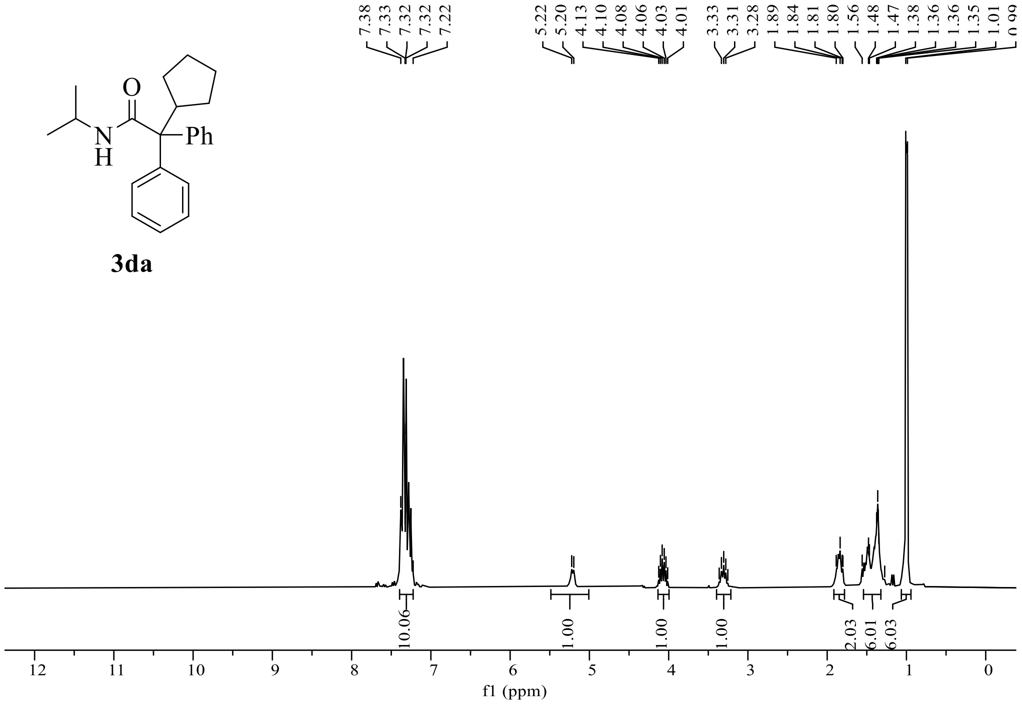

Supplementary Figure 152: ^{1}H NMR (300 MHz, $CDCl_3$) spectrum of compound **3da**.

^{13}C NMR (75 MHz, $CDCl_3$):

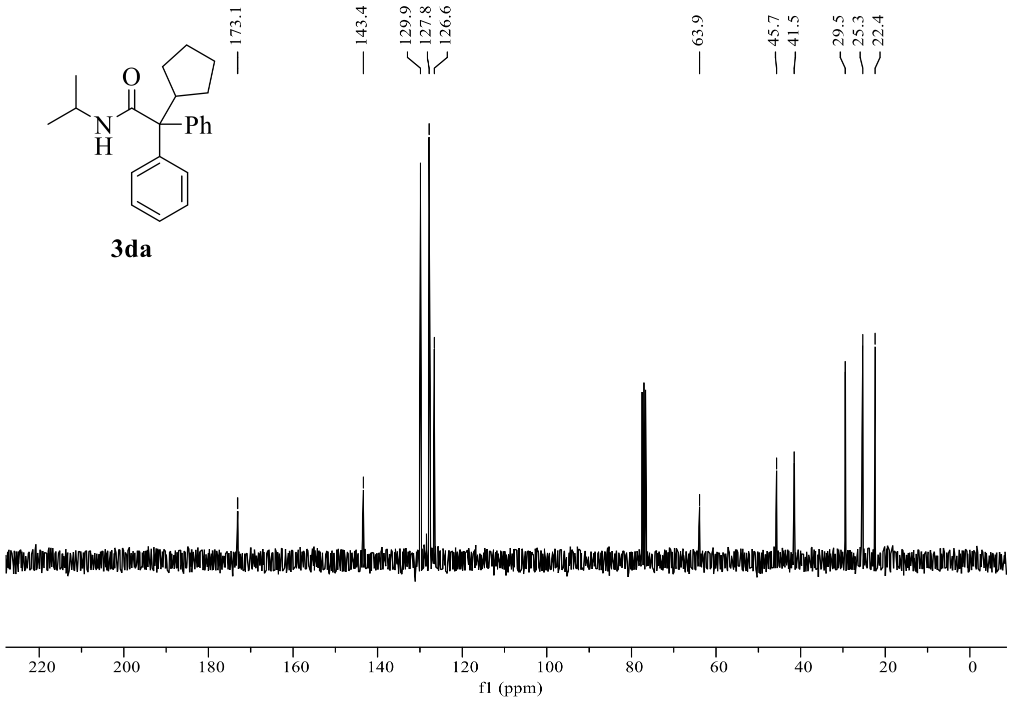

Supplementary Figure 153: ^{13}C NMR (75 MHz, $CDCl_3$) spectrum of compound **3da**.

^{1}H NMR (300 MHz, $CDCl_3$):

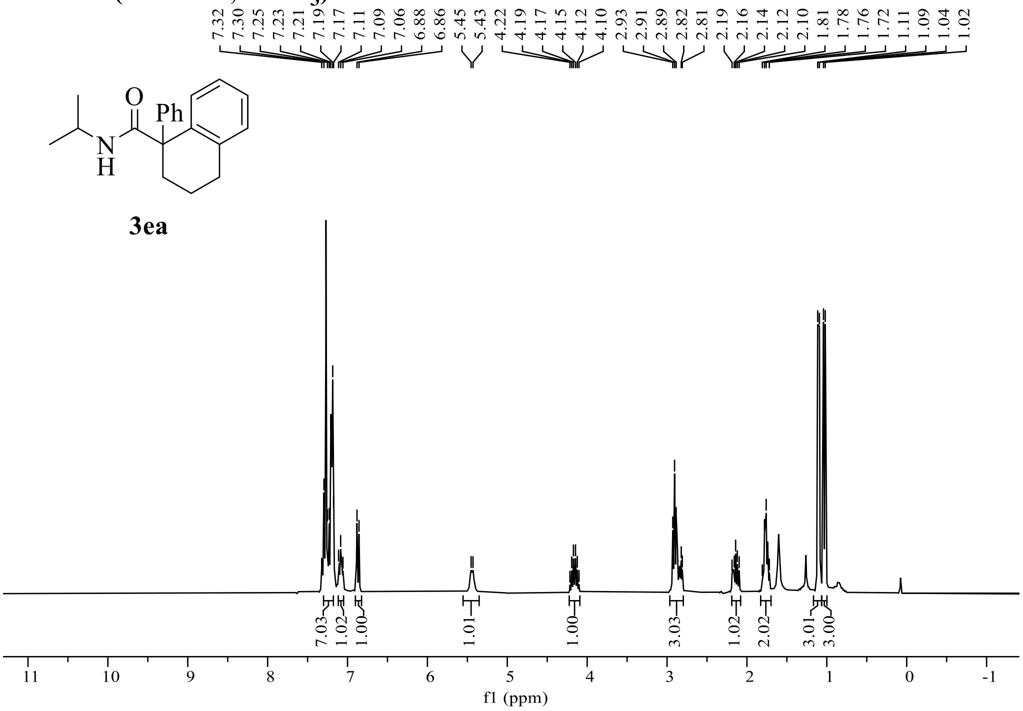

Supplementary Figure 154: ^{1}H NMR (300 MHz, $CDCl_3$) spectrum of compound **3ea**.

^{13}C NMR (75 MHz, $CDCl_3$):

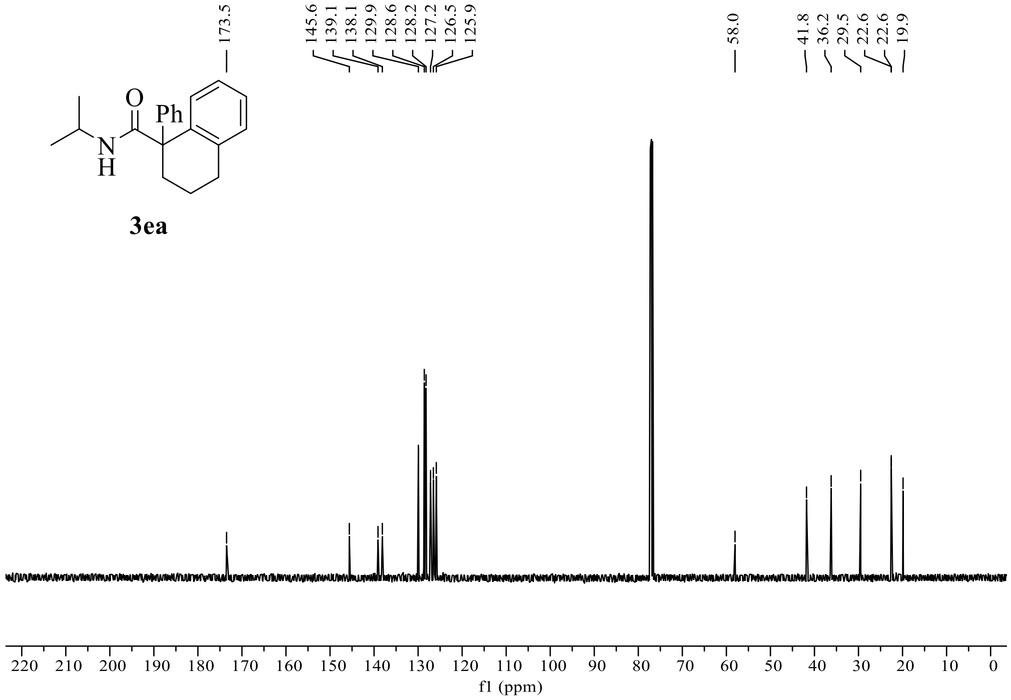

Supplementary Figure 155: ^{13}C NMR (75 MHz, $CDCl_3$) spectrum of compound **3ea**.

^{1}H NMR (300 MHz, $CDCl_3$):

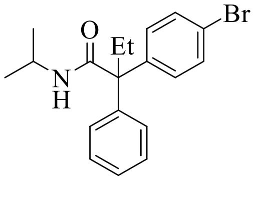

3fa

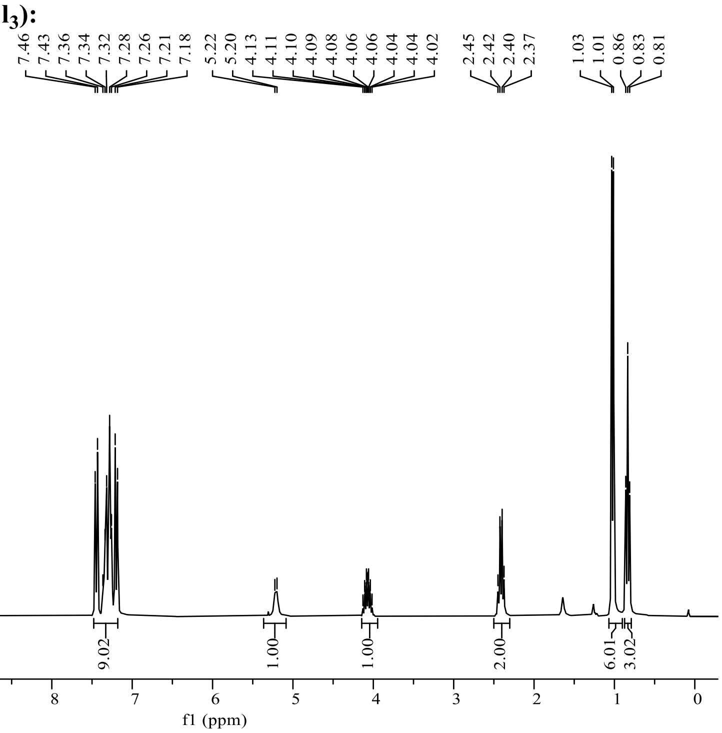

Supplementary Figure 156: ^{1}H NMR (300 MHz, $CDCl_3$) spectrum of compound **3fa**.

^{13}C NMR (75 MHz, $CDCl_3$):

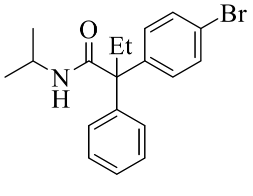

3fa

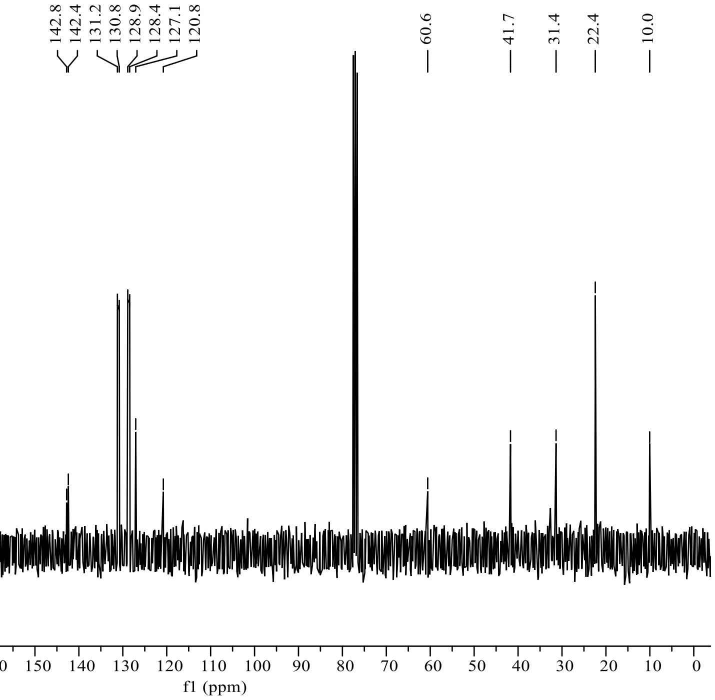

Supplementary Figure 157: ^{13}C NMR (75 MHz, $CDCl_3$) spectrum of compound **3fa**.

^{1}H NMR (300 MHz, $CDCl_3$):

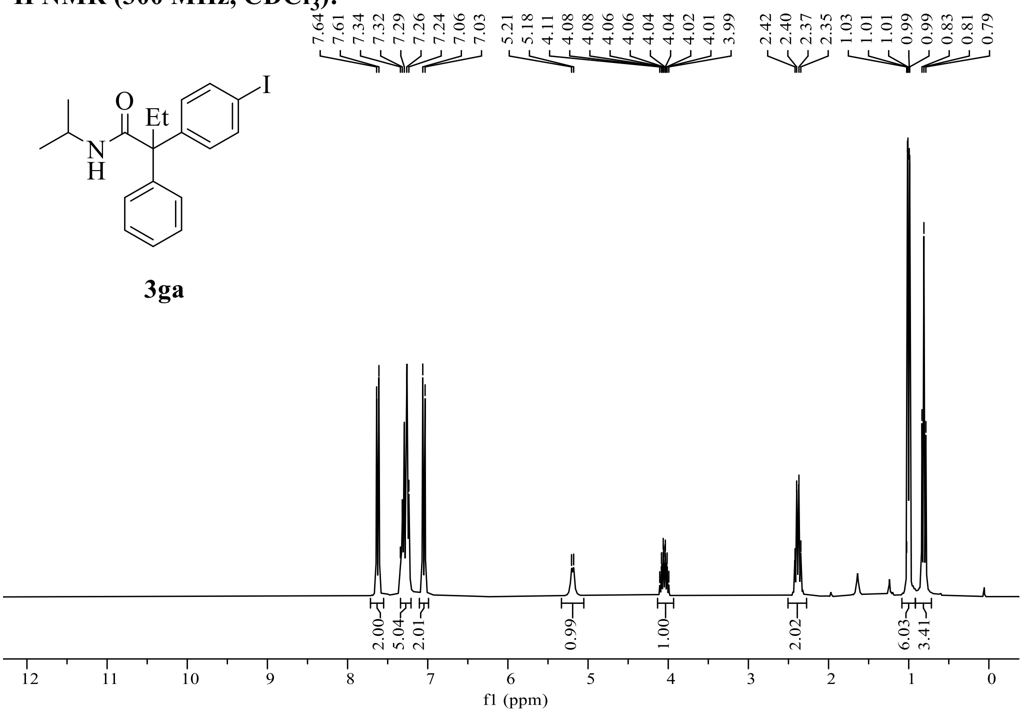

Supplementary Figure 158: ^{1}H NMR (300 MHz, $CDCl_3$) spectrum of compound **3ga**.

^{13}C NMR (75 MHz, $CDCl_3$):

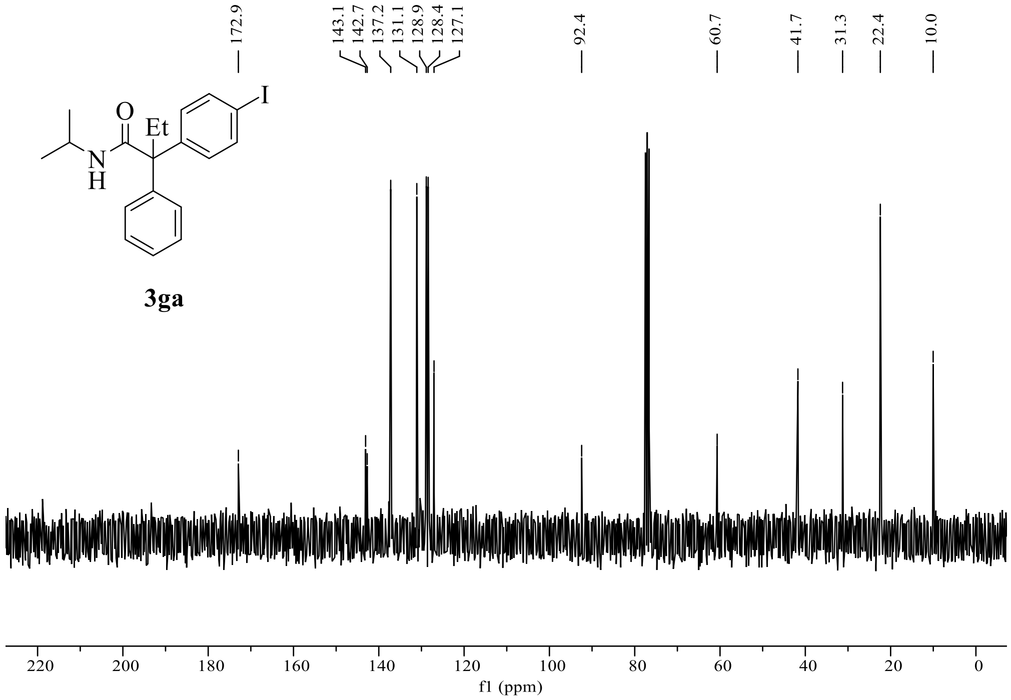

Supplementary Figure 159: ^{13}C NMR (75 MHz, $CDCl_3$) spectrum of compound **3ga**.

^{1}H NMR (300 MHz, $CDCl_3$):

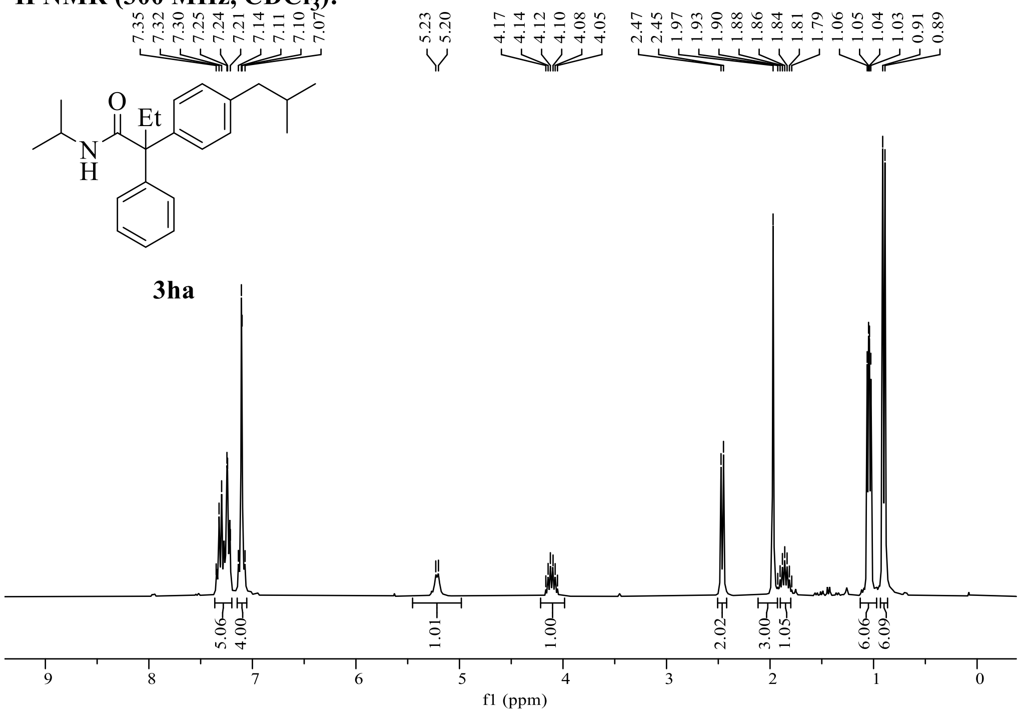

Supplementary Figure 160: ^{1}H NMR (300 MHz, $CDCl_3$) spectrum of compound **3ha**.

^{13}C NMR (75 MHz, $CDCl_3$):

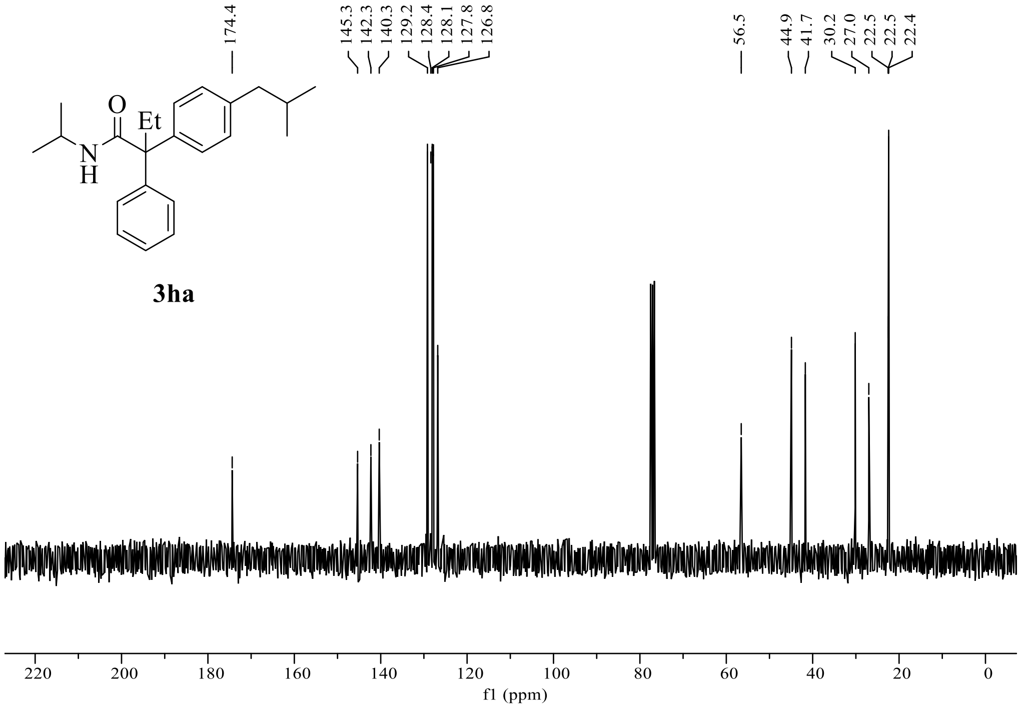

Supplementary Figure 161: ^{13}C NMR (75 MHz, $CDCl_3$) spectrum of compound **3ha**.

^{1}H NMR (600 MHz, $CDCl_3$):

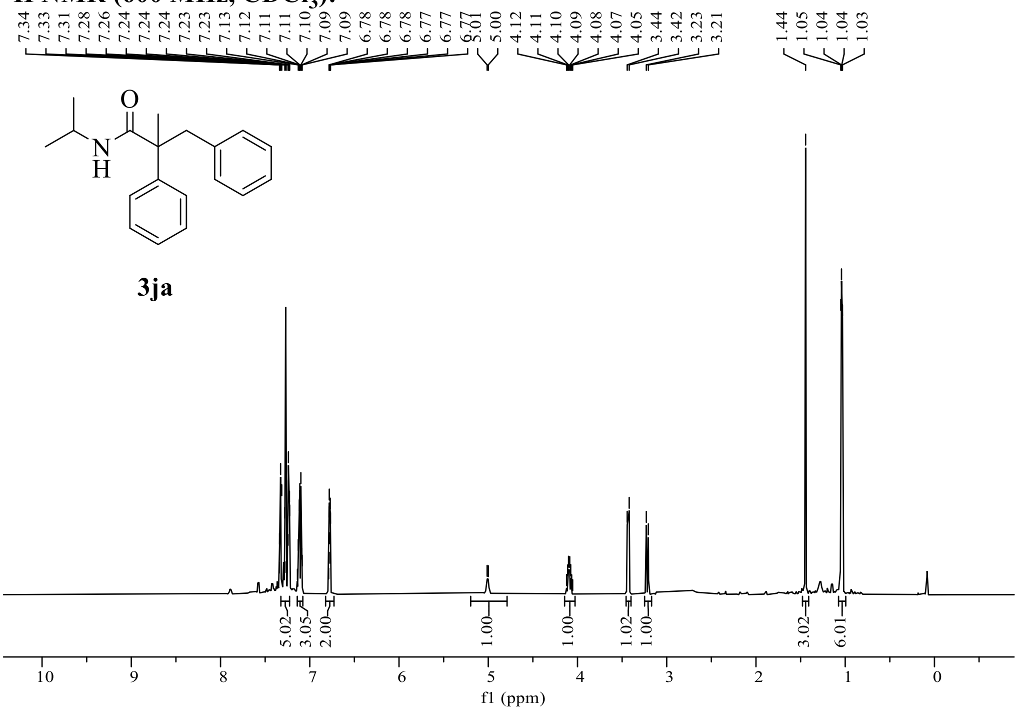

Supplementary Figure 162: ^{1}H NMR (600 MHz, $CDCl_3$) spectrum of compound **3ja**.

^{13}C NMR (151 MHz, $CDCl_3$):

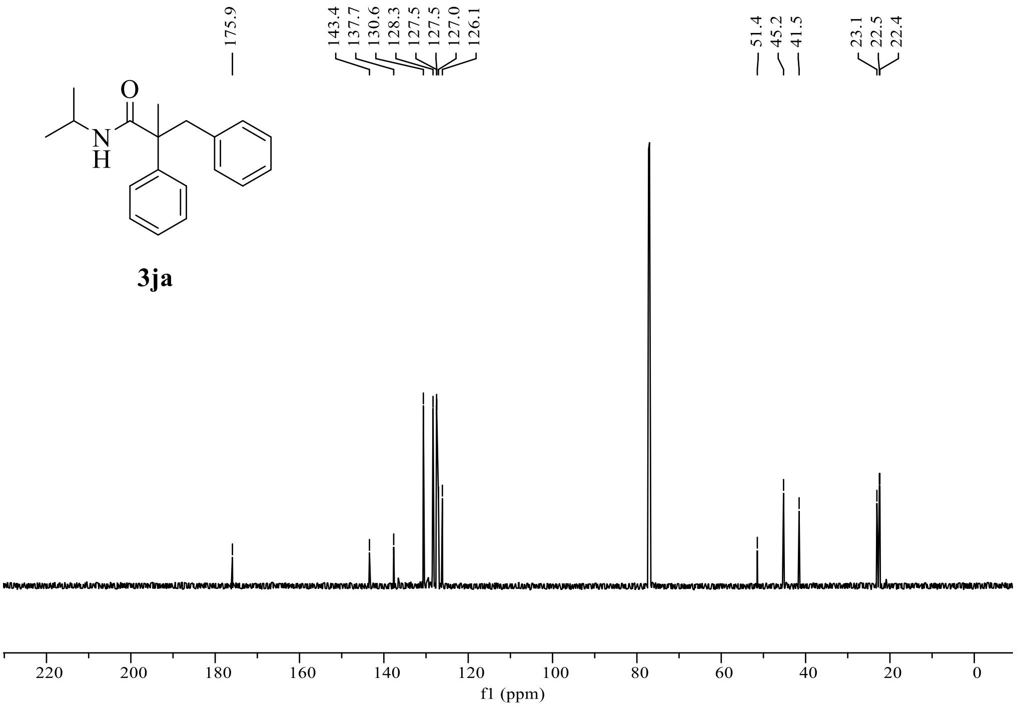

Supplementary Figure 163: ^{13}C NMR (151 MHz, $CDCl_3$) spectrum of compound **3ja**.

^{1}H NMR (300 MHz, $CDCl_3$):

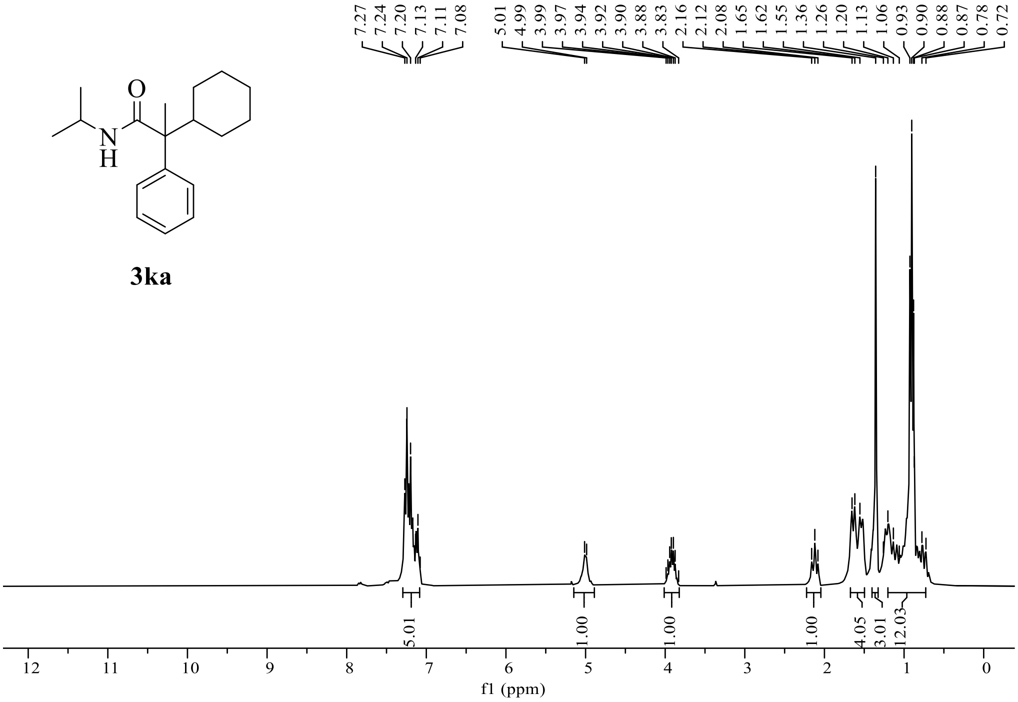

Supplementary Figure 164: ^{1}H NMR (300 MHz, $CDCl_3$) spectrum of compound **3ka**.

^{13}C NMR (75 MHz, $CDCl_3$):

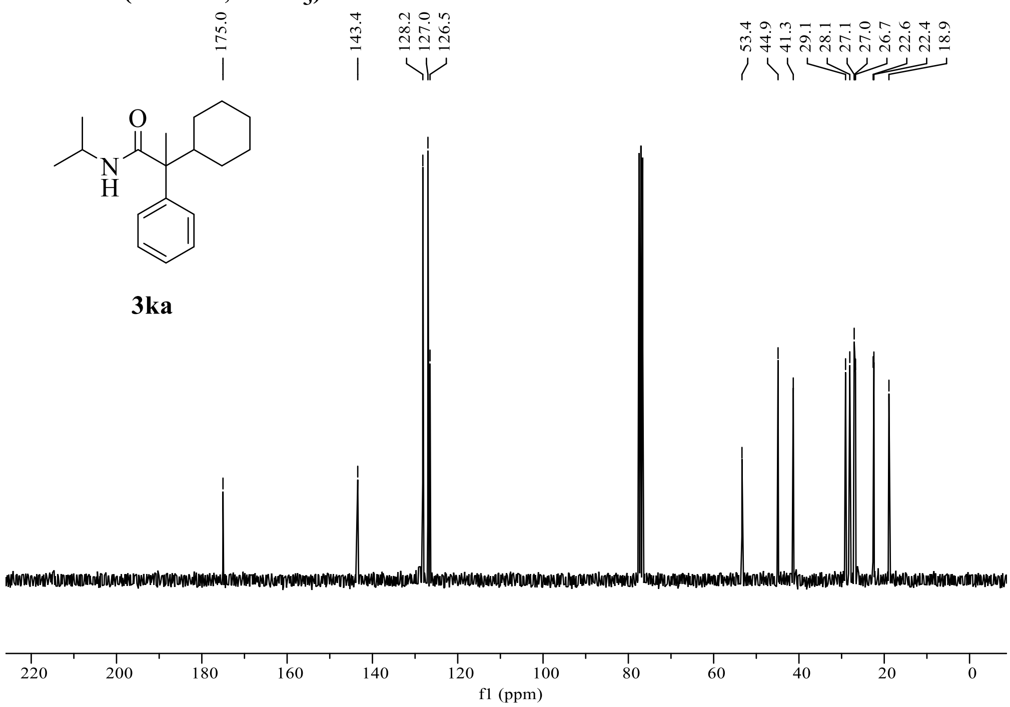

Supplementary Figure 165: ^{13}C NMR (75 MHz, $CDCl_3$) spectrum of compound **3ka**.

^{1}H NMR (300 MHz, $CDCl_3$):

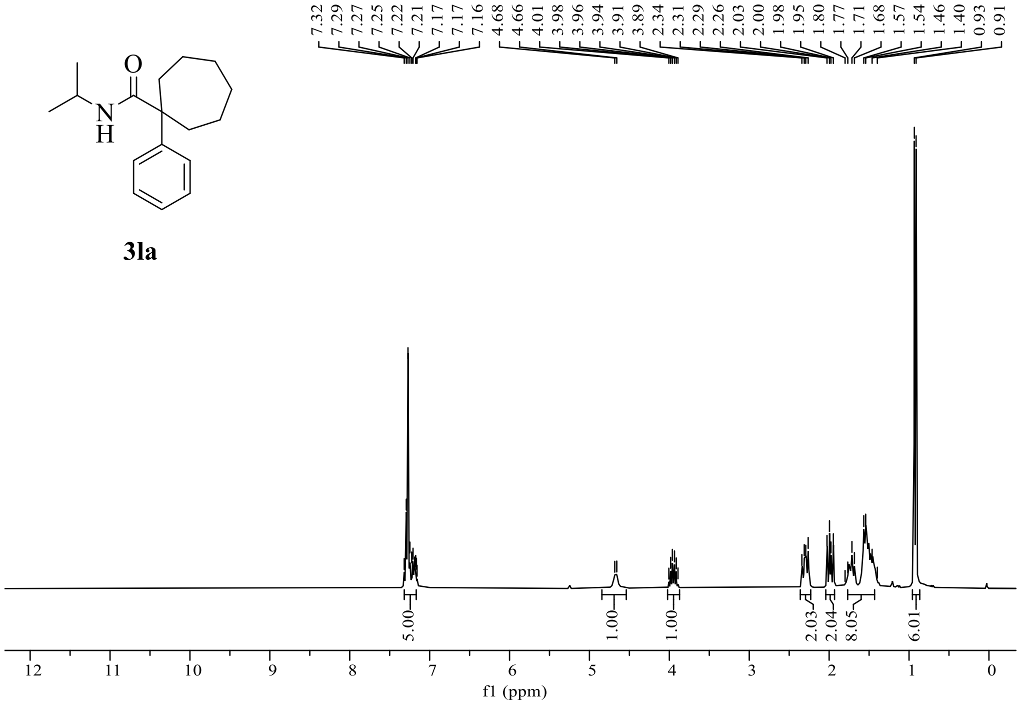

Supplementary Figure 166: ^{1}H NMR (300 MHz, $CDCl_3$) spectrum of compound **3la**.

^{13}C NMR (75 MHz, $CDCl_3$):

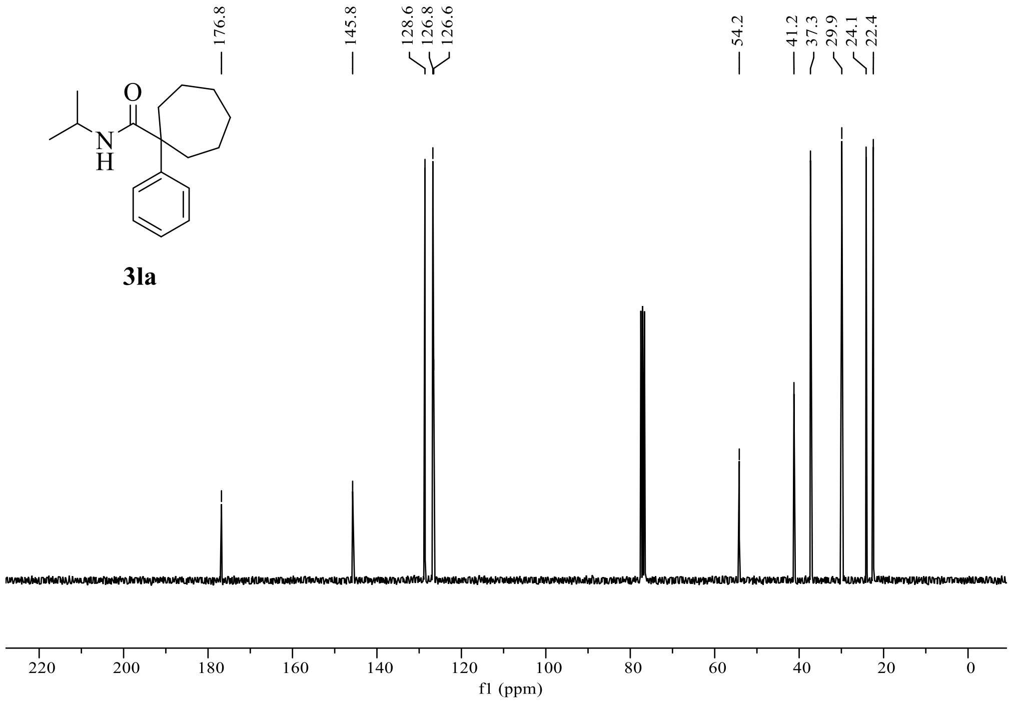

Supplementary Figure 167: ^{13}C NMR (75 MHz, $CDCl_3$) spectrum of compound **3la**.

^{1}H NMR (300 MHz, $CDCl_3$):

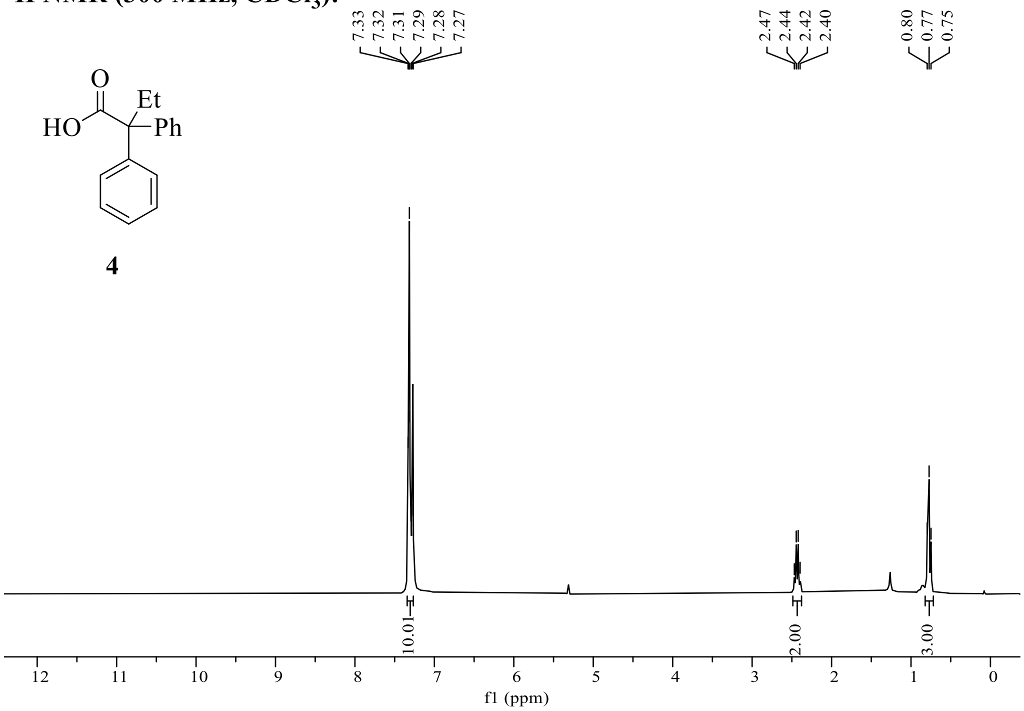

Supplementary Figure 168: ^{1}H NMR (300 MHz, $CDCl_3$) spectrum of compound **4**.

^{13}C NMR (151 MHz, $CDCl_3$):

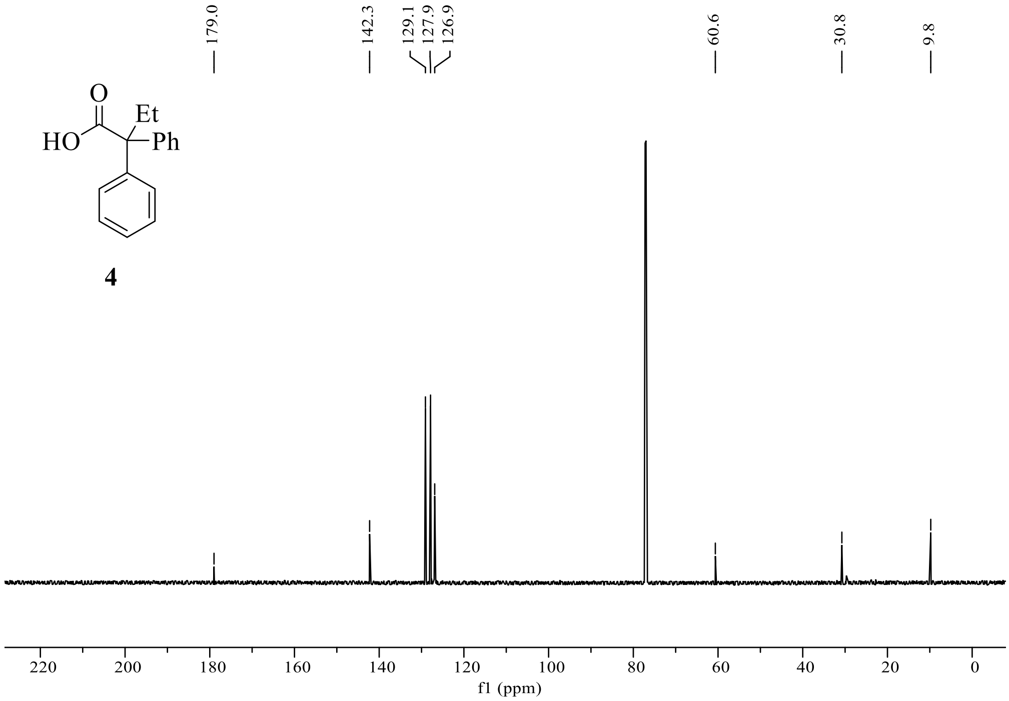

Supplementary Figure 169: ^{13}C NMR (151 MHz, $CDCl_3$) spectrum of compound **4**.

3. Supplementary References

1. Vogler, T. & Studer, A. Rhodium-Catalyzed Oxidative Homocoupling of Boronic Acids. *Adv. Synth. Catal.* **13,** 1963–1967 (2008).
2. Niu, B. *et al.* Synthesis of Nitromethyl-Substituted Oxindole Derivatives via a Desulfonylation Cascade. *Synlett* **26,** 635–638 (2015).
3. Baigrie, L. M., Seiklay, H. R. & Tidwell, T. T. Stereospecific formation of enolates from reaction of unsymmetrical ketenes and organolithium reagents. *J. Am. Chem. Soc.* **107,** 5391–5396 (1985).
4. Connelly, N. G. & Geiger, W. E. Chemical redox agents for organometallic chemistry. *Chem. Rev.* **96,** 877–910 (1996).
5. Saidykhan, A., Bowen, R. D., Gallagher, R. T. & Martin, W. Intramolecular NC rearrangements involving sulfonamide protecting groups. *Tetrahedron Lett.* **56,** 66–68 (2015).
6. Moriyama, K., Nakamura, Y. & Togo, H. Oxidative debenzylation of N-benzyl amides and O-benzyl ethers using alkali metal bromide. *Org. Lett.* **16,** 3812–3815 (2014).
7. Rattanaburi, P., Khumraksa, B. & Pattarawarapan, M. Synthesis and applications of Fe3O4-diisopropylaminoacetamide as a versatile and reusable magnetic nanoparticle supported N,N-diisopropylethylamine equivalent. *Tetrahedron Lett.* **53,** 2689–2693 (2012).
8. Jie, Y. *et al.* An acyclic trialkylamine virtually planar at nitrogen. Some chemical consequences of nitrogen planarity. *J. Org. Chem.* **75,** 4472–4479 (2010).
9. Feng, J.-B. & Wu, X.-F. A general iodine-mediated synthesis of primary sulfonamides from thiols and aqueous ammonia. *Org. Biomol. Chem.* **14,** 6951–6954 (2016).
10. Sarkar, D., Ghosh, M. K. & Rout, N. PTAB mediated open air synthesis of sulfonamides, thiosulfonates and symmetrical disulfanes. *Tetrahedron Lett.* **59,** 2360–2364 (2018).
11. Ikawa, T. *et al.* Synthesis of Optically Active 2,3-Disubstituted Indoline -Derivatives through Cycloaddition Reactions between Benzynes and α,β-Unsaturated γ-Aminobutyronitriles. *Synlett* **29,** 530–536 (2018).
12. Morales, M. R., Mellem, K. T. & Myers, A. G. Pseudoephenamine: A Practical Chiral Auxiliary for Asymmetric Synthesis. *Angew. Chem. Int. Ed.* **124,** 4646–4649 (2012).
13. Gediya, S. K., Clarkson, G. J. & Wills, M. Asymmetric Transfer Hydrogenation: Dynamic Kinetic Resolution of α-Amino Ketones. *J. Org. Chem.* **85,** 11309–11330 (2020).
14. Hashimoto, Y., Takaoki, K., Sudo, A., Ogasawara, T. & Saigo, K. Stereoselective Addition Reaction of Organolithium Reagents to Chiral Imines Derived from erythro -2-Amino-1,2-diphenylethanol. *Chem. Lett.* **24,** 235–236 (1995).
15. Ishii, K., Aoki, S. & Koga, K. Enantioselective methylation of the lithium enolate of 1-tetralone mediated by chiral C2-symmetric DMEU derivatives. *Tetrahedron Lett.* **38,** 563–566 (1997).
16. Meyer, A. U., Berger, A. L. & König, B. Metal-free C-H sulfonamidation of pyrroles by visible light photoredox catalysis. *Chem. Commun.* **52,** 10918–10921 (2016).
17. Nozu, R., Osaka, T., Kitano, H. & Fukui, K.-I. Studies on Aromatic Fluorine Compounds. I. Preparations of p-Fluorobenzensulfonic Acid and Related Compounds. *Nippon Kagaku Zasshi* **76,** 775 (1955).
18. Yu, T. T., Qi, L.-J., Cui, D.-M., Zhang, C. & Zhao, Y. N-alkylation of sulfonamides with alcohols by Tf2O. *Bull. Chem. Soc. Jpn.* **88,** 610–612 (2015).

19. Shi, W., Bai, C.-M., Zhu, K., Cui, D.-M. & Zhang, C. Brønsted acid-assisted N-alkylation of sulfonamides using ethers as the alkylation reagents. *Tetrahedron* **70,** 434–438 (2014).

20. Saidykhan, A. *et al.* The scope and regioselectivity of intramolecular N-C rearrangements of orthogonally protected sulfonamides, including cyclization to saccharin derivatives. *Tetrahedron Lett.* **58,** 3089–3091 (2017).

21. Colombe, J. R., DeBergh, J. R. & Buchwald, S. L. Synthesis of Heteroaryl Sulfonamides from Organozinc Reagents and 2,4,6-Trichlorophenyl Chlorosulfate. *Org. Lett.* **17,** 3170–3173 (2015).

22. Gonçalves, C. R. *et al.* Unified Approach to the Chemoselective α-Functionalization of Amides with Heteroatom Nucleophiles. *J. Am. Chem. Soc.* **141,** 18437–18443 (2019).

23. Bornholdt, J., Felding, J., Clausen, R. P. & Kristensen, J. L. Ring opening of pymisyl-protected aziridines with organocuprates. *Chem. Eur. J.* **16,** 12474–12480 (2010).

24. Douglas, J., Taylor, J. E., Churchill, G., Slawin, A. M. Z. & Smith, A. D. NHC-Promoted Asymmetric β-Lactone Formation from Arylalkylketenes and Electron-Deficient Benzaldehydes or Pyridinecarboxaldehydes. *J. Org. Chem.* **78,** 3925–3938 (2013).

25. Douglas, J. J., Churchill, G., Slawin, A. M. Z., Fox, D. J. & Smith, A. D. Stereo- and Chemodivergent NHC-Promoted Functionalisation of Arylalkylketenes with Chloral. *Chem. Eur. J.* **21,** 16354–16358 (2015).

26. Allen, A. D., Baigrie, L. M., Gong, L. & Tidwell, T. T. Cyclopropylketenes: preparation and nucleophilic additions. *Can. J. Chem.* **69,** 138–145 (1991).

27. Richmond, E., Duguet, N., Slawin, A. M. Z., Lébl, T. & Smith, A. D. Asymmetric pericyclic cascade approach to spirocyclic oxindoles. *Org. Lett.* **14,** 2762–2765 (2012).

28. Xia, H.-D. *et al.* Photoinduced Copper-Catalyzed Asymmetric Decarboxylative Alkynylation with Terminal Alkynes. *Angew. Chem. Int. Ed.* **59,** 16926–16932 (2020).

29. Roy, A. & Oestreich, M. Metal-Free Hydrosilylation of Ketenes with Silicon Electrophiles: Access to Fully Substituted Aldehyde-Derived Silyl Enol Ethers. *Chem. Eur. J.* **27,** 8273–8276 (2021).

30. Viceriat, A., Marchand, I., Carret, S. & Poisson, J.-F. Synthesis of γ-Lactams by Formal Cycloadditions with Ketenes. *Org. Lett.* **23,** 2449–2454 (2021).

31. Wang, K., Yu, J., Shao, Y., Tang, S. & Sun, J. Forming All-Carbon Quaternary Stereocenters by Organocatalytic Aminomethylation: Concise Access to β2,2 -Amino Acids. *Angew. Chem. Int. Ed.* **59,** 23516–23520 (2020).

32. Le, K. K. A., Nguyen, H. & Daugulis, O. 1-Aminopyridinium Ylides as Monodentate Directing Groups for sp3 C-H Bond Functionalization. *J. Am. Chem. Soc.* **141,** 14728–14735 (2019).

33. Tan, F.-L., Song, R.-J., Hu, M. & Li, J.-H. Metal-Free Oxidative 1,2-Arylmethylation Cascades of N-(Arylsulfonyl)acrylamides Using Peroxides as the Methyl Resource. *Org. Lett.* **18,** 3198–3201 (2016).

34. Radhoff, N. & Studer, A. Functionalization of α-C(sp3)-H Bonds in Amides Using Radical Translocating Arylating Groups. *Angew. Chem. Int. Ed.* **60,** 3561–3565 (2021).

35. Bartalucci, N., Bortoluzzi, M., Zacchini, S., Pampaloni, G. & Marchetti, F. Straightforward formation of carbocations from tertiary carboxylic acids via CO release at room temperature. *Dalton Trans.* **48,** 1574–1577 (2019).
